# Ligand relay catalysis for cobalt-catalyzed sequential hydrosilylation and hydrohydrazidation of terminal alkynes

**DOI:** 10.1038/s41467-022-28285-w

**Published:** 2022-02-03

**Authors:** Yufeng Sun, Jun Guo, Xuzhong Shen, Zhan Lu

**Affiliations:** 1 Department of Chemistry, Zhejiang University, Hangzhou, 310058 China; 2 College of Chemistry, Zhengzhou University, Zhengzhou, 450001 China

**Keywords:** Synthetic chemistry methodology, Homogeneous catalysis

## Abstract

Sequential double hydrofunctionalizationalization of alkynes is a powerful method to construct useful vicinal compounds. Herein, we report a cobalt-catalyzed sequential hydrosilylation/hydrohydrazidation of alkynes to afford 1,2-*N,Si* compounds via ligand relay catalysis. A phenomenon of ligand relay is found that the tridentate anionic *N*-ligand (OPAQ) could capture the cobalt ion from bidentate neutral *P*-ligand (Xantphos) cobalt complex. This protocol uses three abundant chemical feedstocks, alkynes, silanes, and diazo compounds, and also features operationally simple, mild conditions, low catalyst loading (1 mol%), and excellent functional group tolerance. The 1,2-*N,Si* compounds can be easily further derivatized to afford various substituted silane derivatives via *Si-H* functionalization, alcohols via Fleming-Tamao oxidation, free amines and amides via *N-N* bond cleavage and protection. The asymmetric reaction could also be carried out to afford chiral products with up to 86% *ee*. The ligand relay has been supported by control experiments and absorption spectra.

## Abstract (web summary)

In organic chemistry, performing sequential catalytic cycles with a single catalyst improves efficiency. Here the authors present a methodology to functionalize alkynes with nitrogen and silicon atoms, through two catalytic cycles with a homogeneous cobalt catalyst, which is bound to different ligands in each cycle.

## Introduction

**Fig. 1:**
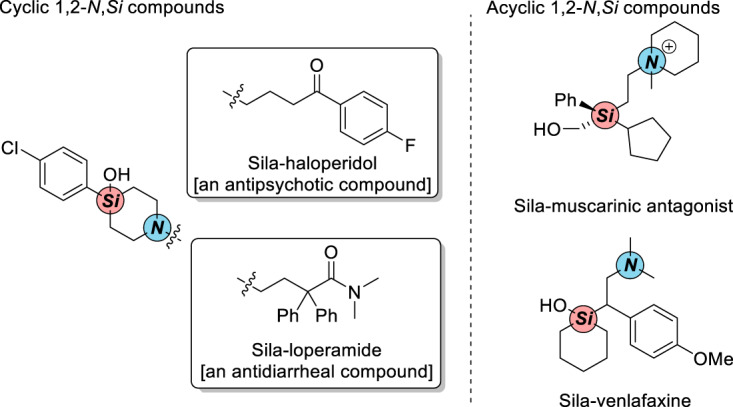
Representative 1,2-*N,Si* compounds in Pharmaceuticals. Cyclic and acyclic 1,2-*N,Si* compounds in pharmaceuticals.

Organosilicon compounds have been widely used in materials science^1^ as well as agrochemistry and organic synthesis^2,3^, also play a growing role in medicinal chemistry as silasubstitution or bioisosteres of carbon or other moieties due to slight physical and electronic alterations^4,5^. Vicinal amino and silyl (1,2-*N,Si*) compounds as an important subclass of organosilicon compounds simultaneously bearing amino and silyl groups have presented in various biologically active compounds such as sila-haloperidol and sila-venlafaxine (Fig. 1)^6–8^.

**Fig. 2:**
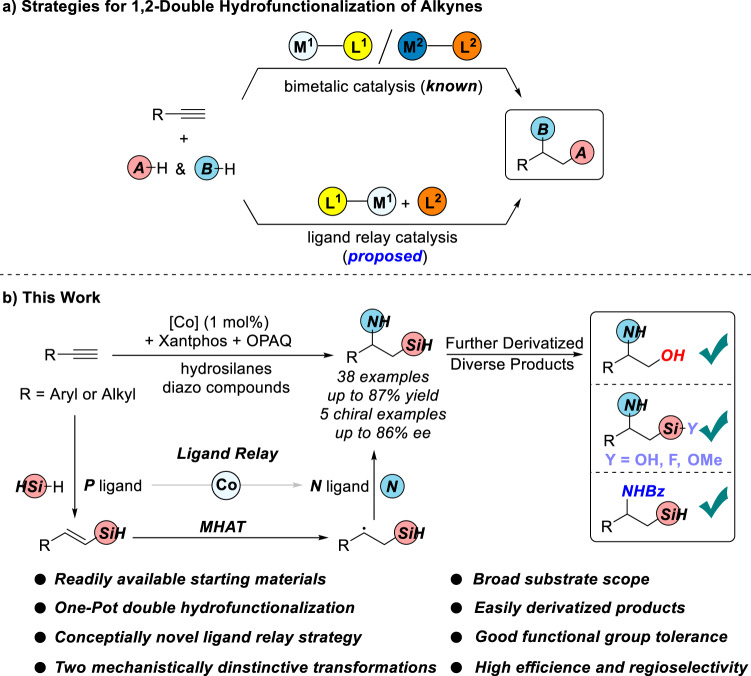
Sequential strategies for double hydrofunctionalization of alkynes to construct 1,2-*N,Si* Compounds. **a** Strategies for 1,2-double hydrofunctionalization of alkynes. **b** Cobalt-catalyzed sequential hydrosilylation/hydrohydrazidation of terminal alkynes via ligand relay catalysis.

Sequential double hydrofunctionalization of readily available alkynes could atom- and pot-economically install two functional groups^9,10^. For two transformations with same catalytic species, one metal catalyst could achieve the sequential reactions^11–20^. For two mechanistically distinctive transformations, a strategy of bimetallic catalysis in which two different metal catalysts respectively play an individual role has been usually employed, however, compatibility issues of two metal catalysts used to limit their utility (Fig. 2a). Ligand exchange is a well-known fundamental elementary reaction in organometallic chemistry^21^. To avoid compatibility issues and also achieve metal economy, a strategy of ligand exchange by using mono metal and two ligands could be proposed in which the former ligand metal complex for catalyzing the first-step reaction would undergo ligand exchange after adding the latter ligand to catalyze the second-step reaction (Fig. 2a)^22–33^. Two reactions would not or slightly interrupt with each other. This one-way ligand exchange for catalyzing two mechanistically distinctive transformations could be called as ligand relay catalysis. However, to the best of our knowledge, this ligand relay catalysis has not been used in sequential double hydrofunctionalization of alkynes.

Sequential double hydrofunctionalization of alkynes could be potentially used to access 1,2-*N,Si* compounds. Nevertheless, sequential hydrosilylation/hydrohydrazidation of alkynes to deliver 1-amino-2-silylalkanes has not yet been reported. There are several challenges: (1) inhibiting the side- reactions in sequential transformations, including dihydrosilylation^11–13^ and hydrogenation^14,15^; (2) controlling the compatibility of two reactions in one pot as well as one product bearing both *N-H* bond and *Si-H* bond; (3) achieving high regioselectivity to reduce the possibility of *gem*-substituted undesired products^16–18^, and improving the efficiency of reactions to make the transformation synthetically useful.

Our group is quite interested in earth-abundant transition-metal-catalyzed reactions of alkenes and alkynes^11–15,34–37^, here, we report a cobalt-catalyzed chemo- and regioselective sequential hydrosilylation/hydrohydrazidation of terminal alkynes via ligand relay catalysis with hydrosilanes and diazo compounds to afford vicinal amino and silyl products bearing both *N-H* bond and *Si-H* bond which could be further derivatized (Fig. 2b).

## Results

### Reaction optimization

**Fig. 3:**
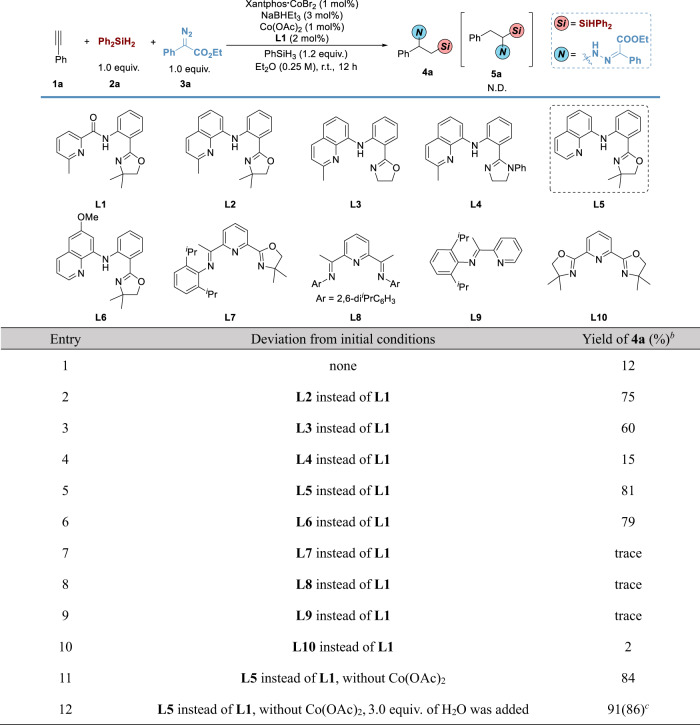
Optimization of Sequential Hydrosilylation/Hydrohydrazidation of Terminal Alkynes via Ligand Relay Catalysis. ^*a*^Reaction conditions: **1a** (0.3 mmol), **2a** (1.0 equiv.), **3a** (1.0 equiv.), Xantphos•CoBr_2_ (1 mol%), NaBHEt_3_ (3 mol%), Co(OAc)_2_ (1 mol%), **L1** (2 mol%) and PhSiH_3_ (1.2 equiv.) in Et_2_O (1.2 mL) at room temperature under nitrogen for 12 h. ^*b*^Yield was determined by ^1^H NMR using TMSPh as an internal standard. ^*c*^Isolated yield was in parentheses. TMSPh = (Trimethylsilyl)benzene.

We began our investigation on sequential hydrosilylation/hydrohydrazidation of alkynes with selected initial reaction conditions, using ethynyl benzene **1a** as a model substrate, diphenyl silane **2a** as a silicon source, ethyl 2-diazo-2-phenylacetate **3a** as a nitrogen source. Based on our previous studies on the cobalt-catalyzed sequential reaction of alkynes^14^, various cobalt complexes for alkyne hydrosilylation have been tested as a solo catalyst for the sequential reaction, however, reactions were messy and fewer desired products have been obtained. To achieve two reactions in one pot, a combined catalytic system using two different catalysts has been tested. One catalytic system is using Xantphos•CoBr_2_ and activator NaBHEt_3_ for the first hydrosilylation step^38,39^. The other catalytic system is using *N*-(2-(4,4-dimethyl-4,5-dihydrooxazol-2-yl)phenyl)-6-methylpicolinamide (OPPA) **L1** and Co(OAc)_2_ for the second hydrohydrazidation step^36^. The combined catalytic system using PhSiH_3_ as a hydrogen donor in a solution of Et_2_O could promote the sequential hydrosilylation/hydrohydrazidation of alkynes to access 1-amino-2-silylalkane **4a** in 12% yield (**entry 1**, Fig. 3). The use of different quinoline amine scaffolds improved the reactivity of reaction drastically (**entries 2-6**). The optimized ligand was *N*-oxazolinylphenyl 8-aminoquinoline (OPAQ) **L5**, delivering **4a** in 81% yield. Altering several *N*-containing classical ligands such as OIP, PDI, PI, and Pybox, only trace amounts of **4a** could be observed (**entries 7-10**). It is worth noting that this transformation in the absence of Co(OAc)_2_ could smoothly occur, delivering **4a** in 84% yield, which presented a unique phenomenon of ligand relay (**entry 11**). With 3 equiv. of H_2_O as promotor, the reaction smoothly afforded **4a** in 86% isolated yield (**entry 12**). The standard conditions are as shown in **entry 12**.

### Substrate scope

**Fig. 4:**
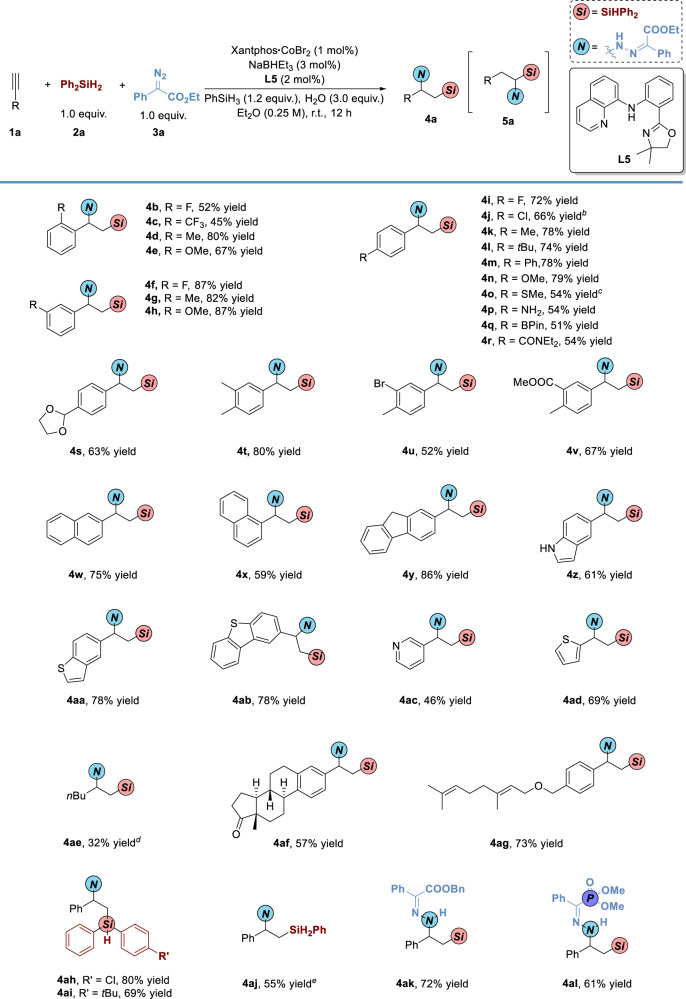
Substrate Scope of Sequential Hydrosilylation/Hydrohydrazidation of Terminal Alkynes via Ligand Relay Catalysis. ^*a*^Reaction conditions: **1** (0.3 mmol), **2** (1.0 equiv.), **3** (1.0 equiv.), Xantphos•CoBr_2_ (1 mol %), NaBHEt_3_ (3 mol %), **L5** (2 mol %), PhSiH_3_ (1.2 equiv.), and H_2_O (3.0 equiv.) in Et_2_O (1.2 mL) under nitrogen at room temperature for 12 h, isolated yield of **4** with complete regioselectivity without additional indications. ^*b*^Stirred in THF, without H_2_O. ^*c*^Stirred for 24 h. ^*d*^**L11** (see Supplementary Information) was instead of **L5**, PhMeSiH_2_ was instead of PhSiH_3_, without H_2_O and stirred for 24 h (7/1 *rr*). ^*e*^DPEphos•CoBr_2_ was instead of Xantphos•CoBr_2_, PhSiH_3_ was instead of Ph_2_SiH_2_.

With the optimized conditions in hand, substrate scope was explored as shown in Fig. 4. Substituents at *ortho*- and *meta*-positions on aryl rings can be well accommodated (**4b**-**4h**). Various functional groups, such as halo, ether, thiol, ether, free amine, Bpin, amide, acetal, and ester, could be tolerated (**4i**-**4v**). Substrates bearing fused rings and heterocycles, such as naphthyl, fluorenyl, indolyl, thiophene, benzothiophene, and pyridine, were also compatible in this system delivering corresponding products in 46-86% yields (**4w**-**4ad**). The alkyl substituted 1-amino-2-silylalkanes could hardly be observed in the process of aminosilylation of alkenes^40^ or ring-opening *C(sp*^3^*)-Si* cross-coupling of aziridines^41–43^, and it was challenging to obtain these compounds. Preliminary study found that hex-1-yne could be converted into **4ae** in 32% yield (7/1 *rr*) under modified conditions. Aryl alkynes containing bioactive skeletons like estrone and geraniol could undergo the reaction to afford **4af** and **4ag** in 57-73% yields, which exhibited the underlying feasibility in late-stage modification of complicated molecules. Various hydrosilane, *para*-chloride and *tert*-butyl substituents diphenyl silanes and phenylsilane, could participate in this transformation smoothly to deliver **4ah** and **4ai** in 69-80% yields and **4aj** bearing two silicon-hydrogen bonds in 55% yield. The diazo compounds with benzyl ester and dimethyl phosphate were also suitable for the system under standard conditions, delivering **4ak** in 72% yield and **4al** bearing three hetero atoms *P, N*, and *Si* in 61% yield.

**Fig. 5:**
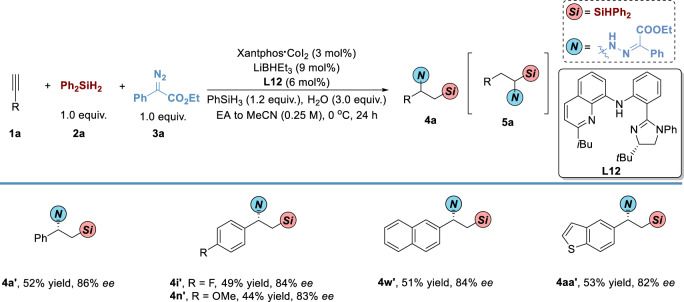
Substrate Scope of Asymmetric Sequential Hydrosilylation/Hydrohydrazidation of Alkynes via Ligand Relay Catalysis. ^*a*^Reaction conditions: **1** (0.3 mmol), **2** (1.0 equiv.), **3** (1.0 equiv.), Xantphos•CoI_2_ (1 mol %), LiBHEt_3_ (3 mol %), **L12** (2 mol %), PhSiH_3_ (1.2 equiv.), and H_2_O (3.0 equiv.) in EA to MeCN (1.2 mL) under nitrogen at 0 ^o^C for 24 h, isolated yield of **4** with complete regioselectivity without additional indications. The details can be found in the Supplementary Information.

To the best of our knowledge, the sequential hydrosilylation/hydrohydrazidation of alkynes for the synthesis of chiral vicinal *N,Si* compounds in an enantioselective manner has scarcely been reported. Thus, We preliminarily conducted asymmetric transformation of alkynes to afford chiral 1,2-*N,Si* compounds in moderate yields with good *ee* (Fig. 5). Under the optimized conditions, the standard substrates could be transformed to the chiral product in 52% yield with 86% *ee*. Both electron-withdrawing and electron-donating groups could be tolerated in the catalytic system, delivering the end-product in moderate yields with good *ee*. Alkynes bearing 2-naphthyl and benzothiophenyl underwent the reaction smoothly in 51% and 53% yields with 84% and 82% *ee*, respectively.

### Gram-Scale reaction and synthetic applications

**Fig. 6:**
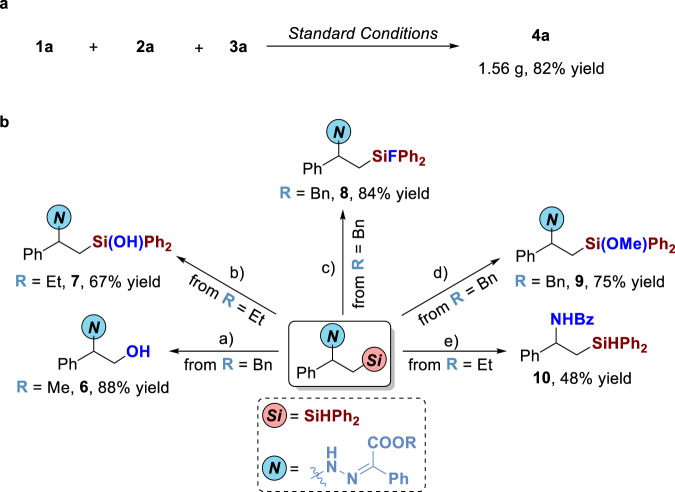
Gram-scale reaction and synthetic applications. **a** Gram-scale reaction. **b** Synthetic applications. Reagents and conditions: (a) KF (4.0 equiv.), KHCO_3_ (4.0 equiv.), H_2_O_2_ (23.0 equiv.), MeOH/THF = 1/1, 65 ^o^C, 12 h; (b) KHCO_3_ (1.0 equiv.), H_2_O_2_ (18.0 equiv.), MeOH/THF = 1/1, r.t., 12 h; (c) CuCl_2_ (4.0 equiv.), CuI (18 mol%), KF (2.4 equiv.), THF, r.t., 12 h; (d) Pd/C (10 wt%), MeOH/THF = 4/1, r.t., 12 h; (e) i) SmI_2_ (3.4 equiv.), MeOH/THF = 1/1, r.t., 12 h; ii) BzCl (3.0 equiv.), Et_3_N (4.0 equiv.), THF, r.t., 2 h. The details can be found in the Supplementary Information.

The gram-scale reaction could be smoothly conducted to access **4a** in 82% yield (Fig. 6). The 1,2-*N,Si* compounds could be further derivatized via *C-Si* cleavage, *Si-H* transformation, and *N-N* cleavage. Firstly, these compounds could be easily transferred to alcohol **6** in excellent yield via Fleming-Tamao oxidation. Then, 1,2-*N,Si* compounds could be smoothly transformed to silanol **7**, fluorosilane **8**, and siloxane **9** via *Si-H* transformation in 67-84% yields. The product can also be converted into amide silane **10** in 48% yield via mild *N-N* bond cleavage and benzoyl group protection. These compounds containing both *N-H* bond and *Si-H* bond might be interesting and potentially useful, due to that, generally, *N-H* bond could provide a proton and *Si-H* bond could afford a hydride as a polar opposite reagent.

### Mechanistic studies

**Fig. 7:**
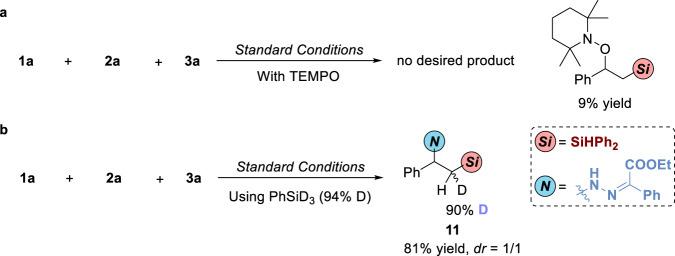
Radical trapping and Deuterium-labeling experiments. **a** Radical trapping experiment using TEMPO as radical trapping reagent. **b** Deuterium-labeling experiment using PhSiD_3_ as deuterated reagent.

**Fig. 8:**
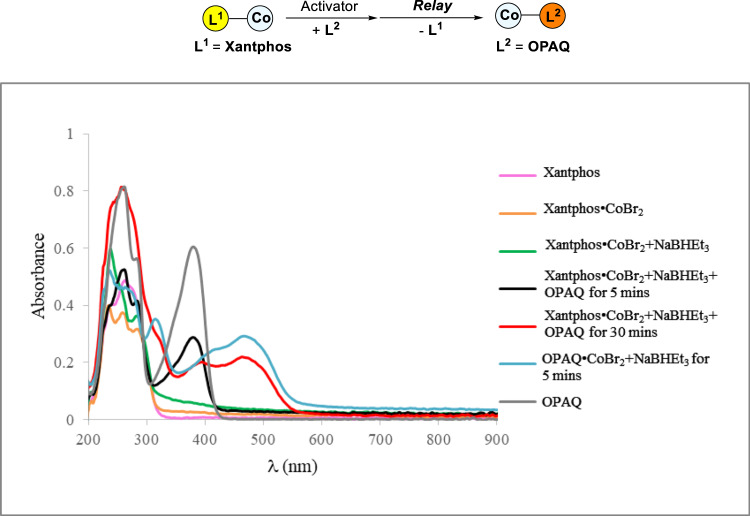
Absorption spectra of ligand relay process between Xantphos and OPAQ. The absorption spectra experiment is conducted under nitrogen using optic fiber spectrophotometer.

To gain insight into the mechanism, two control experiments were conducted to illuminate possible mechanism (Fig. 7). With the existence of TEMPO, no desired product can be observed. The benzyl radical generated from MHAT process was trapped by TEMPO delivering the product in 9% yield, indicating that the reaction may go through radical process. The deuterium-labeling experiment was also conducted using PhSiD_3_ as a D source to afford **11** in 81% yield with 90% D in the adjacent methylene of silyl group (*dr* = 1/1), indicating that radical process may exist in this reaction. To further understand the ligand relay process, the absorption measurement was conducted (Fig. 8). Initially, we added NaBHEt_3_ to the solution of Xantphos•CoBr_2_ and it did not change the shape of absorption peaks. When adding OPAQ to the mixture, in the first 5 mins, there was no change in absorption peaks. A broad peak was observed at 468 nm in 30 mins (red line) which is cater to the absorption peak of OPAQ•CoBr_2_ activated by NaBHEt_3_ at 467 nm (blue line) indicating that it takes a period of time for OPAQ to coordinate with cobalt, the catalytic active species would emerge and keep active via ligand exchange process under N_2_ atmosphere while no peaks will emerge at 468 nm conducting the reaction under air (see Supplementary Information, Supplementary Figs. 14–15). Substrates will not influence the ligand exchange process (see Supplementary Information, Supplementary Figs. 14–17).

**Fig. 9:**
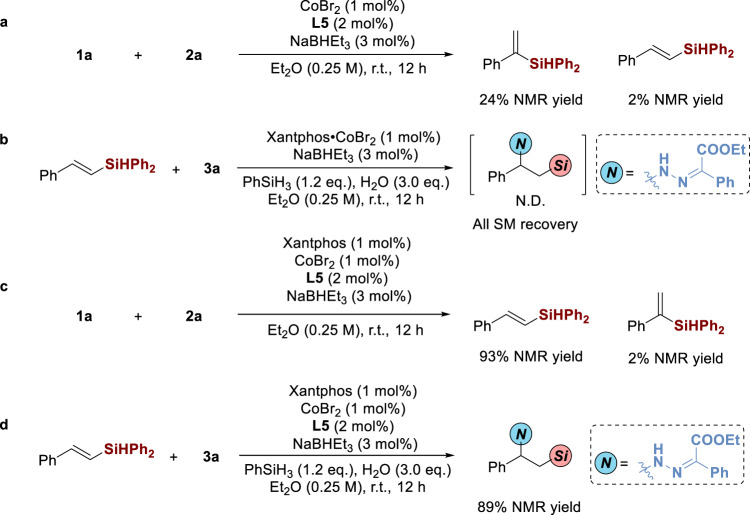
Control experiments. **a** Investigation of hydrosilylation step using CoBr_2_ and **L5**. **b** Investigation of hydrohydrazidation step using Xantphos•CoBr_2_. **c** Investigation of hydrosilylation step using a combination of Xantphos, **L5**, and CoBr_2_. **d** Investigation of hydrosilylation step using a combination of Xantphos, **L5**, and CoBr_2_.

Several control experiments were also conducted. Firstly, the *anti*-Markovnikov hydrosilylation of alkynes could not successfully occur, delivering the α-adduct as the main product instead. Herein, Xantphos was essential for the first hydrosilylation step (Fig. 9a). Next, hydrohydrazidation of alkenyl silane failed in the absence of OPAQ, indicating that the relay process between Xantphos and OPAQ would promote the second step (Fig. 9b). From Fig. 9c we could know that OPAQ would slightly influence the *anti*-Markovnikov hydrosilylation step and 2% NMR yield of α-adduct was observed. In the end, Xantphos barely affected the hydrohydrazidation step, affording the 1-amino-2-silylalkanes in good yield (Fig. 9d).

**Fig. 10:**
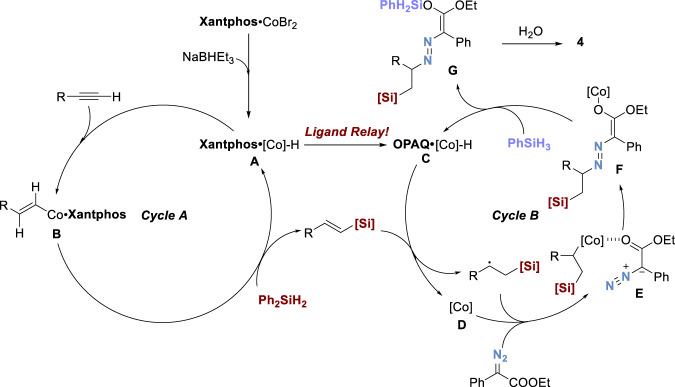
Proposed mechanism. Proposed reaction pathway starts from Xantphos•Co-H species to catalyze the hydrosilylation step, followed by the key ligand relay process to generate the OPAQ•Co-H to catalyze the hydrohydrazidation step.

Based on the control experiments and previous studies^38,39,44–49^, we envisioned a possible mechanism shown in Fig. 10. Initially, Xantphos•CoBr_2_ was activated by NaBHEt_3_ to generate the cobalt hydride intermediate **A** which would coordinate with the alkyne. Then a following *anti*-Markovnikov type alkyne insertion into the cobalt hydride species gave the *β*-vinyl cobalt intermediate **B** which could undergo σ-bond metathesis with diphenyl silane to deliver the vinyl silane. The regenerated intermediate **A** would undergo a ligand exchange process to deliver OPAQ cobalt hydride species **C**. Selecting the appropriately exchangeable ligand to replace the former ligand is the key point in the ligand relay process. Here, the tridentate anionic *N*-ligand (OPAQ) presented a better coordination effect with cobalt ion than bidentate neutral *P*-ligand (Xantphos), ensuring the fluency of hydrohydrazidation of alkenyl silanes. The posterior reaction will not be influenced by the former metal catalyst which already loses the central metal ion. The newly emerging intermediate **C** could perform through metal hydride hydrogen atom transfer (MHAT)^48^ to afford a radical intermediate and cobalt intermediate **D** which could react with each other in the presence of diazo compound to form the intermediate **E**. The cobalt species **E** could then undergo alkyl group migration to deliver the azo enolate cobalt intermediate **F**, followed by *σ*-bond metathesis with PhSiH_3_ to give the silyl enol ester intermediate **G** and regenerate cobalt hydride intermediate **C**. In the presence of H_2_O, intermediate **G** would go through sequential hydrolysis and isomerization to deliver final product.

## Discussion

In summary, we reported a cobalt-catalyzed sequential hydrosilylation/hydrohydrazidation of readily available terminal alkynes with hydrosilanes and diazo compounds to deliver 1,2-*N,Si* compounds with good functional group tolerance. These products bearing both *N-H* bond and *Si-H* bond can be easily transformed to hydrazino alcohol, diversified silicon-substituted hydrazino silanes, and amide silane. Two mechanistically distinctive transformations including cobalt-catalyzed alkyne insertion for alkyne hydrosilylation and the HAT process for hydrohydrazidation of alkenyl silane could be conducted using 1 mol% of cobalt as a solo metal in one pot. The asymmetric reaction could also be carried out to afford chiral products with up to 86% *ee*. A phenomenon of ligand relay is found to deliver cobalt from diphosphine ligand to *NNN*-tridentate ligand which is also illustrated by absorption measurement. Ligand relay could offer an opportunity as a potentially powerful strategy for metal-catalyzed sequential reactions using a solo metal. Further studies on ligand relay guided sequential reactions and the synthesis of organosilicon compounds will be continuously explored in our laboratory.

## Methods

### Materials

For NMR spectra of compounds in this manuscript, see Supplementary Information. For gram-scale reaction, see Supplementary Figure 1. For synthesis of amide silane, see Supplementary Fig. 2. For radical trapping experiment, isotopic labeling experiment, time course study, control experiment and absorption measurement, see Supplementary Figs. 3–17 and Table 1.

### Standard conditions A for the synthesis of 1,2-*N,Si* compounds

To a 25 mL Schlenk flask equipped with a magnetic stirrer and a flanging rubber plug was dried with flame under vacuum. When cooled to ambient temperature (10–25 ^o^C), it was vacuumed and flushed with N_2_. This degassed procedure was repeated for three times. To the flame-dried Schlenk flask Xantphos•CoBr_2_ complex (0.0030 mmol, 1 mol%), 1.2 mL (0.25 M) of Et_2_O and diphenylsilane (0.30 mmol, 1.0 equiv.) (or other silanes) were added by dropwise sequentially. After that, NaBHEt_3_ (9 μL, 1.0 M in THF, 0.0090 mmol) and alkyne (0.30 mmol, 1.0 equiv.) were added to the mixture sequentially and stirred for 5 s, and then **L5** (0.0060 mmol, 2 mol%), phenylsilane (0.36 mmol, 1.2 equiv.), phenyldiazoacetate (0.30 mmol, 1.0 equiv.) (or other aryldiazoacetates), H_2_O (0.90 mmol, 3.0 equiv.) were added by dropwise sequentially. Pinholes were sealed with silicone grease and the flanging rubber plug was wrapped with sealing film tightly. The mixture was stirred at ambient temperature for 12 h, and then quenched by 5 mL of petroleum ether (PE) and stirred for 5 min until catalyst precipitated. The resulting solution was filtered through a pad of silica gel and the Schlenk flask and silica gel were washed by PE/EtOAc (5/1) (15 mL× 3) (or other suitable solvent). The combined filtrates were concentrated *in vacuo*. NMR yield was monitored by ^1^H NMR analysis using TMSPh as internal standard. The crude mixture was purified by short flash column chromatography to give the corresponding product.

## Supplementary information

Supporting Information

## Data Availability

The authors declare that the data Supplementary the findings of this study are available within the paper and its Supplementary Information file. The experimental procedures and characterization of all new compounds are provided in the Supplementary Information. Source data are provided with this paper.

## Source data

Source Data

## References

[1] Ciriminna R (2013). The sol-gel route to advanced silica-based materials and recent applications. Chem. Rev..

[2] Ojima, I. In *The Chemistry of Organic Silicon Compounds*; (eds. Patai, S., Rappoport, Z.) (Wiley: Chichester, 1989).

[3] Denmark SE, Sweis RF (2002). Design and implementation of new, silicon-based, cross-coupling reactions: importance of silicon-oxygen bonds. Acc. Chem. Res..

[4] Min GK, Hernández D, Skrydstrup T (2013). Efficient routes to carbon-silicon bond formation for the synthesis of silicon-containing peptides and azasilaheterocycles. Acc. Chem. Res..

[5] Franz AK, Wilson SO (2013). Organosilicon molecules with medicinal applications. J. Med. Chem..

[6] Meanwell NA (2011). Synopsis of some recent tactical application of bioisosteres in drug design. J. Med. Chem..

[7] Barraza SJ, Denmark SE (2018). Synthesis, reactivity, functionalization, and ADMET properties of silicon-containing nitrogen heterocycles. J. Am. Chem. Soc..

[8] Ramesh R, Reddy DS (2018). Quest for novel chemical entities through incorporation of silicon in drug scaffolds. J. Med. Chem..

[9] Zeng X (2013). Recent advances in catalytic sequential reactions involving hydroelement addition to carbon-carbon multiple bonds. Chem. Rev..

[10] Cheng Z, Guo J, Lu Z (2020). Recent advances in metal-catalysed asymmetric sequential double hydrofunctionalization of alkynes. Chem. Commun..

[11] Guo J, Wang H, Xing S, Hong X, Lu Z (2019). Cobalt-catalyzed asymmetric synthesis of *gem*-Bis(silyl)alkanes by double hydrosilylation of aliphatic terminal alkynes. Chem.

[12] Cheng Z (2019). Highly regioselective sequential 1,1-dihydrosilylation of terminal aliphatic alkynes with primary silanes. Chin. J. Chem..

[13] Hu M-Y, Lian J, Sun W, Qiao T-Z, Zhu S-F (2019). Iron-catalyzed dihydrosilylation of alkynes: efficient access to geminal bis(silanes). J. Am. Chem. Soc..

[14] Guo J, Shen X, Lu Z (2017). Regio- and enantioselective cobalt-catalyzed sequential hydrosilylation/hydrogenation of terminal alkynes. Angew. Chem. Int. Ed..

[15] Chen J, Shen X, Lu Z (2020). Cobalt-catalyzed Markovnikov selective sequential hydrogenation/hydrohydrazidation of aliphatic terminal alkynes. J. Am. Chem. Soc..

[16] Niljianskul N, Zhu S, Buchwald SL (2015). Enantioselective synthesis of alpha-aminosilanes by copper-catalyzed hydroamination of vinylsilanes. Angew. Chem., Int. Ed..

[17] Nishino S, Hirano K, Miura M (2020). Cu-catalyzed reductive *gem*-difunctionalization of terminal alkynes via hydrosilylation/hydroamination cascade: concise synthesis of α-aminosilanes. Chem. Eur. J..

[18] Gao D-W (2020). Cascade CuH-catalysed conversion of alkynes into enantioenriched 1,1-disubstitued products. Nat. Catal..

[19] Jin S, Liu K, Wang S, Song Q (2021). Enatioselective cobalt-catalyzed cascade hydrosilylation and hydroboration of alkynes to access enantioenriched 1,1-silylboryl alkanes. J. Am. Chem. Soc..

[20] You Y, Ge S (2021). Cobalt-catalyzed one-pot asymmetric difunctionalization of alkynes to accesss chiral *gem*-(Borylsilyl)alkanes. Angew. Chem. Int. Ed..

[21] *Organotransition Metal Chemistry: From Bonding to Catalysis* (ed. Hartwig, J.) (University Science Books: Sausalito, CA, 2010).

[22] *The Organometallic Chemistry of the Transition Metals,* Sixth Edition (ed. Crabtree, R. H.) (John Wiley: Hoboken, NJ. 2014).

[23] Duursma A (2003). First examples of improved catalytic asymmetric C–C bond formation using the monodentate ligand combination approach. Org. Lett..

[24] Reetz MT, Sell T, Meiswinkel A, Mehler G (2003). A new principle in combinatorial asymmetric transition-metal catalysis: mixtures of chiral monodentate P ligands. Angew. Chem. Int. Ed..

[25] Reetz MT (2008). Combinatorial transition-metal catalysis: mixing monodentate ligands to control enantio-, diastereo-, and regioselectivity. Angew. Chem. Int. Ed..

[26] Pignataro L (2009). Combination of a binaphthol-derived phosphite and a *C1*-symmetric phosphinamine generates heteroleptic catalysts in Rh- and Pd-mediated reactions. Chem. Commun..

[27] Teichert JF, Feringa BL (2010). Phosphoramidites: privileged ligands in asymmetric catalysis. Angew. Chem. Int. Ed..

[28] Wieland J, Breit B (2010). A combinatorial approach to the identification of self-assembled ligands for rhodium-catalysed asymmetric hydrogenation. Nat. Chem..

[29] Fors BP, Buchwald SL (2010). A multiligand based Pd catalyst for C-N cross-coupling reactions. J. Am. Chem. Soc..

[30] Gao S, Liu Y, Ma S (2011). CuCl-catalyzed aerobic oxidation of 2,3-allenols to 1,2-allenic ketones with 1:1 combination of phenanthroline and bipyridine as ligands. Beilstein J. Org. Chem..

[31] Sheng J, Ni HQ, Liu G, Li Y, Wang XS (2017). Combinatorial nickel-catalyzed monofluoroalkylation of aryl boronic acids with unactivated fluoroalkyl iodides. Org. Lett..

[32] Liu W (2019). Iron-catalyzed intramolecular amination of aliphatic C–H bonds of sulfamate esters with high reactivity and chemoselectivity. Org. Lett..

[33] Li W (2020). Design of Ru(II)-NHC-diamine precatalysts directed by ligand cooperation: applications and mechanistic investigations for asymmetric hydrogenation. J. Am. Chem. Soc..

[34] Chen J, Guo J, Lu Z (2018). Recent advances in hydrometallation of alkenes and alkynes via the first row transition metal catalysis. Chin. J. Chem..

[35] Chen J, Shen X, Lu Z (2021). Cobalt-catalyzed Markovnikov-type selective hydroboration of terminal alkynes. Angew. Chem. Int. Ed..

[36] Shen X (2020). Ligand-promoted cobalt-catalyzed radical hydroamination of alkenes. Nat. Commun..

[37] Cheng Z (2021). Regio-contollable cobalt-catalyzed sequential hydrisilylation/hydroborylation of arylacetylenes. Angew. Chem. Int. Ed..

[38] Cheng B, Lu P, Zhang H, Cheng X, Lu Z (2017). Highly enantioselective cobalt-catalyzed hydrosilylation of alkenes. J. Am. Chem. Soc..

[39] Wu C, Teo WJ, Ge S (2018). Cobalt-catalyzed (*E*)-selective *anti*-Markovnikov hydrosilylation of terminal alkynes. ACS Catal..

[40] Yang Y (2017). Iron-catalyzed intermolecular 1,2-difunctionalization of styrenes and conjugated alkenes with silanes and nucleophiles. Angew. Chem. Int. Ed..

[41] Fleming, I., Frackenpohl, J. & Ila, H. Cleavage of sulfonamides with phenyldimethylsilyllithium. *J. Chem. Soc., Perkin Trans.* **1**, 1229–1236 (1998).

[42] Takeda Y, Shibuta K, Aoki S, Tohnai N, Minakata S (2019). Catalyst-controlled regiodivergent ring-opening *C(sp*^*3*^*)-Si* bond-forming reactions of 2-arylaziridines with silylborane enabled by synergistic palladium/copper dual catalysis. Chem. Sci..

[43] Yi H, Oestreich M (2019). Regiodivergent and stereospecific aziridine opening by copper-catalyzed addition of silicon Grignard reagents. Chem. Eur. J..

[44] Li W (2011). New electrophilic addition of α-diazoesters with ketones for enantioselective C–N bond formation. J. Am. Chem. Soc..

[45] Zheng J, Qi J, Cui S (2016). Fe-catalyzed olefin hydroamination with diazo compounds for hydrazone synthesis. Org. Lett..

[46] Waser J, Gaspar B, Nambu H, Carreira EM (2006). Hydrazines and azides via the metal-catalyzed hydrohydrazination and hydroazidation of olefins. J. Am. Chem. Soc..

[47] Gui J (2015). Practical olefin hydroamination with nitroarenes. Science.

[48] Green SA (2018). The high chemofidelity of metal-catalyzed hydrogen atom transfer. Acc. Chem. Res..

[49] Gu Q-S, Li Z-L, Liu X-Y (2020). Copper(I)-catalyzed asymmetric reactions involving radicals. Acc. Chem. Res..

