# Supplementary material for: Ligand relay catalysis for cobalt-catalyzed sequential hydrosilylation and hydrohydrazidation of terminal alkynes
Source: Nat Commun. 2022 Feb 3;13:650. doi: 10.1038/s41467-022-28285-w (PMC8813943; doi:10.1038/s41467-022-28285-w)
Supplement: Supplementary file 1 — Supporting Information [file 41467_2022_28285_MOESM1_ESM.pdf]

Supplementary Information

Ligand Relay Catalysis: Sequential Hydrosilylation/Hydrohydrazidation of Terminal Alkynes

Yufeng Sun[1], Jun Guo[1], Xuzhong Shen[1] and Zhan Lu[1,2]*

[1]*Department of Chemistry, Zhejiang University, Hangzhou 310058, China*

[2] *College of Chemistry, Zhengzhou University, Zhengzhou 450001, China.*

Table of contents

Supplementary Methods.....3

I. General Information.....3

II. Synthesis of Substrates and Ligands.....3

III. Sequential Hydrosilylation/Hydrohydrazidation of Alkynes.....9

IV. Asymmetric Sequential Hydrosilylation/Hydrohydrazidation of Alkynes.....37

V. Gram-Scale Reaction.....41

VI. Further Derivatitions.....42

VII. Mechanistic Studies and Control Experiments.....46

VIII. NMR Spectra.....58

IX. HPLC Spectra.....179

Supplementary Reference.....184

Supplementary Methods

I. General Information

THF, Et_2O, toluene and dioxane were distilled from sodium benzophenone ketyl prior to use. $Pd_2(dba)_3$ (98%) was purchased from Zhejiang Metallurgical Research Institute and used as received. 1,1'-Bis(diphenylphosphino)ferrocene (dppf) (98%) and $CoBr_2$ (97%) was purchased from Energy and used as received. Sodium triethylborohydride solution (1 M in THF) was purchased from Sigma-Aldrich and used as received. The other commercially available chemicals were used as received. Phenylsilane and diphenylsilane were prepared according to the previously reported procedures.[1] NMR spectra were recorded on a Bruker-400 instrument, a Bruker-500 instrument and a Wuhan Zhongke-Niujin-400 instrument. ^{1}H NMR chemical shifts were referenced to tetramethylsilane signal (0 ppm), ^{13}C NMR chemical shifts were referenced to the solvent resonance (77.00 ppm, $CDCl_3$). ^{2}D NMR chemical shifts were referenced to $CDCl_3$ signal (7.26 ppm). The following abbreviations (or combinations) were used to explain multiplicities: s = singlet, d = doublet, t = triplet, m= multiplet, br = broad, q = quadruplet. Melting points were obtained using a WRR melting point apparatus (Laboratory Devices, Shanghai Precision & Scientific Instrument Co., Ltd.). High-resolution mass spectra (HRMS) were recorded on ESI-TOF or EI. IR spectra were recorded on a Perkin-Elmer Spectrum One FTIR spectrometer with diamond ATR accessory. Absorption spectrum was recorded on optic fiber spectrophotometer (QE-Pro, Ocean Optics).

II. Synthesis of Substrates and Ligands.

Alkynes were prepared according to Sonogashira-Coupling reaction or the previously reported procedures.[2-9] **L1**[10], **L5**[10], **L7**[11], **L8**[12], **L9**[13], **L10**[14], **L11**[15], and **L12**[10] were prepared according to the previously reported procedures.

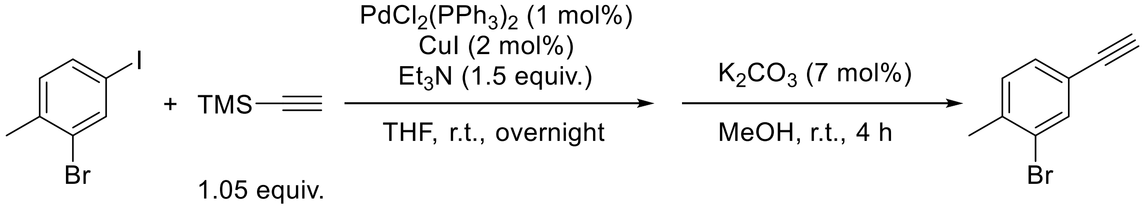

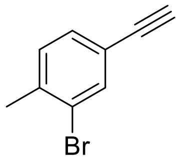

2-Bromo-4-ethynyl-1-methylbenzene (1u). To a 100 mL flame-dried Schlenk flask, $PdCl_2(PPh_3)_2$ (0.12 g, 0.17 mmol), CuI (0.0642 g, 0.34 mmol), THF (60 mL), 2-bromo-4-iodo-1-methylbenzene (5.00 g, 16.9 mmol), Et_3N (3.5 mL, 0.73 g/mL, 25 mmol) and ethynyltrimethylsilane (1.74 g, 17.7 mmol) were added in sequence under the atmosphere of nitrogen. The solution was stirred at room temperature overnight, quenched by PE and filtered through a pad of silica gel. The schlenk flask and silica gel were washed by PE/EtOAc (20/1) (30 mL × 3). The combined filtrates were concentrated *in vacuo*. Then MeOH (50 mL) and K_2CO_3 (0.16 g, 1.2 mmol) were added into the residue and the solution was stirred at room temperature for 8 h. The solution was filtered through a pad of silica gel. All volatiles were removed from the solution *via* rotary evaporation to give a brown sticky oil. And the residue was purified by flash column chromatography using PE as the eluent to give 2.28 g (11.7 mmol, 69% yield) of the title compound as a yellow oil. IR (cm^{-1}): 3292, 2110, 1548, 1485, 1451. ^1H NMR: (400 MHz, $CDCl_3$) δ 7.66 (d, J = 1.2 Hz, 1H), 7.32 (dd, J = 8.0, 1.2 Hz, 1H), 7.16 (d, J = 8.0 Hz, 1H), 3.06 (s, 1H), 2.39 (s, 3H); ^{13}C NMR: (100 MHz, $CDCl_3$) δ 139.0, 135.6, 130.9, 130.5, 124.4, 121.2, 82.1, 77.7, 22.9; HRMS (EI) calculated for $[C_9H_7Br]^+$ $[M]^+$ requires m/z 193.9731, found m/z 193.9733.

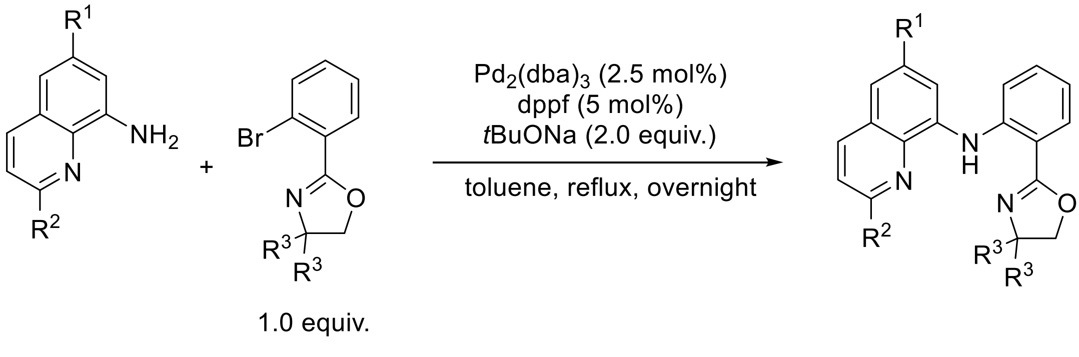

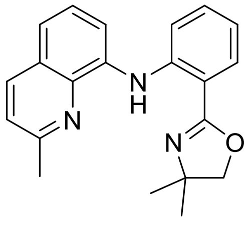

***N*-(2-(4,4-dimethyl-4,5-dihydrooxazol-2-yl)phenyl)-2-methylquinolin-8-amine (L4).** To a 50

mL flame-dried Schlenk flask, $Pd_2(dba)_3$ (0.0755 g, 0.0825 mmol), dppf (0.0915 g, 0.165 mmol) and toluene (10 mL) were added in sequence under the atmosphere of nitrogen. The solution was stirred for 10 mins. 2-(2-bromophenyl)-4,4-dimethyl-4,5-dihydrooxazole (0.83 g, 3.3 mmol), 2-methylquinolin-8-amine (0.52 g, 3.3 mol), sodium *tert*-butoxide (0.63 g, 6.6 mmol) were added in sequence under the atmosphere of nitrogen. The solution was refluxed overnight, thereafter, cooled down to room temperature. The solution was filtered through a pad of silica gel. All volatiles were removed from the solution *via* rotary evaporation to give a brown sticky oil. And the residue was purified by flash column chromatography using PE/EtOAc (20/1) as the eluent to give 0.93 g (2.8 mmol, 85% yield) of the title compound as a yellow solid. M.p. 113.6-114.1 ^{o}C. IR (cm^{-1}): 2967, 2925, 2089, 1637, 1576, 1531. ^{1}H NMR: (400 MHz, $CDCl_3$) δ 11.72 (brs, 1H), 8.00 (d, J = 8.4 Hz, 1H), 7.89-7.82 (m, 2H), 7.79 (d, J = 7.6 Hz, 1H), 7.40-7.32 (m, 2H), 7.31-7.26 (m, 2H), 6.88-6.81 (m, 1H), 4.07 (s, 2H), 2.79 (s, 3H), 1.45 (s, 6H); ^{13}C NMR: (100 MHz, $CDCl_3$) δ 161.4, 156.6, 143.5, 140.0, 138.7, 136.0, 131.4, 130.0, 127.2, 125.7, 122.2, 118.3, 118.1, 115.2, 113.6, 111.5, 68.1, 28.8, 25.5; HRMS (ESI) calculated for $[C_{21}H_{22}N_3O]^+$ $[M+H]^+$ requires m/z 332.1757, found m/z 332.1759.

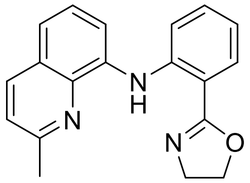

***N*-(2-(4,5-dihydrooxazol-2-yl)phenyl)-2-methylquinolin-8-amine (L3).** To a 50 mL flame-dried Schlenk flask, $Pd_2(dba)_3$ (0.0830 g, 0.091 mmol), dppf (0.0998 g, 0.180 mmol) and toluene (10 mL) were added in sequence under the atmosphere of nitrogen. The solution was stirred for 10 mins. 2-(2-bromophenyl)-4,5-dihydrooxazole (0.81 g, 3.6 mmol), 2-methylquinolin-8-amine (0.57 g, 3.6 mmol), sodium *tert*-butoxide (0.70 g, 7.2 mmol) were added in sequence under the atmosphere of nitrogen. The solution was refluxed overnight, thereafter, cooled down to room temperature. The solution was filtered through a pad of silica gel. All volatiles were removed from the solution *via* rotary evaporation to give a brown sticky oil. And the residue was purified by flash column chromatography using PE/EtOAc (10/1) as the eluent to give 0.72 g (2.4 mmol, 67% yield) of the title compound as a pale yellow solid. M.p. 133.2-135.4 ^{o}C. IR (cm^{-1}): 2925, 1638,

1578, 1531, 1500, 1452. ^{1}H NMR: (400 MHz, $CDCl_3$) δ 11.71 (brs, 1H), 8.00 (d, J = 8.4 Hz, 1H), 7.90 (dd, J = 1.6, 8.0 Hz, 1H), 7.85 (d, J = 8.4 Hz, 1H), 7.78 (d, J = 7.6 Hz, 1H), 7.40-7.34 (m, 2H), 7.32-7.26 (m, 2H), 6.90-6.83 (m, 1H), 4.43-4.35 (m, 2H), 4.29-4.21 (m, 2H), 2.79 (s, 3H); ^{13}C NMR: (100 MHz, $CDCl_3$) δ 164.4, 156.8, 143.6, 138.5, 136.1, 131.6, 130.3, 127.2, 125.8, 122.2, 118.4, 118.2, 115.2, 113.4, 111.7, 66.0, 55.2, 25.5; HRMS (ESI) calculated for $[C_{19}H_{18}N_3O]^+$ $[M+H]^+$ requires m/z 304.1444, found m/z 304.1445.

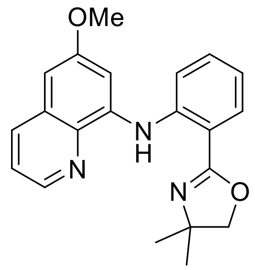

***N*-(2-(4,4-dimethyl-4,5-dihydrooxazol-2-yl)phenyl)-6-methoxyquinolin-8-amine (L6).** To a 25 mL flame-dried Schlenk flask, $Pd_2(dba)_3$ (0.0230 g, 0.025 mmol), dppf (0.0278 g, 0.050 mmol) and toluene (2 mL) were added in sequence under the atmosphere of nitrogen. The solution was stirred for 10 mins. 2-(2-bromophenyl)-4,4-dimethyl-4,5-dihydrooxazole (0.25 g, 1.0 mmol), 6-methoxyquinolin-8-amine (0.17 g, 1.0 mmol), sodium tert-butoxide (0.19 g, 2.0 mmol) were added in sequence under the atmosphere of nitrogen. The solution was refluxed overnight, thereafter, cooled down to room temperature. The solution was filtered through a pad of silica gel. All volatiles were removed from the solution *via* rotary evaporation to give a brown sticky oil. And the residue was purified by flash column chromatography using PE/EtOAc (10/1) as the eluent to give 0.32 g (0.91 mmol, 91% yield) of the title compound as a pale yellow solid. M.p. 130.4-131.4 ^{o}C. IR (cm^{-1}): 2965, 2927, 1638, 1576, 1531, 1453. ^{1}H NMR: (400 MHz, $CDCl_3$) δ 11.80 (brs, 1H), 8.74-8.70 (m, 1H), 7.99 (d, J = 8.4 Hz, 1H), 7.87 (m, 2H), 7.44 (d, J = 2.0 Hz, 1H), 7.41-7.33 (m, 2H), 6.92-6.85 (m, 1H), 6.61 (d, J = 2.4 Hz, 1H), 4.06 (s, 2H), 3.92 (s, 3H), 1.44 (s, 6H); ^{13}C NMR: (100 MHz, $CDCl_3$) δ 161.3, 158.4, 145.5, 143.1, 140.6, 137.6, 134.6, 131.5, 129.95, 129.90, 121.9, 118.7, 115.8, 114.1, 103.5, 96.3, 68.1, 55.4, 28.7; HRMS (ESI) calculated for $[C_{21}H_{22}N_3O_2]^+$ $[M+H]^+$ requires m/z 348.1707, found m/z 348.1708.

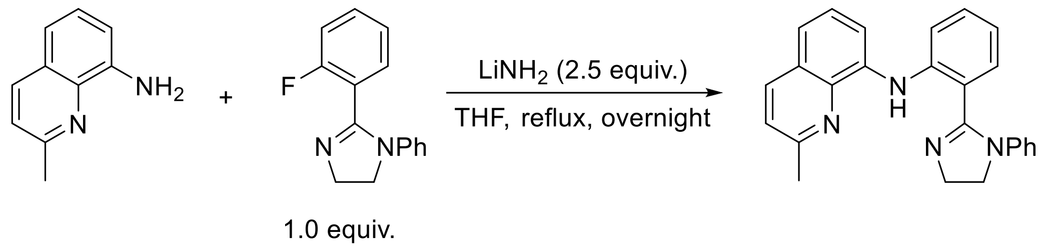

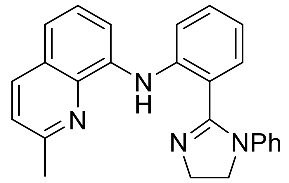

2-Methyl-*N*-(2-(1-phenyl-4,5-dihydro-1*H*-imidazol-2-yl)phenyl)quinolin-8-amine (L4). To a 25 mL flame-dried Schlenk flask, 2-(2-fluorophenyl)-1-phenyl-4,5-dihydro-1*H*-imidazole (0.24 g, 1.0 mmol), 2-methylquinolin-8-amine (0.16 g, 1.0 mmol), lithium amide (0.057 g, 2.5 mmol) and THF (2 mL) were added in sequence under the atmosphere of nitrogen. The solution was refluxed at 90 ^{o}C for 5-10 mins with two-port valve linked to N_2 pipeline due to lots of NH_3 would be released in the initial stage of the reaction. The two-port valve could be closed until the solution color turned from yellow to red and the solution was reposefully refluxed at 90 ^{o}C overnight. Thereafter, the solution was cooled down to room temperature and saturated solution of NH_4Cl (10 mL) was added and extracted with EtOAc (20 mL × 3). The combined organic layers were dried over anhydrous Na_2SO_4, filtered, concentrated to give a red oil. And the residue was purified by flash column chromatography using PE/EtOAc (1/1) as the eluent to give 0.25 g (0.67 mmol, 67% yield) of the title compound as a pale yellow solid. M.p. 94.4-95.0 ^{o}C. IR (cm^{-1}): 3053, 2866, 1573, 1525, 1496, 1453. ^{1}H NMR: (400 MHz, $CDCl_3$) δ 10.30 (brs, 1H), 7.97 (d, J = 8.4 Hz, 1H), 7.75 (d, J = 8.4 Hz, 1H), 7.50 (d, J = 7.6 Hz, 1H), 7.32-7.23 (m, 4H), 7.20-7.15 (m, 1H), 7.10 (m, 2H), 6.92-6.86 (m, 1H), 6.83-6.77 (m, 1H), 6.74 (d, J = 8.0 Hz, 2H), 4.21 (t, J = 9.6 Hz, 2H), 3.99 (t, J = 9.6 Hz, 2H), 2.78 (s, 3H); ^{13}C NMR: (100 MHz, $CDCl_3$) δ 161.0, 156.2, 142.7, 141.4, 139.1, 138.7, 136.0, 130.5, 130.1, 128.5, 126.9, 126.0, 122.9, 122.2, 121.8, 120.7, 120.0, 118.5, 116.8, 108.3 , 53.1, 52.6, 25.3; HRMS (ESI) calculated for $[C_{25}H_{23}N_4]^+$ $[M+H]^+$ requires m/z 379.1917, found m/z 379.1919.

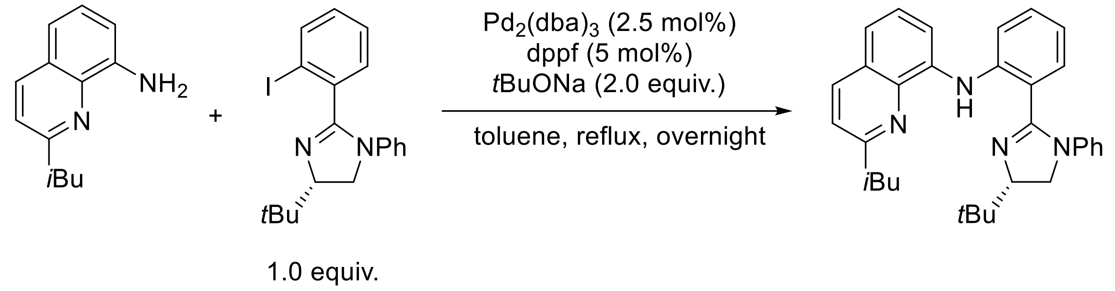

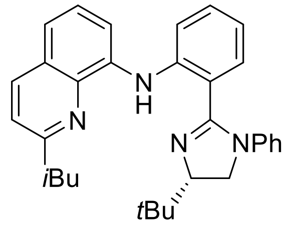

(*S*)-*N*-(2-(4-(*tert*-butyl)-1-phenyl-4,5-dihydro-1*H*-imidazol-2-yl)phenyl)-2-isobutylquinolin-8-amine (L12). To a 50 mL flame-dried Schlenk flask, $Pd_2(dba)_3$ (0.0408 g, 0.05 mmol), dppf (0.0554 g, 0.10 mmol) and toluene (10 mL) were added in sequence under the atmosphere of nitrogen. The solution was stirred for 10 mins. (*S*)-4-(*tert*-butyl)-2-(2-iodophenyl)-1-phenyl-4,5-dihydro-1*H*-imidazole (0.81 g, 2.0 mmol), 2-isobutylquinolin-8-amine (0.40 g, 2.0 mol), sodium *tert*-butoxide (0.38 g, 4.0 mmol) were added in sequence under the atmosphere of nitrogen. The solution was refluxed overnight, thereafter, cooled down to room temperature. The solution was filtered through a pad of silica gel. All volatiles were removed from the solution *via* rotary evaporation to give a brown sticky oil. And the residue was purified by flash column chromatography using PE/Et_3N (50/1) as the eluent to give 0.74 g (1.6 mmol, 78% yield) of the title compound as a pale yellow solid. M.p. 134.3-136.2 ^{o}C. Optical Rotation: $[\alpha]_{20}^{D}$ = +58 (c 0.87, $CHCl_3$). IR (cm^{-1}): 2956, 2926, 2869, 1570, 1521, 1497, 1456. ^{1}H NMR: (400 MHz, $CDCl_3$) δ 9.41 (s, 1H), 7.98 (d, *J* = 8.4 Hz, 1H), 7.68 (d, *J* = 8.4 Hz, 1H), 7.38 (d, *J* = 7.6 Hz, 1H), 7.34-7.29 (m, 1H), 7.28-7.22 (m, 3H), 7.17 (dd, *J* = 8.0, 0.8 Hz, 1H), 7.08-7.00 (m, 2H), 6.90-6.80 (m, 2H), 6.73 (d, *J* = 7.6 Hz, 2H), 4.09 (dd, *J* = 10.8, 8.8 Hz, 1H), 3.98-3.88 (m, 1H), 3.70 (dd, *J* = 8.8, 8.8 Hz, 1H), 2.90 (dd, *J* = 13.6, 7.6 Hz, 1H), 2.81 (dd, *J* = 13.2, 7.6 Hz, 1H), 2.35-2.20 (m, 1H), 1.02 (s, 9H), 0.97 (t, *J* = 6.4 Hz, 6H); ^{13}C NMR: (100 MHz, $CDCl_3$) δ 160.0, 159.6, 142.8, 141.2, 139.6, 138.8, 135.8, 130.6, 129.9, 128.5, 127.2, 125.8, 123.0, 122.4, 122.2, 121.8, 120.8, 119.8, 116.9, 109.0, 53.8, 48.0, 34.1, 29.1, 26.1, 22.65, 22.58; HRMS (ESI) calculated for $[C_{32}H_{37}N_4]^+$ $[M+H]^+$ requires m/z 477.3013, found m/z 477.3011.

III. Sequential Hydrosilylation/Hydrohydrazidation of Alkynes

General procedure (sequential hydrosilylation/hydrohydrazidation of alkynes):

A 25 mL Schlenk flask equipped with a magnetic stirrer and a flanging rubber plug was dried with flame under vacuum. When cooled to ambient temperature (10 - 25 °C), it was vacuumed and flushed with N_2. This degassed procedure was repeated for three times. To the flame-dried Schlenk flask Xantphos•$CoBr_2$ complex (0.0030 mmol, 1 mol%), 1.2 mL (0.25 M) of Et_2O and diphenylsilane (0.30 mmol, 1.0 equiv.) (or other secondary silanes) were added by dropwise sequentially. After that, $NaBHEt_3$ (9 μL, 1.0 M in THF, 0.0090 mmol) and alkyne (0.30 mmol, 1.0 equiv.) were added to the mixture sequentially and stirred for 5 s, and then **L5** (0.0060 mmol, 2 mol%), phenylsilane (0.36 mmol, 1.2 equiv.), phenyldiazoacetate (0.30 mmol, 1.0 equiv.) (or other aryldiazoacetates), and H_2O (0.90 mmol, 3.0 equiv.) were added by dropwise sequentially. Pinholes are sealed with silicone grease and the flanging rubber plug was wrapped with sealing film tightly. The mixture was stirred at ambient temperature for 12 h, and quenched by 5 mL of petroleum ether (PE) and stirred for 5 mins until catalyst precipitated. The resulting solution was filtered through a pad of silica gel and washed using PE/EtOAc (5/1) (15 mL × 3) (or other suitable solvent). The combined filtrates were concentrated *in vacuo*. NMR yield was monitored by ^1H NMR analysis using TMSPh as internal standard. The crude mixture was purified by short flash column chromatography to give the corresponding product.

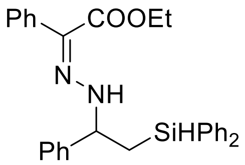

Ethyl (*Z*)-2-(2-(2-(diphenylsilyl)-1-phenylethyl)hydrazono)-2-phenylacetate (4a). Prepared according to the general procedure using 33 μL (0.93 g/mL, 0.30 mmol) of phenylacetylene, 56 μL (0.99 g/mL, 0.30 mmol) of diphenylsilane, 0.0081 g (0.0090 mmol) of Xantphos•CoI_2, 27 μL (1.0 M in THF, 0.0090 mmol) of $NaBHEt_3$, 1.2 mL (0.25 M) of Et_2O, 0.0087 g (0.0180 mmol) of **L5**, 45 μL (0.88 g/mL, 0.36 mmol) of phenylsilane, 48 μL (1.19 g/mL, 0.30 mmol) of ethyl 2-diazo-2-phenylacetate, and 16 μL (1.00 g/mL, 0.90 mmol) of H_2O. After 12 h, the resulting solution was quenched. The combined filtrate was concentrated and the crude mixture was purified by short flash column chromatography using PE to PE/EtOAc = 100/1 as the eluent to

give 0.1235 g (0.26 mmol, 86% yield) of the title compound as a colorless oil. IR (cm^{-1}): 3241, 3065, 2133, 1670, 1514. ^{1}H NMR: (400 MHz, $CDCl_3$) δ 10.82 (d, J = 4.8 Hz, 1H), 7.55-7.50 (m, 2H), 7.49-7.42 (m, 4H), 7.40-7.25 (m, 12H), 7.24-7.20 (m, 2H), 4.85-4.76 (m, 2H), 4.21 (q, J = 7.2 Hz, 2H), 2.20-2.08 (m, 1H), 1.90-1.80 (m, 1H), 1.28 (t, J = 7.2 Hz, 3H); ^{13}C NMR: (100 MHz, $CDCl_3$) δ 163.5, 143.4, 137.1, 135.2, 135.1, 133.7, 133.6, 129.64, 129.58, 128.6, 128.3, 128.00, 127.95, 127.6, 127.4, 126.7, 125.6, 62.2, 60.3, 21.0, 14.2; HRMS (ESI) calculated for $[C_{30}H_{30}N_2O_2SiNa]^+$ $[M+Na]^+$ requires m/z 501.1969, found m/z 501.1968.

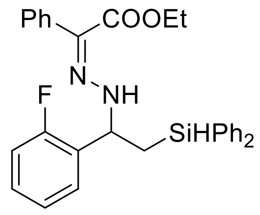

Ethyl (*Z*)-2-(2-(2-(diphenylsilyl)-1-(2-fluorophenyl)ethyl)hydrazono)-2-phenylacetate (4b). Prepared according to the general procedure using 0.0365 g (0.30 mmol) of 1-ethynyl-2-fluorobenzene, 56 μL (0.99 g/mL, 0.30 mmol) of diphenylsilane, 0.0024 g (0.0030 mmol) of Xantphos•$CoBr_2$, 9 μL (1.0 M in THF, 0.0090 mmol) of $NaBHEt_3$, 1.2 mL (0.25 M) of Et_2O, 0.0020 g (0.0063 mmol) of **L5**, 45 μL (0.88 g/mL, 0.36 mmol) of phenylsilane, 48 μL (1.19 g/mL, 0.30 mmol) of ethyl 2-diazo-2-phenylacetate, and 16 μL (1.00 g/mL, 0.90 mmol) of H_2O. After 12 h, the resulting solution was quenched. The combined filtrate was concentrated and the crude mixture was purified by short flash column chromatography using PE to PE/EtOAc = 200/1 as the eluent to give 0.0775 g (0.16 mmol, 52% yield) of the title compound as a light yellow oil. IR (cm^{-1}): 3251, 3066, 2929, 2132, 1735, 1672, 1515. ^{1}H NMR: (400 MHz, $CDCl_3$) δ 10.84 (d, J = 5.6 Hz, 1H), 7.55-7.50 (m, 2H), 7.50-7.46 (m, 2H), 7.46-7.42 (m, 2H), 7.37-7.20 (m, 10H), 7.17-7.10 (m, 1H), 7.05-6.98 (m, 1H), 6.98-6.91 (m, 1H), 5.15-5.06 (m, 1H), 4.84 (t, J = 3.6 Hz, 1H), 4.22 (q, J = 7.2 Hz, 2H), 2.15-2.05 (m, 1H), 1.97-1.88 (m, 1H), 1.28 (t, J = 7.2 Hz, 3H); ^{13}C NMR: (100 MHz, $CDCl_3$) δ 163.4, 161.1 (d, J = 244.5 Hz, 1C), 137.0, 135.1, 135.0, 133.4, 133.3, 130.7 (d, J = 13.2 Hz, 1C), 129.62, 129.58, 128.8 (d, J = 8.3 Hz, 1C), 128.3, 128.2, 128.1 (d, J = 4.5 Hz, 1C), 127.9, 127.6, 126.8, 126.0, 124.2 (d, J = 3.3 Hz, 1C), 115.6 (d, J = 21.8 Hz, 1C), 60.3, 57.4, 20.1, 14.2; ^{19}F NMR: (376 MHz, $CDCl_3$) δ -118.0; HRMS (ESI) calculated for $[C_{30}H_{30}FN_2O_2Si]^+$ $[M+H]^+$ requires m/z 497.2055, found m/z 497.2055.

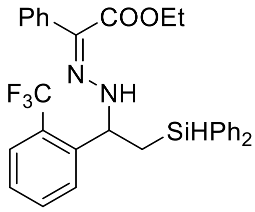

Ethyl (*Z*)-2-(2-(2-(diphenylsilyl)-1-(2-(trifluoromethyl)phenyl)ethyl)hydrazono)-2-phenylacetate (4c). Prepared according to the general procedure using 0.0510 g (0.30 mmol) of 1-ethynyl-2-(trifluoromethyl)benzene, 56 μL (0.99 g/mL, 0.30 mmol) of diphenylsilane, 0.0024 g (0.0030 mmol) of Xantphos•$CoBr_2$, 9 μL (1.0 M in THF, 0.0090 mmol) of $NaBHEt_3$, 1.2 mL (0.25 M) of Et_2O, 0.0019 g (0.0060 mmol) of **L5**, 45 μL (0.88 g/mL, 0.36 mmol) of phenylsilane, 48 μL (1.19 g/mL, 0.30 mmol) of ethyl 2-diazo-2-phenylacetate, and 16 μL (1.00 g/mL, 0.90 mmol) of H_2O. After 12 h, the resulting solution was quenched. The combined filtrate was concentrated and the crude mixture was purified by short flash column chromatography using PE to PE/EtOAc = 100/1 as the eluent to give 0.0738 g (0.14 mmol, 45% yield) of the title compound as a light yellow oil. IR (cm^{-1}): 3262, 3069, 2872, 2140, 1737, 1691, 1516. ^{1}H NMR: (400 MHz, $CDCl_3$) δ 10.77 (brs, 1H), 7.63 (d, J = 8.0 Hz, 1H), 7.60-7.48 (m, 5H), 7.48-7.42 (m, 2H), 7.38-7.21 (m, 11H), 7.8 (dd, J = 4.8, 10.4 Hz, 1H), 4.95 (t, J = 3.2 Hz, 1H), 4.22 (q, J = 7.2 Hz, 2H), 2.20-2.18 (m, 1H), 1.83 (dt, J = 4.4, 14.8 Hz, 1H), 1.30 (t, J = 7.2 Hz, 3H); ^{13}C NMR: (100 MHz, $CDCl_3$) δ 163.3, 143.5, 137.0, 135.12, 135.10, 133.6, 133.2, 132.3, 129.7, 129.6, 128.3, 128.0, 127.96, 126.95, 127.7, 127.6, 127.13, 127.06, 126.8, 126.4, 125.6 (q, J = 5.9 Hz, 1C), 60.4, 56.8, 21.4, 14.2; ^{19}F NMR: (376 MHz, $CDCl_3$) δ -57.9; HRMS (ESI) calculated for $[C_{30}H_{29}F_3N_2O_2SiNa]^+$ $[M+Na]^+$ requires m/z 569.1843, found m/z 569.1842.

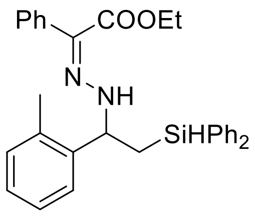

Ethyl (*Z*)-2-(2-(2-(diphenylsilyl)-1-(*o*-tolyl)ethyl)hydrazono)-2-phenylacetate (4d). Prepared according to the general procedure using 0.0348 g (0.30 mmol) of 1-ethynyl-2-methylbenzene, 56

μL (0.99 g/mL, 0.30 mmol) of diphenylsilane, 0.0024 g (0.0030 mmol) of Xantphos•$CoBr_2$, 9 μL (1.0 M in THF, 0.0090 mmol) of $NaBHEt_3$, 1.2 mL (0.25 M) of Et_2O, 0.0020 g (0.0063 mmol) of **L5**, 45 μL (0.88 g/mL, 0.36 mmol) of phenylsilane, 48 μL (1.19 g/mL, 0.30 mmol) of ethyl 2-diazo-2-phenylacetate, and 16 μL (1.00 g/mL, 0.90 mmol) of H_2O. After 12 h, the resulting solution was quenched. The combined filtrate was concentrated and the crude mixture was purified by short flash column chromatography using PE to PE/EtOAc = 100/1 as the eluent to give 0.1182 g (0.24 mmol, 80% yield) of the title compound as a light yellow oil. IR (cm^{-1}): 3669, 2981, 2903, 2135, 1736, 1688, 1596. ^1H NMR: (400 MHz, $CDCl_3$) δ 10.80 (brs, 1H), 7.52-7.44 (m, 6H), 7.40-7.25 (m, 9H), 7.25-7.19 (m, 1H), 7.19-7.05 (m, 3H), 5.07 (t, J = 7.6 Hz, 1H), 4.82 (t, J = 3.6 Hz, 1H), 4.21 (q, J = 7.2 Hz, 2H), 2.16 (s, 3H), 2.15-2.07 (m, 1H), 1.90-1.80 (m, 1H), 1.28 (t, J = 7.2 Hz, 3H); ^{13}C NMR: (100 MHz, $CDCl_3$) δ 163.5, 141.2, 137.1, 135.2, 135.1, 135.0, 133.7, 133.6, 130.6, 129.62, 129.58, 128.3, 128.0, 127.9, 127.6, 127.2, 126.7, 126.4, 126.0, 125.2, 60.2, 58.2, 20.4, 19.3, 14.2; HRMS (ESI) calculated for $[C_{31}H_{32}N_2O_2SiNa]^+$ $[M+Na]^+$ requires m/z 515.2125, found m/z 515.2132.

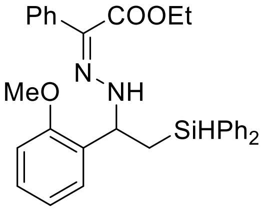

Ethyl (*Z*)-2-(2-(2-(diphenylsilyl)-1-(2-methoxyphenyl)ethyl)hydrazono)-2-phenylacetate (4e). Prepared according to the general procedure using 0.0397 g (0.30 mmol) of 1-ethynyl-2-methoxybenzene, 56 μL (0.99 g/mL, 0.30 mmol) of diphenylsilane, 0.0024 g (0.0030 mmol) of Xantphos•$CoBr_2$, 9 μL (1.0 M in THF, 0.0090 mmol) of $NaBHEt_3$, 1.2 mL (0.25 M) of Et_2O, 0.0020 g (0.0063 mmol) of **L5**, 45 μL (0.88 g/mL, 0.36 mmol) of phenylsilane, 48 μL (1.19 g/mL, 0.30 mmol) of ethyl 2-diazo-2-phenylacetate, and 16 μL (1.00 g/mL, 0.90 mmol) of H_2O. After 12 h, the resulting solution was quenched. The combined filtrate was concentrated and the crude mixture was purified by short flash column chromatography using PE to PE/EtOAc = 100/1 as the eluent to give 0.1022 g (0.20 mmol, 67% yield) of the title compound as a light yellow oil. IR (cm^{-1}): 3262, 3000, 2131, 1735, 1596, 1491. ^1H NMR: (400 MHz, $CDCl_3$) δ 11.05 (brs, 1H), 7.55-7.49 (m, 2H), 7.48 (m, 4H), 7.38-7.23 (m, 8H), 7.23-7.13 (m, 3H), 6.89-6.82 (m, 1H), 6.77 (d,

J = 8.0 Hz, 1H), 5.17-5.08 (m, 1H), 4.78 (t, J = 7.6 Hz, 1H), 4.22 (q, J = 7.2 Hz, 2H), 3.76 (s, 3H), 2.15-2.04 (m, 1H), 2.02-1.92 (m, 1H), 1.28 (t, J = 7.2 Hz, 3H); ^{13}C NMR: (100 MHz, $CDCl_3$) δ 163.4, 156.5, 137.4, 135.1, 135.0, 134.1, 134.0, 131.5, 129.4, 129.3, 128.33, 128.30, 127.9, 127.8, 127.63, 127.55, 126.5, 125.0, 120.6, 110.7, 60.0, 59.6, 55.1, 20.0, 14.2; HRMS (ESI) calculated for $[C_{31}H_{32}N_2O_3SiNa]^+$ $[M+Na]^+$ requires m/z 531.2074, found m/z 531.2071.

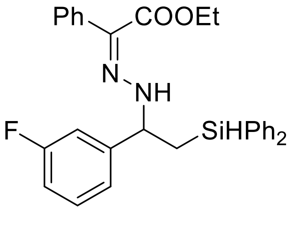

Ethyl (*Z*)-2-(2-(2-(diphenylsilyl)-1-(3-fluorophenyl)ethyl)hydrazono)-2-phenylacetate (4f). Prepared according to the general procedure using 0.0360 g (0.30 mmol) of 1-ethynyl-3-fluorobenzene, 56 μL (0.99 g/mL, 0.30 mmol) of diphenylsilane, 0.0024 g (0.0030 mmol) of Xantphos•$CoBr_2$, 9 μL (1.0 M in THF, 0.0090 mmol) of $NaBHEt_3$, 1.2 mL (0.25 M) of Et_2O, 0.0020 g (0.0063 mmol) of **L5**, 45 μL (0.88 g/mL, 0.36 mmol) of phenylsilane, 48 μL (1.19 g/mL, 0.30 mmol) of ethyl 2-diazo-2-phenylacetate, and 16 μL (1.00 g/mL, 0.90 mmol) of H_2O. After 12 h, the resulting solution was quenched. The combined filtrate was concentrated and the crude mixture was purified by short flash column chromatography using PE to PE/EtOAc = 100/1 as the eluent to give 0.1296 g (0.26 mmol, 87% yield) of the title compound as a colorless oil. IR (cm^{-1}): 3252, 3066, 2928, 2134, 1735, 1672, 1515. ^{1}H NMR: (400 MHz, $CDCl_3$) δ 10.77 (brs, 1H), 7.56-7.50 (m, 2H), 7.50-7.42 (m, 4H), 7.39-7.22 (m, 10H), 7.02 (d, J = 7.6 Hz, 1H), 7.00-6.93 (m, 1H), 6.93-6.85 (m, 1H), 4.84-4.75 (m, 2H), 4.23 (q, J = 7.2 Hz, 2H), 2.15-2.05 (m , 1H), 1.87-1.78 (m, 1H), 1.29 (t, J = 7.2 Hz, 3H); ^{13}C NMR: (100 MHz, $CDCl_3$) δ 163.4, 162.9 (d, J = 244.5 Hz, 1C), 146.2 (d, J = 6.2 Hz, 1C), 136.9, 135.2, 135.0, 133.34, 133.30, 130.0 (d, J = 8.2 Hz, 1C), 129.7 (d, J = 5.8 Hz, 1C), 128.3, 128.05, 128.00, 127.7, 126.9, 126.3, 122.4, 122.3, 114.2 (d, J = 21.0 Hz, 1C), 113.6 (d, J = 21.4 Hz, 1C), 61.6, 60.4, 20.8, 14.2; ^{19}F NMR: (376 MHz, $CDCl_3$) δ -112.6; HRMS (ESI) calculated for $[C_{30}H_{30}FN_2O_2Si]^+$ $[M+H]^+$ requires m/z 497.2055, found m/z 497.2055.

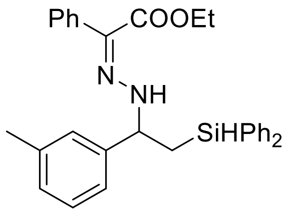

Ethyl (*Z*)-2-(2-(2-(diphenylsilyl)-1-(*m*-tolyl)ethyl)hydrazono)-2-phenylacetate (4g). Prepared according to the general procedure using 0.0349 g (0.30 mmol) of 1-ethynyl-3-methylbenzene, 56 μL (0.99 g/mL, 0.30 mmol) of diphenylsilane, 0.0024 g (0.0030 mmol) of Xantphos•$CoBr_2$, 9 μL (1.0 M in THF, 0.0090 mmol) of $NaBHEt_3$, 1.2 mL (0.25 M) of Et_2O, 0.0020 g (0.0063 mmol) of **L5**, 45 μL (0.88 g/mL, 0.36 mmol) of phenylsilane, 48 μL (1.19 g/mL, 0.30 mmol) of ethyl 2-diazo-2-phenylacetate, and 16 μL (1.00 g/mL, 0.90 mmol) of H_2O. After 12 h, the resulting solution was quenched. The combined filtrate was concentrated and the crude mixture was purified by short flash column chromatography using PE to PE/EtOAc = 200/1 as the eluent to give 0.1206 g (0.25 mmol, 82% yield) of the title compound as a light yellow oil. IR (cm^{-1}): 3240, 2978, 2907, 2130, 1737, 1670, 1513. ^1H NMR: (400 MHz, $CDCl_3$) δ 10.82 (brs, 1H), 7.55-7.50 (m, 2H), 7.49-7.43 (m, 4H), 7.39-7.25 (m, 8H), 7.25-7.13 (m, 2H), 7.10-7.00 (m, 3H), 4.82-4.74 (m, 2H), 4.21 (q, *J* = 7.2 Hz, 2H), 2.28 (s, 3H), 2.18-2.09 (m, 1H), 1.89-1.79 (m, 1H), 1.28 (t, *J* = 7.2 Hz, 3H); ^{13}C NMR: (100 MHz, $CDCl_3$) δ 163.5, 143.1, 138.1, 137.2, 135.2, 135.0, 133.72, 133.66, 129.6, 129.5, 128.5, 128.3, 128.2, 128.0, 127.9, 127.6, 127.5, 126.7, 125.4, 123.7, 62.3, 60.2, 21.4, 21.0, 14.2; HRMS (ESI) calculated for $[C_{31}H_{32}N_2O_2SiNa]^+$ $[M+Na]^+$ requires m/z 515.2125, found m/z 515.2124.

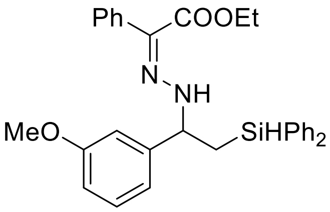

Ethyl (*Z*)-2-(2-(2-(diphenylsilyl)-1-(3-methoxyphenyl)ethyl)hydrazono)-2-phenylacetate (4h). Prepared according to the general procedure using 0.0397 g (0.30 mmol) of 1-ethynyl-3-methoxybenzene, 56 μL (0.99 g/mL, 0.30 mmol) of diphenylsilane, 0.0026 g (0.0033 mmol) of Xantphos•$CoBr_2$, 9 μL (1.0 M in THF, 0.0090 mmol) of $NaBHEt_3$, 1.2 mL (0.25 M) of Et_2O, 0.0020 g (0.0063 mmol) of **L5**, 45 μL (0.88 g/mL, 0.36 mmol) of phenylsilane, 48 μL (1.19 g/mL, 0.30 mmol) of ethyl 2-diazo-2-phenylacetate, and 16 μL (1.00 g/mL, 0.90 mmol) of H_2O.

After 12 h, the resulting solution was quenched. The combined filtrate was concentrated and the crude mixture was purified by short flash column chromatography using PE to PE/EtOAc = 100/1 as the eluent to give 0.1328 g (0.26 mmol, 87% yield) of the title compound as a light yellow oil. IR (cm^{-1}): 3243, 2981, 2906, 2133, 1669, 1601, 1514. ^1H NMR: (400 MHz, $CDCl_3$) δ 10.81 (d, J = 5.2 Hz, 1H), 7.55-7.50 (m, 2H), 7.49-7.44 (m, 4H), 7.33-7.25 (m, 8H), 7.25-7.15 (m, 2H), 6.90-6.84 (m, 1H), 6.80-6.73 (m, 2H), 4.82-4.75 (m, 2H), 4.21 (q, J = 7.2 Hz, 2H), 3.72 (s, 3H), 2.17-2.08 (m, 1H), 1.90-1.80 (m, 1H), 1.28 (t, J = 7.2 Hz, 3H); ^{13}C NMR: (100 MHz, $CDCl_3$) δ 163.4, 159.7, 145.0, 137.1, 135.2, 135.0, 133.63, 133.57, 129.6, 129.5, 128.3, 128.0, 127.9, 127.6, 126.7, 125.7, 119.0, 112.8, 112.4, 62.2, 60.2, 55.1, 20.9, 14.2; HRMS (ESI) calculated for $[C_{31}H_{32}N_2O_3SiNa]^+$ $[M+Na]^+$ requires m/z 531.2074, found m/z 531.2078.

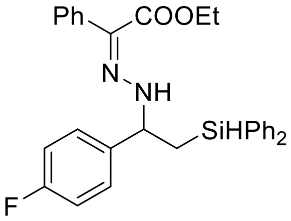

Ethyl (*Z*)-2-(2-(2-(diphenylsilyl)-1-(4-fluorophenyl)ethyl)hydrazono)-2-phenylacetate (4i). Prepared according to the general procedure using 0.0367 g (0.31 mmol) of 1-ethynyl-4-fluorobenzene, 56 μL (0.99 g/mL, 0.30 mmol) of diphenylsilane, 0.0024 g (0.0030 mmol) of Xantphos•$CoBr_2$, 9 μL (1.0 M in THF, 0.0090 mmol) of $NaBHEt_3$, 1.2 mL (0.25 M) of Et_2O, 0.0020 g (0.0063 mmol) of **L5**, 45 μL (0.88 g/mL, 0.36 mmol) of phenylsilane, 48 μL (1.19 g/mL, 0.30 mmol) of ethyl 2-diazo-2-phenylacetate, and 16 μL (1.00 g/mL, 0.90 mmol) of H_2O. After 12 h, the resulting solution was quenched. The combined filtrate was concentrated and the crude mixture was purified by short flash column chromatography using PE to PE/EtOAc = 100/1 as the eluent to give 0.1071 g (0.22 mmol, 72% yield) of the title compound as a colorless oil. IR (cm^{-1}): 3249, 3067, 2927, 2132, 1735, 1670, 1509. ^1H NMR: (400 MHz, $CDCl_3$) δ 10.76 (brs, 1H), 7.54-7.49 (m, 2H), 7.48-7.42 (m, 4H), 7.37-7.25 (m, 8H), 7.24-7.16 (m, 3H), 6.96-6.89 (m, 2H), 4.82-4.75 (m, 2H), 4.21 (q, J = 7.2 Hz, 2H), 2.16-2.08 (m, 1H), 1.87-1.78 (m, 1H), 1.27 (t, J = 7.2 Hz, 3H); ^{13}C NMR: (100 MHz, $CDCl_3$) δ 163.4, 162.0 (d, J = 244.0 Hz, 1C), 139.0 (d, J = 2.9 Hz, 1C), 137.0, 135.1, 135.0, 133.4, 133.3, 129.7, 129.6, 128.4 (d, J = 8.4 Hz, 1C), 128.3, 128.01, 127.95, 127.6, 126.8, 126.0, 115.3 (d, J = 21.1 Hz, 1C), 61.4, 60.3, 20.9, 14.1; ^{19}F NMR: (376

MHz, $CDCl_3$) δ -115.0; HRMS (ESI) calculated for $[C_{30}H_{30}FN_2O_2Si]^+$ $[M+H]^+$ requires m/z 497.2055, found m/z 497.2055.

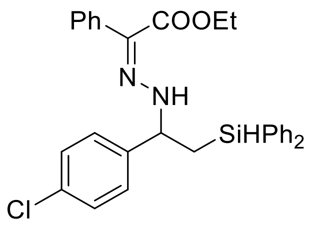

Ethyl (*Z*)-2-(2-(1-(4-chlorophenyl)-2-(diphenylsilyl)ethyl)hydrazono)-2-phenylacetate (4j). Prepared according to the general procedure using 0.0412 g (0.30 mmol) of 1-chloro-4-ethynylbenzene, 56 μL (0.99 g/mL, 0.30 mmol) of diphenylsilane, 0.0024 g (0.0030 mmol) of Xantphos•$CoBr_2$, 9 μL (1.0 M in THF, 0.0090 mmol) of $NaBHEt_3$, 1.2 mL (0.25 M) of Et_2O, 0.0020 g (0.0063 mmol) of **L5**, 45 μL (0.88 g/mL, 0.36 mmol) of phenylsilane, 48 μL (1.19 g/mL, 0.30 mmol) of ethyl 2-diazo-2-phenylacetate, and 16 μL (1.00 g/mL, 0.90 mmol) of H_2O. After 12 h, the resulting solution was quenched. The combined filtrate was concentrated and the crude mixture was purified by short flash column chromatography using PE to PE/EtOAc = 100/1 as the eluent to give 0.0662 g (0.13 mmol, 43% yield) of the title compound as a light yellow oil. IR (cm^{-1}): 3240, 3065, 2926, 2133, 1738, 1593, 1513. ^1H NMR: (400 MHz, $CDCl_3$) δ 10.75 (d, J = 4.8 Hz, 1H), 7.55-7.49 (m, 2H), 7.48-7.41 (m, 4H), 7.40-7.33 (m, 3H), 7.33-7.26 (m, 5H), 7.26-7.20 (m, 3H), 7.19-7.14 (m, 2H), 4.82-4.73 (m, 2H), 4.22 (q, J = 7.2 Hz, 2H), 2.15-2.06 (m, 1H), 1.86-1.77 (m, 1H), 1.32-1.27 (m, J = 7.2 Hz, 3H); ^{13}C NMR: (100 MHz, $CDCl_3$) δ 163.4, 141.9, 136.9, 135.1, 135.0, 133.32, 133.27, 133.1, 129.74, 129.65, 128.6, 128.3, 128.2, 128.1, 128.0, 127.7, 126.9, 126.2, 61.4, 60.4, 20.8, 14.2; HRMS (ESI) calculated for $[C_{30}H_{29}ClN_2O_2SiNa]^+$ $[M+Na]^+$ requires m/z 535.1579, found m/z 535.1577.

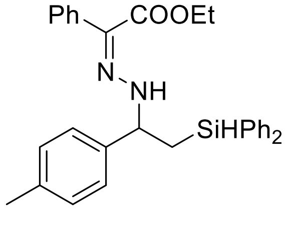

Ethyl (*Z*)-2-(2-(2-(diphenylsilyl)-1-(*p*-tolyl)ethyl)hydrazono)-2-phenylacetate (4k). Prepared according to the general procedure using 0.0351 g (0.30 mmol) of 1-ethynyl-4-methylbenzene, 56

μL (0.99 g/mL, 0.30 mmol) of diphenylsilane, 0.0024 g (0.0030 mmol) of Xantphos•$CoBr_2$, 9 μL (1.0 M in THF, 0.0090 mmol) of $NaBHEt_3$, 1.2 mL (0.25 M) of Et_2O, 0.0020 g (0.0063 mmol) of **L5**, 45 μL (0.88 g/mL, 0.36 mmol) of phenylsilane, 48 μL (1.19 g/mL, 0.30 mmol) of ethyl 2-diazo-2-phenylacetate, and 16 μL (1.00 g/mL, 0.90 mmol) of H_2O. After 12 h, the resulting solution was quenched. The combined filtrate was concentrated and the crude mixture was purified by short flash column chromatography using PE to PE/EtOAc = 100/1 as the eluent to give 0.1153 g (0.23 mmol, 78% yield) of the title compound as a colorless oil. IR (cm^{-1}): 3250, 3052, 2970, 2133, 1734, 1670, 1513. ^1H NMR: (400 MHz, $CDCl_3$) δ 10.81 (brs, 1H), 7.55-7.50 (m, 2H), 7.49-7.44 (m, 4H), 7.40-7.25 (m, 8H), 7.25-7.20 (m, 1H), 7.18-7.05 (m, 4H), 4.82-4.74 (m, 2H), 4.20 (q, J = 6.8 Hz, 2H), 2.30 (s, 3H), 2.18-2.19 (m, 1H), 1.89-1.79 (m, 1H), 1.27 (t, J = 6.8 Hz, 3H); ^{13}C NMR: (100 MHz, $CDCl_3$) δ 163.4, 140.3, 137.2, 137.0, 135.2, 135.0, 133.7, 133.6, 129.6, 129.5, 129.2, 128.3, 128.0, 127.9, 127.6, 126.7, 126.6, 125.4, 61.9, 60.2, 21.1, 21.0, 14.2; HRMS (ESI) calculated for $[C_{31}H_{32}N_2O_2SiNa]^+$ $[M+Na]^+$ requires m/z 515.2125, found m/z 515.2129.

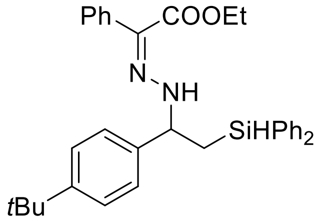

Ethyl (*Z*)-2-(2-(1-(4-(*tert*-butyl)phenyl)-2-(diphenylsilyl)ethyl)hydrazono)-2-phenylacetate (4l). Prepared according to the general procedure using 0.0478 g (0.30 mmol) of 1-(*tert*-butyl)-4-ethynylbenzene, 56 μL (0.99 g/mL, 0.30 mmol) of diphenylsilane, 0.0024 g (0.0030 mmol) of Xantphos•$CoBr_2$, 9 μL (1.0 M in THF, 0.0090 mmol) of $NaBHEt_3$, 1.2 mL (0.25 M) of Et_2O, 0.0020 g (0.0063 mmol) of **L5**, 45 μL (0.88 g/mL, 0.36 mmol) of phenylsilane, 48 μL (1.19 g/mL, 0.30 mmol) of ethyl 2-diazo-2-phenylacetate, and 16 μL (1.00 g/mL, 0.90 mmol) of H_2O. After 12 h, the resulting solution was quenched. The combined filtrate was concentrated and the crude mixture was purified by short flash column chromatography using PE to PE/EtOAc = 100/1 as the eluent to give 0.1181 g (0.22 mmol, 74% yield) of the title compound as a light yellow oil. IR (cm^{-1}): 3243, 2966, 2904, 2134, 1670, 1513. ^1H NMR: (400 MHz, $CDCl_3$) δ 10.83 (brs, 1H), 7.53-7.42 (m, 6H), 7.36-7.26 (m, 9H), 7.26-7.23 (m, 1H), 7.23-7.17 (m, 3H), 4.86-4.78

(m, 2H), 4.21 (q, J = 7.2 Hz, 2H), 2.20-2.10 (m, 1H), 1.91-1.81 (m, 1H), 1.31-1.25 (m, 12H); ^{13}C NMR: (100 MHz, $CDCl_3$) δ 163.5, 150.2, 140.1, 137.2, 135.2, 135.1, 133.8, 133.7, 129.54, 129.49, 128.3, 127.94, 127.88, 127.6, 126.7, 126.4, 125.4, 125.2, 62.0, 60.2, 34.4, 31.3, 21.0, 14.2; HRMS (ESI) calculated for $[C_{34}H_{39}N_2O_2Si]^+$ $[M+H]^+$ requires m/z 535.2775, found m/z 535.2772.

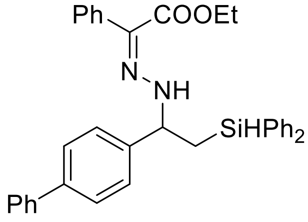

Ethyl (*Z*)-2-(2-(2-(diphenylsilyl)-1-(4-methoxyphenyl)ethyl)hydrazono)-2-phenylacetate (4m). Prepared according to the general procedure using 0.0535 g (0.30 mmol) of 4-ethynyl-1,1'-biphenyl, 56 μL (0.99 g/mL, 0.30 mmol) of diphenylsilane, 0.0024 g (0.0030 mmol) of Xantphos•$CoBr_2$, 9 μL (1.0 M in THF, 0.0090 mmol) of $NaBHEt_3$, 1.2 mL (0.25 M) of Et_2O, 0.0019 g (0.0060 mmol) of **L5**, 45 μL (0.88 g/mL, 0.36 mmol) of phenylsilane, 48 μL (1.19 g/mL, 0.30 mmol) of ethyl 2-diazo-2-phenylacetate, and 16 μL (1.00 g/mL, 0.90 mmol) of H_2O. After 12 h, the resulting solution was quenched. The combined filtrate was concentrated and the crude mixture was purified by short flash column chromatography using PE to PE/EtOAc = 100/1 as the eluent to give 0.1298 g (0.23 mmol, 78% yield) of the title compound as a colorless oil. IR (cm^{-1}): 3240, 2980, 2906, 2132, 1735, 1685, 1513. ^{1}H NMR: (400 MHz, $CDCl_3$) δ 10.86 (brs, 1H), 7.57-7.50 (m, 4H), 7.51-7.45 (m, 6H), 7.44-7.38 (m, 2H), 7.37-7.20 (m, 12H), 4.91-4.82 (m, 2H), 4.22 (q, J = 7.2 Hz, 2H), 2.23-2.13 (m, 1H), 1.95-1.85 (m, 1H), 1.28 (t, J = 7.2 Hz, 3H); ^{13}C NMR: (100 MHz, $CDCl_3$) δ 163.5, 142.3, 140.8, 140.3, 137.1, 135.2, 135.1, 133.5, 129.6, 129.5, 128.7, 128.3, 128.0, 127.9, 127.6, 127.3, 127.2, 127.0, 126.8, 125.7, 61.9, 60.3, 21.0, 14.2; HRMS (ESI) calculated for $[C_{36}H_{35}N_2O_2Si]^+$ $[M+H]^+$ requires m/z 555.2462, found m/z 555.2459.

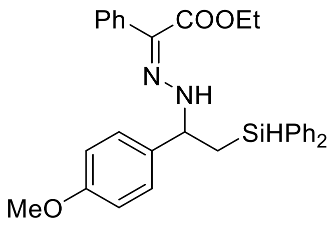

Ethyl (*Z*)-2-(2-(2-(diphenylsilyl)-1-(4-methoxyphenyl)ethyl)hydrazono)-2-phenylacetate (4n).

Prepared according to the general procedure A using 0.0399 g (0.30 mmol) of 1-ethynyl-4-methoxybenzene, 56 μL (0.99 g/mL, 0.30 mmol) of diphenylsilane, 0.0024 g (0.0030 mmol) of Xantphos•$CoBr_2$, 9 μL (1.0 M in THF, 0.0090 mmol) of $NaBHEt_3$, 1.2 mL (0.25 M) of Et_2O, 0.0019 g (0.0060 mmol) of **L5**, 45 μL (0.88 g/mL, 0.36 mmol) of phenylsilane, 48 μL (1.19 g/mL, 0.30 mmol) of ethyl 2-diazo-2-phenylacetate, and 16 μL (1.00 g/mL, 0.90 mmol) of H_2O. After 12 h, the resulting solution was quenched. The combined filtrate was concentrated and the crude mixture was purified by short flash column chromatography using PE to PE/EtOAc = 20/1 as the eluent to give 0.1206 g (0.24 mmol, 79% yield) of the title compound as a colorless oil. IR (cm^{-1}): 3430, 2973, 2905, 2133, 1736, 1687, 1511. ^1H NMR: (400 MHz, $CDCl_3$) δ 10.77 (d, J = 4.8 Hz, 1H), 7.55-7.50 (m, 2H), 7.49-7.43 (m, 4H), 7.40-7.27 (m, 8H), 7.25-7.22 (m, 1H), 7.18 (d, J = 8.8 Hz, 2H), 6.80 (d, J = 8.8 Hz, 2H), 4.82-4.72 (m, 2H), 4.21 (q, J = 7.2 Hz, 2H), 3.77 (s, 3H), 2.20-2.09 (m, 1H), 1.90-1.79 (m, 1H), 1.28 (t, J = 7.2 Hz, 3H); ^{13}C NMR: (100 MHz, $CDCl_3$) δ 163.5, 158.9, 137.2, 135.2, 135.0, 133.8, 133.6, 129.6, 129.5, 128.3, 128.0, 127.9, 127.6, 126.7, 125.4, 113.9, 61.6, 60.2, 55.2, 20.9, 14.2; HRMS (ESI) calculated for $[C_{31}H_{32}N_2O_3SiNa]^+$ $[M+Na]^+$ requires m/z 531.2074, found m/z 531.2070

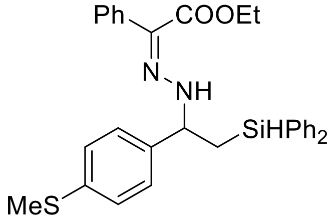

Ethyl (*Z*)-2-(2-(2-(diphenylsilyl)-1-(4-(methylthio)phenyl)ethyl)hydrazono)-2-phenylacetate (4o). Prepared according to the general procedure using 0.0443 g (0.30 mmol) of (4-ethynylphenyl)(methyl)sulfane, 56 μL (0.99 g/mL, 0.30 mmol) of diphenylsilane, 0.0024 g (0.0030 mmol) of Xantphos•$CoBr_2$, 9 μL (1.0 M in THF, 0.0090 mmol) of $NaBHEt_3$, 1.2 mL (0.25 M) of Et_2O, 0.0020 g (0.0063 mmol) of **L5**, 45 μL (0.88 g/mL, 0.36 mmol) of phenylsilane, 48 μL (1.19 g/mL, 0.30 mmol) of ethyl 2-diazo-2-phenylacetate, and 16 μL (1.00 g/mL, 0.90 mmol) of H_2O. After 24 h, the resulting solution was quenched. The combined filtrate was concentrated and the crude mixture was purified by short flash column chromatography using PE to PE/EtOAc = 100/1 as the eluent to give 0.0850 g (0.16 mmol, 54% yield) of the title compound as a light yellow oil. IR (cm^{-1}): 3245, 3058, 2922, 2133, 1732, 1672, 1508. ^1H NMR: (400 MHz, $CDCl_3$) δ

10.78 (brs, 1H), 7.55-7.48 (m, 2H), 7.48-7.42 (m, 4H), 7.40-7.20 (m, 9H), 7.20-7.13 (m, 4H), 4.81-4.73 (m, 2H), 4.21 (q, J = 7.2 Hz, 2H), 2.44 (s, 3H), 2.17-2.07 (m, 1H), 1.88-1.78 (m, 1H), 1.28 (m, J = 7.2 Hz, 3H); ^{13}C NMR: (100 MHz, $CDCl_3$) δ 163.4, 140.2, 137.4, 137.1, 135.2, 135.0, 133.53, 133.45, 129.64, 129.56, 128.3, 128.0, 127.9, 127.6, 127.3, 126.7, 125.8, 61.7, 60.3, 20.8, 15.9, 14.2; HRMS (ESI) calculated for $[C_{31}H_{32}N_2O_2SSiNa]^+$ $[M+Na]^+$ requires m/z 547.1846, found m/z 547.1843.

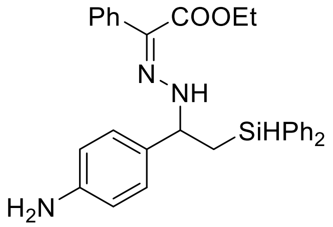

Ethyl (*Z*)-2-(2-(1-(4-aminophenyl)-2-(diphenylsilyl)ethyl)hydrazono)-2-phenylacetate (4p). Prepared according to the general procedure using 0.0352 g (0.30 mmol) of 4-ethynylaniline, 56 μL (0.99 g/mL, 0.30 mmol) of diphenylsilane, 0.0024 g (0.0030 mmol) of Xantphos•$CoBr_2$, 9 μL (1.0 M in THF, 0.0090 mmol) of $NaBHEt_3$, 1.2 mL (0.25 M) of Et_2O, 0.0020 g (0.0063 mmol) of **L5**, 45 μL (0.88 g/mL, 0.36 mmol) of phenylsilane, 48 μL (1.19 g/mL, 0.30 mmol) of ethyl 2-diazo-2-phenylacetate, and 16 μL (1.00 g/mL, 0.90 mmol) of H_2O. After 12 h, the resulting solution was quenched. The combined filtrate was concentrated and the crude mixture was purified by short flash column chromatography using PE to PE/EtOAc = 5/1 as the eluent to give 0.0795 g (0.16 mmol, 54% yield) of the title compound as a light yellow oil. IR (cm^{-1}): 3242, 2980, 2904, 2133, 1666, 1621, 1513. ^{1}H NMR: (400 MHz, $CDCl_3$) δ 10.74 (d, J = 4.8 Hz, 1H), 7.56-7.50 (m, 2H), 7.50-7.43 (m, 4H), 7.40-7.25 (m, 8H), 7.25-7.20 (m, 1H), 7.05 (d, J = 8.4 Hz, 2H), 6.58 (d, J = 8.0 Hz, 2H), 4.76 (t, J = 4.0 Hz, 1H), 4.74-4.67 (m, 1H), 4.20 (q, J = 7.2 Hz, 2H), 3.60 (brs, 2H), 2.19-2.08 (m, 1H), 1.88-1.77 (m, 1H), 1.28 (t, J = 7.2 Hz, 3H); ^{13}C NMR: (100 MHz, $CDCl_3$) δ 163.4, 145.7, 137.3, 135.2, 135.0, 133.9, 133.7, 133.0, 129.5, 129.4, 128.3, 127.93, 127.88, 127.6, 126.6, 125.0, 115.1, 61.7, 60.1, 20.8, 14.2; HRMS (ESI) calculated for $[C_{30}H_{31}N_3O_2SiNa]^+$ $[M+Na]^+$ requires m/z 516.2078, found m/z 516.2078.

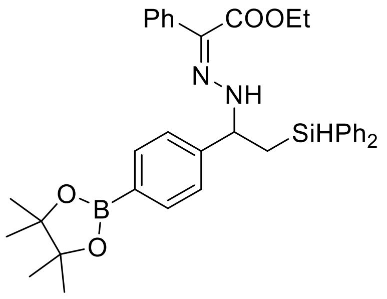

Ethyl (*Z*)-2-(2-(2-(diphenylsilyl)-1-(4-(4,4,5,5-tetramethyl-1,3,2-dioxaborolan-2-yl)phenyl) ethyl)hydrazono)-2-phenylacetate (4q). Prepared according to the general procedure using 0.0687 g (0.30 mmol) of 2-(4-ethynylphenyl)-4,4,5,5-tetramethyl-1,3,2-dioxaborolane, 56 μL (0.99 g/mL, 0.30 mmol) of diphenylsilane, 0.0024 g (0.0030 mmol) of Xantphos•$CoBr_2$, 9 μL (1.0 M in THF, 0.0090 mmol) of $NaBHEt_3$, 1.2 mL (0.25 M) of Et_2O, 0.0020 g (0.0063 mmol) of **L5**, 45 μL (0.88 g/mL, 0.36 mmol) of phenylsilane, 48 μL (1.19 g/mL, 0.30 mmol) of ethyl 2-diazo-2-phenylacetate, and 16 μL (1.00 g/mL, 0.90 mmol) of H_2O. After 12 h, the resulting solution was quenched. The combined filtrate was concentrated and the crude mixture was purified by short flash column chromatography using PE to PE/EtOAc = 20/1 as the eluent to give 0.0925 g (0.15 mmol, 51% yield) of the title compound as a colorless oil. IR (cm^{-1}): 3248, 3050, 2929, 2134, 1670, 1513. ^1H NMR: (400 MHz, $CDCl_3$) δ 10.80 (d, J = 5.2 Hz, 1H), 7.74 (d, J = 8.0 Hz, 2H), 7.55-7.50 (m, 2H), 7.49-7.42 (m, 4H), 7.37-7.25 (m, 10H), 7.24-7.20 (m, 1H), 4.85-4.76 (m, 2H), 4.21 (q, J = 7.2 Hz, 2H), 2.16-2.07 (m, 1H), 1.89-1.80 (m, 1H), 1.33 (s, 12H), 1.28 (t, J = 7.2 Hz, 3H); ^{13}C NMR: (100 MHz, $CDCl_3$) δ 163.4, 146.6, 137.1, 135.2, 135.1, 135.0, 133.6, 133.4, 129.64, 129.56, 128.3, 128.0, 127.9, 127.6, 126.7, 126.1, 125.9, 83.7, 62.2, 60.2, 24.8, 20.8, 14.2; HRMS (ESI) calculated for $[C_{36}H_{41}BN_2O_4SiNa]^+$ $[M+Na]^+$ requires m/z 627.2821, found m/z 627.2826.

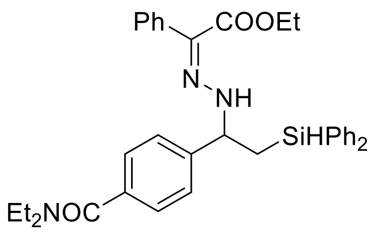

Ethyl (*Z*)-2-(2-(1-(4-(diethylcarbamoyl)phenyl)-2-(diphenylsilyl)ethyl)hydrazono)-2-phenylacetate (4r). Prepared according to the general procedure using 0.0605 g (0.30 mmol) of *N*,

N-diethyl-4-ethynylbenzamide, 56 μL (0.99 g/mL, 0.30 mmol) of diphenylsilane, 0.0024 g (0.0030 mmol) of Xantphos•$CoBr_2$, 9 μL (1.0 M in THF, 0.0090 mmol) of $NaBHEt_3$, 1.2 mL (0.25 M) of Et_2O, 0.0020 g (0.0063 mmol) of **L5**, 45 μL (0.88 g/mL, 0.36 mmol) of phenylsilane, 48 μL (1.19 g/mL, 0.30 mmol) of ethyl 2-diazo-2-phenylacetate, and 16 μL (1.00 g/mL, 0.90 mmol) of H_2O. After 12 h, the resulting solution was quenched. The combined filtrate was concentrated and the crude mixture was purified by short flash column chromatography using PE to PE/EtOAc = 1/1 as the eluent to give 0.1510 g (0.26 mmol, 87% yield) of the title compound as a colorless oil. IR (cm^{-1}): 3240, 3050, 2932, 2132, 1671, 1630, 1514. ^{1}H NMR: (400 MHz, $CDCl_3$) δ 10.79 (brs, 1H), 7.55-7.51 (m, 2H), 7.51-7.47 (m, 2H), 7.47-7.42 (m, 2H), 7.40-7.25 (m, 13H), 4.87-4.78 (m, 2H), 4.22 (q, J = 7.2 Hz, 2H), 3.52 (br, 2H), 3.22 (br, 2H), 2.17-2.06 (m, 1H), 1.90-1.80 (m, 1H), 1.29 (t, J = 7.2 Hz, 3H), 1.22 (br, 3H), 1.09 (br, 3H); ^{13}C NMR: (100 MHz, $CDCl_3$) δ 171.0, 163.4, 144.6, 136.9, 136.2, 135.1, 135.0, 133.4, 133.3, 129.7, 129.6, 128.2, 127.99, 127.95, 127.6, 126.8, 126.7, 126.6, 126.1, 61.8, 60.3, 43.2, 39.1, 20.7, 14.1, 12.8; HRMS (ESI) calculated for $[C_{35}H_{39}N_3O_3SiNa]^+$ $[M+Na]^+$ requires m/z 600.2653, found m/z 600.2650.

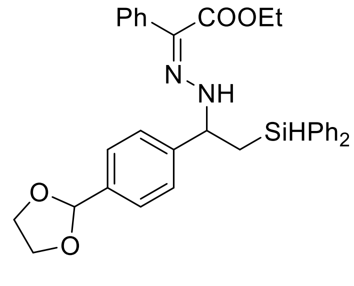

Ethyl (*Z*)-2-(2-(1-(4-(1,3-dioxolan-2-yl)phenyl)-2-(diphenylsilyl)ethyl)hydrazono)-2-phenylacetate (4s). Prepared according to the general procedure using 0.0520 g (0.30 mmol) of 2-(4-ethynylphenyl)-1,3-dioxolane, 56 μL (0.99 g/mL, 0.30 mmol) of diphenylsilane, 0.0024 g (0.0030 mmol) of Xantphos•$CoBr_2$, 9 μL (1.0 M in THF, 0.0090 mmol) of $NaBHEt_3$, 1.2 mL (0.25 M) of Et_2O, 0.0022 g (0.0069 mmol) of **L5**, 45 μL (0.88 g/mL, 0.36 mmol) of phenylsilane, 48 μL (1.19 g/mL, 0.30 mmol) of ethyl 2-diazo-2-phenylacetate, and 16 μL (1.00 g/mL, 0.90 mmol) of H_2O. After 12 h, the resulting solution was quenched. The combined filtrate was concentrated and the crude mixture was purified by short flash column chromatography using PE to PE/EtOAc = 20/1 as the eluent to give 0.1041g (0.19 mmol, 63% yield) of the title compound as a colorless oil.

IR (cm^{-1}): 3260, 3051, 2924, 2133, 1702, 1671, 1515. ^1H NMR: (400 MHz, $CDCl_3$) δ 10.79 (brs, 1H), 7.55-7.50 (m, 2H), 7.49-7.42 (m, 4H), 7.42-7.38 (m, 2H), 7.37-7.25 (m, 10H), 7.25-7.19 (m, 1H), 5.78 (s, 1H), 4.85-4.77 (m, 2H), 4.21 (q, J = 7.2 Hz, 2H), 4.12-4.06 (m, 2H), 4.05-3.95 (m, 2H), 2.17-2.08 (m, 1H), 1.88-1.79 (m, 1H), 1.28 (t, J = 7.2 Hz, 3H); ^{13}C NMR: (100 MHz, $CDCl_3$) δ 163.4, 144.5, 137.0, 135.2, 135.0, 133.5, 133.4, 129.64, 129.58, 128.3, 128.0, 127.9, 127.6, 126.73, 126.70, 125.9, 103.5, 65.2, 61.9, 60.3, 20.8, 14.2; HRMS (ESI) calculated for $[C_{33}H_{34}N_2O_4SiNa]^+$ $[M+Na]^+$ requires m/z 573.2180, found m/z 573.2186.

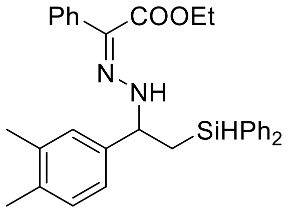

Ethyl (*Z*)-2-(2-(1-(3,4-dimethylphenyl)-2-(diphenylsilyl)ethyl)hydrazono)-2-phenylacetate (4t). Prepared according to the general procedure using 0.0392 g (0.30 mmol) of 4-ethynyl-1,2-dimethylbenzene, 56 μL (0.99 g/mL, 0.30 mmol) of diphenylsilane, 0.0024 g (0.0030 mmol) of Xantphos•$CoBr_2$, 9 μL (1.0 M in THF, 0.0090 mmol) of $NaBHEt_3$, 1.2 mL (0.25 M) of Et_2O, 0.0021 g (0.0066 mmol) of **L5**, 45 μL (0.88 g/mL, 0.36 mmol) of phenylsilane, 48 μL (1.19 g/mL, 0.30 mmol) of ethyl 2-diazo-2-phenylacetate, and 16 μL (1.00 g/mL, 0.90 mmol) of H_2O. After 12 h, the resulting solution was quenched. The combined filtrate was concentrated and the crude mixture was purified by short flash column chromatography using PE to PE/EtOAc = 200/1 as the eluent to give 0.1216 g (0.24 mmol, 80% yield) of the title compound as a colorless oil. IR (cm^{-1}): 3248, 3067, 2923, 2132, 1736, 1670, 1511. ^1H NMR: (400 MHz, $CDCl_3$) δ 10.81 (brs, 1H), 7.55-7.42 (m, 6H), 7.39-7.17 (m, 9H), 7.05-6.97 (m, 3H), 4.82-4.73 (m, 2H), 4.20 (q, J = 7.2 Hz, 2H), 2.19 (s, 3H), 2.18 (s, 3H), 2.17-2.10 (m, 1H), 1.89-1.80 (m, 1H), 1.27 (t, J = 7.2 Hz, 3H); ^{13}C NMR: (100 MHz, $CDCl_3$) δ 163.5, 140.6, 137.2, 136.6, 135.7, 135.2, 135.0, 133.8, 133.7, 129.8, 129.5, 129.4, 128.3, 128.0, 127.9, 127.8, 127.6, 126.6, 125.2, 124.1, 62.1, 60.2, 21.1, 19.8, 19.4, 14.2; HRMS (ESI) calculated for $[C_{32}H_{35}N_2O_2Si]^+$ $[M+H]^+$ requires m/z 507.2462, found m/z 507.2465.

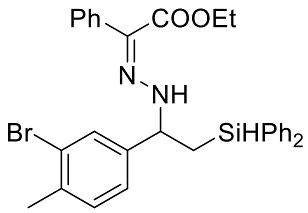

Ethyl (*Z*)-2-(2-(1-(3-bromo-4-methylphenyl)-2-(diphenylsilyl)ethyl)hydrazono)-2-phenylacetate (4u). Prepared according to the general procedure using 0.0588 g (0.30 mmol) of 2-bromo-4-ethynyl-1-methylbenzene, 56 μL (0.99 g/mL, 0.30 mmol) of diphenylsilane, 0.0024 g (0.0030 mmol) of Xantphos•$CoBr_2$, 9 μL (1.0 M in THF, 0.0090 mmol) of $NaBHEt_3$, 1.2 mL (0.25 M) of Et_2O, 0.0020 g (0.0063 mmol) of **L5**, 45 μL (0.88 g/mL, 0.36 mmol) of phenylsilane, 48 μL (1.19 g/mL, 0.30 mmol) of ethyl 2-diazo-2-phenylacetate, and 16 μL (1.00 g/mL, 0.90 mmol) of H_2O. After 12 h, the resulting solution was quenched. The combined filtrate was concentrated and the crude mixture was purified by short flash column chromatography using PE to PE/EtOAc = 100/1 as the eluent to give 0.0909 g (0.16 mmol, 53% yield) of the title compound as a light yellow oil. IR (cm^{-1}): 3428, 3066, 2924, 2133, 1735, 1687, 1514. ^1H NMR: (400 MHz, $CDCl_3$) δ 10.74 (brs, 1H), 7.55-7.49 (m, 2H), 7.48-7.42 (m, 4H), 7.40 (s, 1H), 7.37-7.20 (m, 9H), 7.10-7.06 (m, 2H), 4.80 (t, *J* = 4.0 Hz, 1H), 4.73 (t, *J* = 8.0 Hz, 1H), 4.22 (q, *J* = 7.2 Hz, 2H), 2.32 (s, 3H), 2.13-2.05 (m, 1H), 1.85-1.77 (m, 1H), 1.29 (t, *J* = 7.2 Hz, 3H); ^{13}C NMR: (100 MHz, $CDCl_3$) δ 163.4, 142.9, 137.0, 136.8, 135.2, 135.0, 133.3, 130.9, 130.7, 129.7, 129.6, 128.3, 128.0, 127.9, 127.6, 126.9, 126.1, 125.6, 124.9, 61.3, 60.3, 22.5, 20.8, 14.2; HRMS (ESI) calculated for $[C_{31}H_{31}BrN_2O_2SiNa]^+$ $[M+Na]^+$ requires m/z 593.1230, found m/z 593.1227.

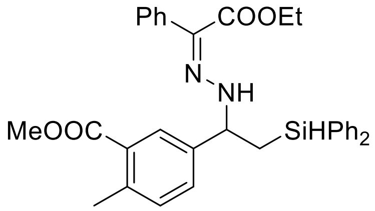

Methyl (*Z*)-5-(2-(diphenylsilyl)-1-(2-(2-ethoxy-2-oxo-1-phenylethylidene)hydrazinyl)ethyl)-2-methylbenzoate (4v). Prepared according to the general procedure using 0.0525 g (0.30 mmol) of methyl 5-ethynyl-2-methylbenzoate, 56 μL (0.99 g/mL, 0.30 mmol) of diphenylsilane, 0.0024 g (0.0030 mmol) of Xantphos•$CoBr_2$, 9 μL (1.0 M in THF, 0.0090 mmol) of $NaBHEt_3$, 1.2 mL (0.25 M) of Et_2O, 0.0021 g (0.0066 mmol) of **L5**, 45 μL (0.88 g/mL, 0.36 mmol) of phenylsilane, 48 μL

(1.19 g/mL, 0.30 mmol) of ethyl 2-diazo-2-phenylacetate, and 16 μL (1.00 g/mL, 0.90 mmol) of H_2O. After 12 h, the resulting solution was quenched. The combined filtrate was concentrated and the crude mixture was purified by short flash column chromatography using PE to PE/EtOAc = 50/1 as the eluent to give 0.1107 g (0.20 mmol, 67% yield) of the title compound as a colorless oil. IR (cm^{-1}): 3251, 3067, 2952, 2133, 1724, 1689, 1514. ^{1}H NMR: (400 MHz, $CDCl_3$) δ 10.78 (brs, 1H), 7.82 (d, J = 1.6 Hz, 1H), 7.55-7.49 (m, 2H), 7.48-7.43 (m, 4H), 7.40-7.25 (m, 9H), 7.25-7.20 (m, 1H), 7.13 (d, J = 8.0 Hz, 1H), 4.85-4.76 (m, 2H), 4.21 (q, J = 7.2 Hz, 2H), 3.86 (s, 3H), 2.55 (s, 3H), 2.19-2.09 (m, 1H), 1.90-1.81 (m, 1H), 1.28 (t, J = 7.2 Hz, 3H); ^{13}C NMR: (100 MHz, $CDCl_3$) δ 167.7, 163.4, 140.8, 139.4, 137.0, 135.2, 135.0, 133.4, 131.9, 130.4, 129.6, 129.5, 129.4, 129.2, 128.3, 128.0, 127.9, 127.6, 126.8, 126.0, 61.5, 60.3, 51.7, 21.4, 20.7, 14.1; HRMS (ESI) calculated for $[C_{33}H_{35}N_2O_4Si]^+$ $[M+H]^+$ requires m/z 551.2361, found m/z 551.2362.

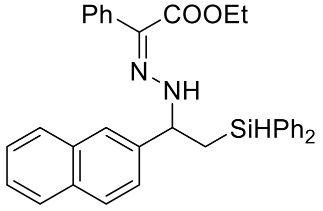

Ethyl (*Z*)-2-(2-(2-(diphenylsilyl)-1-(naphthalen-2-yl)ethyl)hydrazono)-2-phenylacetate (4w). Prepared according to the general procedure using 0.0458 g (0.30 mmol) of 2-ethynylnaphthalene, 56 μL (0.99 g/mL, 0.30 mmol) of diphenylsilane, 0.0024 g (0.0030 mmol) of Xantphos•$CoBr_2$, 9 μL (1.0 M in THF, 0.0090 mmol) of $NaBHEt_3$, 1.2 mL (0.25 M) of Et_2O, 0.0019 g (0.0060 mmol) of **L5**, 45 μL (0.88 g/mL, 0.36 mmol) of phenylsilane, 48 μL (1.19 g/mL, 0.30 mmol) of ethyl 2-diazo-2-phenylacetate, and 16 μL (1.00 g/mL, 0.90 mmol) of H_2O. After 12 h, the resulting solution was quenched. The combined filtrate was concentrated and the crude mixture was purified by short flash column chromatography using PE to PE/EtOAc = 100/1 as the eluent to give 0.1190 g (0.22 mmol, 75% yield) of the title compound as a light yellow oil. IR (cm^{-1}): 3436, 2981, 2906, 2133, 1735, 1686, 1510. ^{1}H NMR: (400 MHz, $CDCl_3$) δ 10.90 (d, J = 5.2 Hz, 1H), 7.80-7.70 (m, 3H), 7.65-7.61 (m, 1H), 7.55-7.50 (m, 2H), 7.49-7.40 (m, 7H), 7.37-7.18 (m, 9H), 5.02-4.93 (m, 1H), 4.79 (t, J = 4.0 Hz, 1H), 4.21 (q, J = 6.8 Hz, 2H), 2.27-2.17 (m, 1H), 2.00-1.90 (m, 1H), 1.27 (t, J = 7.2 Hz, 3H); ^{13}C NMR: (100 MHz, $CDCl_3$) δ 163.5, 140.6, 137.1, 135.2, 135.0, 133.53, 133.49, 133.2, 132.8, 129.7, 129.5, 128.5, 128.3, 128.0, 127.94, 127.90, 127.62,

127.58, 126.8, 126.0, 125.81, 125.78, 125.6, 124.8, 62.3, 60.3, 20.7, 14.2; HRMS (ESI) calculated for $[C_{34}H_{32}N_2O_2SiNa]^+$ $[M+Na]^+$ requires m/z 551.2125, found m/z 515.2123.

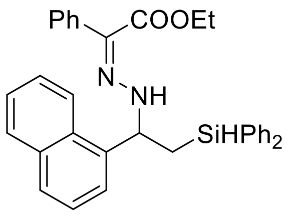

Ethyl (*Z*)-2-(2-(2-(diphenylsilyl)-1-(naphthalen-1-yl)ethyl)hydrazono)-2-phenylacetate (4x). Prepared according to the general procedure using 0.0462 g (0.30 mmol) of 1-ethynylnaphthalene, 56 μL (0.99 g/mL, 0.30 mmol) of diphenylsilane, 0.0024 g (0.0030 mmol) of Xantphos•$CoBr_2$, 9 μL (1.0 M in THF, 0.0090 mmol) of $NaBHEt_3$, 1.2 mL (0.25 M) of Et_2O, 0.0020 g (0.0063 mmol) of **L5**, 45 μL (0.88 g/mL, 0.36 mmol) of phenylsilane, 48 μL (1.19 g/mL, 0.30 mmol) of ethyl 2-diazo-2-phenylacetate, and 16 μL (1.00 g/mL, 0.90 mmol) of H_2O. After 12 h, the resulting solution was quenched. The combined filtrate was concentrated and the crude mixture was purified by short flash column chromatography using PE to PE/EtOAc = 100/1 as the eluent to give 0.0936 g (0.18 mmol, 59% yield) of the title compound as a colorless oil. IR (cm^{-1}): 3242, 3050, 2132, 1954, 1669, 1512. ^1H NMR: (400 MHz, $CDCl_3$) δ 10.91 (brs, 1H), 7.96-7.90 (m, 1H), 7.84-7.78 (m, 1H), 7.74-7.68 (m, 1H), 7.54-7.46 (m, 7H), 7.44-7.40 (m, 2H), 7.40-7.25 (m, 8H), 7.25-7.17 (m, 2H), 5.70-5.62 (m, 1H), 4.91 (t, J = 4.0 Hz, 1H), 4.20 (q, J = 7.2 Hz, 2H), 2.26-2.17 (m, 1H), 2.10-2.02 (m, 1H), 1.30-1.24 (t, J = 7.2 Hz, 3H);^{13}C NMR: (100 MHz, $CDCl_3$) δ 163.5, 139.2, 137.1, 135.14, 135.11, 133.9, 133.6, 133.5, 130.6, 129.64, 129.58, 128.8, 128.3, 127.99, 127.96, 127.9, 127.6, 126.7, 126.1, 125.6, 125.5, 125.4, 123.5, 123.1, 60.3, 58.4, 20.8, 14.2; HRMS (ESI) calculated for $[C_{34}H_{32}N_2O_2SiNa]^+$ $[M+Na]^+$ requires m/z 551.2125, found m/z 551.2123.

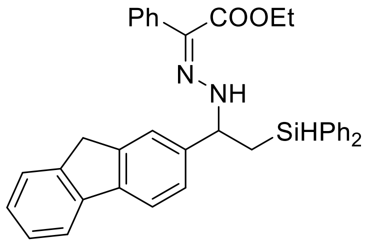

Ethyl (*Z*)-2-(2-(2-(diphenylsilyl)-1-(9*H*-fluoren-2-yl)ethyl)hydrazono)-2-phenylacetate (4y).

Prepared according to the general procedure using 0.0480 g (0.25 mmol) of 2-ethynyl-9*H*-fluorene, 56 μL (0.99 g/mL, 0.30 mmol) of diphenylsilane, 0.0025 g (0.0031 mmol) of Xantphos•$CoBr_2$, 9 μL (1.0 M in THF, 0.0090 mmol) of $NaBHEt_3$, 1.2 mL (0.25 M) of Et_2O, 0.0020 g (0.0063 mmol) of **L5**, 45 μL (0.88 g/mL, 0.36 mmol) of phenylsilane, 48 μL (1.19 g/mL, 0.30 mmol) of ethyl 2-diazo-2-phenylacetate, and 16 μL (1.00 g/mL, 0.90 mmol) of H_2O. After 12 h, the resulting solution was quenched. The combined filtrate was concentrated and the crude mixture was purified by short flash column chromatography using PE to PE/EtOAc = 100/1 as the eluent to give 0.1234 g (0.22 mmol, 86% yield) of the title compound as a colorless oil. IR (cm^{-1}): 3247, 3065, 2901, 2131, 1735, 1669, 1512. ^1H NMR: (400 MHz, $CDCl_3$) δ 10.89 (brs, 1H), 7.73 (d, J = 7.6 Hz, 1H), 7.67 (d, J = 7.6 Hz, 1H), 7.55-7.42 (m, 7H), 7.38 (s, 1H), 7.36-7.20 (m, 12H), 4.89 (t, J = 8.0 Hz, 1H), 4.80 (t, J = 3.6 Hz, 1H), 4.21 (q, J = 7.2 Hz, 2H), 3.78 (s, 2H), 2.25-2.15 (m, 1H), 1.96-1.86 (m, 1H), 1.27 (m, J = 7.2 Hz, 3H); ^{13}C NMR: (100 MHz, $CDCl_3$) δ 163.5, 143.6, 143.3, 141.8, 141.4, 141.1, 137.1, 135.2, 135.0, 133.64, 133.59, 129.6, 129.5, 128.3, 128.0, 127.9, 127.6, 126.73, 126.67, 126.6, 125.6, 124.9, 123.5, 119.9, 119.8, 62.4, 60.2, 36.8, 21.1, 14.2; HRMS (ESI) calculated for $[C_{37}H_{35}N_2O_2Si]^+$ $[M+H]^+$ requires m/z 567.2462, found m/z 567.2465.

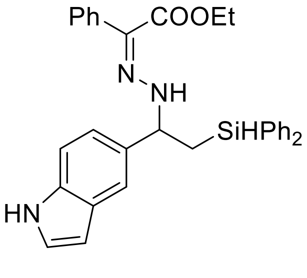

Ethyl (*Z*)-2-(2-(2-(diphenylsilyl)-1-(1H-indol-5-yl)ethyl)hydrazono)-2-phenylacetate (4z). Prepared according to the general procedure using 0.0425 g (0.30 mmol) of 5-ethynyl-1H-indole, 56 μL (0.99 g/mL, 0.30 mmol) of diphenylsilane, 0.0024 g (0.0030 mmol) of Xantphos•$CoBr_2$, 9 μL (1.0 M in THF, 0.0090 mmol) of $NaBHEt_3$, 1.2 mL (0.25 M) of Et_2O, 0.0020 g (0.0063 mmol) of **L5**, 45 μL (0.88 g/mL, 0.36 mmol) of phenylsilane, 48 μL (1.19 g/mL, 0.30 mmol) of ethyl 2-diazo-2-phenylacetate, and 16 μL (1.00 g/mL, 0.90 mmol) of H_2O. After 12 h, the resulting solution was quenched. The combined filtrate was concentrated and the crude mixture was purified by short flash column chromatography using PE to PE/EtOAc = 10/1 as the eluent to give 0.0947 g (0.18 mmol, 61% yield) of the title compound as a colorless oil. IR (cm^{-1}): 3250, 3053,

2925, 2134, 1668, 1511. ^{1}H NMR: (400 MHz, $CDCl_3$) δ 10.88 (d, J = 4.8 Hz, 1H), 8.08 (s, 1H), 7.55-7.49 (m, 4H), 7.49-7.43 (m, 3H), 7.37-7.25 (m, 10H), 7.17-.714 (m, 1H), 7.12 (dd, J = 8.4, 1.2 Hz, 1H), 6.50-6.46 (m, 1H), 4.95-4.88 (m, 1H), 4.75 (t, J = 3.6 Hz, 1H), 4.20 (q, J = 7.2 Hz, 2H), 2.29-2.20 (m, 1H), 1.98-1.88 (m, 1H), 1.27 (t, J = 7.2 Hz, 3H); ^{13}C NMR: (100 MHz, $CDCl_3$) δ 163.5, 137.3, 135.2, 135.0, 134.5, 133.9, 133.8, 129.5, 129.4, 128.3, 127.9, 127.8, 127.6, 126.6, 124.9, 124.6, 121.0, 118.9, 111.2, 102.7, 62.7, 60.1, 21.4, 14.2; HRMS (ESI) calculated for $[C_{32}H_{31}N_3O_2SiNa]^+$ $[M+Na]^+$ requires m/z 540.2078, found m/z 540.2075.

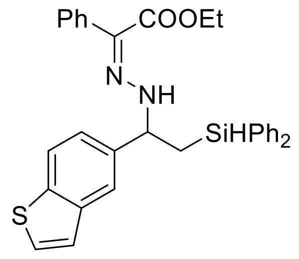

Ethyl (*Z*)-2-(2-(1-(benzo[b]thiophen-5-yl)-2-(diphenylsilyl)ethyl)hydrazono)-2-phenylacetate (4aa). Prepared according to the general procedure using 0.0475 g (0.30 mmol) of 5-ethynylbenzo[*b*]thiophene, 56 μL (0.99 g/mL, 0.30 mmol) of diphenylsilane, 0.0024 g (0.0030 mmol) of Xantphos•$CoBr_2$, 9 μL (1.0 M in THF, 0.0090 mmol) of $NaBHEt_3$, 1.2 mL (0.25 M) of Et_2O, 0.0019 g (0.0060 mmol) of **L5**, 45 μL (0.88 g/mL, 0.36 mmol) of phenylsilane, 48 μL (1.19 g/mL, 0.30 mmol) of ethyl 2-diazo-2-phenylacetate, and 16 μL (1.00 g/mL, 0.90 mmol) of H_2O. After 12 h, the resulting solution was quenched. The combined filtrate was concentrated and the crude mixture was purified by short flash column chromatography using PE to PE/EtOAc = 100/1 as the eluent to give 0.1251 g (0.23 mmol, 78% yield) of the title compound as a colorless oil. IR (cm^{-1}): 3417, 3067, 2982, 2133, 1735, 1686, 1595. ^{1}H NMR: (400 MHz, $CDCl_3$) δ 10.84 (brs, 1H), 7.77 (d, J = 8.4 Hz, 1H), 7.66 (d, J = 1.2 Hz, 1H), 7.55-7.50 (m, 2H), 7.49-7.40 (m, 5H), 7.38-7.20 (m, 11H), 4.94 (t, J = 8.0 Hz, 1H), 4.78 (t, J = 3.6 Hz, 1H), 4.22 (q, J = 7.2 Hz, 2H), 2.26-2.16 (m, 1H), 1.97-1.87 (m, 1H), 1.28 (t, J = 7.2 Hz, 3H); ^{13}C NMR: (100 MHz, $CDCl_3$) δ 163.5, 139.7, 139.5, 138.8, 137.1, 135.2, 135.0, 133.5, 129.6, 129.5, 128.3, 128.0, 127.9, 127.6, 126.8, 125.7, 123.9, 123.3, 122.7, 121.7, 62.3, 60.3, 21.2, 14.2; HRMS (ESI) calculated for $[C_{32}H_{30}N_2O_2SSiNa]^+$ $[M+Na]^+$ requires m/z 557.1689, found m/z 557.1690.

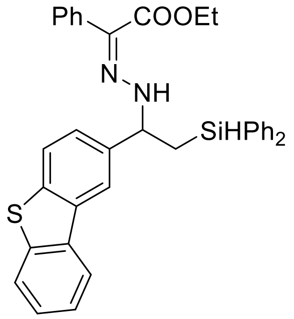

Ethyl (*Z*)-2-(2-(1-(dibenzo[*b,d*]thiophen-2-yl)-2-(diphenylsilyl)ethyl)hydrazono)-2-phenylacetate (4ab). Prepared according to the general procedure using 0.0625 g (0.30 mmol) of 2-ethynyldibenzo[*b,d*]thiophene, 56 μL (0.99 g/mL, 0.30 mmol) of diphenylsilane, 0.0024 g (0.0030 mmol) of Xantphos•$CoBr_2$, 9 μL (1.0 M in THF, 0.0090 mmol) of $NaBHEt_3$, 1.2 mL (0.25 M) of Et_2O, 0.0019 g (0.0060 mmol) of **L5**, 45 μL (0.88 g/mL, 0.36 mmol) of phenylsilane, 48 μL (1.19 g/mL, 0.30 mmol) of ethyl 2-diazo-2-phenylacetate, and 16 μL (1.00 g/mL, 0.90 mmol) of H_2O. After 12 h, the resulting solution was quenched. The combined filtrate was concentrated and the crude mixture was purified by short flash column chromatography using PE to PE/EtOAc = 100/1 as the eluent to give 0.1361 g (0.23 mmol, 78% yield) of the title compound as a colorless oil. IR (cm^{-1}): 3240, 3001, 2133, 1734, 1684, 1593. ^1H NMR: (400 MHz, $CDCl_3$) δ 10.91 (brs, 1H), 8.07-8.00 (m, 1H), 7.95 (s, 1H), 7.85 (m, 1H), 7.73 (d, J = 8.4 Hz, 1H), 7.55-7.50 (m, 2H), 7.50-7.40 (m, 6H), 7.40-7.34 (m, 2H), 7.34-7.20 (m, 8H), 4.98 (t, J = 8.0 Hz, 1H), 4.77 (t, J = 4.0 Hz, 1H), 4.22 (q, J = 7.2 Hz, 2H), 2.30-2.20 (m, 1H), 2.21-1.92 (m, 1H), 1.28 (t, J = 7.2 Hz, 3H); ^{13}C NMR: (100 MHz, $CDCl_3$) δ 163.5, 139.8, 139.6, 138.5, 137.1, 135.6, 135.4, 135.2, 135.0, 133.5, 129.7, 129.5, 128.3, 128.0, 127.9, 127.7, 126.8, 126.7, 126.0, 125.6, 124.3, 123.0, 122.8, 121.7, 120.1, 62.3, 60.3, 21.1, 14.2; HRMS (ESI) calculated for $[C_{36}H_{32}N_2O_2SSiNa]^+$ $[M+Na]^+$ requires m/z 607.1846, found m/z 607.1848.

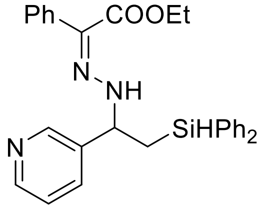

Ethyl (*Z*)-2-(2-(2-(diphenylsilyl)-1-(pyridin-3-yl)ethyl)hydrazono)-2-phenylacetate (4ac).

Prepared according to the general procedure using 0.0310 g (0.30 mmol) of 3-ethynylpyridine, 56 μL (0.99 g/mL, 0.30 mmol) of diphenylsilane, 0.0024 g (0.0030 mmol) of Xantphos•$CoBr_2$, 9 μL (1.0 M in THF, 0.0090 mmol) of $NaBHEt_3$, 1.2 mL (0.25 M) of Et_2O, 0.0019 g (0.0060 mmol) of **L5**, 45 μL (0.88 g/mL, 0.36 mmol) of phenylsilane, 48 μL (1.19 g/mL, 0.30 mmol) of ethyl 2-diazo-2-phenylacetate, and 16 μL (1.00 g/mL, 0.90 mmol) of H_2O. After 12 h, the resulting solution was quenched. The combined filtrate was concentrated and the crude mixture was purified by short flash column chromatography using PE to PE/EtOAc = 5/1 as the eluent to give 0.0662 g (0.14 mmol, 46% yield) of the title compound as a colorless oil. IR (cm^{-1}): 3252, 3051, 2131, 1672, 1515, 1427. ^1H NMR: (400 MHz, $CDCl_3$) δ 10.75 (d, J = 4.8 Hz, 1H), 8.50 (d, J = 2.0 Hz, 1H), 8.46 (dd, J = 4.8, 1.2 Hz, 1H), 7.60-7.55 (m, 1H), 7.54-7.50 (m, 2H), 7.50-7.45 (m, 2H), 7.45-7.40 (m, 2H), 7.40-7.23 (m, 9H), 7.17 (dd, J = 8.0, 4.8 Hz, 1H), 4.86-4.78 (m, 2H), 4.23 (q, J = 7.2 Hz, 2H), 2.18-2.08 (m, 1H), 1.92-1.82 (m, 1H), 1.29 (t, J = 7.2 Hz, 3H); ^{13}C NMR: (100 MHz, $CDCl_3$) δ 163.3, 148.8, 148.6, 138.8, 136.8, 135.1, 135.0, 134.2, 133.03, 132.97, 129.83, 129.76, 128.2, 128.11, 128.06, 127.7, 127.0, 126.9, 123.4, 60.5, 59.6, 20.5, 14.1; HRMS (ESI) calculated for $[C_{29}H_{30}N_3O_2Si]^+$ $[M+H]^+$ requires m/z 480.2102, found m/z 480.2103.

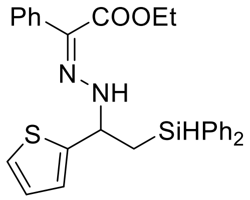

Ethyl (*Z*)-2-(2-(2-(diphenylsilyl)-1-(thiophen-2-yl)ethyl)hydrazono)-2-phenylacetate (4ad). Prepared according to the general procedure using 0.0324 g (0.30 mmol) of 2-ethynylthiophene, 56 μL (0.99 g/mL, 0.30 mmol) of diphenylsilane, 0.0024 g (0.0030 mmol) of Xantphos•$CoBr_2$, 9 μL (1.0 M in THF, 0.0090 mmol) of $NaBHEt_3$, 1.2 mL (0.25 M) of Et_2O, 0.0019 g (0.0060 mmol) of **L5**, 45 μL (0.88 g/mL, 0.36 mmol) of phenylsilane, 48 μL (1.19 g/mL, 0.30 mmol) of ethyl 2-diazo-2-phenylacetate, and 16 μL (1.00 g/mL, 0.90 mmol) of H_2O. After 12 h, the resulting solution was quenched. The combined filtrate was concentrated and the crude mixture was purified by short flash column chromatography using PE to PE/EtOAc = 100/1 as the eluent to give 0.1003 g (0.21 mmol, 69% yield) of the title compound as a colorless oil. IR (cm^{-1}): 3252, 3066, 2924, 2132, 1735, 1672, 1513. ^1H NMR: (400 MHz, $CDCl_3$) δ 10.64 (d, J = 5.6 Hz, 1H),

7.57-7.47 (m, 6H), 7.40-7.27 (m, 8H), 7.26-7.21 (m, 1H), 7.18 (dd, J = 4.4, 1.2 Hz, 1H), 6.93-6.87 (m, 2H), 5.12-5.03 (m, 1H), 4.85 (t, J = 4.0 Hz, 1H), 4.21 (q, J = 7.2 Hz, 2H), 2.23-2.13 (m, 1H), 2.03-1.93 (m, 1H), 1.28 (t, J = 7.2 Hz, 3H); ^{13}C NMR: (100 MHz, $CDCl_3$) δ 163.3, 147.3, 137.0, 135.2, 135.1, 133.45, 133.38, 129.7, 129.6, 128.3, 128.00, 127.99, 127.6, 126.9, 126.5, 126.4, 124.7, 124.3, 60.4, 57.7, 21.6, 14.2; HRMS (ESI) calculated for $[C_{28}H_{28}N_2O_2SSiNa]^+$ $[M+Na]^+$ requires m/z 507.1533, found m/z 507.1534.

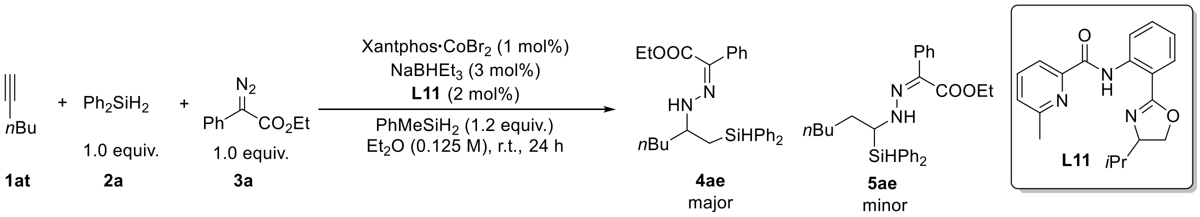

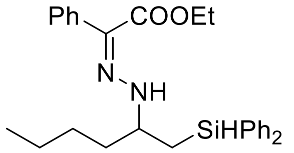

Ethyl (*Z*)-2-(2-(1-(diphenylsilyl)hexan-2-yl)hydrazono)-2-phenylacetate (4ae). Prepared according to the general procedure using 35 μL (0.72 g/mL, 0.30 mmol) of hex-1-yne, 56 μL (0.99 g/mL, 0.30 mmol) of diphenylsilane, 0.0024 g (0.0030 mmol) of Xantphos•$CoBr_2$, 9 μL (1.0 M in THF, 0.0090 mmol) of $NaBHEt_3$, 2.4 mL (0.125 M) of Et_2O, 0.0020 g (0.0063 mmol) of **L5**, 0.0440 g (0.36 mmol) of methyl(phenyl)silane, and 48 μL (1.19 g/mL, 0.30 mmol) of ethyl 2-diazo-2-phenylacetate. After 24 h, the resulting solution was quenched. The combined filtrate was concentrated and the crude mixture was purified by short flash column chromatography using PE to PE/EtOAc = 100/1 as the eluent to give 0.0443 g (0.10 mmol, 32% yield, 7/1 **4ae**/**5ae**) of the title compound as a colorless oil. IR (cm^{-1}): 3468, 3051, 2926, 2126, 1735, 1690, 1510. ^{1}H NMR: (400 MHz, $CDCl_3$) δ 10.92 (d, J = 4.8 Hz, 0.14H, **5ae**), 10.59 (d, J = 5.6 Hz, 1H, **4ae**), 7.58-7.51 (m, 4H), 7.50-7.45 (m, 2H), 7.40-7.26 (m, 9H), 7.25-7.20 (m, 1H), 5.01 (d, J = 2.4 Hz, 0.13H, **5ae**), 4.95 (t, J = 3.6 Hz, 1H, **4ae**), 4.21 (q, J = 7.2 Hz, 2H), 3.88-3.82 (m, 0.13H, **5ae**), 3.81-3.70 (m, 1H, **4ae**), 1.77-1.62 (m, 3H), 1.60-1.55 (m, 1H), 1.34-1.25 (m, 7H), 0.84 (t, J = 7.2 Hz, 3H); HRMS (ESI) calculated for $[C_{28}H_{35}N_2O_2Si]^+$ $[M+H]^+$ requires m/z 459.2462, found m/z 459.2463.

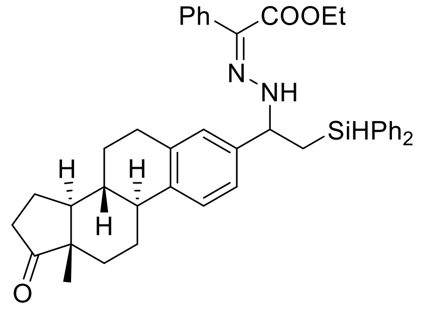

Ethyl (*Z*)-2-(2-(2-(diphenylsilyl)-1-((8*R*,9*S*,13*S*,14*S*)-13-methyl-17-oxo-7,8,9,11,12,13,14,15,16,17-decahydro-6*H*-cyclopenta[*a*]phenanthren-3-yl)ethyl)hydrazono)-2-phenylacetate (4af). Prepared according to the general procedure using 0.0835 g (0.30 mmol) of (8*R*,9*S*,13*S*,14*S*)-3-ethynyl-13-methyl-6,7,8,9,11,12,13,14,15,16-decahydro-17*H*-cyclopenta[*a*]phenanthren-17-one, 56 μL (0.99 g/mL, 0.30 mmol) of diphenylsilane, 0.0025 g (0.0031 mmol) of Xantphos•$CoBr_2$, 9 μL (1.0 M in THF, 0.0090 mmol) of $NaBHEt_3$, 1.2 mL (0.25 M) of Et_2O, 0.0020 g (0.0063 mmol) of **L5**, 45 μL (0.88 g/mL, 0.36 mmol) of phenylsilane, 48 μL (1.19 g/mL, 0.30 mmol) of ethyl 2-diazo-2-phenylacetate, and 16 μL (1.00 g/mL, 0.90 mmol) of H_2O. After 12 h, the resulting solution was quenched. The combined filtrate was concentrated and the crude mixture was purified by short flash column chromatography using PE to PE/EtOAc = 10/1 as the eluent to give 0.1120 g (0.17 mmol, 57% yield) of the title compound as a colorless oil. IR (cm^{-1}): 3242, 2974, 2928, 2130, 1738, 1668, 1511. ^1H NMR: (400 MHz, $CDCl_3$) δ 10.81 (brs, 1H), 7.52-7.41 (m, 6H), 7.37-7.25 (m, 8H), 7.22-7.15 (m, 2H), 7.10-7.03 (m, 1H), 6.95 (d, J = 5.6 Hz, 1H), 4.85-4.73 (m, 2H), 4.20 (q, J = 7.2 Hz, 2H), 2.85-2.75 (m, 2H), 2.49 (dd, J = 18.4, 8.4 Hz, 1H), 2.42-2.35 (m, 1H), 2.28-2.20 (m, 1H), 2.19-1.93 (m, 5H), 1.90-1.80 (m, 1H), 1.67-1.46 (m, 5H), 1.44-1.35 (m, 1H), 1.28 (t, J = 6.8 Hz, 3H), 0.91 (s, 3H); ^{13}C NMR: (100 MHz, $CDCl_3$) δ 220.9, 163.5, 140.53, 140.49, 138.8, 137.2, 136.5, 135.1, 135.0, 133.73, 133.70, 129.5, 129.4, 128.3, 127.9, 127.8, 127.6, 127.4, 127.3, 126.7, 125.6, 125.5, 125.3, 124.1, 124.0, 62.1, 62.0, 60.2, 50.4, 47.9, 44.3, 38.0, 35.8, 31.5, 29.4, 26.4, 25.6, 21.5, 21.0, 14.2, 13.8; HRMS (ESI) calculated for $[C_{42}H_{46}N_2O_3SiNa]^+$ $[M+Na]^+$ requires m/z 677.3170, found m/z 677.3168.

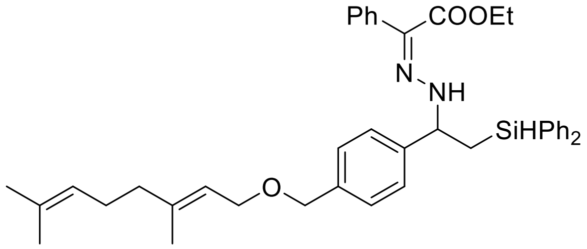

Ethyl (*Z*)-2-(2-(1-(4-((((*E*)-3,7-dimethylocta-2,6-dien-1-yl)oxy)methyl)phenyl)-2-(diphenylsilyl)ethyl)hydrazono)-2-phenylacetate (4ag). Prepared according to the general procedure using 0.0808 g (0.30 mmol) of (*E*)-1-(((3,7-dimethylocta-2,6-dien-1-yl)oxy)methyl)-4-ethynylbenzene, 56 μL (0.99 g/mL, 0.30 mmol) of diphenylsilane, 0.0025 g (0.0031 mmol) of Xantphos•$CoBr_2$, 9 μL (1.0 M in THF, 0.0090 mmol) of $NaBHEt_3$, 1.2 mL (0.25 M) of Et_2O, 0.0020 g (0.0063 mmol) of **L5**, 45 μL (0.88 g/mL, 0.36 mmol) of phenylsilane, 48 μL (1.19 g/mL, 0.30 mmol) of ethyl 2-diazo-2-phenylacetate, and 16 μL (1.00 g/mL, 0.90 mmol) of H_2O. After 12 h, the resulting solution was quenched. The combined filtrate was concentrated and the crude mixture was purified by short flash column chromatography using PE to PE/EtOAc = 50/1 as the eluent to give 0.1415 g (0.22 mmol, 73% yield) of the title compound as a colorless oil. IR (cm^{-1}): 3248, 3050, 2975, 2133, 1733, 1670, 1513. ^{1}H NMR: (400 MHz, $CDCl_3$) δ 10.80 (d, J = 5.2 Hz, 1H), 7.55-7.50 (m, 2H), 7.50-7.42 (m, 4H), 7.38-7.21 (m, 13H), 5.40 (t, J = 6.0 Hz, 1H), 5.10 (t, J = 6.4 Hz, 1H), 4.85-4.75 (m, 2H), 4.46 (s, 2H), 4.21 (q, J = 7.2 Hz, 2H), 4.01 (d, J = 6.4 Hz, 2H), 2.18-2.00 (m, 5H), 1.90-1.80 (m, 1H), 1.67 (s, 3H), 1.64 (s, 3H), 1.60 (s, 3H), 1.28 (t, J = 7.2 Hz, 3H); ^{13}C NMR: (100 MHz, $CDCl_3$) δ 163.4, 142.6, 140.4, 137.7, 137.1, 135.2, 135.0, 133.6, 133.5, 131.6, 129.6, 129.5, 128.3, 128.1, 128.0, 127.9, 127.6, 126.7, 125.6, 124.0, 120.7, 71.6, 66.5, 61.9, 60.2, 39.6, 26.3, 25.7, 20.9, 17.7, 16.5, 14.2; HRMS (ESI) calculated for $[C_{41}H_{48}N_2O_3SiNa]^+$ $[M+Na]^+$ requires m/z 667.3326, found m/z 667.3332.

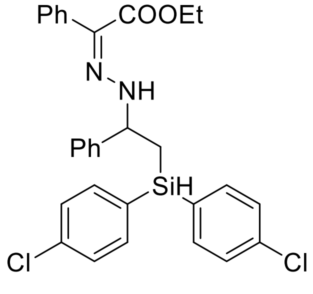

Ethyl (*Z*)-2-(2-(2-(bis(4-chlorophenyl)silyl)-1-phenylethyl)hydrazono)-2-phenylacetate (4ah). Prepared according to the general procedure using 33 μL (0.93 g/mL, 0.30 mmol) of phenylacetylene, 0.0761 g (0.30 mmol) of bis(4-chlorophenyl)silane, 0.0024 g (0.0030 mmol) of Xantphos•$CoBr_2$, 9 μL (1.0 M in THF, 0.0090 mmol) of $NaBHEt_3$, 1.2 mL (0.25 M) of Et_2O, 0.0020 g (0.0063 mmol) of **L5**, 45 μL (0.88 g/mL, 0.36 mmol) of phenylsilane, 48 μL (1.19 g/mL, 0.30 mmol) of ethyl 2-diazo-2-phenylacetate, and 16 μL (1.00 g/mL, 0.90 mmol) of H_2O. After 12 h, the resulting solution was quenched. The combined filtrate was concentrated and the crude mixture was purified by short flash column chromatography using PE to PE/EtOAc = 200/1 as the eluent to give 0.1314 g (0.24 mmol, 80% yield) of the title compound as a colorless oil. IR (cm^{-1}): 3253, 3062, 2931, 2141, 1735, 1688, 1514. ^1H NMR: (400 MHz, $CDCl_3$) δ 10.76 (brs, 1H), 7.47-7.42 (m, 2H), 7.41-7.36 (m, 2H), 7.36-7.30 (m, 2H), 7.30-7.15 (m, 12H), 4.85-4.75 (m, 2H), 4.21 (q, J = 7.2 Hz, 2H), 2.16-2.06 (m, 1H), 1.87-1.77 (m, 1H), 1.28 (t, J = 7.2 Hz, 3H); ^{13}C NMR: (100 MHz, $CDCl_3$) δ 163.4, 143.0, 136.9, 136.4, 136.3, 136.22, 136.16, 131.50, 131.46, 128.7, 128.33, 128.27, 127.64, 127.57, 126.9, 126.6, 125.9, 62.1, 60.4, 20.9, 14.1; HRMS (ESI) calculated for $[C_{30}H_{28}Cl_2N_2O_2SiNa]^+$ $[M+Na]^+$ requires m/z 569.1189, found m/z 569.1192.

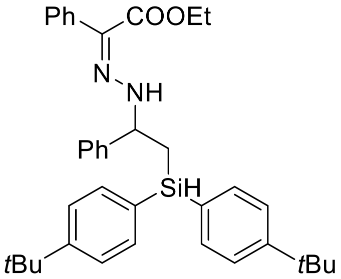

Ethyl (*Z*)-2-(2-(2-(bis(4-(*tert*-butyl)phenyl)silyl)-1-phenylethyl)hydrazono)-2-phenylacetate (4ai). Prepared according to the general procedure using 33 μL (0.93 g/mL, 0.30 mmol) of phenylacetylene, 0.0892 g (0.30 mmol) of bis(4-(*tert*-butyl)phenyl)silane, 0.0024 g (0.0030 mmol) of Xantphos•$CoBr_2$, 9 μL (1.0 M in THF, 0.0090 mmol) of $NaBHEt_3$, 1.2 mL (0.25 M) of Et_2O, 0.0020 g (0.0063 mmol) of **L5**, 45 μL (0.88 g/mL, 0.36 mmol) of phenylsilane, 48 μL (1.19 g/mL, 0.30 mmol) of ethyl 2-diazo-2-phenylacetate, and 16 μL (1.00 g/mL, 0.90 mmol) of H_2O. After 12 h, the resulting solution was quenched. The combined filtrate was concentrated and the crude mixture was purified by short flash column chromatography using PE to PE/EtOAc = 200/1 as the eluent to give 0.1223 g (0.21 mmol, 69% yield) of the title compound as a colorless oil. IR (cm^{-1}):

3250, 3067, 2904, 2130, 1737, 1689, 1514. ^{1}H NMR: (400 MHz, $CDCl_3$) δ 10.87 (d, J = 4.0 Hz, 1H), 7.50-7.39 (m, 6H), 7.37-7.30 (m, 4H), 7.30-7.23 (m, 6H), 7.23-7.17 (m, 2H), 4.85-4.77 (m, 1H), 4.76 (t, J = 3.6 Hz, 1H), 4.22 (q, J = 7.2 Hz, 2H), 2.17-2.07 (m, 1H), 1.88-1.78 (m, 1H), 1.35-1.22 (m, 21H); ^{13}C NMR: (100 MHz, $CDCl_3$) δ 163.5, 152.5, 152.4, 143.5, 137.2, 135.1, 134.9, 130.2, 130.1, 128.5, 128.3, 127.6, 127.3, 126.8, 126.7, 125.5, 125.0, 124.9, 62.2, 60.2, 34.7, 31.2, 21.1, 14.2; HRMS (ESI) calculated for $[C_{38}H_{46}N_2O_2SiNa]^+$ $[M+Na]^+$ requires m/z 613.3221, found m/z 613.3220.

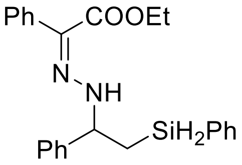

Ethyl (*Z*)-2-phenyl-2-(2-(1-phenyl-2-(phenylsilyl)ethyl)hydrazono)acetate (4aj). Prepared according to the general procedure using 33 μL (0.93 g/mL, 0.30 mmol) of phenylacetylene, 38 μL (0.88 g/mL, 0.30 mmol) of phenylsilane, 0.0023 g (0.0030 mmol) of DPEtphos•$CoBr_2$, 9 μL (1.0 M in THF, 0.0090 mmol) of $NaBHEt_3$, 1.2 mL (0.25 M) of Et_2O, 0.0019 g (0.0060 mmol) of **L5**, 45 μL (0.88 g/mL, 0.36 mmol) of phenylsilane, 0.0760 g (0.30 mmol) of benzyl 2-diazo-2-phenylacetate, and 16 μL (1.00 g/mL, 0.90 mmol) of H_2O. After 12 h, the resulting solution was quenched. The combined filtrate was concentrated and the crude mixture was purified by short flash column chromatography using PE to PE/EtOAc = 50/1 as the eluent to give 0.0666 g (0.17 mmol, 55% yield) of the title compound as a colorless oil. ^{1}H NMR: (400 MHz, $CDCl_3$) δ 10.83 (d, J = 4.8 Hz, 1H), 7.53-7.44 (m, 4H), 7.38-7.20 (m, 11H), 4.86-4.77 (m, 1H), 4.30-4.19 (m, 4H), 1.98-1.88 (m, 1H), 1.72-1.62 (m, 1H), 1.29 (t, J = 7.2 Hz, 3H); ^{13}C NMR: (100 MHz, $CDCl_3$) δ 163.5, 143.0, 137.0, 135.2, 131.6, 129.6, 128.7, 128.3, 128.0, 127.65, 127.55, 126.8, 126.7, 125.9, 62.6, 60.3, 18.8, 14.2; HRMS (ESI) calculated for $[C_{24}H_{26}N_2O_2SiNa]^+$ $[M+Na]^+$ requires m/z 425.1656, found m/z 425.1655.

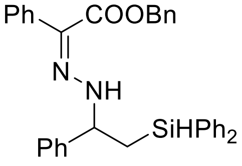

Benzyl (*Z*)-2-(2-(2-(diphenylsilyl)-1-phenylethyl)hydrazono)-2-phenylacetate (4ak). Prepared

according to the general procedure using 33 μL (0.93 g/mL, 0.30 mmol) of phenylacetylene, 56 μL (0.99 g/mL, 0.30 mmol) of diphenylsilane, 0.0024 g (0.0030 mmol) of Xantphos•$CoBr_2$, 9 μL (1.0 M in THF, 0.0090 mmol) of $NaBHEt_3$, 1.2 mL (0.25 M) of Et_2O, 0.0020 g (0.0063 mmol) of **L5**, 45 μL (0.88 g/mL, 0.36 mmol) of phenylsilane, 0.0760 g (0.30 mmol) of benzyl 2-diazo-2-phenylacetate, and 16 μL (1.00 g/mL, 0.90 mmol) of H_2O. After 12 h, the resulting solution was quenched. The combined filtrate was concentrated and the crude mixture was purified by short flash column chromatography using PE to PE/EtOAc = 50/1 as the eluent to give 0.1136 g (0.21 mmol, 70% yield) of the title compound as a colorless oil. IR (cm^{-1}): 3249, 3064, 2926, 2132, 1737, 1670, 1512. ^1H NMR: (400 MHz, $CDCl_3$) δ 10.83 (d, J = 3.6 Hz, 1H), 7.54-7.49 (m, 2H), 7.48-7.43 (m, 4H), 7.35-7.23 (m, 17H), 7.22-7.17 (m, 2H), 5.18 (s, 2H), 4.86-4.7 (m, 2H), 2.18-2.08 (m, 1H), 1.90-1.80 (m, 1H); ^{13}C NMR: (100 MHz, $CDCl_3$) δ 163.0, 143.2, 136.9, 135.7, 135.2, 135.0, 133.6, 133.5, 129.6, 129.63, 128.56, 128.5, 128.3, 128.1, 128.0, 127.93, 127.88, 127.6, 127.4, 126.8, 126.7, 125.2, 65.8, 62.3, 20.9; HRMS (ESI) calculated for $[C_{35}H_{33}N_2O_2Si]^+$ $[M+H]^+$ requires m/z 541.2306, found m/z 541.2308.

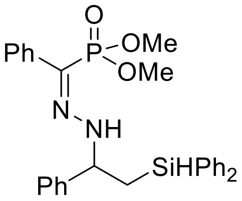

Dimethyl (*Z*)-((2-(2-(diphenylsilyl)-1-phenylethyl)hydrazono)(phenyl)methyl)phosphonate (4al). Prepared according to the general procedure using 33 μL (0.93 g/mL, 0.30 mmol) of phenylacetylene, 56 μL (0.99 g/mL, 0.30 mmol) of diphenylsilane, 0.0025 g (0.0031 mmol) of Xantphos•$CoBr_2$, 9 μL (1.0 M in THF, 0.0090 mmol) of $NaBHEt_3$, 1.2 mL (0.25 M) of Et_2O, 0.0020 g (0.0063 mmol) of **L5**, 45 μL (0.88 g/mL, 0.36 mmol) of phenylsilane, 0.0681g (0.30 mmol) of dimethyl (diazo(phenyl)methyl)phosphonate, and 16 μL (1.00 g/mL, 0.90 mmol) of H_2O. After 12 h, the resulting solution was quenched. The combined filtrate was concentrated and the crude mixture was purified by short flash column chromatography using PE to PE/EtOAc = 10/1 as the eluent to give 0.0942 g (0.18 mmol, 61% yield) of the title compound as a colorless oil. IR (cm^{-1}): 3204, 3065, 2951, 2134, 1596, 1514. ^1H NMR: (400 MHz, $CDCl_3$) δ 10.40-10.31 (m, 1H), 7.56-7.46 (m, 6H), 7.38-7.23 (m, 12H), 7.22-7.16 (m, 2H), 4.81 (t, J = 3.6 Hz, 1H), 4.80-4.72 (m,

1H), 3.64 (d, J = 19.2 Hz, 3H), 3.59 (d, J = 19.2 Hz, 3H), 2.17-2.09 (m, 1H), 1.88-1.78 (m, 1H); ^{13}C NMR: (100 MHz, $CDCl_3$) δ 144.0, 137.4, 137.1, 135.2, 135.0, 133.73, 133.67, 129.6, 129.5, 128.5, 128.1, 128.0, 127.9, 127.2, 126.8, 126.6, 125.69, 125.66, 123.3, 121.8, 61.9, 52.21, 52.17, 20.8; ^{31}P NMR: (162 MHz) δ 14.1; HRMS (ESI) calculated for $[C_{29}H_{32}N_2O_3PSi]^+$ $[M+H]^+$ requires m/z 515.1914, found m/z 515.1916.

IV. Asymmetric Sequential Hydrosilylation/Hydrohydrazidation of Alkynes

General procedure (asymmetric sequential hydrosilylation/hydrohydrazidation of alkynes):

A 25 mL Schlenk flask equipped with a magnetic stirrer and a flanging rubber plug was dried with flame under vacuum. When cooled to ambient temperature (10 - 25 ^{o}C), it was vacuumed and flushed with N_2. This degassed procedure was repeated for three times. To the flame-dried Schlenk flask Xantphos•CoI_2 complex (0.0090 mmol, 3 mol%), 1.2 mL (0.25 M) of distilled EtOAc and diphenylsilane (0.30 mmol, 1.0 equiv.) were added by dropwise sequentially. After that, $LiBHEt_3$ (27 μL, 1.0 M in THF, 0.0270 mmol， 9 mol%) and alkyne (0.30 mmol, 1.0 equiv.) were added to the mixture sequentially and stirred for 5 s at ambient temperature. Later, EtOAc was carefully swabbed-off *in vacuo* and the flask was backfilled with N_2. MeCN and **L12** (0.0180 mmol, 6 mol%) were added at ambient temperature in sequnece. Then phenylsilane (0.36 mmol, 1.2 equiv.), phenyldiazoacetate (0.30 mmol, 1.0 equiv.), and H_2O (0.90 mmol, 3.0 equiv.) were added by dropwise sequentially at 0 ^{o}C. Pinholes are sealed with silicone grease and the flanging rubber plug was wrapped with sealing film tightly. The mixture was stirred at 0 ^{o}C for 24 h, and then quenched by 5 mL of PE/EtOAc (5/1) and stirred for 5 mins until catalyst precipitated. The resulting solution was filtered through a pad of silica gel and washed using PE/EtOAc (5/1) (15 mL × 3). The combined filtrates were concentrated *in vacuo*. NMR yield was monitored by ^{1}H NMR analysis using TMSPh as internal standard. The crude mixture was purified by short flash column chromatography to give the corresponding product.

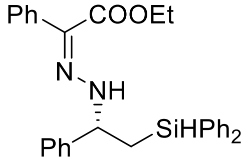

Ethyl (*S*,*Z*)-2-(2-(2-(diphenylsilyl)-1-phenylethyl)hydrazono)-2-phenylacetate (4a'). Prepared according to the general procedure using 33 μL (0.93 g/mL, 0.30 mmol) of phenylacetylene, 56 μL (0.99 g/mL, 0.30 mmol) of diphenylsilane, 0.0081 g (0.0090 mmol) of Xantphos•CoI_2, 27 μL (1.0 M in THF, 0.0270 mmol) of LiBHEt_3, 1.2 mL (0.25 M) of EA, 1.2 mL (0.25 M) of MeCN, 0.0087 g (0.0180 mmol) of **L12**, 45 μL (0.88 g/mL, 0.36 mmol) of phenylsilane, 48 μL (1.19 g/mL, 0.30 mmol) of ethyl 2-diazo-2-phenylacetate, and 16 μL (1.00 g/mL, 0.90 mmol) of H_2O. After 24 h, the resulting solution was quenched. The combined filtrate was concentrated and the crude mixture was purified by short flash column chromatography using PE to PE/EtOAc = 100/1 as the eluent to give 0.0752 g (0.16 mmol, 52% yield) of the title compound as a colorless oil. Optical Rotation: $[\alpha]_{20}^{D}$ = -2.0 (c 1.34, $CHCl_3$), 86.6% *ee* was determined by HPLC, HPLC conditions: Chiralcel OD-H, *n*-hexane/*i*PrOH = 95/5, 1.0 mL/min, n = 220 nm, tr 4.8 (major), 6.0 (minor). ^{1}H NMR: (400 MHz, $CDCl_3$) δ 10.82 (d, *J* = 4.4 Hz, 1H), 7.52 (d, *J* = 6.8 Hz, 2H), 7.50-7.43 (m, 4H), 7.38-7.25 (m, 12H), 7.25-7.18 (m, 2H), 4.86-4.76 (m, 2H), 4.21 (q, *J* = 7.2 Hz, 2H), 2.20-2.09 (m, 1H), 1.91-1.80 (m, 1H), 1.28 (t, *J* = 7.2 Hz, 3H); ^{13}C NMR: (100 MHz, $CDCl_3$) δ 163.5, 143.4, 137.1, 135.2, 135.0, 133.63, 133.55, 129.63, 129.57, 128.6, 128.3, 128.0, 127.9, 127.6, 127.4, 126.7, 125.6, 62.2, 60.3, 21.0, 14.2.

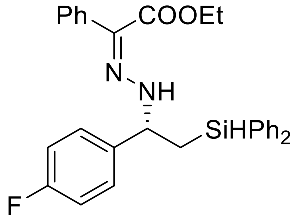

Ethyl (*S*,*Z*)-2-(2-(2-(diphenylsilyl)-1-(4-fluorophenyl)ethyl)hydrazono)-2-phenylacetate (4i'). Prepared according to the general procedure using 0.0394 g (0.30 mmol) of 1-ethynyl-4-fluorobenzene, 56 μL (0.99 g/mL, 0.30 mmol) of diphenylsilane, 0.0081 g (0.0090 mmol) of Xantphos•CoI_2, 27 μL (1.0 M in THF, 0.0270 mmol) of LiBHEt_3, 1.2 mL (0.25 M) of EA, 1.2 mL (0.25 M) of MeCN, 0.0087 g (0.0180 mmol) of **L12**, 45 μL (0.88 g/mL, 0.36 mmol) of phenylsilane, 48 μL (1.19 g/mL, 0.30 mmol) of ethyl 2-diazo-2-phenylacetate, and 16 μL (1.00 g/mL, 0.90 mmol) of H_2O. After 24 h, the resulting solution was quenched. The combined filtrate was concentrated and the crude mixture was purified by short flash column chromatography using PE to PE/EtOAc = 100/1 as the eluent to give 0.0669 g (0.13 mmol, 44% yield) of the title compound as a pale yellow oil. Optical Rotation: $[\alpha]_{20}^{D}$ = -4.5 (c 1.40, $CHCl_3$), 83.8% *ee* was

determined by HPLC, HPLC conditions: Chiralcel OD-H, *n*-hexane/*i*PrOH = 95/5, 1.0 mL/min, n = 220 nm, tr 5.0 (major), 7.3 (minor). ^{1}H NMR: (400 MHz, $CDCl_3$) δ 10.76 (d, J = 4.8 Hz, 1H), 7.54-7.49 (m, 2H), 7.48-7.42 (m, 4H), 7.38-7.27 (m, 8H), 7.26-7.17 (m, 3H), 6.97-6.90 (m, 2H), 4.83-4.72 (m, 2H), 4.22 (q, J = 7.2 Hz, 2H), 2.18-2.07 (m, 1H), 1.88-1.78 (m, 1H), 1.29 (t, J = 7.2 Hz, 3H); ^{13}C NMR: (100 MHz, $CDCl_3$) δ 163.4, 162.0 (d, J = 244.2 Hz, 1C), 139.0 (d, J = 2.9 Hz, 1C), 137.0, 135.1, 135.0, 133.4, 133.3, 129.7, 129.6, 128.4 (d, J = 8.4 Hz, 1C), 128.3, 128.03, 127.97, 127.7, 126.9, 126.0, 115.3 (d, J = 21.1 Hz, 1C), 61.4, 60.3, 20.9, 14.2. ^{19}F NMR: (376 MHz) δ -115.2;

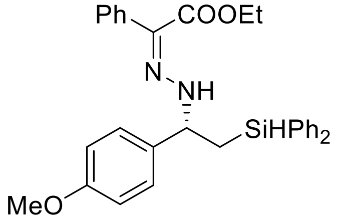

Ethyl (*S*,*Z*)-2-(2-(2-(diphenylsilyl)-1-(4-methoxyphenyl)ethyl)hydrazono)-2-phenylacetate (4n'). Prepared according to the general procedure using 0.0394 g (0.30 mmol) of 1-ethynyl-4-methoxybenzene, 56 μL (0.99 g/mL, 0.30 mmol) of diphenylsilane, 0.0081 g (0.0090 mmol) of Xantphos•CoI_2, 27 μL (1.0 M in THF, 0.0270 mmol) of $LiBHEt_3$, 1.2 mL (0.25 M) of EA, 1.2 mL (0.25 M) of MeCN, 0.0087 g (0.0180 mmol) of **L12**, 45 μL (0.88 g/mL, 0.36 mmol) of phenylsilane, 48 μL (1.19 g/mL, 0.30 mmol) of ethyl 2-diazo-2-phenylacetate, and 16 μL (1.00 g/mL, 0.90 mmol) of H_2O. After 24 h, the resulting solution was quenched. The combined filtrate was concentrated and the crude mixture was purified by short flash column chromatography using PE to PE/EtOAc = 100/1 as the eluent to give 0.0669 g (0.13 mmol, 44% yield) of the title compound as a colorless oil. Optical Rotation: $[\alpha]_{20}^{D}$ = -2.0 (c 1.04, $CHCl_3$), 83.7% *ee* was determined by HPLC, HPLC conditions: Chiralcel OD-H, *n*-hexane/*i*PrOH = 95/5, 1.0 mL/min, n = 220 nm, tr 6.0 (major), 7.6 (minor). ^{1}H NMR: (400 MHz, $CDCl_3$) δ 10.77 (d, J = 4.8 Hz, 1H), 7.55-7.50 (m, 2H), 7.29-7.42 (m, 4H), 7.38-7.26 (m, 8H), 7.25-7.22 (m, 1H), 7.18 (d, J = 8.4 Hz, 2H), 6.80 (d, J = 8.8 Hz, 2H), 4.80-4.72 (m, 2H), 4.21 (q, J = 7.2 Hz, 2H), 3.77 (s, 3H), 2.20-2.10 (m, 1H), 1.88-1.80 (m, 1H), 1.28 (t, J = 7.2 Hz, 3H); ^{13}C NMR: (100 MHz, $CDCl_3$) δ 163.5, 158.9, 137.2, 135.2, 135.0, 133.7, 133.6, 129.6, 129.5, 128.3, 128.0, 127.9, 127.6, 126.7, 125.4, 113.9, 61.6, 60.2, 55.2, 20.9, 14.2.

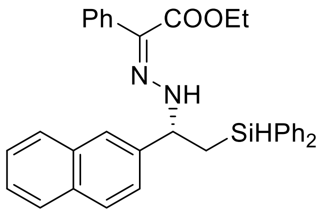

Ethyl (*S*,*Z*)-2-(2-(2-(diphenylsilyl)-1-(naphthalen-2-yl)ethyl)hydrazono)-2-phenylacetate (4w'). Prepared according to the general procedure using 0.0456 g (0.30 mmol) of 2-ethynylnaphthalene, 56 μL (0.99 g/mL, 0.30 mmol) of diphenylsilane, 0.0081 g (0.0090 mmol) of Xantphos•CoI_2, 27 μL (1.0 M in THF, 0.0270 mmol) of $LiBHEt_3$, 1.2 mL (0.25 M) of EA, 1.2 mL (0.25 M) of MeCN, 0.0087 g (0.0180 mmol) of **L12**, 45 μL (0.88 g/mL, 0.36 mmol) of phenylsilane, 48 μL (1.19 g/mL, 0.30 mmol) of ethyl 2-diazo-2-phenylacetate, and 16 μL (1.00 g/mL, 0.90 mmol) of H_2O. After 24 h, the resulting solution was quenched. The combined filtrate was concentrated and the crude mixture was purified by short flash column chromatography using PE to PE/EtOAc = 100/1 as the eluent to give 0.0806 g (0.15 mmol, 51% yield) of the title compound as a pale yellow oil. Optical Rotation: $[\alpha]_{20}^{D}$ = +12.7 (c 1.40, $CHCl_3$), 83.8% *ee* was determined by HPLC, HPLC conditions: Chiralcel OD-H, *n*-hexane/*i*PrOH = 95/5, 1.0 mL/min, n = 220 nm, tr 5.9 (major), 8.2 (minor). ^{1}H NMR: (400 MHz, $CDCl_3$) δ 10.91 (s, 1H), 7.82-7.70 (m, 3H), 7.63 (s, 1H), 7.55-7.50 (m, 2H), 7.49-7.40 (m, 7H), 7.37-7.20 (m, 9H), 5.03-4.93 (m, 1H), 4.79 (t, *J* = 8.0 Hz, 1H), 4.22 (q, *J* = 7.2 Hz, 2H), 2.28-2.18 (m, 1H), 2.00-1.90 (m, 1H), 1.28 (t, *J* = 7.2 Hz, 3H); ^{13}C NMR: (100 MHz, $CDCl_3$) δ 163.5, 140.6, 137.1, 135.2, 135.0, 133.55, 133.50, 133.2, 132.8, 129.7, 129.5, 128.5, 128.3, 128.0, 127.95, 127.90, 127.63, 127.58, 126.8, 126.0, 125.82, 125.77, 125.6, 124.8, 62.3, 60.3, 20.7, 14.2.

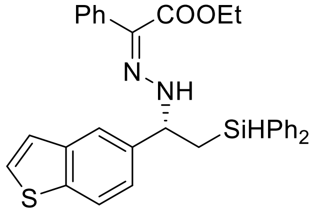

Ethyl(*S*,*Z*)-2-(2-(1-(benzo[*b*]thiophen-5-yl)-2-(diphenylsilyl)ethyl)hydrazono)-2-phenylacetate (4aa'). Prepared according to the general procedure using 0.0473 g (0.30 mmol) of 5-ethynylbenzo[*b*]thiophene, 56 μL (0.99 g/mL, 0.30 mmol) of diphenylsilane, 0.0081 g (0.0090 mmol) of Xantphos•CoI_2, 27 μL (1.0 M in THF, 0.0270 mmol) of $LiBHEt_3$, 1.2 mL (0.25 M) of EA, 1.2 mL (0.25 M) of MeCN, 0.0087 g (0.0180 mmol) of **L12**, 45 μL (0.88 g/mL, 0.36 mmol)

of phenylsilane, 48 μL (1.19 g/mL, 0.30 mmol) of ethyl 2-diazo-2-phenylacetate, and 16 μL (1.00 g/mL, 0.90 mmol) of H_2O. After 24 h, the resulting solution was quenched. The combined filtrate was concentrated and the crude mixture was purified by short flash column chromatography using PE to PE/EtOAc = 100/1 as the eluent to give 0.0844 g (0.16 mmol, 53% yield) of the title compound as a pale yellow oil. Optical Rotation: $[\alpha]_{20}^{D}$ = -1.3 (c 1.12, $CHCl_3$), 82.5% *ee* was determined by HPLC, HPLC conditions: Chiralcel OD-H, *n*-hexane/*i*PrOH = 95/5, 1.0 mL/min, n = 220 nm, tr 6.4 (major), 9.7 (minor). ^{1}H NMR: (400 MHz, $CDCl_3$) δ 10.87 (s, 1H), 7.76 (d, *J* = 8.4 Hz, 1H), 7.66 (d, *J* = 1.2 Hz, 1H), 7.55-7.50 (m, 2H), 7.49-7.41 (m, 4H), 7.40 (d, *J* = 5.6 Hz, 1H), 7.35-7.20 (m, 11H), 4.98-4.88 (m, 1H), 4.79 (t, *J* = 7.8 Hz, 1H), 4.21 (q, *J* = 7.2 Hz, 2H), 2.26-2.16 (m, 1H), 1.97-1.87 (m, 1H), 1.28 (t, *J* = 7.2 Hz, 3H); ^{13}C NMR: (100 MHz, $CDCl_3$) δ 163.5, 139.7, 139.5, 138.8, 137.1, 135.2, 135.0, 133.5, 129.6, 129.5, 128.3, 128.0, 127.9, 127.6, 126.8, 125.7, 123.9, 123.3, 122.7, 121.7, 62.3, 60.3, 21.2, 14.2;

V. Gram-Scale Reaction

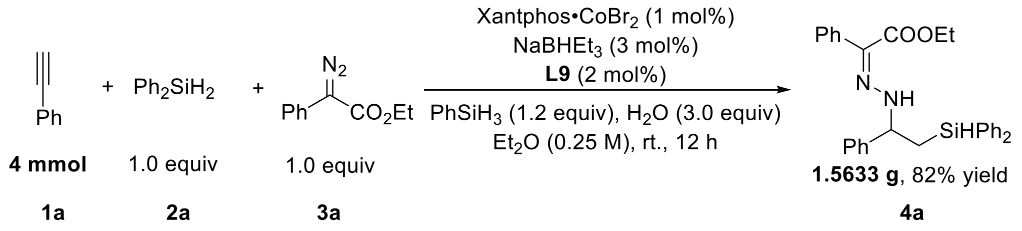

Supplementary Figure 1. Gram-Scale Reaction.

A 50 mL Schlenk flask equipped with a magnetic stirrer and a flanging rubber plug was dried with flame under vacuum. When cooled to ambient temperature, it was vacuumed and flushed with N_2. This degassed procedure was repeated for three times. To the flame-dried Schlenk flask 0.0320 g (0.040 mmol, 1 mol%) of Xantphos•$CoBr_2$ complex, 16 mL (0.25 M) of Et_2O and 747 μL (0.99 g/mL, 4 mmol, 1.0 equiv.) of diphenylsilane were added by dropwise sequentially. After that, 120 μL (1.0 M in THF, 0.120 mmol, 3 mol%) of $NaBHEt_3$ and 440 μL (0.93 g/mL, 4 mmol, 1.0 equiv.) of phenylacetylene were added to the mixture sequentially and slowly, stirred for 2 mins and cool to ambient temperature for 2 mins, and then 0.0254 g (0.08 mmol, 2 mol%) of **L5**, 600 μL (0.88

g/mL, 4.8 mmol, 1.2 equiv.) of phenylsilane, 640 μL (1.19 g/mL, 4 mmol, 1.0 equiv.) of ethyl 2-diazo-2-phenylacetate, and 216 μL (1.00 g/mL, 12 mmol) of H_2O were added by dropwise sequentially. Pinholes were sealed with silicone grease and the flanging rubber plug was wrapped with sealing film tightly. The mixture was stirred at ambient temperature for 12 h, and quenched by 20 mL PE, stirring for 5 mins. The resulting solution was filtered through a pad of silica gel and the Schlenk flask and silica gel were washed by PE/EtOAc (5/1) (50 mL × 3). The combined filtrates were concentrated *in vacuo*. The residue was purified by short flash column chromatography using PE PE/EtOAc = 100/1 as the eluent to give 1.5633 g (3.28 mmol, 82% yield) of the title compound as a colorless oil.

VI. Further Derivatizations

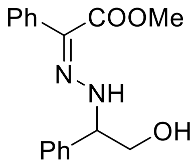

Methyl (*Z*)-2-(2-(2-hydroxy-1-phenylethyl)hydrazono)-2-phenylacetate (6). Prepared according to the previously reported procedure.[16] To a 25 mL Schlenk flask equipped with magnetic stir bar, 2.0 mL of THF/MeOH = 1/1 (*v/v*), 0.0465 g (0.8 mmol, 4.0 equiv.) of KF, 0.0805 g (0.8 mmol, 4.0 equiv.) of $KHCO_3$, 0.1082 g of **4al** (0.20 mmol) in 2.0 mL of THF/MeOH = 1/1 (*v/v*) were added in sequence, and 0.51 mL (1.11 g/mL, 5.0 mmol, 25.0 equiv.) of H_2O_2 was added dropwise at ambient temperature. After 12 h, the reaction was quenched by addition of H_2O (2.0 mL), and the organic layer was separated. The aqueous layer was extracted with EtOAc (3 × 20 mL). The combined organic layers were washed with brine, dried over Na_2SO_4 and concentrated *in vacuo* to give a crude oil. The residue was purified by short flash column chromatography using PE to PE/EtOAc = 5/1 as the eluent to give 0.0525 g (0.18 mmol, 88% yield) of the title compound as a colorless oil. IR (cm^{-1}): 3486, 2924, 1737, 1687, 1595, 1512. ^1H NMR: (400 MHz, $CDCl_3$) δ 10.87 (d, J = 2.8 Hz, 1H), 7.54-7.45 (m, 2H), 7.40-7.26 (m, 8H), 4.84-4.76 (m, 1H), 4.18-4.08 (m, 1H), 3.99-3.90 (m, 1H), 3.79 (s, 3H), 2.78-2.70 (m, 1H); ^{13}C NMR: (100 MHz, $CDCl_3$) δ 163.9, 138.5, 136.6, 128.8, 128.3, 128.1, 127.9, 127.3, 127.2, 127.1, 66.7, 65.8, 51.4; HRMS (ESI) calculated for $[C_{17}H_{18}N_2O_3Na]^+$ $[M+Na]^+$ requires m/z 321.1210,

found m/z 321.1210.

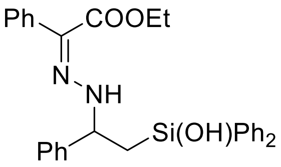

Ethyl (*Z*)-2-(2-(2-(hydroxydiphenylsilyl)-1-phenylethyl)hydrazono)-2-phenylacetate (7). Prepared according to the previously reported procedure.[7] To a 50 mL flamed-dried Schlenk flask equipped with magnetic stir bar, 4.0 mL of THF/MeOH = 1/1 (*v/v*), 0.0304 g (0.30 mmol, 1.0 equiv.) of $KHCO_3$, 0.1441 g (0.30 mmol, 1.0 equiv.) of **4a** in 2.0 mL of THF/MeOH = 1/1 (*v/v*) were added in sequence, and 0.6 mL (1.11 g/mL, 5.4 mmol, 18.0 equiv.) of H_2O_2 was added dropwise at ambient temperature. After 12 h, the reaction was quenched by addition of H_2O (2.0 mL), and the organic layer was separated. The aqueous layer was extracted with EtOAc (3 × 20 mL). The combined organic layers were washed with brine, dried over Na_2SO_4 and concentrated *in vacuo* to give a crude oil. The residue was purified by short flash column chromatography using PE to PE/EtOAc = 10/1 as the eluent to give 0.1024 g (0.21 mmol, 69% yield) of the title compound as a colorless oil. IR (cm^{-1}): 3257, 3065, 2984, 1671, 1595, 1514. ^{1}H NMR: (400 MHz, $CDCl_3$) δ 10.72 (d, J = 4.8 Hz, 1H), 7.58-7.50 (m, 4H), 7.45-7.40 (m, 2H), 7.39-7.23 (m, 14H), 4.95-4.86 (m, 1H), 4.25-4.12 (m, 2H), 2.74 (s, 1H), 2.09 (dd, J = 15.2, 9.6 Hz, 1H), 1.82 (dd, J = 15.2, 6.0 Hz, 1H), 1.26 (t, J = 7.2 Hz, 3H); ^{13}C NMR: (100 MHz, $CDCl_3$) δ 163.4, 143.6, 136.7, 135.9, 135.8, 134.1, 134.0, 129.8, 129.7, 128.8, 128.3, 127.9, 127.82, 127.77, 127.6, 127.1, 126.6, 126.4, 61.4, 60.4, 24.2, 14.1; HRMS (ESI) calculated for $[C_{30}H_{30}N_2O_3SiNa]^+$ $[M+Na]^+$ requires m/z 517.1918, found m/z 517.1921.

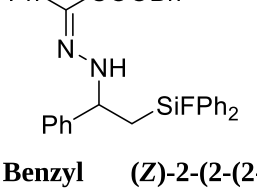

Benzyl (*Z*)-2-(2-(2-(fluorodiphenylsilyl)-1-phenylethyl)hydrazono)-2-phenylacetate (8). Prepared according to a previously reported procedure.[7] A 25 mL Schlenk flask equipped with a magnetic stirrer and a flanging rubber plug was dried with flame under vacuum. When cooled to ambient temperature, it was vacuumed and flushed with N_2. This degassing procedure was

repeated for three times. To the flame-dried Schlenk flask 0.1076 g (0.8 mmol, 4.0 equiv.) of CuCl, 0.0068 g (0.036 mmol, 18 mol%) of CuI, 0.0284 g (0.48 mmol, 2.4 equiv.) of KF, **4al** (0.1082 g, 0.20 mmol) in 2.0 mL of THF were added in sequence. The mixture was stirred at ambient temperature for 12 h, and quenched by 5 mL PE. The resulting solution was filtered through a pad of silica gel washed by PE/EtOAc (5/1) (15 mL × 3). The combined filtrates were concentrated *in vacuo* to give 0.1028 g (0.18 mmol, 92% yield) of the title compound as a colorless oil. IR (cm^{-1}): 3250, 2953, 1735, 1672, 1593, 1514. ^{1}H NMR: (400 MHz, $CDCl_3$) δ 10.76 (d, J = 5.2 Hz, 1H), 7.56-7.47 (m, 4H), 7.45-7.35 (m, 5H), 7.34-7.26 (m, 11H), 7.25-7.24 (m, 2H), 7.23-7.17 (m, 3H), 5.17 (s, 2H), 4.95-4.88 (m, 1H), 2.31-2.20 (m, 1H), 1.98-1.88 (m, 1H); ^{13}C NMR: (100 MHz, $CDCl_3$) δ 162.9, 143.1, 136.9, 135.7, 134.08, 134.06, 134.0, 132.9, 132.7, 132.6, 130.53 (d, J = 2.1 Hz, 1C), 128.6, 128.5, 128.3, 128.1, 128.0, 127.9, 127.7, 127.6, 127.5, 126.8, 126.6, 125.4, 65.8, 60.9, 22.7 (d, J = 14.6 Hz, 1C); ^{19}F NMR: (376 MHz, $CDCl_3$) δ -169.8; HRMS (ESI) calculated for $[C_{35}H_{31}FN_2O_2SiNa]^+$ $[M+Na]^+$ requires m/z 581.2031, found m/z 581.2035.

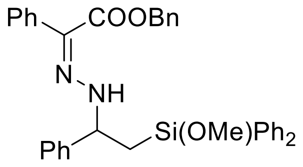

Benzyl (*Z*)-2-(2-(2-(methoxydiphenylsilyl)-1-phenylethyl)hydrazono)-2-phenylacetate (9). Prepared according to a previously reported procedure.[7] A 25 mL Schlenk flask equipped with a magnetic stirrer and a flanging rubber plug was dried with flame under vacuum. When cooled to ambient temperature, it was vacuumed and flushed with N_2. This degassing procedure was repeated for three times. To the flame-dried Schlenk flask 0.0108 g (10 wt%) of Pd/C (5% Pd on C, wetted with ca. 55% water), 0.1081 g of **4al** (0.20 mmol) in 2.5 mL of MeOH/THF = 4/1 (*v/v*) were added in sequence. The mixture was stirred at ambient temperature for 12 h, and quenched by 5 mL PE. The resulting solution was filtered through a pad of silica gel washed by PE/EtOAc (5/1) (15 mL × 3). The combined filtrates were concentrated *in vacuo.* The residue was purified by short flash column chromatography using PE/EtOAc = 50/1 as the eluent to give 0.0856 g (0.15 mmol, 75% yield) of the title compound as a colorless oil. IR (cm^{-1}): 3458, 2956, 2926, 1738, 1690, 1596. ^{1}H NMR: (400 MHz, $CDCl_3$) δ 10.77 (d, J = 4.8 Hz, 1H), 7.56-7.47 (m, 4H),

7.45-7.40 (m, 2H), 7.38-7.26 (m, 11H), 7.25-7.15 (m, 8H), 5.23-5.13 (m, 2H), 4.91-4.83 (m, 1H), 3.37 (s, 3H), 2.18-2.08 (m, 1H), 1.90-1.81 (m, 1H); ^{13}C NMR: (100 MHz, $CDCl_3$) δ 162.9, 144.0, 137.0, 135.8, 134.7, 134.6, 134.0, 133.9, 129.88, 129.85, 128.50, 128.45, 128.3, 128.1, 127.9, 127.8, 127.6, 127.2, 126.7, 124.9, 65.7, 61.3, 51.2, 22.0; HRMS (ESI) calculated for $[C_{36}H_{34}N_2O_3SiNa]^+$ $[M+Na]^+$ requires m/z 593.2231, found m/z 593.2227.

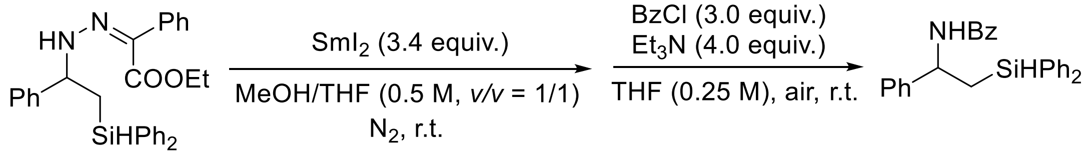

Supplementary Figure 2. Cleavage of *N*-*N* bond and protection of amine.

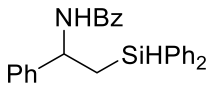

***N*-(2-(diphenylsilyl)-1-phenylethyl)benzamide (10).** A 50 mL Schlenk flask equipped with a magnetic stirrer and a flanging rubber plug was dried with flame under vacuum. When cooled to ambient temperature, it was vacuumed and flushed with N_2. This degassing procedure was repeated for three times. To the flame-dried Schlenk flask, 0.3524 g (0.74 mmol, 1.0 equiv.) of **4a**, 1.4 mL (0.5 M) of MeOH/THF = 1/1 (*v/v*) were added in sequence. Then the tube was degassed with N_2 three times and 25 mL of SmI_2 solution (0.1 M in THF, 2.5 mmol, 3.4 equiv.) was added dropwise at room temperature until the mixture turned dark blue (extra SmI_2 solution could be added if the mixture did not turn blue). The mixture was stirred at ambient temperature until the blue faded and the resulting solution was concentrated *in vacuo.* Then 3 mL (0.25 M) of anhydrous THF was added into the residue and 258 μL (1.21 g/ mL, 2.21 mmol, 3.0 equiv.) of BzCl, 405 μL (0.73 g/ mL, 2.94 mmol, 4.0 equiv.) were slowly dropped into the mixture in sequence. The mixture was stirred at ambient temperature for 12 h, and quenched by 5 mL EtOAc. The resulting solution was filtered through a pad of silica gel washed by EtOAc (20 mL × 3) and he combined filtrates were concentrated *in vacuo.* The operation of filtration and concentration was repeated again. Then 50 mL H_2O and 50 mL EtOAc were added into the mixture and the organic layer was separated. The aqueous layer was extracted with EtOAc (3 × 50 mL). The combined organic layers were washed with brine, dried over Na_2SO_4 and concentrated *in vacuo* to

give a crude oil. The residue was purified by short flash column chromatography using PE to PE/EtOAc = 5/1 as the eluent to give 0.1447 g (0.36 mmol, 48% yield) of the title compound as a white solid. M.p. 147.1-148.2 °C. IR (cm^{-1}): 3316, 3066, 2134, 1635, 1532, 1488. ^{1}H NMR: (400 MHz, $CDCl_3$) δ 7.58 (d, J = 6.8 Hz, 2H), 7.50 (d, J = 6.8 Hz, 2H), 7.45-7.28 (m, 15H), 7.25-7.20 (m, 1H), 6.31 (d, J = 6.8 Hz, 1H), 5.43 (q, J = 7.6 Hz, 1H), 4.81 (t, J = 3.6 Hz, 1H), 2.08-1.97 (m, 1H), 1.92-1.82 (m, 1H); ^{13}C NMR: (100 MHz, $CDCl_3$) δ 166.1, 143.6, 135.2, 135.0, 134.2, 133.7, 133.4, 131.3, 129.9, 129.8, 128.7, 128.4, 128.35, 128.32, 128.2, 127.4, 126.8, 126.2, 51.6, 21.6; HRMS (ESI) calculated for $[C_{27}H_{25}NOSiNa]^+$ $[M+Na]^+$ requires m/z 430.1598, found m/z 430.1596.

VII. Mechanistic Studies and Control Experiments

A) Radical Trapping Experiment

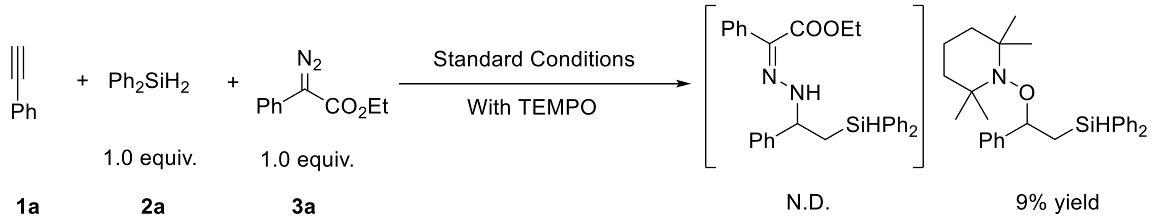

Supplementary Figure 3. Radical Trapping Experiment.

The experiment was conducted according to the general procedure using 33 μL (0.93 g/mL, 0.30 mmol) of phenylacetylene, 56 μL (0.99 g/mL, 0.30 mmol) of diphenylsilane, 0.0024 g (0.0030 mmol) of Xantphos•$CoBr_2$, 9 μL (1.0 M in THF, 0.0090 mmol) of $NaBHEt_3$, 2.4 mL (0.125 M) of Et_2O, 0.0020 g (0.0063 mmol) of **L5**, 45 μL (0.88 g/mL, 0.36 mmol) of phenylsilane, 48 μL (1.19 g/mL, 0.30 mmol) of ethyl 2-diazo-2-phenylacetate, 16 μL (1.00 g/mL, 0.90 mmol) of H_2O, and finally adding 0.0940g (0.60 mmol) of TEMPO. After 12 h, the resulting solution was quenched. The combined filtrate was concentrated in *vacuo* and no desired product was observed by ^{1}H NMR and 0.0121g (0.03 mmol, 9%) of the radical trapped product was obtain as a colorless liquid (the residue was purified by short flash column chromatography using PE to PE/EtOAc = 20/1 as the eluent). IR (cm^{-1}): 3066, 2968, 2138, 1457, 1429. ^{1}H NMR: (400 MHz) δ 7.50-7.44 (m, 2H), 7.42-7.28 (m, 6H), 7.27-7.14 (m, 7H), 4.69 (dd, J = 8.0, 3.2 Hz, 1H), 4.31 (dd, J = 6.0, 2.4 Hz, 1H), 2.25 (ddd, J = 14.0, 5.6, 3.6 Hz, 1H), 1.81 (ddd, J = 14.0, 14.0, 2.0 Hz, 1H), 1.47-1.38 (m, 2H), 1.34-1.07 (m, 10H), 0.96 (s, 3H), 0.41 (s, 3H); ^{13}C NMR: (100 MHz) δ 143.8, 135.2, 134.8,

134.3, 133.9, 129.5, 129.3, 127.9, 127.8, 127.7, 127.2, 85.0, 60.0, 59.1, 40.4, 40.2, 34.4, 34.2, 21.9, 20.3, 17.2; HRMS (ESI) calculated for $[C_{29}H_{38}NOSi]^+$ $[M+H]^+$ requires m/z 444.2717, found m/z 444.2718.

B) Isotopic Labeling Experiment

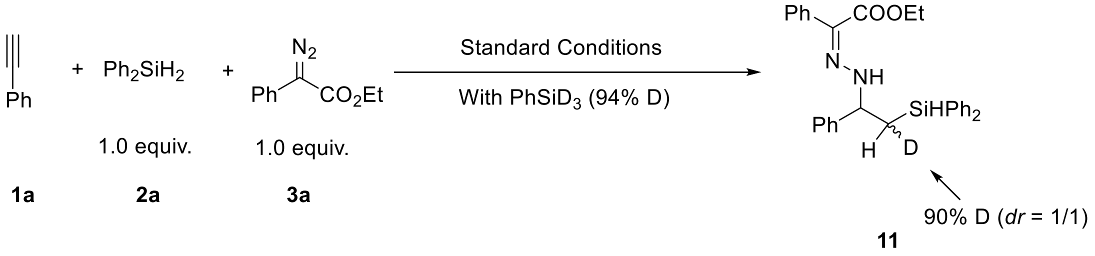

Supplementary Figure 4. Isotopic Labeling Experiment.

The experiment was conducted according to the general procedure using 33 μL (0.93 g/mL, 0.30 mmol) of phenylacetylene, 56 μL (0.99 g/mL, 0.30 mmol) of diphenylsilane, 0.0024 g (0.0030 mmol) of Xantphos•$CoBr_2$, 9 μL (1.0 M in THF, 0.0090 mmol) of $NaBHEt_3$, 1.2 mL (0.25 M) of Et_2O, 0.0019 g (0.0060 mmol) of **L5**, 0.0400 g (0.36 mmol, 94% D) of $PhSiD_3$, 48 μL (1.19 g/mL, 0.30 mmol) of ethyl 2-diazo-2-phenylacetate, and 16 μL (1.00 g/mL, 0.90 mmol) of H_2O. After 12 h, the resulting solution was quenched. The combined filtrate was concentrated and the crude mixture was purified by short flash column chromatography using PE to PE/EtOAc = 100/1 as the eluent to give 0.1166 g (0.24 mmol, 81% yield) of the title compound as a colorless oil. IR (cm^{-1}): 3249, 3060, 2988, 2134, 1735, 1682, 1510. ^{1}H NMR: (400 MHz, $CDCl_3$) δ 10.84 (d, J = 4.8 Hz, 1H), 7.55-7.50 (m, 2H), 7.49-7.43 (m, 4H), 7.40-7.25 (m, 11H), 7.26-7.17 (m, 3H), 4.85-4.78 (m, 2H), 4.20 (q, J = 7.2 Hz, 2H), 2.19-2.08 (m, 0.55H), 1.90-1.80 (m, 0.55H), 1.27 (t, J = 7.2 Hz, 3H); ^{13}C NMR: (100 MHz, $CDCl_3$) δ 163.4, 143.33, 143.31, 137.1, 135.2, 135.0, 133.6, 133.5, 129.6, 129.5, 128.6, 128.3, 128.0, 127.9, 127.6, 127.4, 126.72, 126.68, 125.6, 62.1, 60.2, 21.0-20.4 (m, 1C) 14.2; ^{2}H NMR: (77 MHz, $CHCl_3$) δ 2.21 (s, 0.45D), 1.92 (s, 0.45D); HRMS (ESI) calculated for $[C_{30}H_{30}DN_2O_2Si]^+$ $[M+H]^+$ requires m/z 480.2212, found m/z 480.2210.

C) Time course study

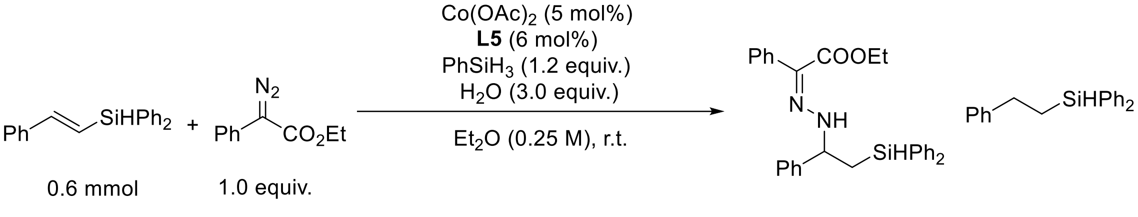

Supplementary Figure 5. Time Course Study.

A 25 mL Schlenk flask equipped with a magnetic stirrer and a flanging rubber plug was dried with flame under vacuum. When cooled to ambient temperature, it was vacuumed and flushed with N_2. This degassing procedure was repeated for three times. Then 0.0053 g (0.030 mmol) of $Co(OAc)_2$, 0.0114 g (0.036 mmol) of **L5** and Et_2O (2.4 mL, 0.25M) were added sequentially. The mixture was stirred for 30 mins. Then 0.1717 g (0.6 mmol, 1.0 equiv.) of (*E*)-diphenyl(styryl)silane, 90 μL (0.88 g/mL, 0.72 mmol, 1.2 equiv.) of $PhSiH_3$, 96 μL (1.19 g/mL, 0.6 mmol, 1.0 equiv.) of ethyl 2-diazo-2-phenylacetate and 32 μL (1.00 g/mL, 1.8 mmol, 3.0 equiv.) of H_2O were added sequentially. 50 μL of the reaction mixture was taken out at 0.5 h, 1 h, 1.5 h, 2 h, 3 h, 4 h, 5 h, 6 h, 8 h, 10 h, 12 h and quenched with 2 mL PE immediately followed by concentration *in vacuo*. 5 μL of dilute trimethylphenylsilane standard solution (prepared using 10 μL trimethylphenylsilane and 230 μL $CDCl_3$) and 0.5 mL $CDCl_3$ were added to the residue as internal standard. Yields were monitored by ^1H NMR analysis.

Supplementary Table 1. Time Course of Hydrohydrazidation of (*E*)-diphenyl(styryl)silane.

Entry	Time (h)	**4a** (%)	**12** (%)	**13** (%)
1	0	0	100	0
2	0.5	4	95	1
3	1	9	89	2
4	1.5	14	83	3
5	2	22	74	4
6	3	33	62	5
7	4	48	43	9
8	5	60	31	9

9	6	76	14	10
10	8	85	5	10
11	10	86	4	10
12	12	87	2	11

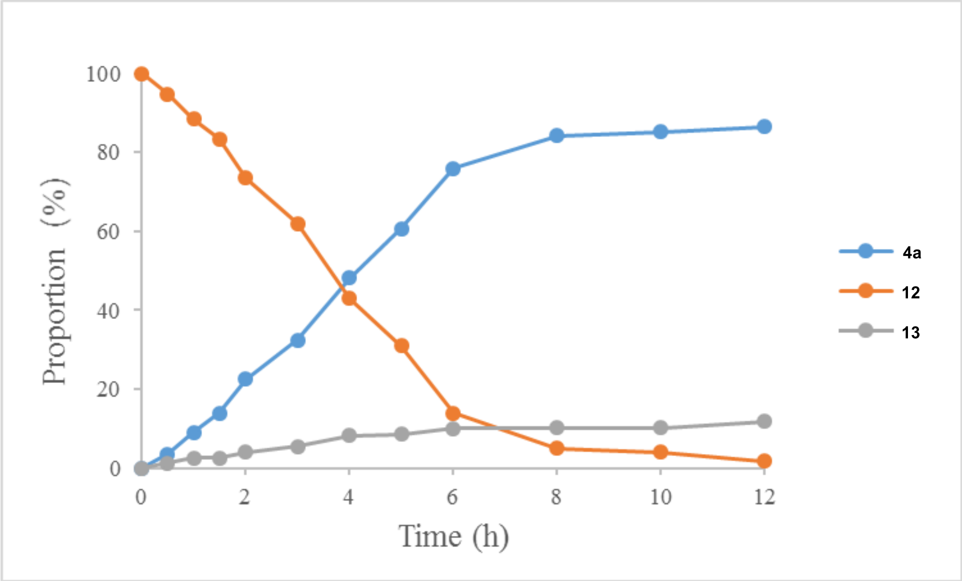

Supplementary Figure 6. Plot of Yield of Components in Different Time Interval.

D) Control Experiments

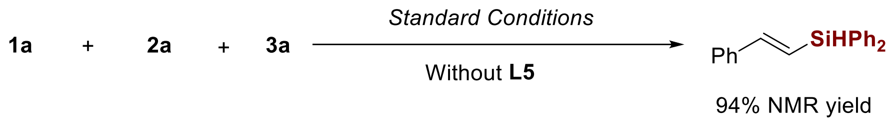

Supplementary Figure 7. Control Experiment without **L5**

The experiment was conducted according to the general procedure using 33 μL (0.93 g/mL, 0.30 mmol) of phenylacetylene, 56 μL (0.99 g/mL, 0.30 mmol) of diphenylsilane, 0.0024 g (0.0030 mmol, 1 mol%) of Xantphos•$CoBr_2$, 9 μL (1.0 M in THF, 0.0090 mmol, 3 mol%) of $NaBHEt_3$, 1.2 mL (0.25 M) of Et_2O, 45 μL (0.88 g/mL, 0.36 mmol) of phenylsilane, 48 μL (1.19 g/mL, 0.30 mmol) of ethyl 2-diazo-2-phenylacetate, and 16 μL (1.00 g/mL, 0.90 mmol) of H_2O. After 12 h, the resulting solution was quenched. The resulting solution was filtered through a pad of silica gel washed by PE/EtOAc (5/1) (15 mL × 3). The combined filtrate was concentrated in *vacuo* and product of hydrosilylation was observed by ^{1}H NMR in 94% NMR yield, indicating that **L5** was

essential for hydrohydrazidation step.

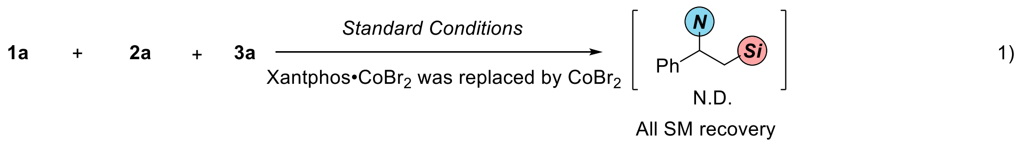

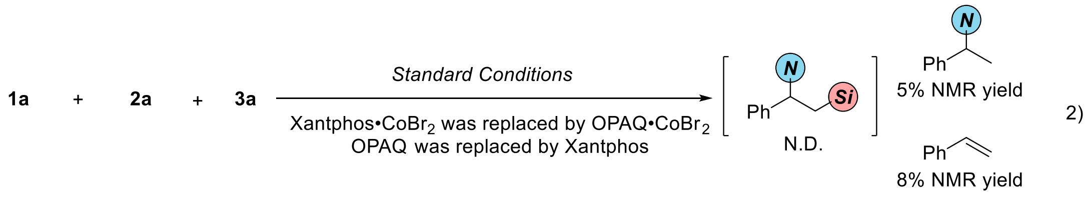

Supplementary Figure 8. Control Experiment with $CoBr_2$ or $OPAQCoBr_2$+Xantphos

For the equation 1, a 25 mL Schlenk flask equipped with a magnetic stirrer and a flanging rubber plug was dried with flame under vacuum. When cooled to ambient temperature, it was vacuumed and flushed with N_2. This degassing procedure was repeated for three times. Then 0.0007 g (0.0030 mmol, 1 mol%) of $CoBr_2$, 1.2 mL (0.25 M) of Et_2O, 9 μL (1.0 M in THF, 0.0090 mmol, 3 mol%) of $NaBHEt_3$, 56 μL (0.99 g/mL, 0.30 mmol) of diphenylsilane, 33 μL (0.93 g/mL, 0.30 mmol) of phenylacetylene, 0.0020 g (0.0063 mmol, 2 mol%) of **L5**, 45 μL (0.88 g/mL, 0.36 mmol) of phenylsilane, 48 μL (1.19 g/mL, 0.30 mmol) of ethyl 2-diazo-2-phenylacetate, and 16 μL (1.00 g/mL, 0.90 mmol) of H_2O. After 12 h, the resulting solution was quenched. The resulting solution was filtered through a pad of silica gel washed by PE/EtOAc (5/1) (15 mL × 3). The combined filtrate was concentrated in *vacuo* and no desired product was observed by ^1H NMR. For the equation 2, a 25 mL Schlenk flask equipped with a magnetic stirrer and a flanging rubber plug was dried with flame under vacuum. When cooled to ambient temperature, it was vacuumed and flushed with N_2. This degassing procedure was repeated for three times. Then 0.0016 g (0.0030 mmol, 1 mol%) of OPAQ•$CoBr_2$, 1.2 mL (0.25 M) of Et_2O, 9 μL (1.0 M in THF, 0.0090 mmol, 3 mol%) of $NaBHEt_3$, 56 μL (0.99 g/mL, 0.30 mmol) of diphenylsilane, 33 μL (0.93 g/mL, 0.30 mmol) of phenylacetylene, 0.0035 g (0.0060 mmol, 2 mol%) of Xantphos, 45 μL (0.88 g/mL, 0.36 mmol) of phenylsilane, 48 μL (1.19 g/mL, 0.30 mmol) of ethyl 2-diazo-2-phenylacetate, and 16 μL (1.00 g/mL, 0.90 mmol) of H_2O. After 12 h, the resulting solution was quenched. The resulting

solution was filtered through a pad of silica gel washed by PE/EtOAc (5/1) (15 mL × 3). The combined filtrate was concentrated in *vacuo* and no desired product was observed by ^1H NMR, 5% of sequential hydrogenation/hydrohydrazidation product and 8% of styrene were observed, indicating that ligand relay between Xantphos and **L5** may play a role in sequential hydrosilylation/hydrohydrazidation of alkynes. The choice of the precatalyst did matters and Xantphos•$CoBr_2$ is labile after activation for the ligand exchange to **L5**.

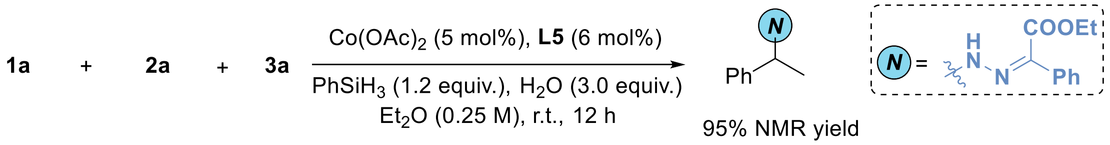

Supplementary Figure 9. Control Experiment without Xantphos•$CoBr_2$ and $NaBHEt_3$.

A 25 mL Schlenk flask equipped with a magnetic stirrer and a flanging rubber plug was dried with flame under vacuum. When cooled to ambient temperature, it was vacuumed and flushed with N_2. This degassing procedure was repeated for three times. Then 0.0028 g (0.015 mmol, 5 mol%) of $Co(OAc)_2$, 0.0059 g (0.018 mmol, 6 mol%) of **L5**, 1.2 mL (0.25 M) of Et_2O were added sequentially. The mixture was stirred for 30 mins. Then 56 μL (0.99 g/mL, 0.30 mmol) of diphenylsilane, 33 μL (0.93 g/mL, 0.30 mmol) of phenylacetylene was added in sequence and stirred for 5 s. After that, 45 μL (0.88 g/mL, 0.36 mmol, 1.2 equiv.) of $PhSiH_3$, 48 μL (1.19 g/mL, 0.3 mmol, 1.0 equiv.) of ethyl 2-diazo-2-phenylacetate, and 16 μL (1.00 g/mL, 0.9 mmol, 3.0 equiv.) of H_2O were added sequentially. After 12 h, the resulting solution was quenched. The resulting solution was filtered through a pad of silica gel washed by PE/EtOAc (5/1) (15 mL × 3). The combined filtrate was concentrated in *vacuo* and product of sequential hydrogenation/hydrohydrazidation was observed by ^1H NMR in 95% NMR yield, indicating that Xantphos•$CoBr_2$ was essential for the first hydrosilylation step.

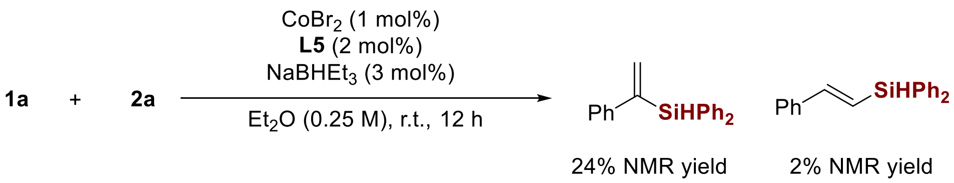

Supplementary Figure 10. Control Experiment using Cobalt and **L5** to Catalyze Hydrosilylation Step

A 25 mL Schlenk flask equipped with a magnetic stirrer and a flanging rubber plug was dried with flame under vacuum. When cooled to ambient temperature, it was vacuumed and flushed with N_2. This degassing procedure was repeated for three times. Then 0.0007 g (0.003 mmol, 1 mol%) of $CoBr_2$, 0.0020 g (0.0063 mmol, 2 mol%) of **L5**, 1.2 mL (0.25 M) of Et_2O were added sequentially. Then 56 μL (0.99 g/mL, 0.30 mmol) of diphenylsilane，9 μL (1.0 M in THF, 0.0090 mmol, 3 mol%) of $NaBHEt_3$, 33 μL (0.93 g/mL, 0.30 mmol) of phenylacetylene was added in sequence and stirred. After 12 h, the resulting solution was quenched and filtered through a pad of silica gel washed by PE/EtOAc (5/1) (15 mL × 3). The combined filtrate was concentrated in *vacuo* and only 24% yield of Markovnikov hydrosilylation product was observed by ^{1}H NMR with 2% yield of *anti*-Markovnikov hydrosilylation product, indicating that Cobalt salts and **L5** could hardly not catalyze the *anti*-Markovnikov hydrosilylation of alkynes.

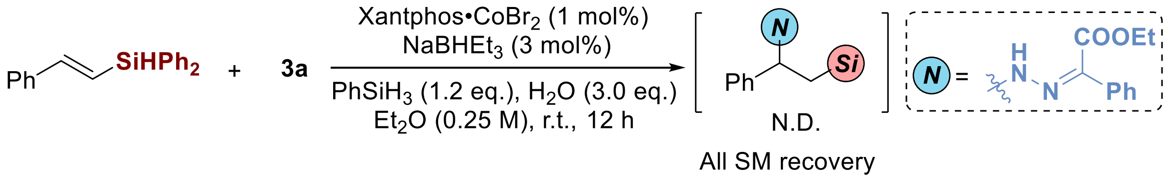

Supplementary Figure 11. Control Experiment using Xantphos•$CoBr_2$ to Catalyze Hydrohydrazidation Step

The experiment was conducted according to the general procedure using 0.0024 g (0.0030 mmol, 1 mol%) of Xantphos•$CoBr_2$, 9 μL (1.0 M in THF, 0.0090 mmol, 3 mol%) of $NaBHEt_3$, 0.0858 g (0.30 mmol) of (*E*)-diphenyl(styryl)silane, 1.2 mL (0.25 M) of Et_2O, 45 μL (0.88 g/mL, 0.36 mmol) of phenylsilane, 48 μL (1.19 g/mL, 0.30 mmol) of ethyl 2-diazo-2-phenylacetate, and 16 μL (1.00 g/mL, 0.90 mmol) of H_2O. After 12 h, the resulting solution was quenched and filtered through a pad of silica gel washed by PE/EtOAc (5/1) (15 mL × 3). The combined filtrate was concentrated in *vacuo* and no desired product was observed by ^{1}H NMR, indicating that ligand relay between Xantphos and **L5** may play a role in sequential hydrosilylation/hydrohydrazidation of alkynes.

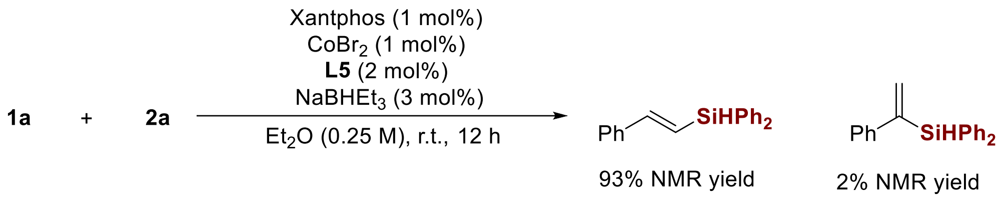

Supplementary Figure 12. Control Experiment using Cobalt, Xantphos, and **L5** to Catalyze Hydrosilylation Step

A 25 mL Schlenk flask equipped with a magnetic stirrer and a flanging rubber plug was dried with flame under vacuum. When cooled to ambient temperature, it was vacuumed and flushed with N_2. This degassing procedure was repeated for three times. Then 0.0007 g (0.0030 mmol, 1 mol%) of $CoBr_2$, 0.0018 g (0.0032 mmol, 1 mol%) of Xantphos, 0.0019 g (0.0060 mmol, 2 mol%) of **L5**, 1.2 mL (0.25 M) of Et_2O were added sequentially. Then 56 μL (0.99 g/mL, 0.30 mmol) of diphenylsilane，9 μL (1.0 M in THF, 0.0090 mmol, 3 mol%) of $NaBHEt_3$, 33 μL (0.93 g/mL, 0.30 mmol) of phenylacetylene was added in sequence and stirred. After 12 h, the resulting solution was quenched and filtered through a pad of silica gel washed by PE/EtOAc (5/1) (15 mL × 3). The combined filtrate was concentrated in *vacuo* and 93% yield of *anti*-Markovnikov hydrosilylation product was observed by ^1H NMR with 2% yield of Markovnikov hydrosilylation product, indicating that **L5** has little effect on hydrosilylation step.

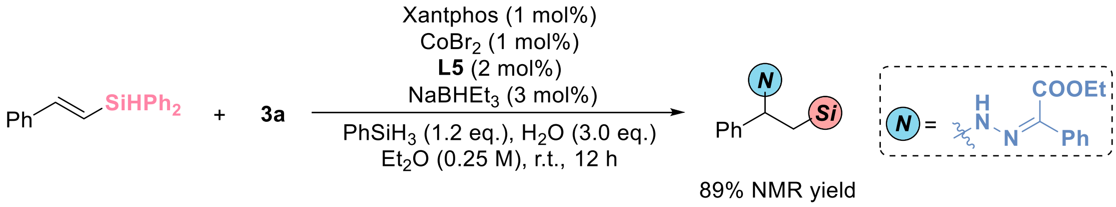

Supplementary Figure 13. Control Experiment using Cobalt salts, Xantphos, and **L5** to Catalyze Hydrohydrazidation Step

A 25 mL Schlenk flask equipped with a magnetic stirrer and a flanging rubber plug was dried with flame under vacuum. When cooled to ambient temperature, it was vacuumed and flushed with N_2. This degassing procedure was repeated for three times. Then 0.0861 g (0.30 mmol) of (*E*)-diphenyl(styryl)silane, 0.0008 g (0.0034 mmol, 1 mol%) of $CoBr_2$, 0.0018 g (0.0032 mmol, 1 mol%) of Xantphos, 0.0020 g (0.0063 mmol, 2 mol%) of **L5**, 1.2 mL (0.25 M) of Et_2O were added

sequentially. Then 9 μL (1.0 M in THF, 0.0090 mmol) of $NaBHEt_3$, 45 μL (0.88 g/mL, 0.36 mmol) of phenylsilane, 48 μL (1.19 g/mL, 0.30 mmol) of ethyl 2-diazo-2-phenylacetate, and 16 μL (1.00 g/mL, 0.90 mmol) of H_2O was added in sequence and stirred. After 12 h, the resulting solution was quenched and filtered through a pad of silica gel washed by PE/EtOAc (5/1) (15 mL × 3). The combined filtrate was concentrated in *vacuo* and 89% yield of final product was observed by ^1H NMR, indicating that Xantphos has little effect on hydrohydrazidation step.

E) Absorption measurement

Ligand relay process was investigated by optic fiber spectrophotometer. A 25 mL Schlenk flask equipped with a magnetic stirrer and a flanging rubber plug was dried with flame under vacuum. When cooled to ambient temperature, it was vacuumed and flushed with N_2. This degassing procedure was repeated for three times. Then 0.0024 g (0.003 mmol) of Xantphos•$CoBr_2$ and 1.2 mL THF were added into the flask. 50 μL of the solution was extracted and injected into a quartz cuvette quickly using 3 mL anhydrous THF (3 mM) to dilute the solution. The absorption curve (blue line) was generated immediately. Then 9 μL (1.0 M in THF, 0.0090 mmol) of $NaBHEt_3$ was added into the flask and same operation was conducted. A small peak (λ = 290 nm) emerged (green line). Sequential addition of 0.0018 g (0.0057 mmol) of **L5** (OPAQ) made no difference from the green line in the first 5mins. However, after 30 mins, a broad peak was observed at 468 nm after 30 mins (red line) which is cater to the absorption peak of OPAQ•$CoBr_2$ activated by $NaBHEt_3$ at 467 nm (blue line) indicating that it takes a period of time for the latter ligand to coordinate with the metal, the catalytic active species would emerge and keep active *via* ligand relay process under N_2 atmosphere while no peaks will emerge at 468 nm conducting the reaction under air (Fig. S15). The pink line was similar to the black line indicating that when adding $NaBHEt_3$ and OPAQ to the system, Xantphos might dissociate from the precatalyst.

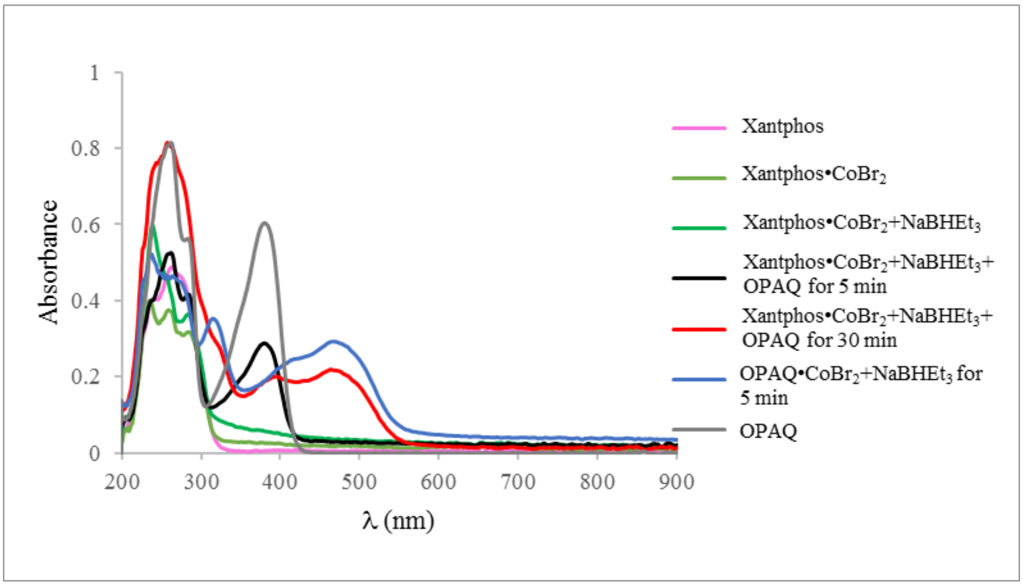

Supplementary Figure 14. Plot of Absorption Measurement for Ligand Relay

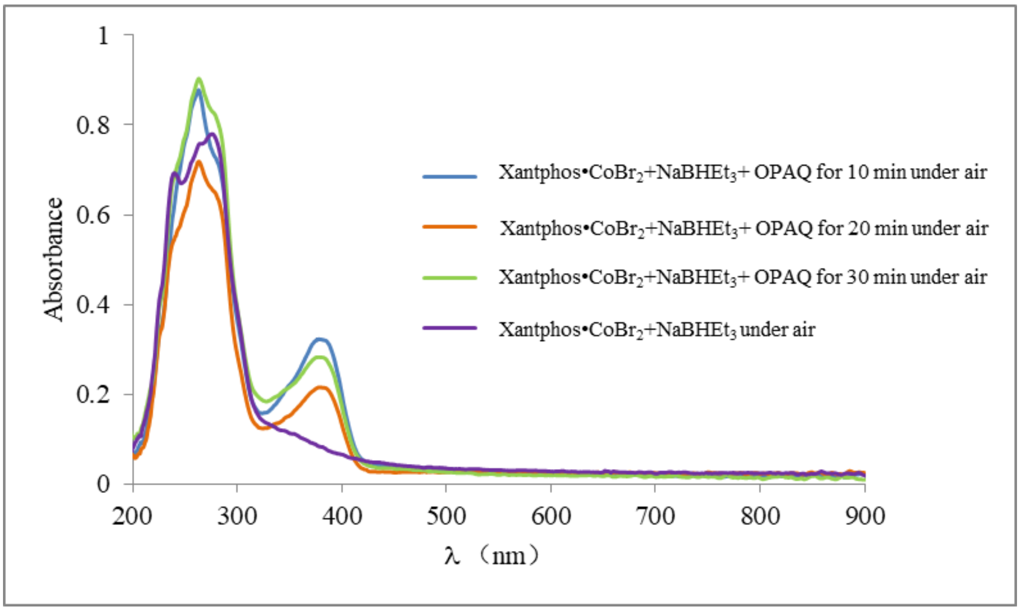

Supplementary Figure 15. Plot of Absorption Measurement for Ligand Relay under Air.

Ligand relay process under standard conditions was also investigated. A 25 mL Schlenk flask equipped with a magnetic stirrer and a flanging rubber plug was dried with flame under vacuum. When cooled to ambient temperature, it was vacuumed and flushed with N_2. This degassing procedure was repeated for three times. Then 0.0480 g (0.060 mmol) of Xantphos•$CoBr_2$, 1.2 mL (0.25 M) of THF, 11.2 μL (0.99 g/mL, 0.06 mmol) of diphenylsilane, 180 μL (1.0 M in THF, 0.18 mmol) of $NaBHEt_3$, 6.6 μL (0.93 g/mL, 0.06 mmol) of phenylacetylene, 0.0019 g (0.060mmol) of

L5, 9.0 μL (0.88 g/mL, 0.072 mmol) of phenylsilane, 9.6 μL (1.19 g/mL, 0.06 mmol) of ethyl 2-diazo-2-phenylacetate, and 3.2 μL (1.00 g/mL, 0.18 mmol) of H_2O were added in sequence. 2 μL of the solution was extracted and injected into a quartz cuvette quickly using 3 mL anhydrous THF (2.4 mM) to dilute the solution. The absorption curve (blue line) was generated immediately at 460 nm. Due to the increase of concentration, the broad peak was emerged at 463 nm after 5 mins (green line). The absorption curves of 10 mins, 30 mins, and 12 h were similar to the curve of 5 mins. This absorption measurement indicated that the ligand relay process would take place in the presence of substrates and hydrogen source under the conditions similar to the catalytic reactions. Absorption measurement of starting materials, **1a**, **2a**, **3a**, and $PhSiH_3$ was also conducted as a reference to Fig. S17.

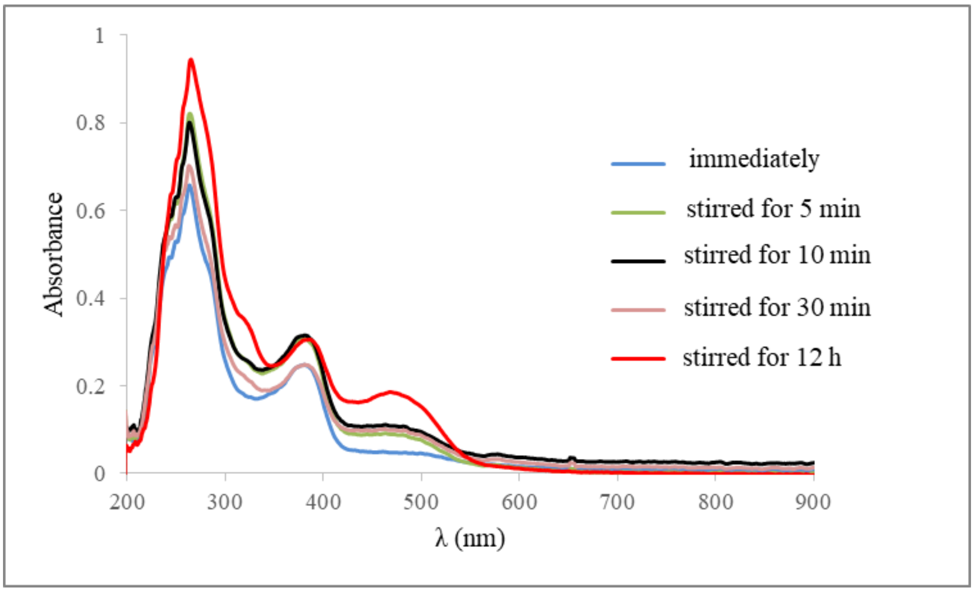

Supplementary Figure 16. Plot of Absorption Measurement for Ligand Relay under Standard Conditions.

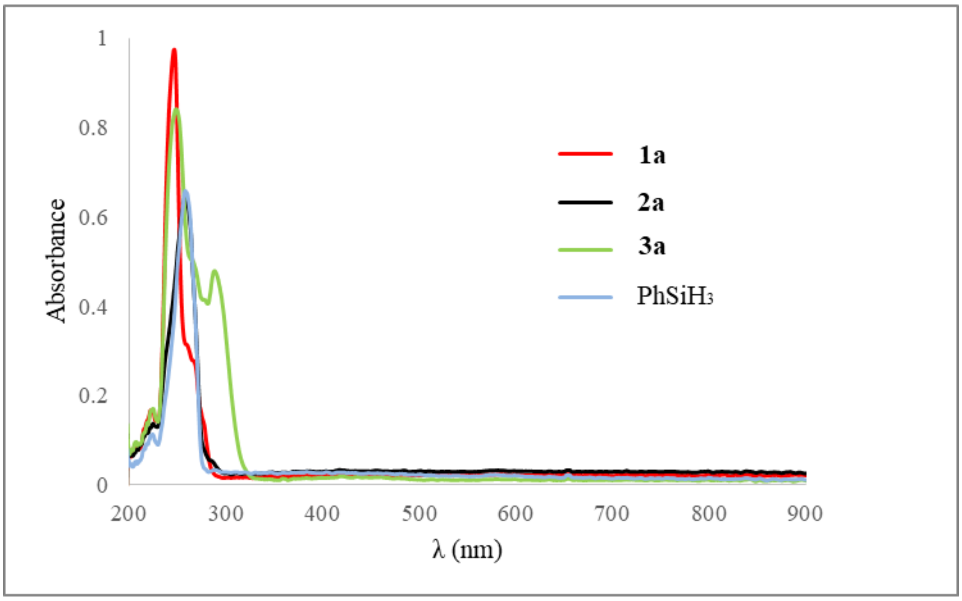

Supplementary Figure 17. Plot of Absorption Measurement for Starting Materials.

X. NMR Spectra

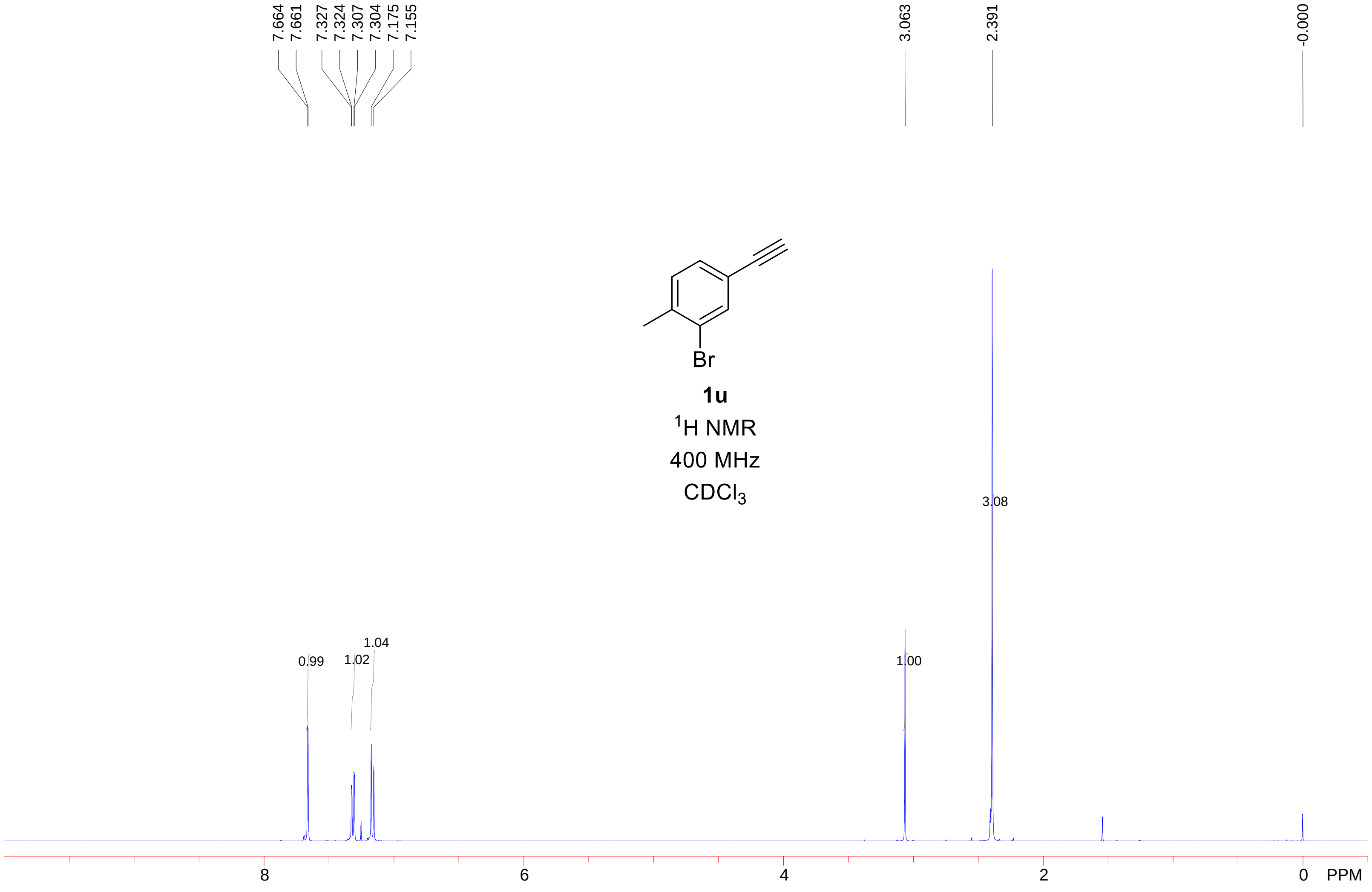

Supplementary Figure 18. ^{1}H NMR spectrum of **1u**.

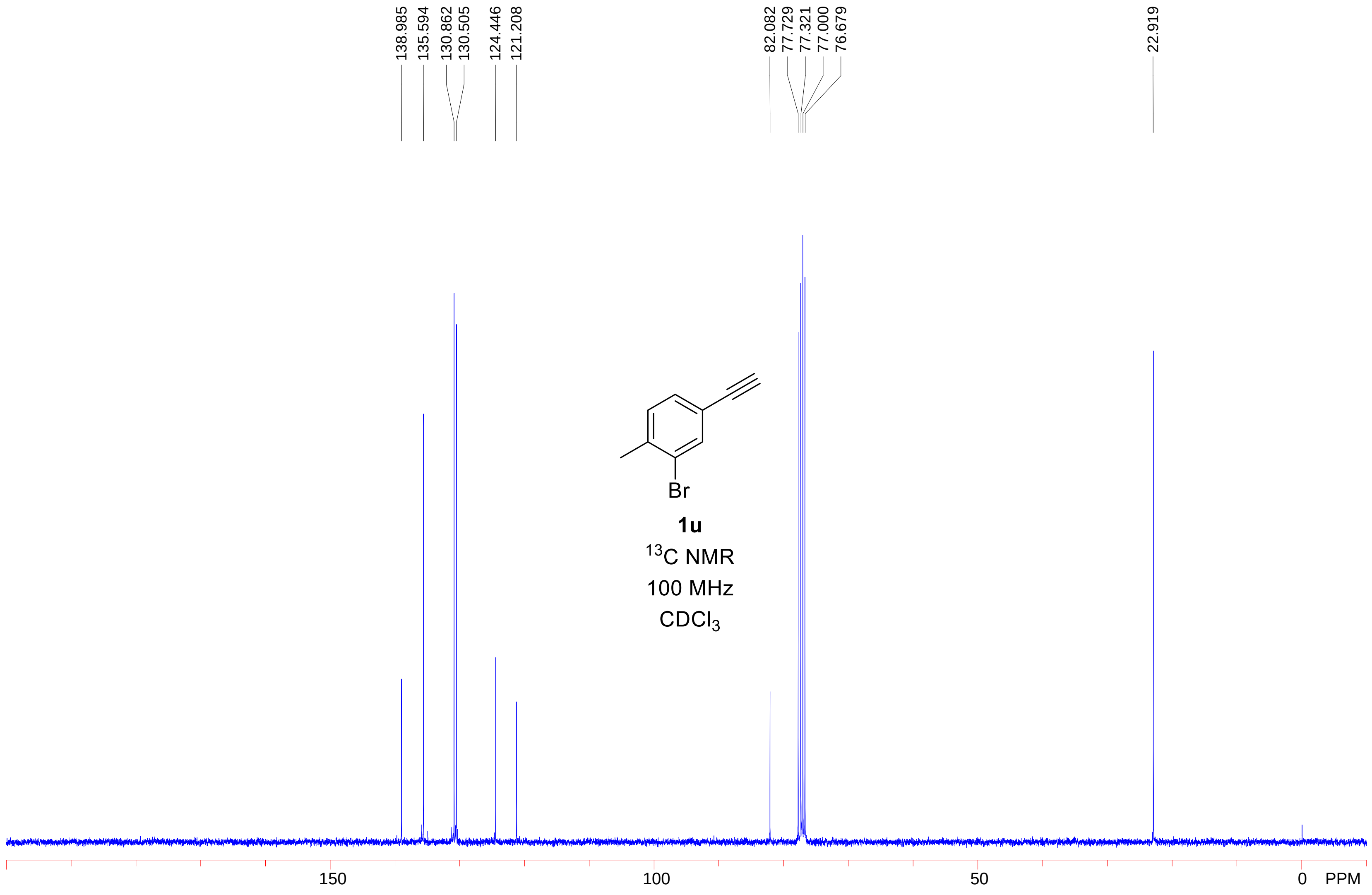

Supplementary Figure 19. ^{13}C NMR spectrum of **1u**.

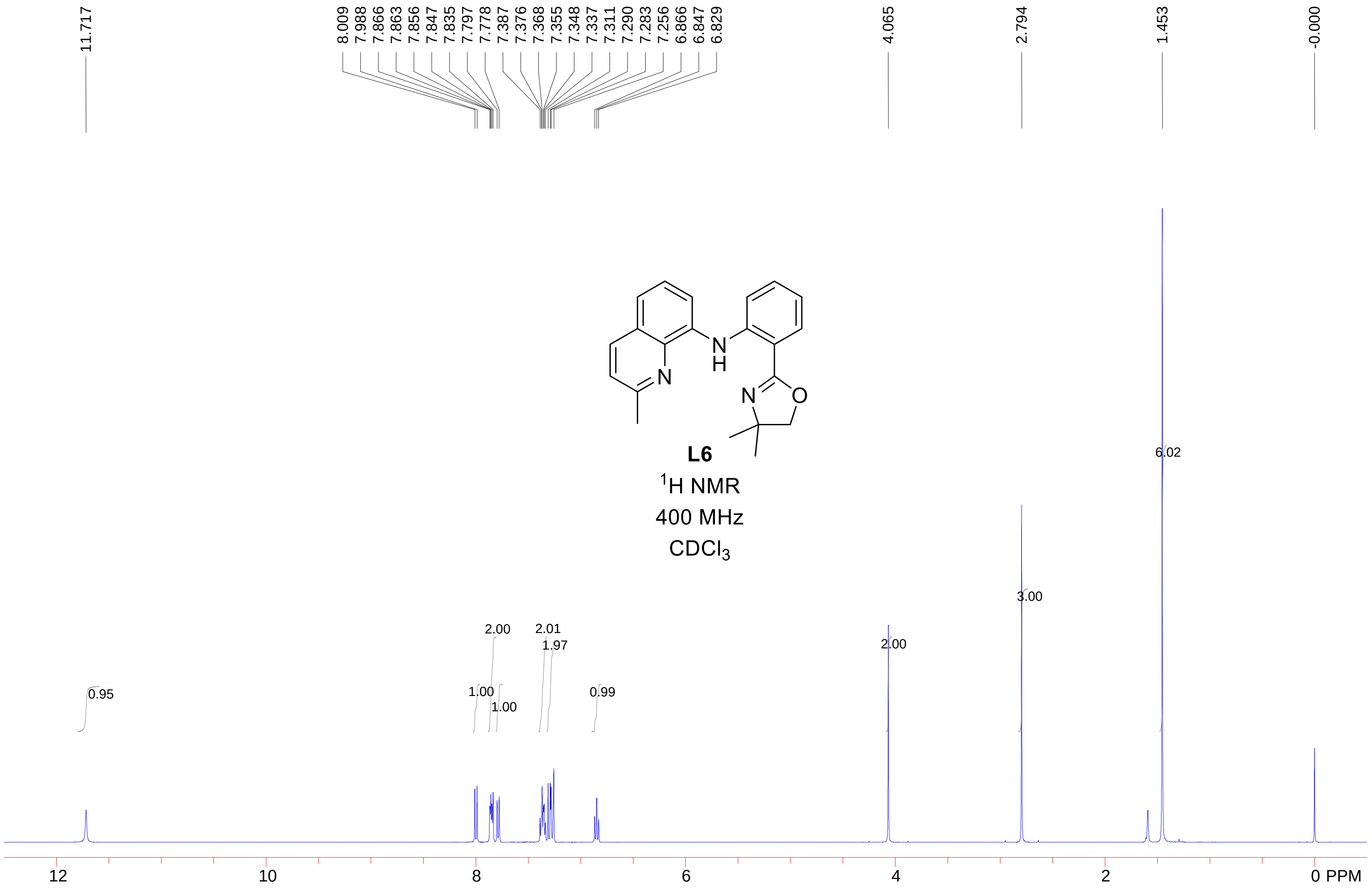

Supplementary Figure 20. ¹H NMR spectrum of **L6**.

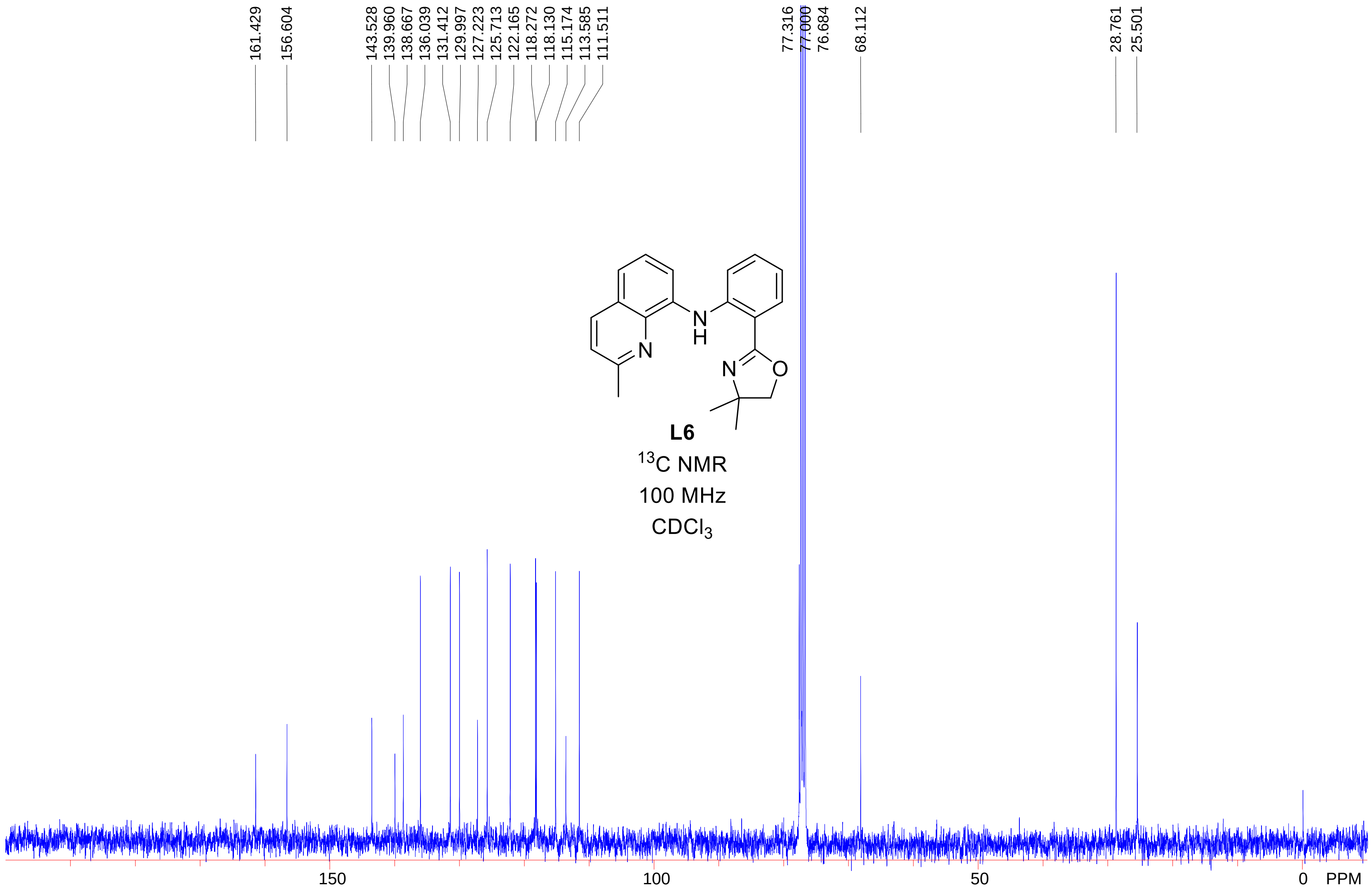

Supplementary Figure 21. ^{13}C NMR spectrum of **L6**.

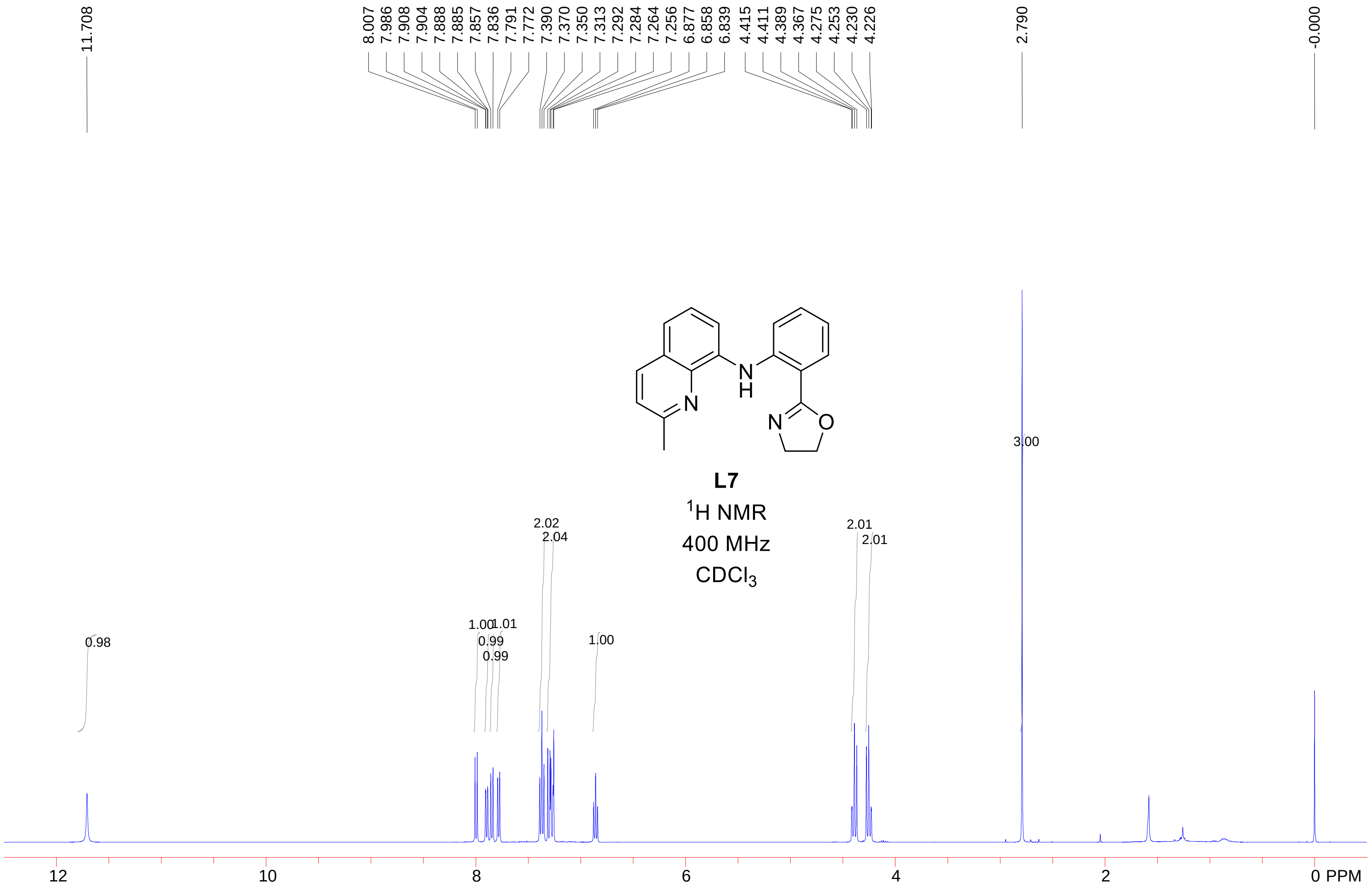

Supplementary Figure 22. ^{1}H NMR spectrum of **L7**.

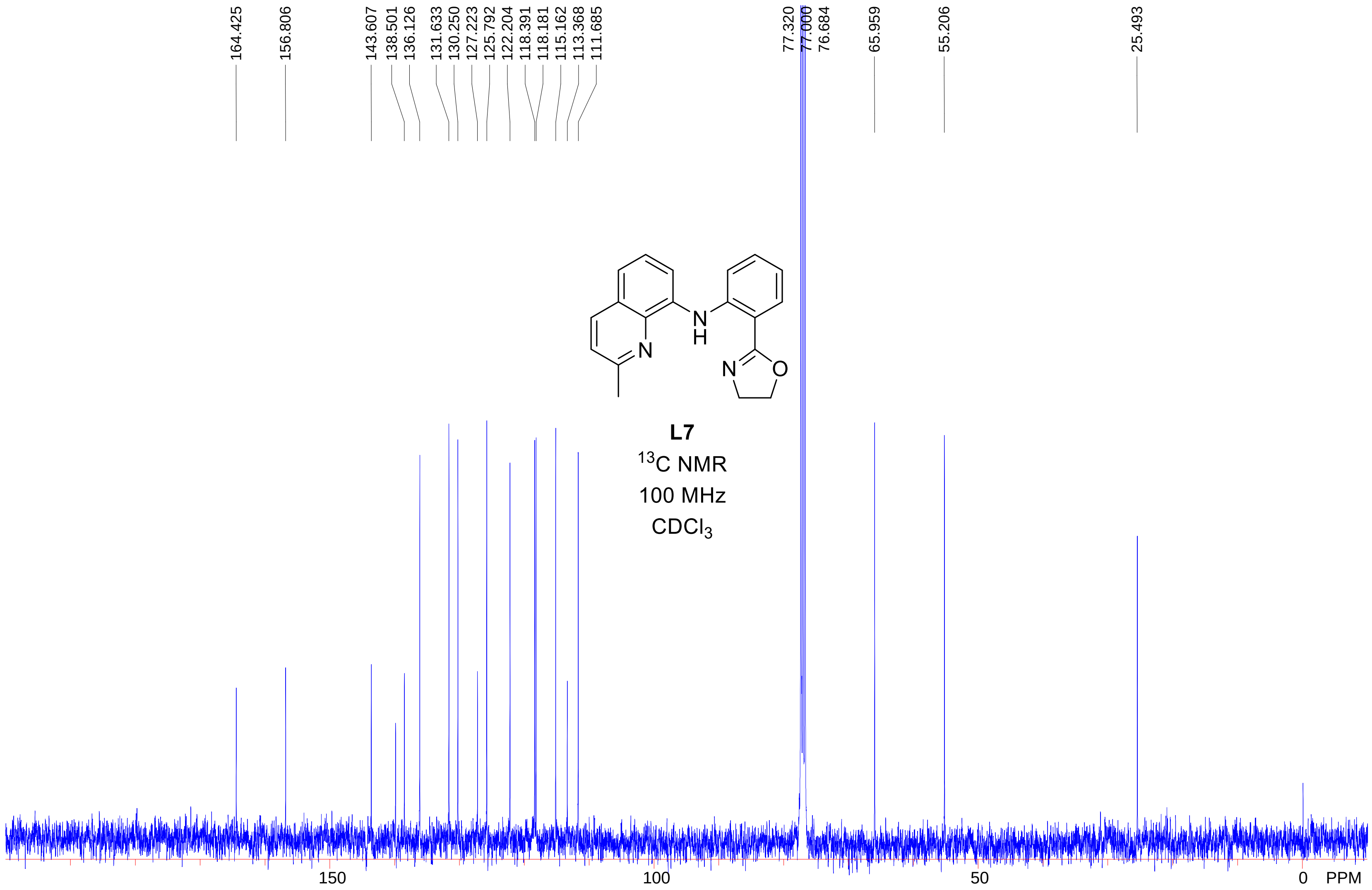

Supplementary Figure 23. ^{13}C NMR spectrum of **L7**.

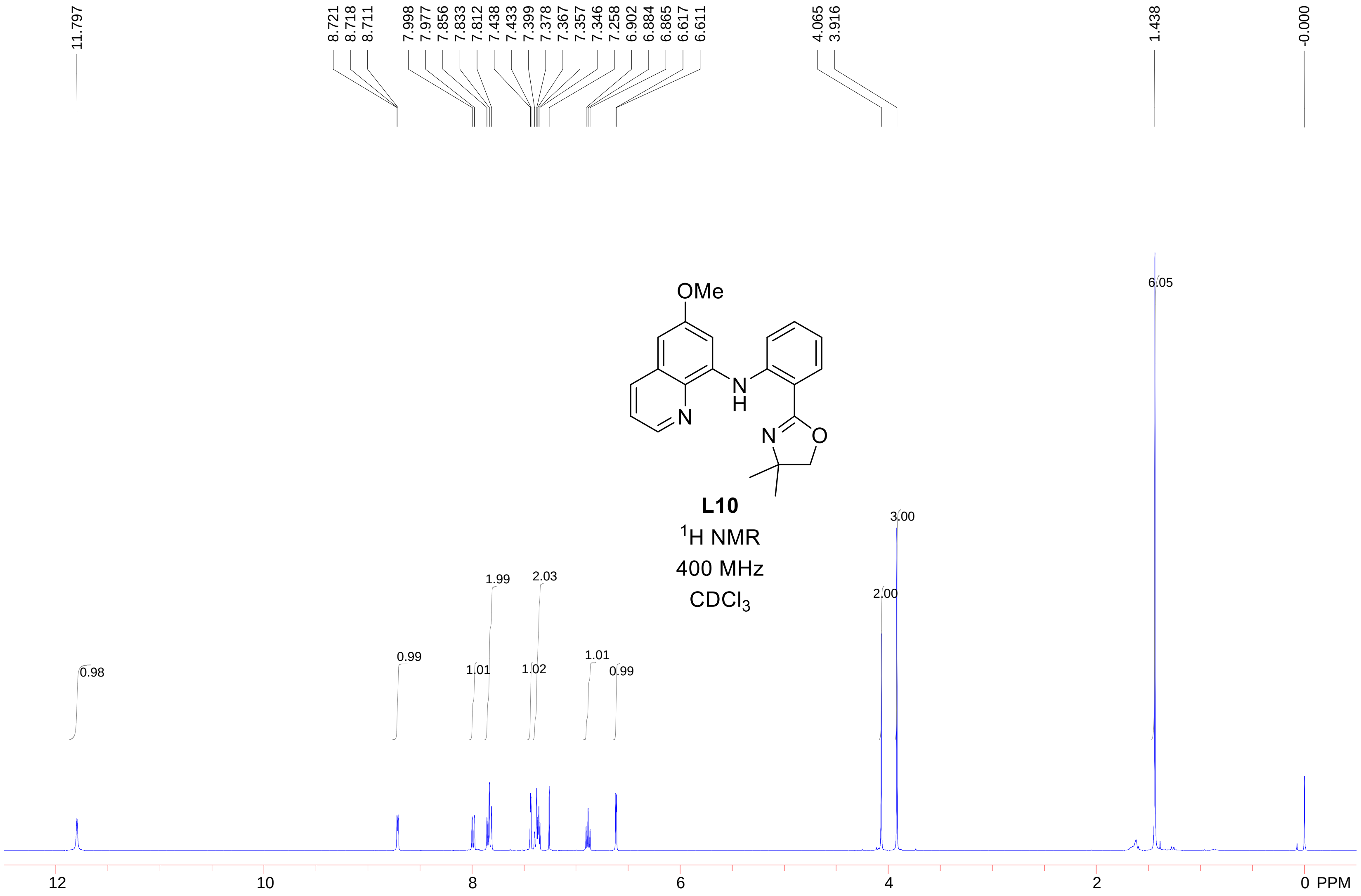

Supplementary Figure 24. ^{1}H NMR spectrum of **L10**.

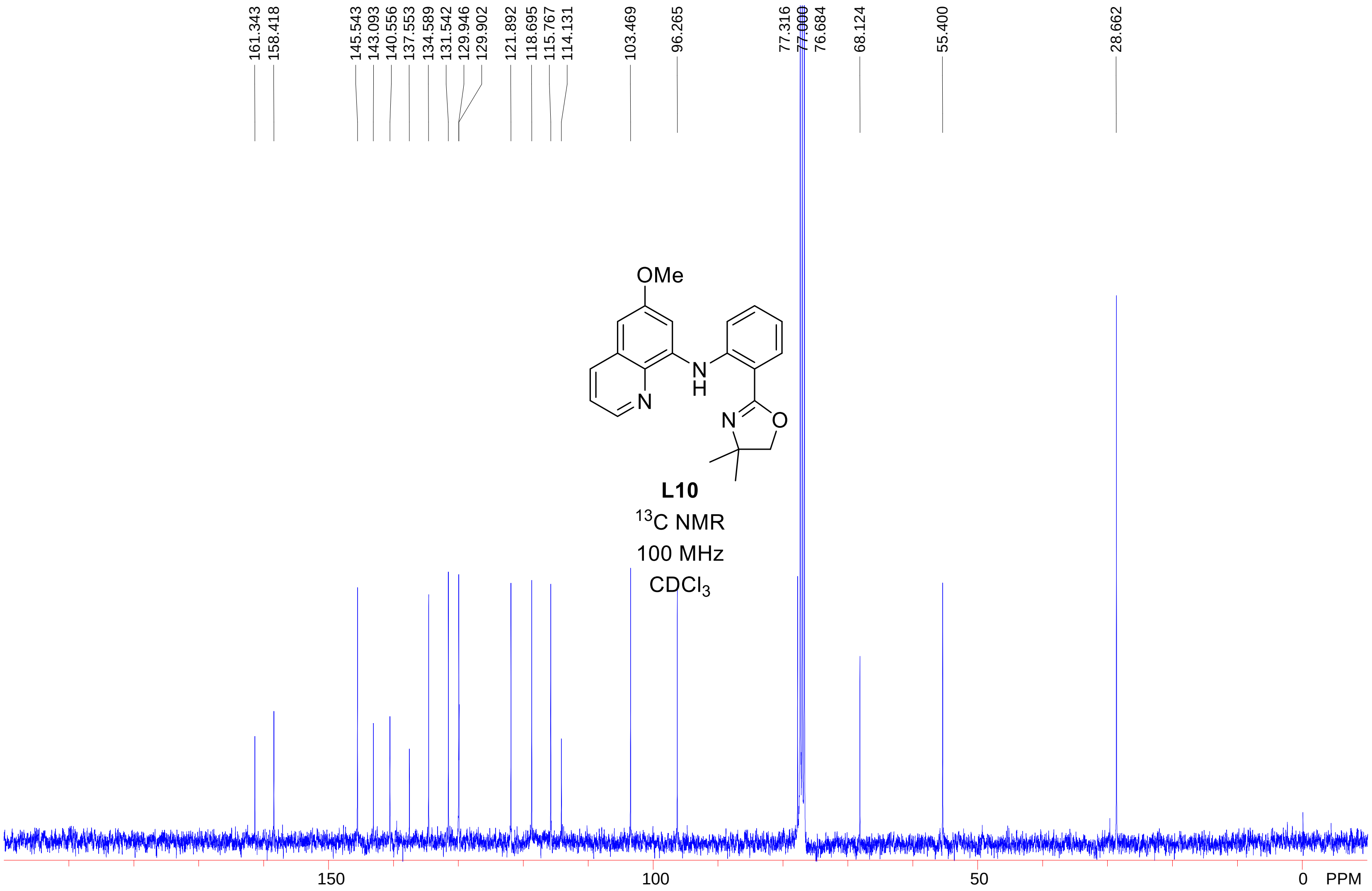

Supplementary Figure 25. ^{13}C NMR spectrum of **L10**.

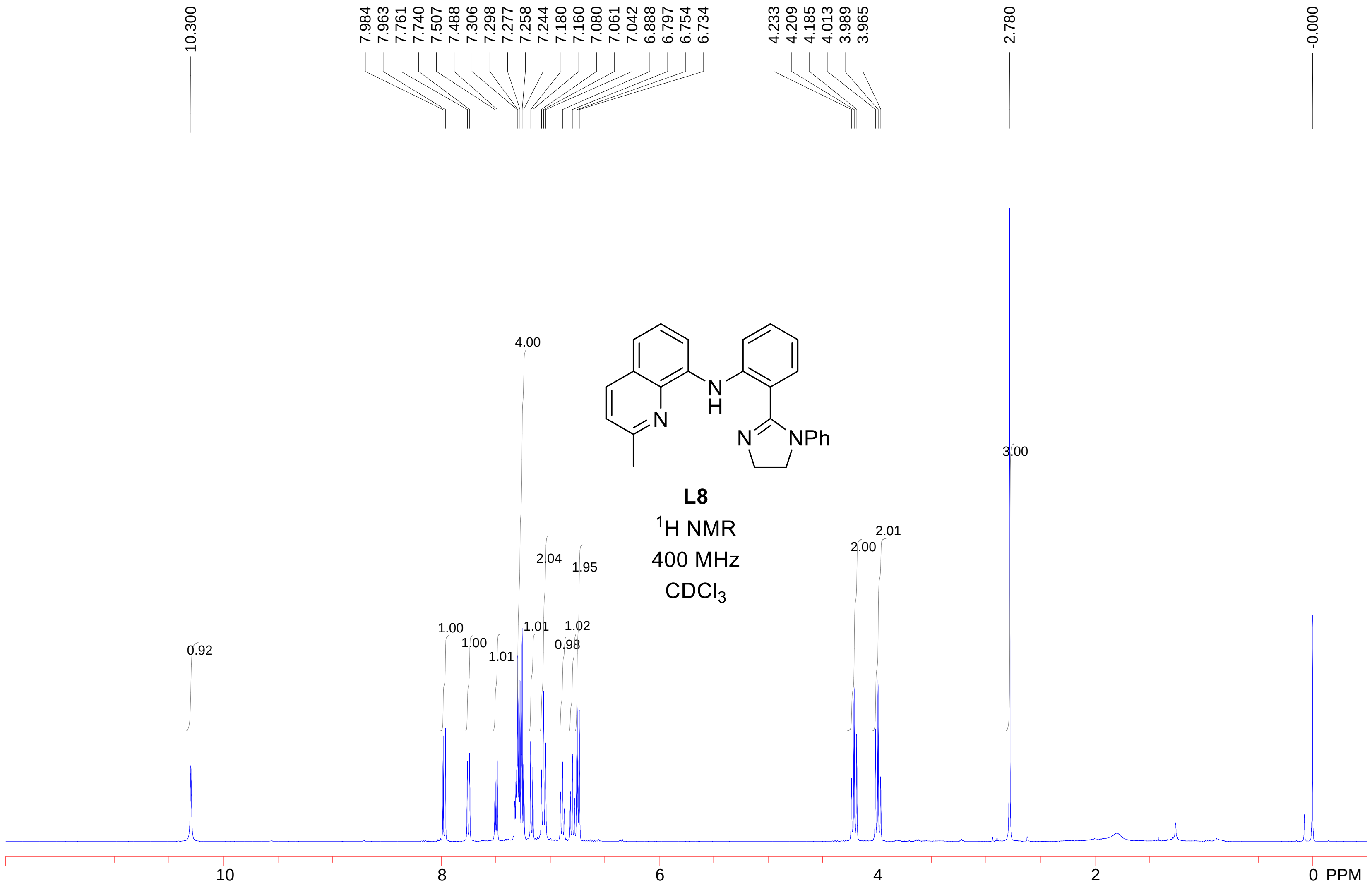

Supplementary Figure 26. ^{1}H NMR spectrum of **L8**.

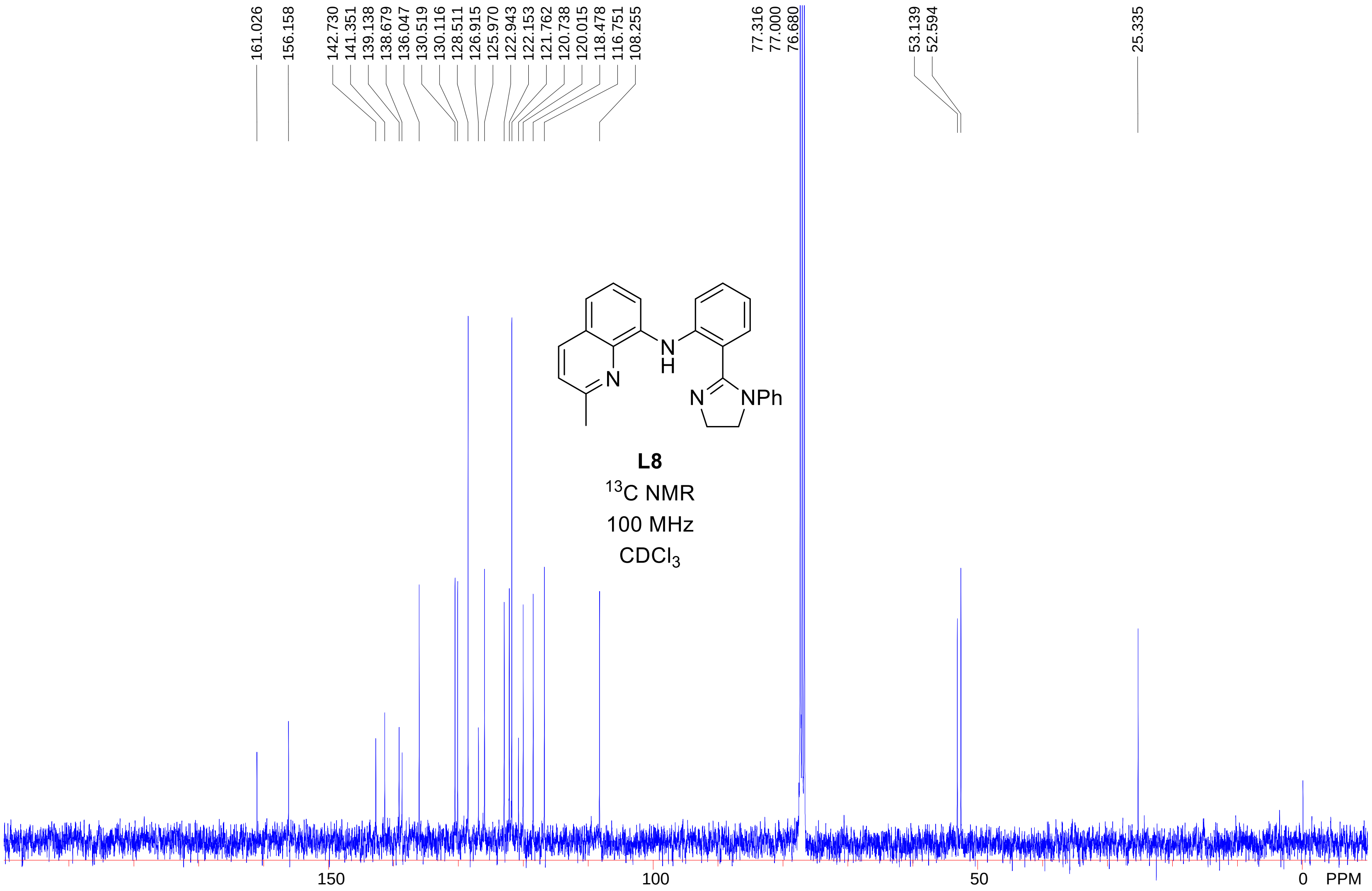

Supplementary Figure 27. ^{13}C NMR spectrum of **L8**.

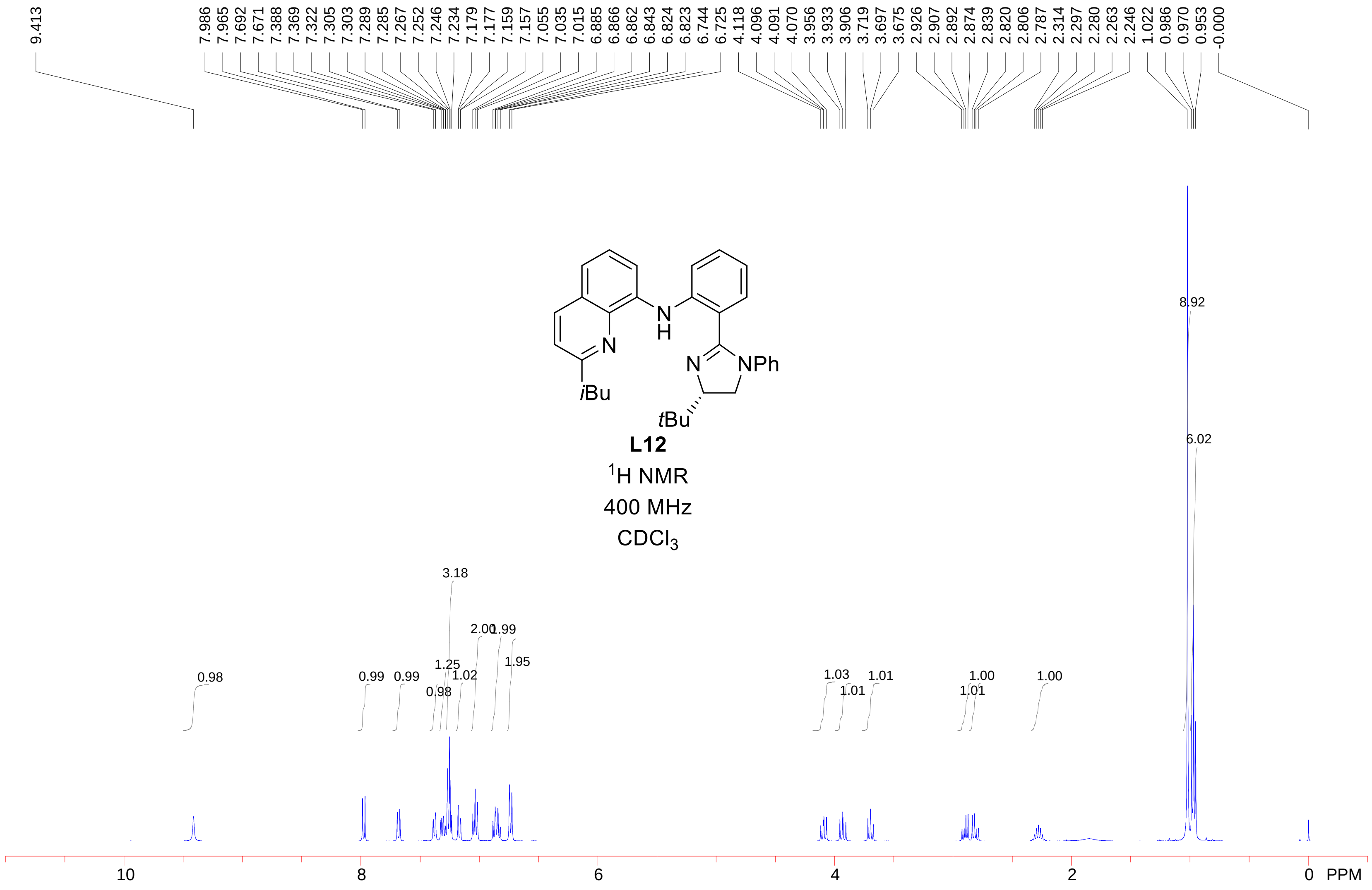

Supplementary Figure 28. ^{1}H NMR spectrum of **L12**.

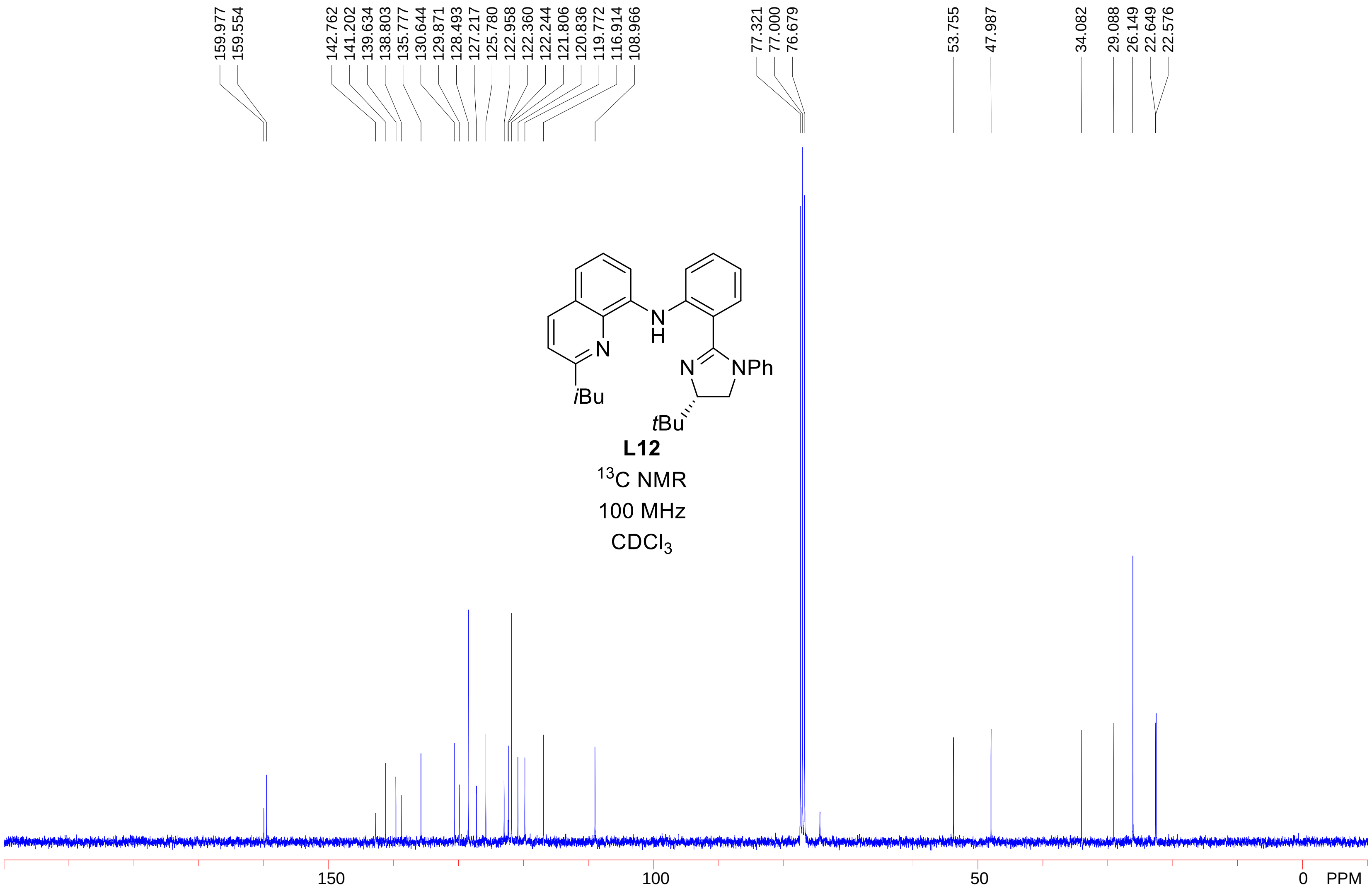

Supplementary Figure 29. ^{13}C NMR spectrum of **L12**.

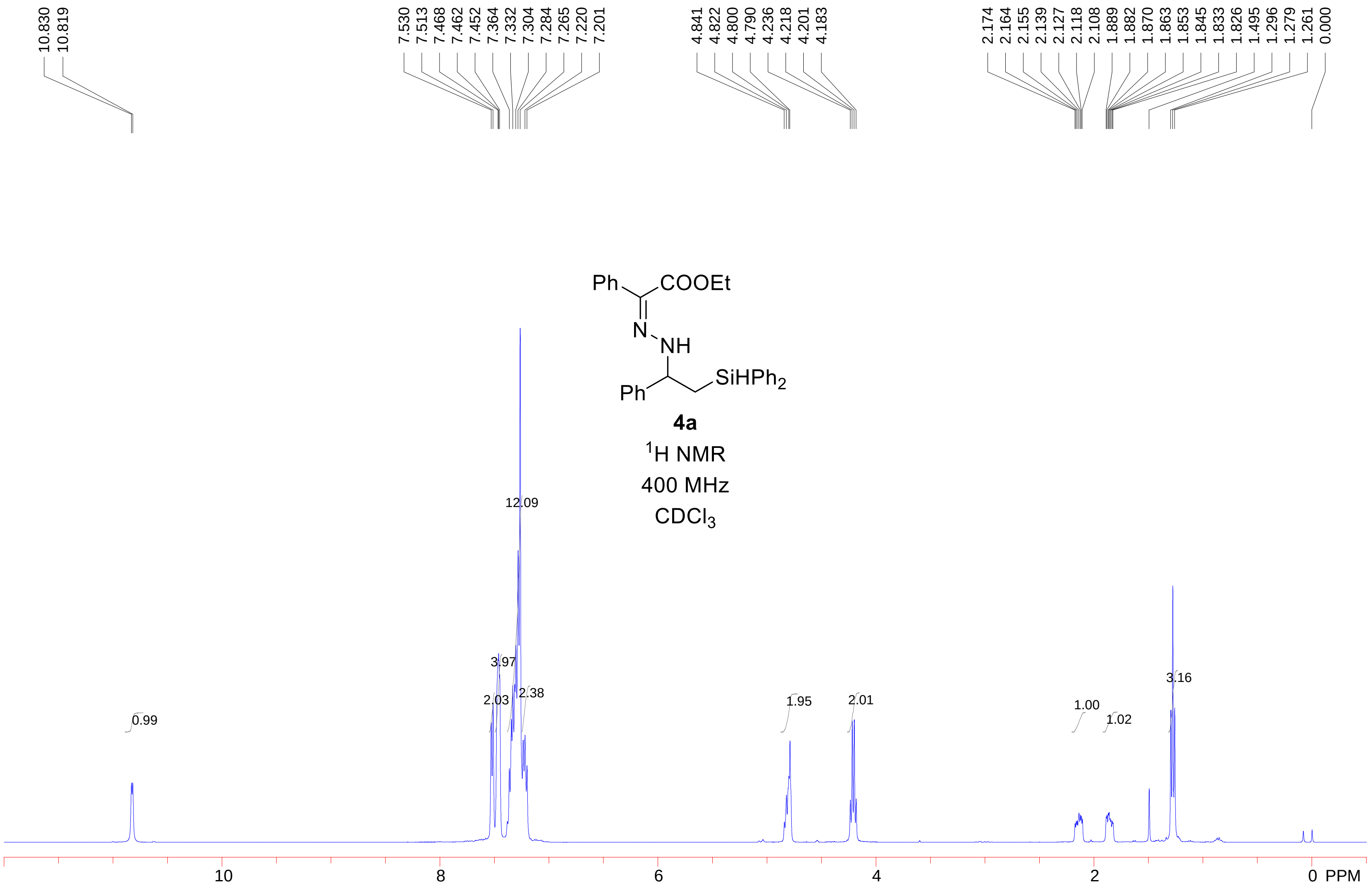

Supplementary Figure 30. ^{1}H NMR spectrum of **4a**.

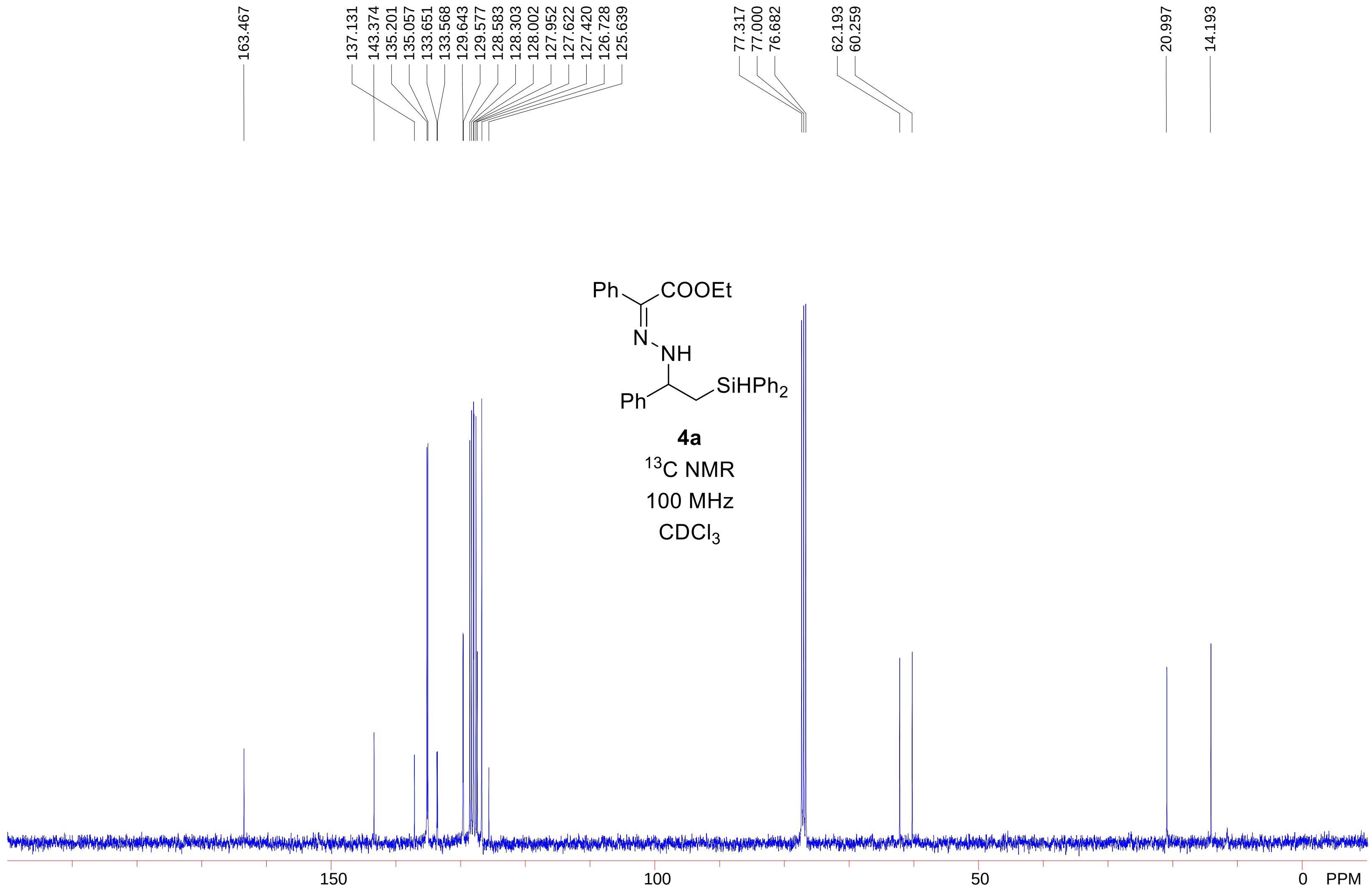

Supplementary Figure 31. ^{13}C NMR spectrum of **4a**.

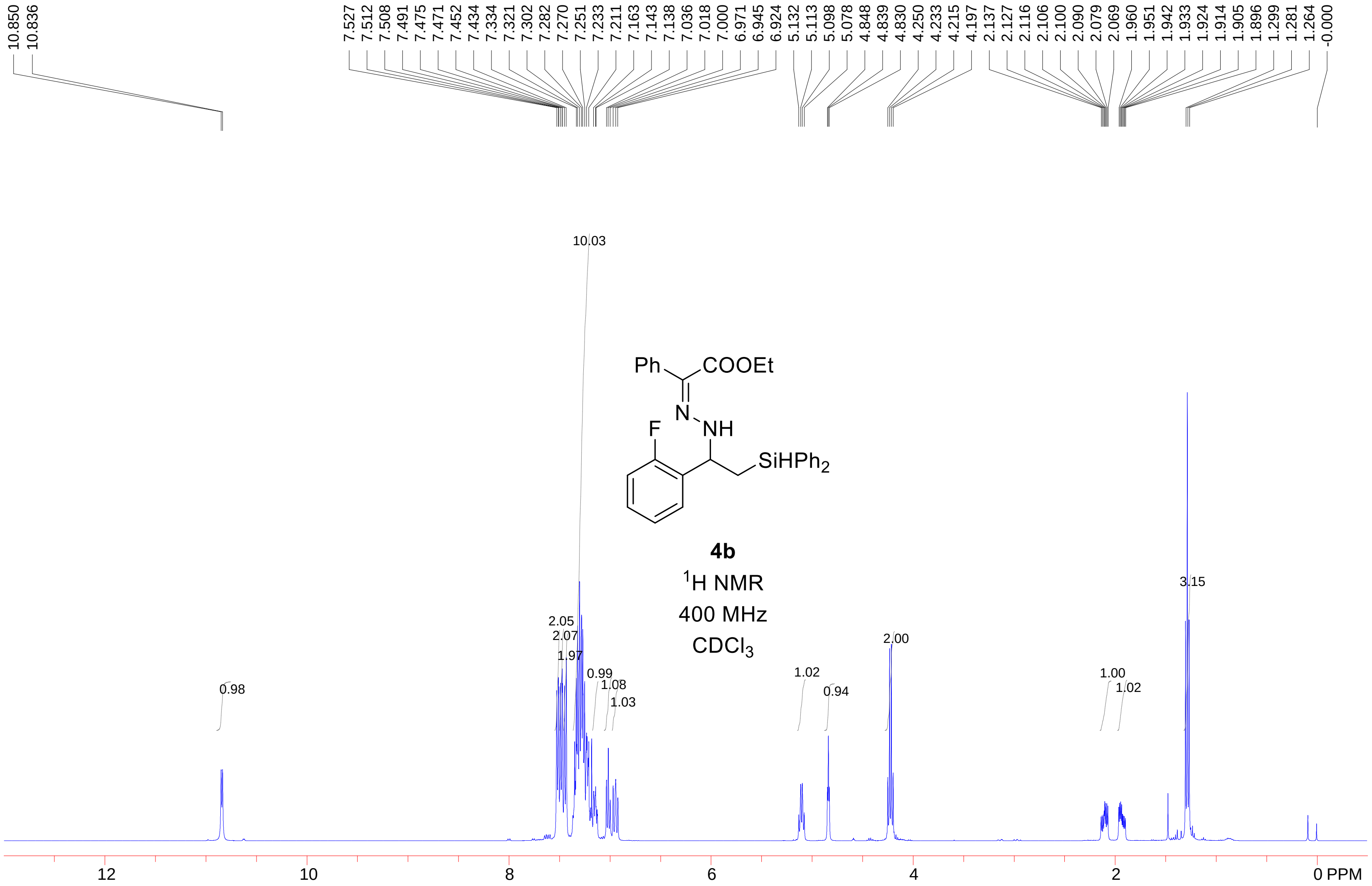

Supplementary Figure 32. ^{1}H NMR spectrum of **4b**.

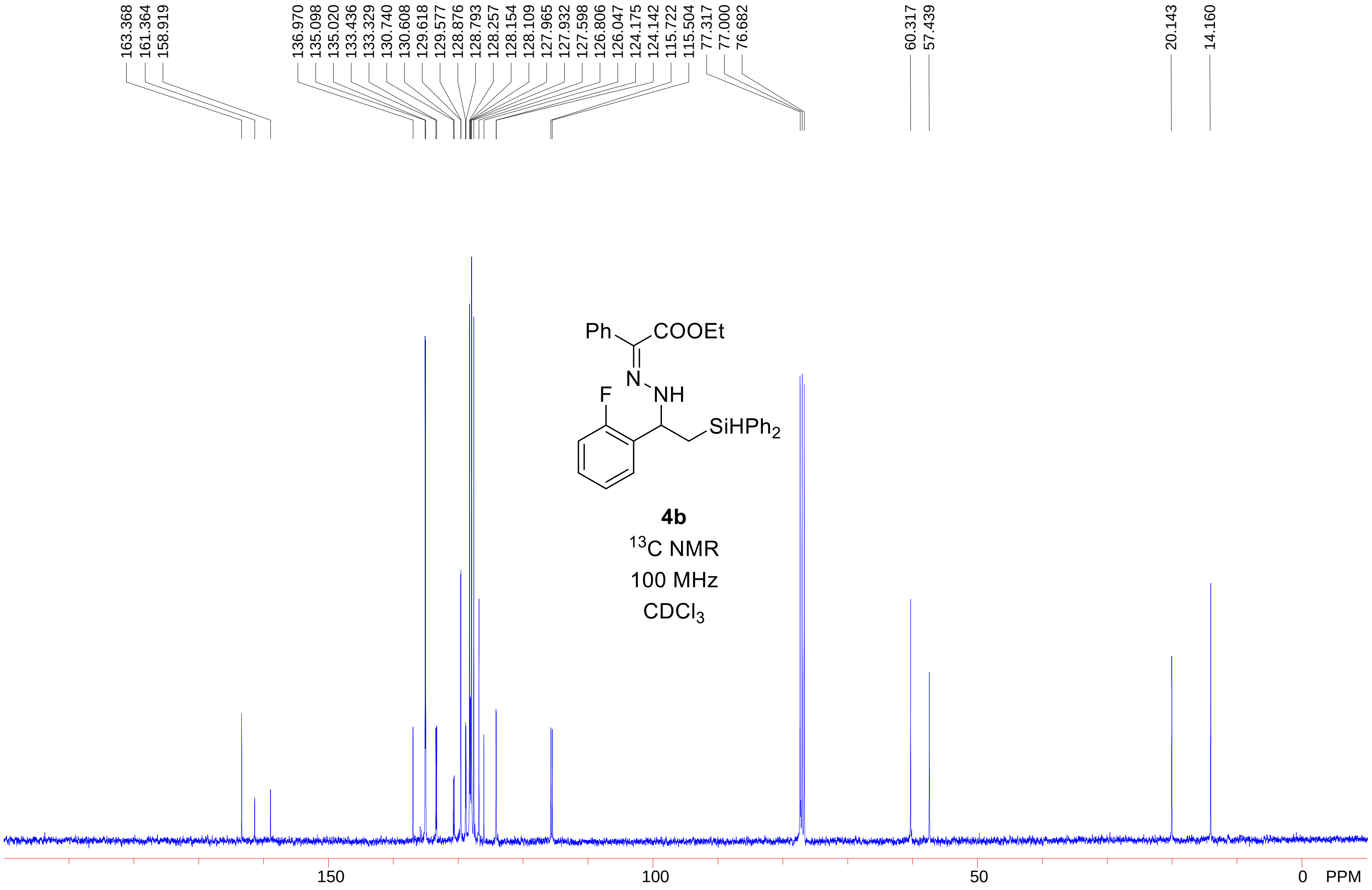

Supplementary Figure 33. ^{13}C NMR spectrum of **4b**.

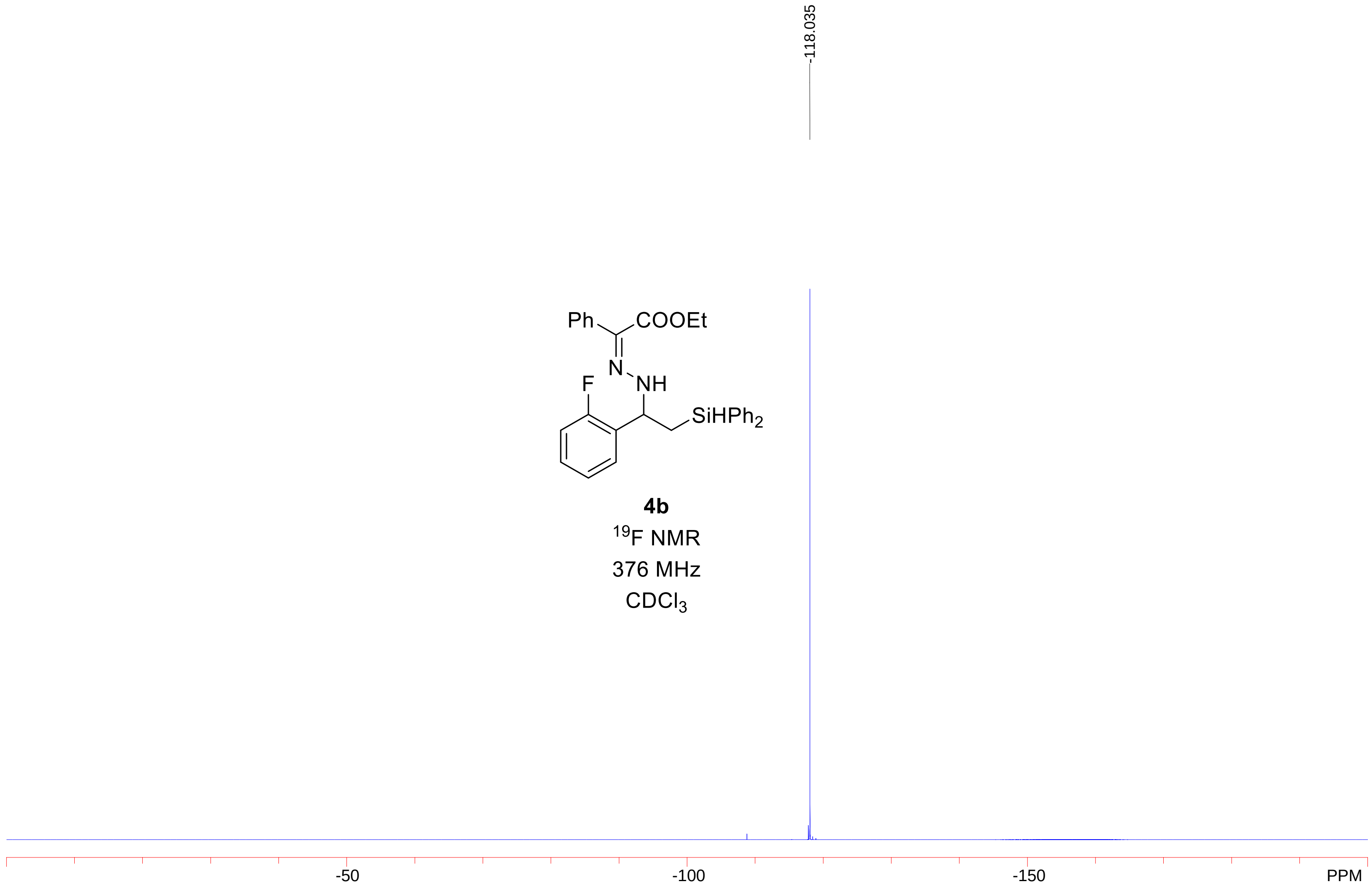

Supplementary Figure 34. ^{19}F NMR spectrum of **4b**.

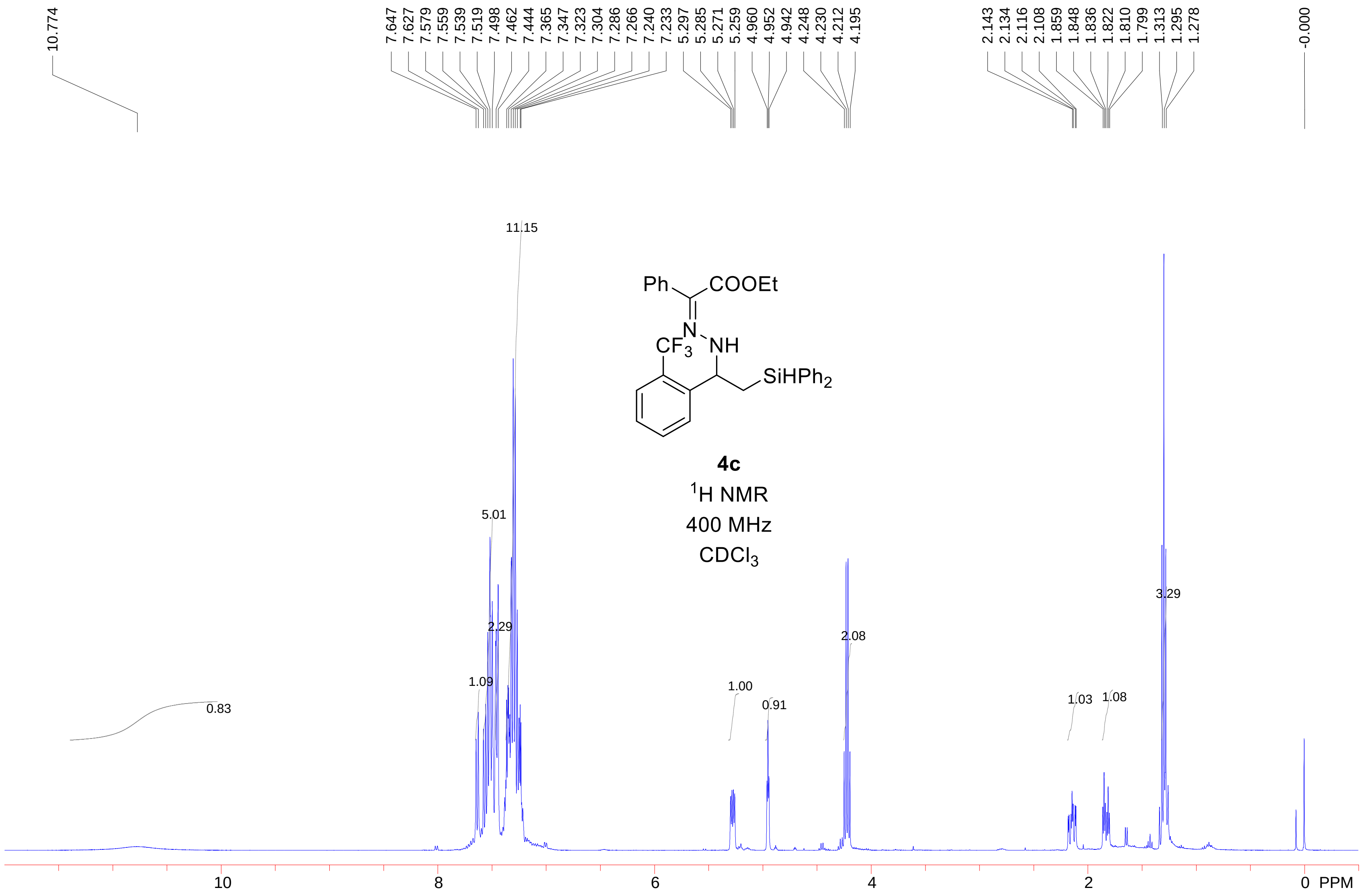

Supplementary Figure 35. ^{1}H NMR spectrum of **4c**.

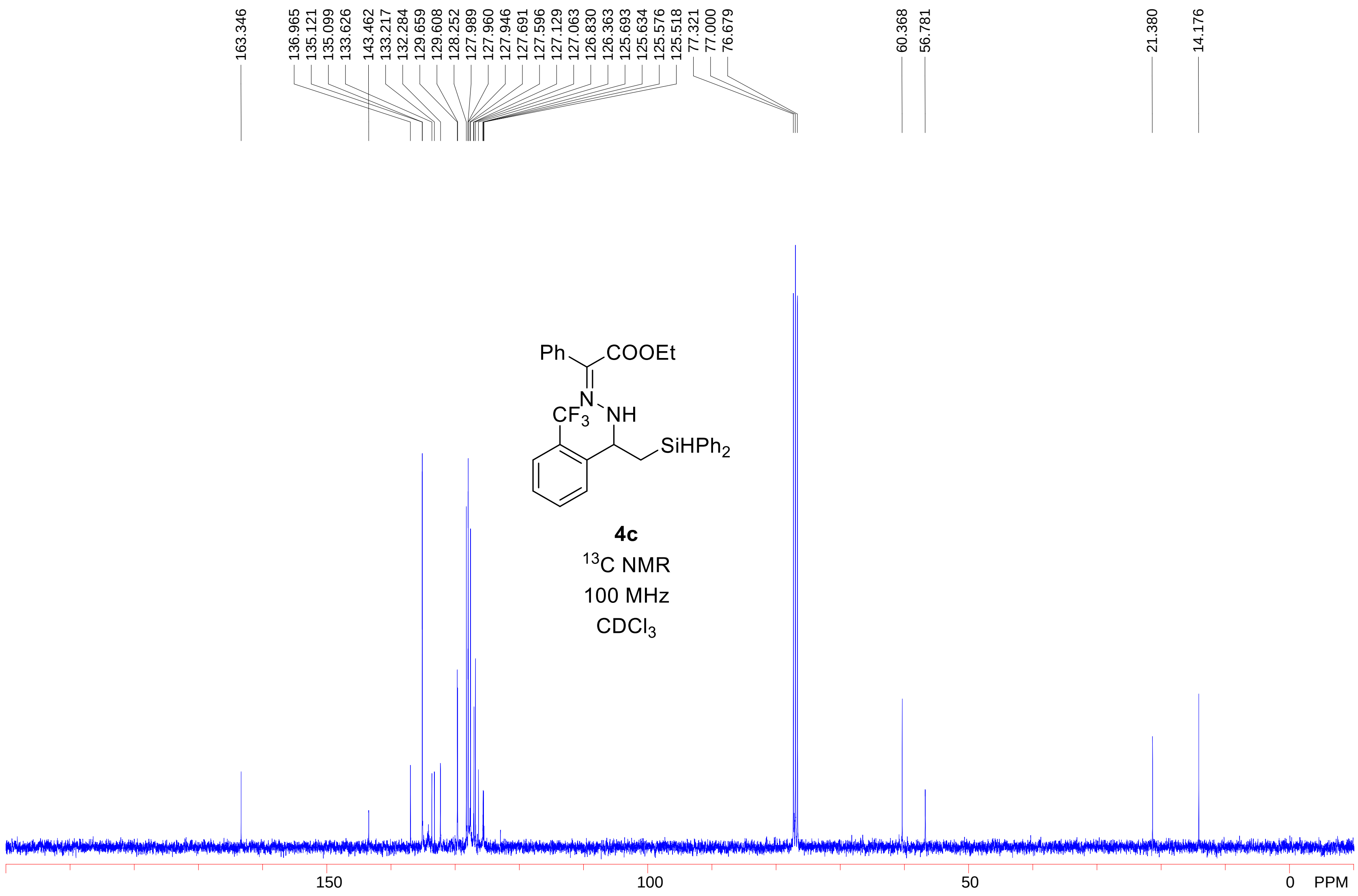

Supplementary Figure 36. ^{13}C NMR spectrum of **4c**.

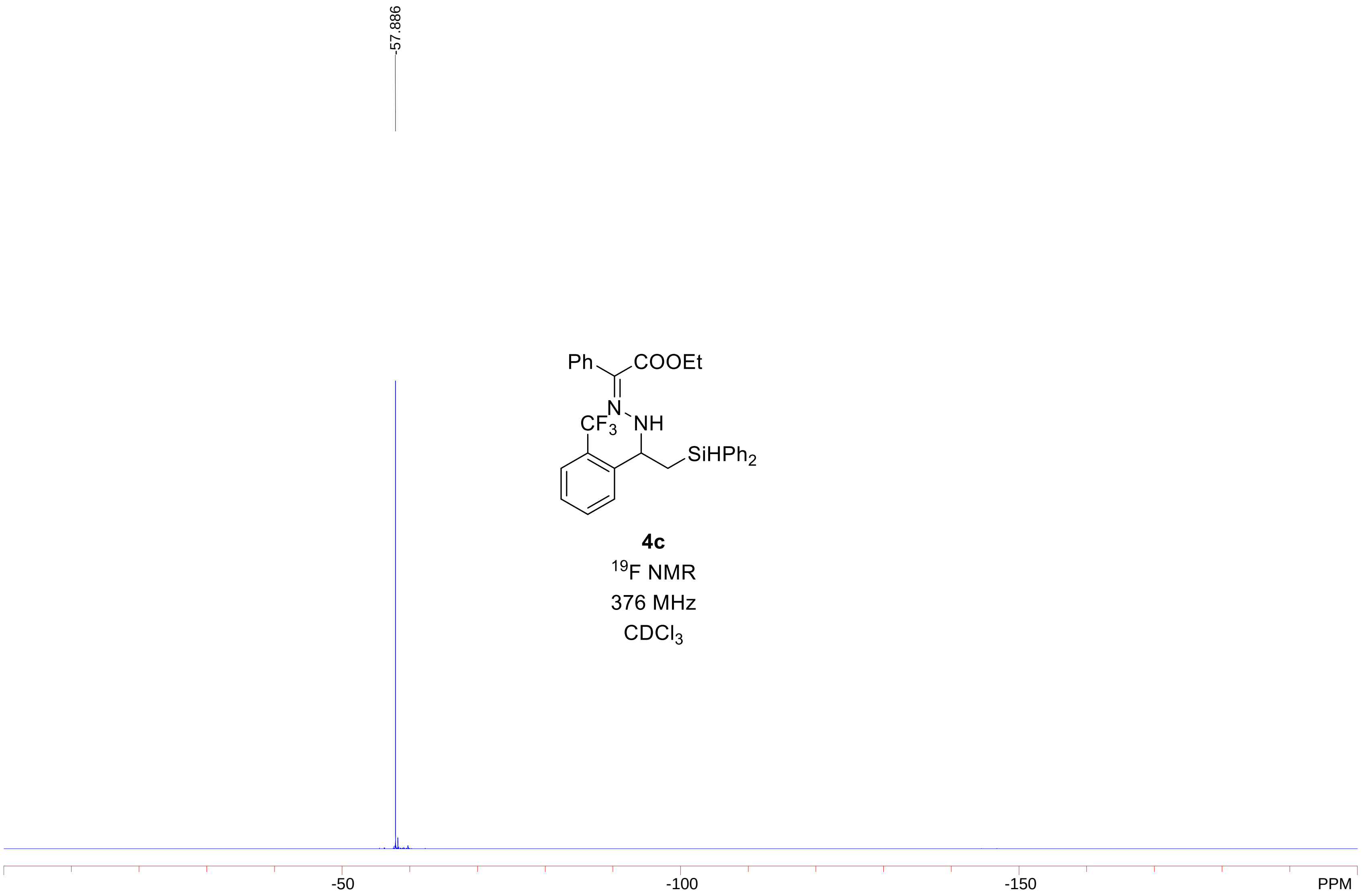

Supplementary Figure 37. ^{19}F NMR spectrum of **4c**.

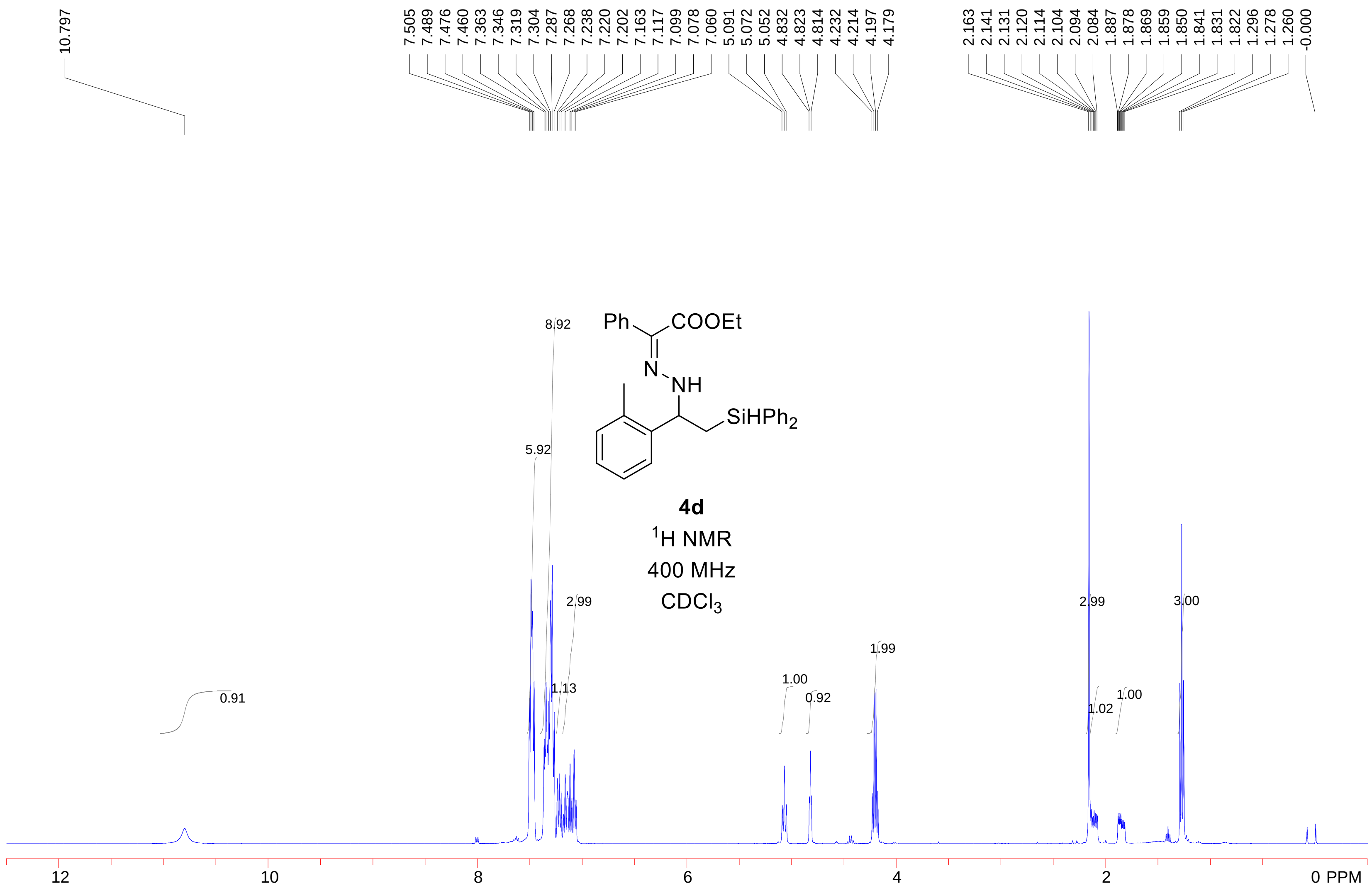

Supplementary Figure 38. ^{1}H NMR spectrum of **4d**.

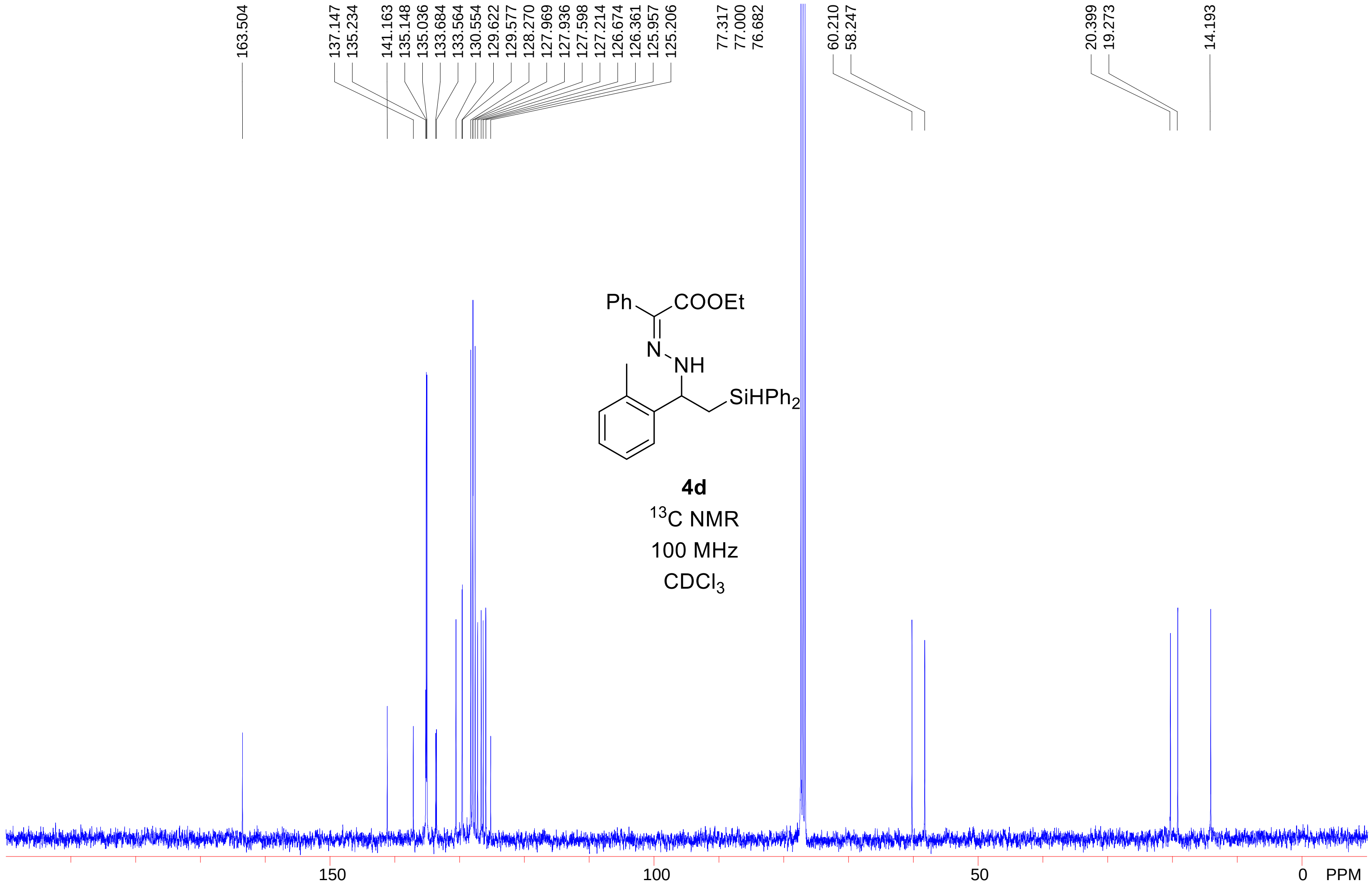

Supplementary Figure 39. ^{13}C NMR spectrum of **4d**.

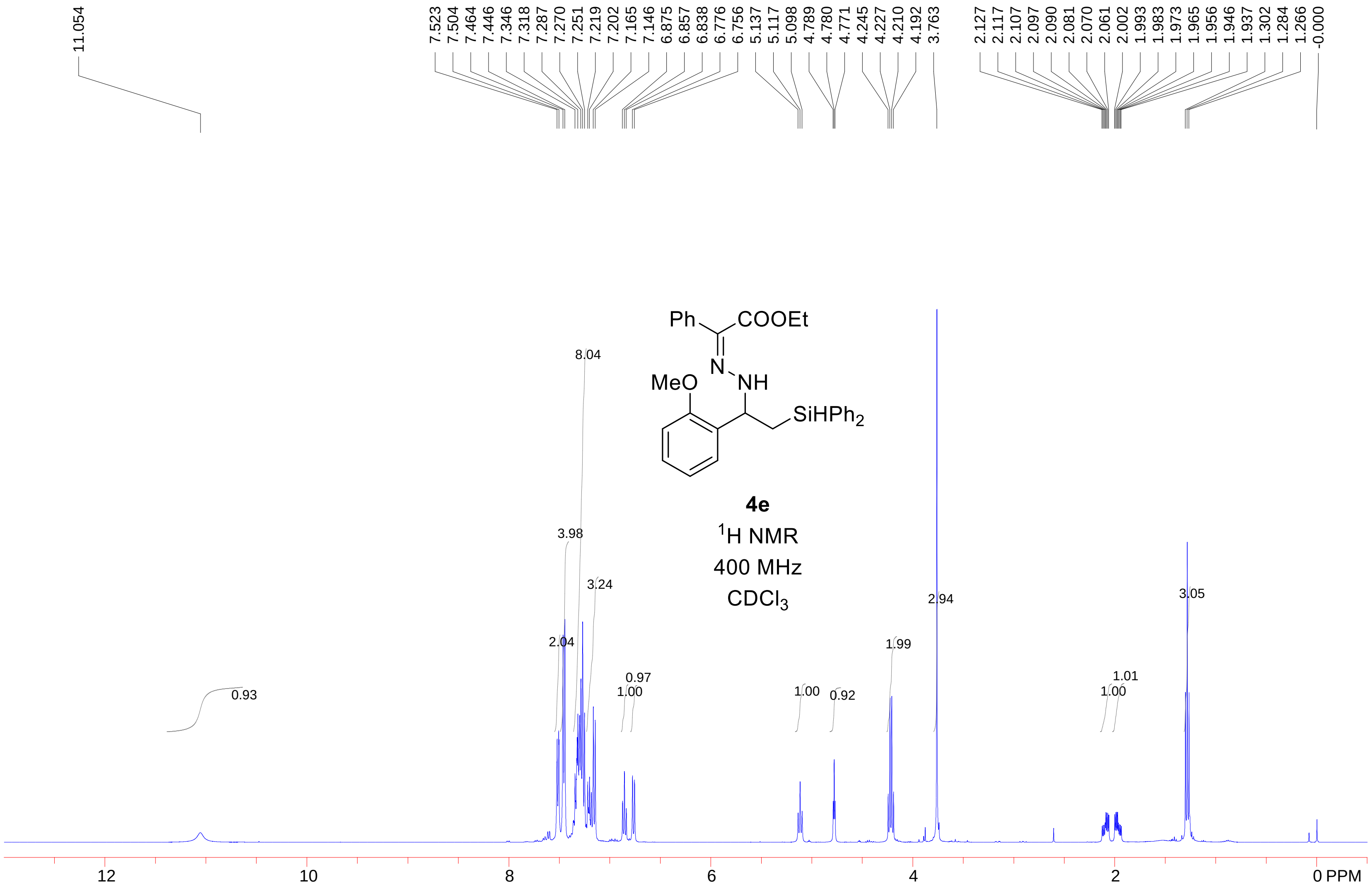

Supplementary Figure 40. ^{1}H NMR spectrum of **4e**.

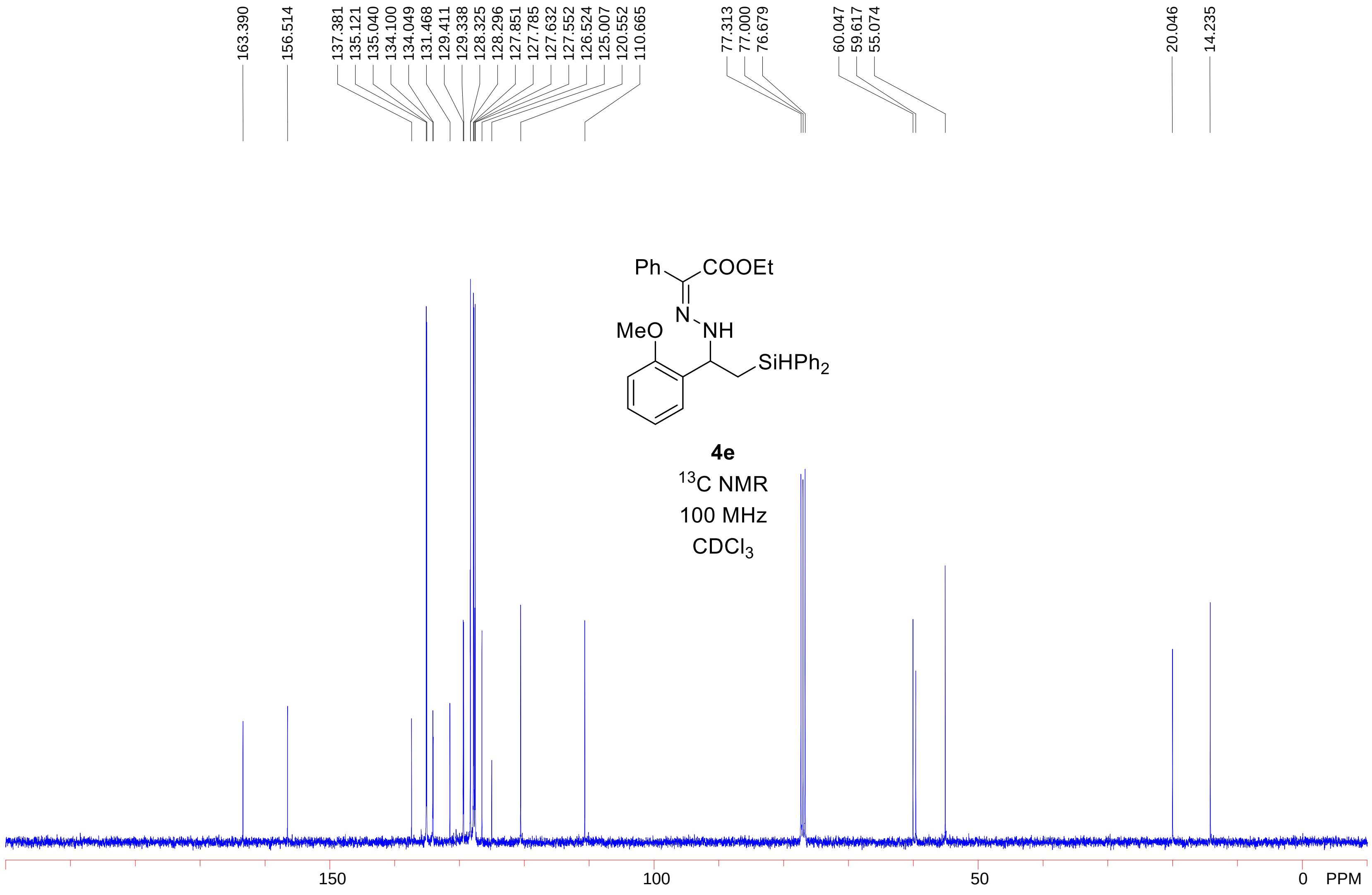

Supplementary Figure 41. ^{13}C NMR spectrum of **4e**.

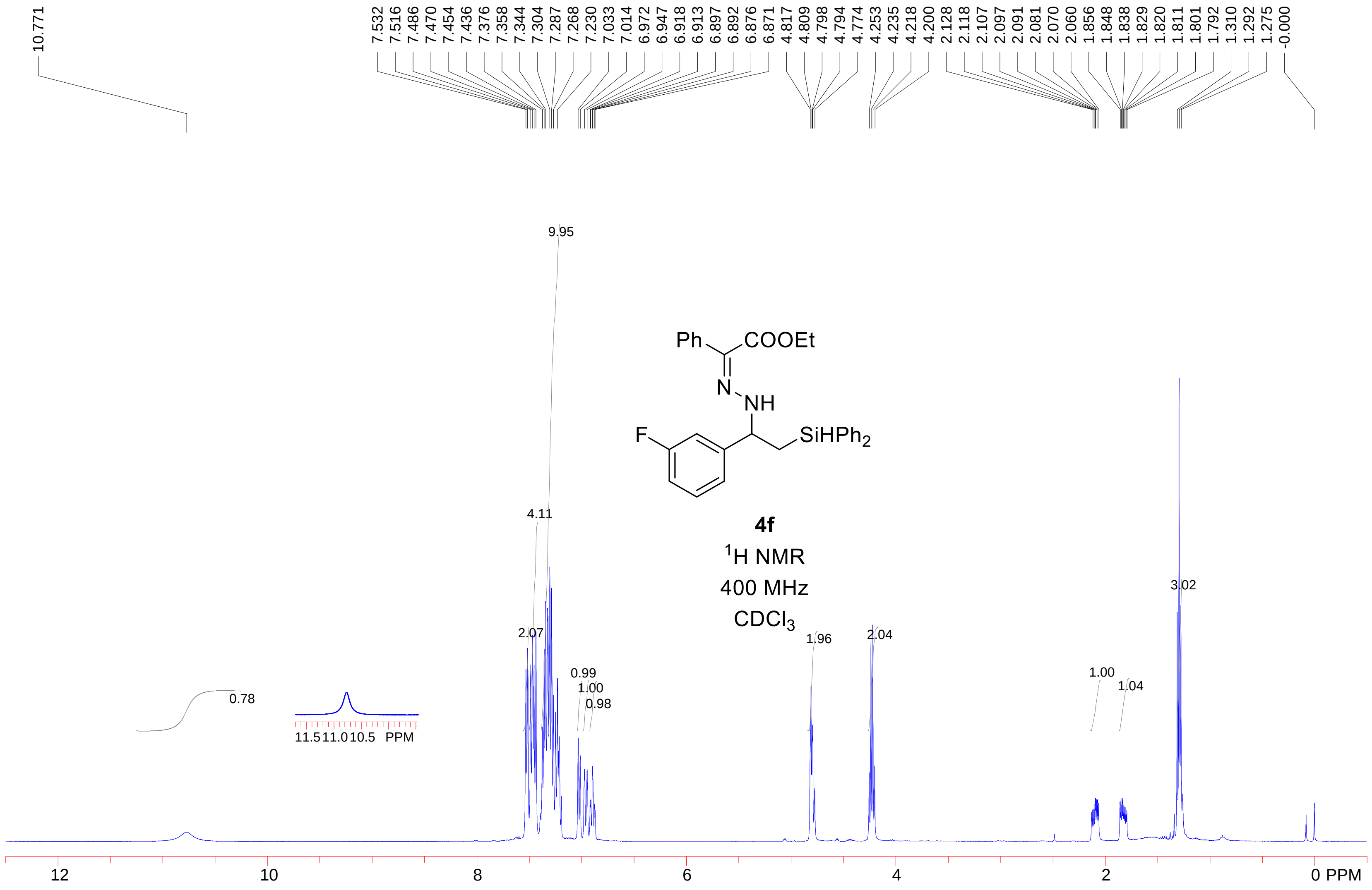

Supplementary Figure 42. ^{1}H NMR spectrum of **4f**.

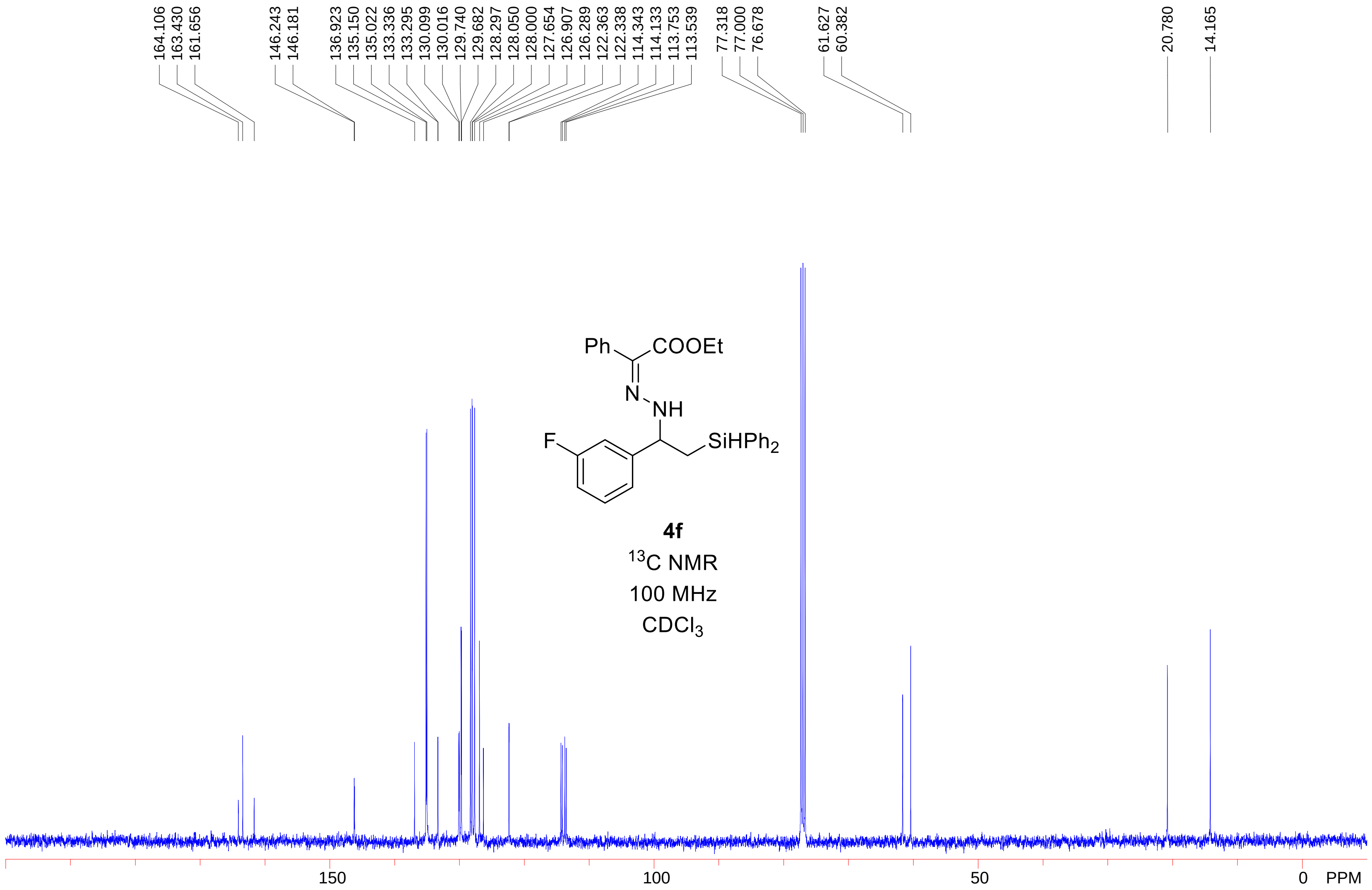

Supplementary Figure 43. ^{13}C NMR spectrum of **4f**.

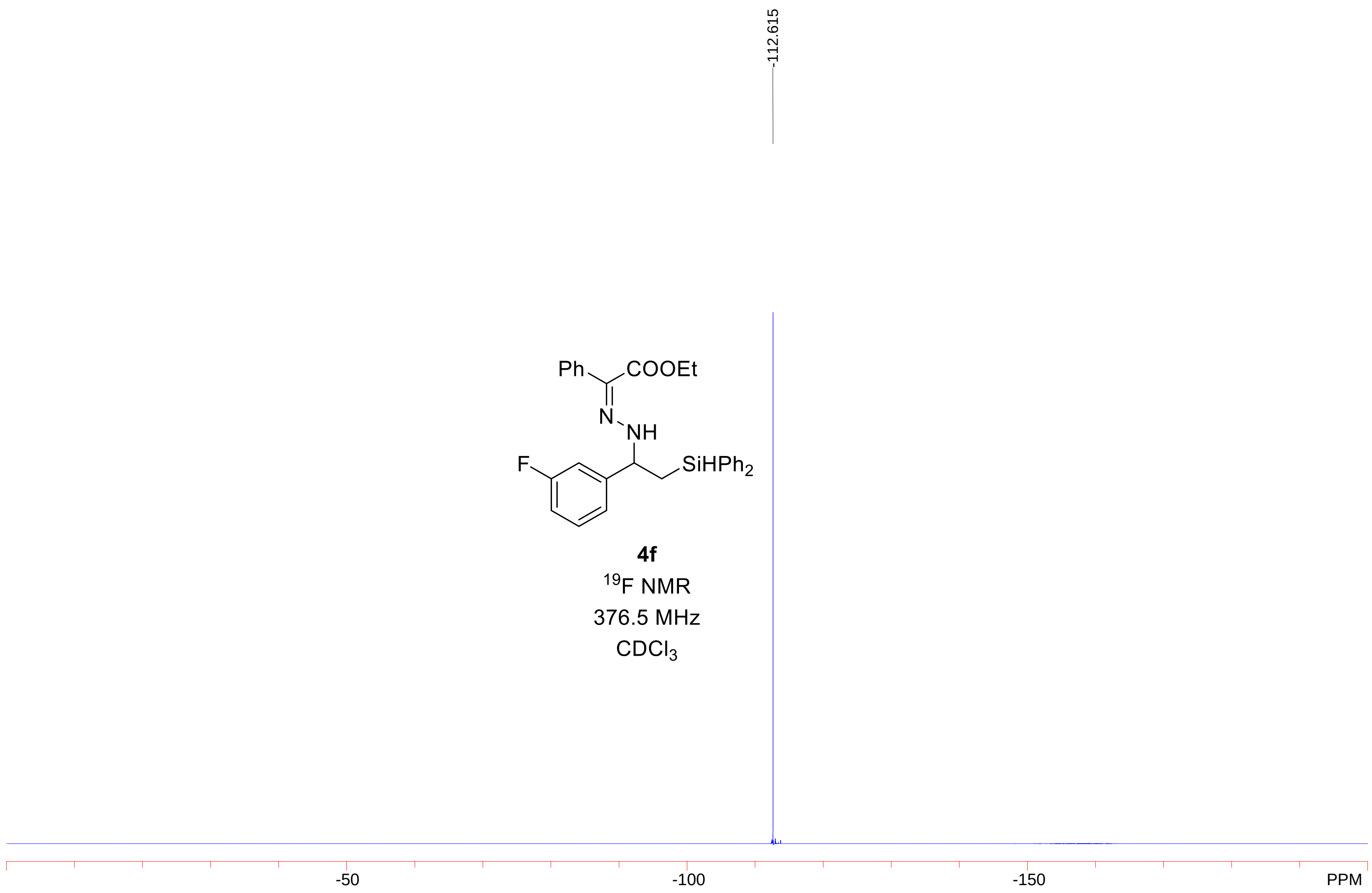

Supplementary Figure 44. ^{19}F NMR spectrum of **4f**.

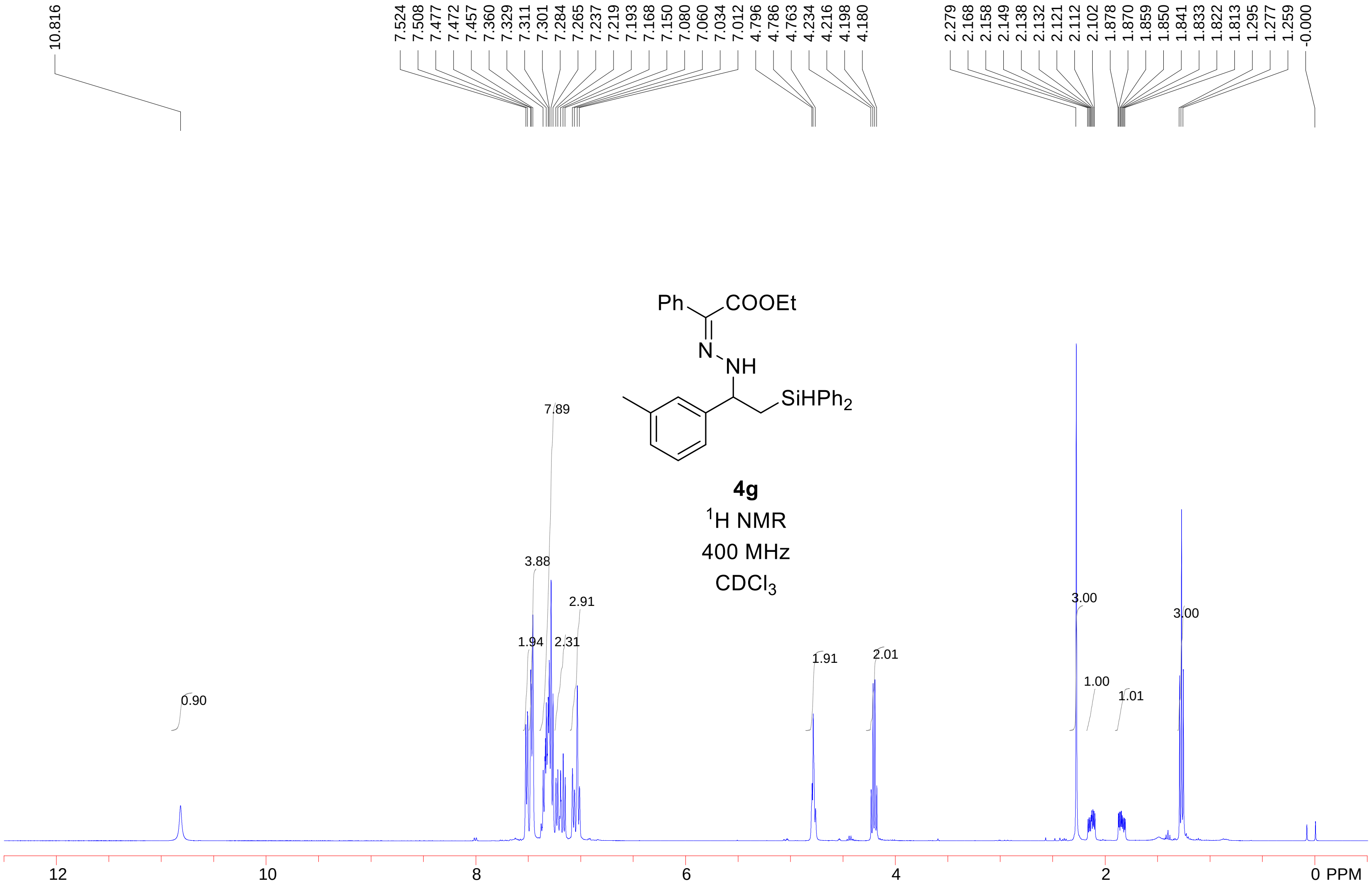

Supplementary Figure 45. ^{1}H NMR spectrum of **4g**.

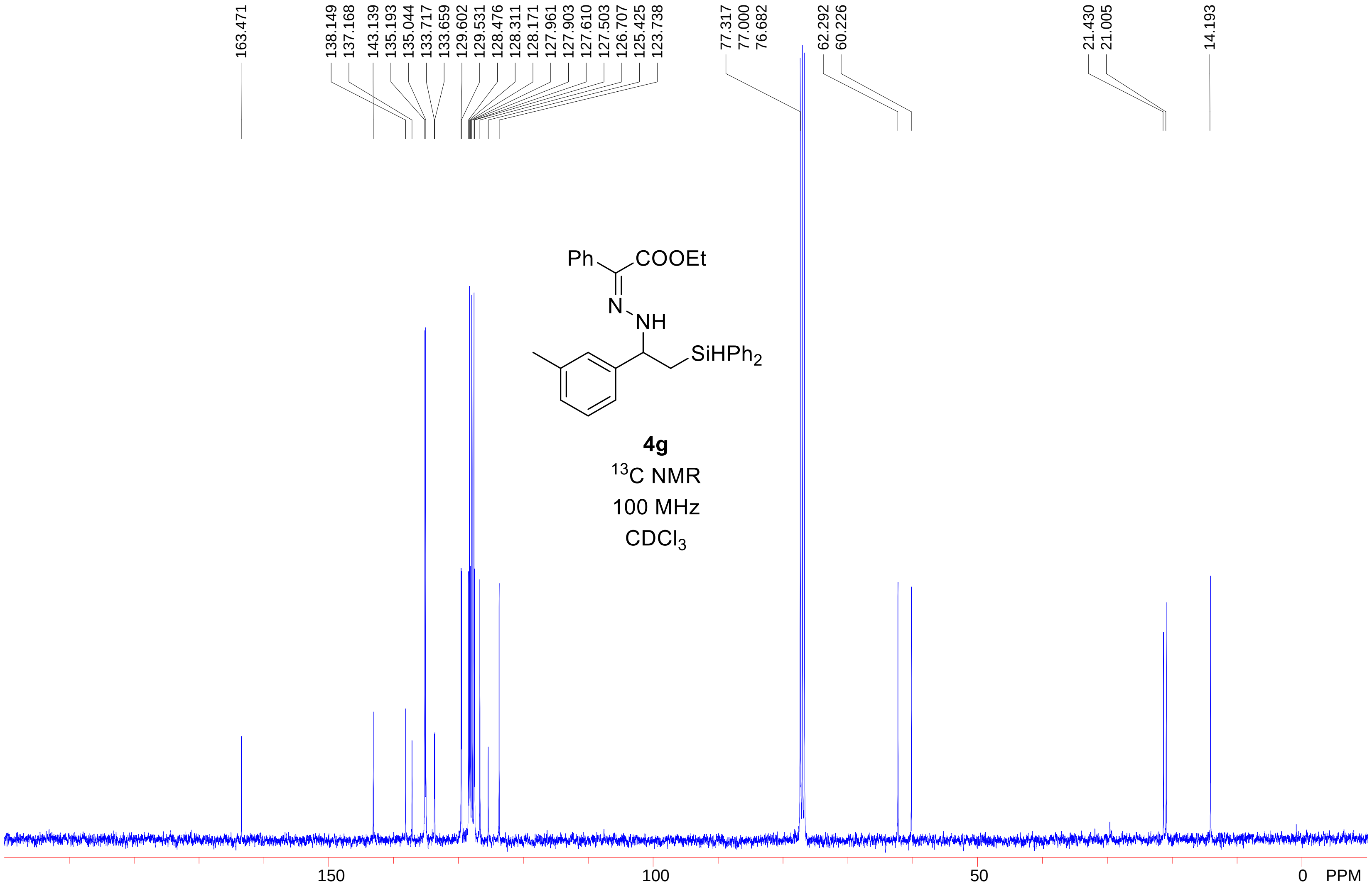

Supplementary Figure 46. ^{13}C NMR spectrum of **4g**.

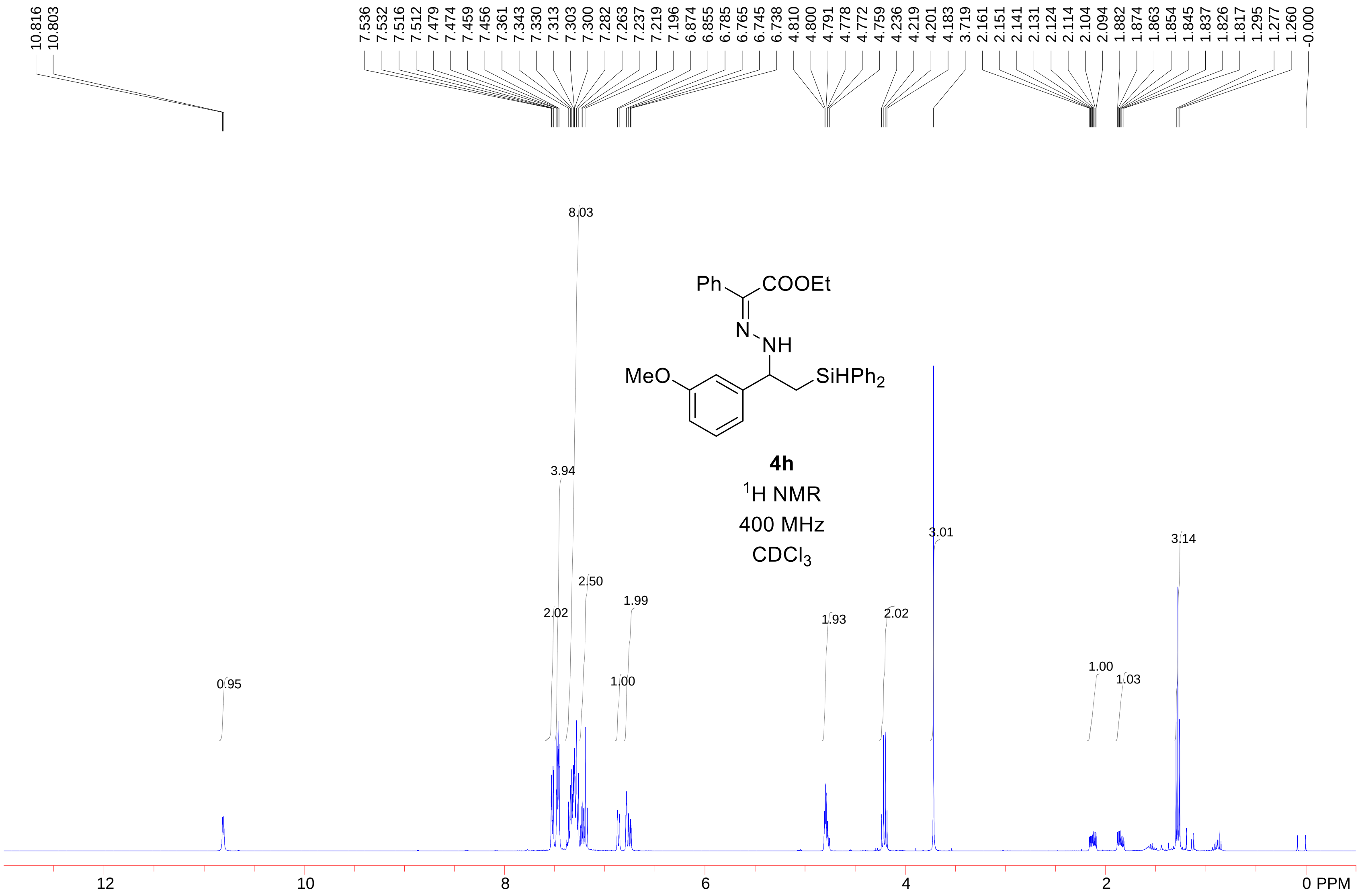

Supplementary Figure 47. ^{1}H NMR spectrum of **4h**.

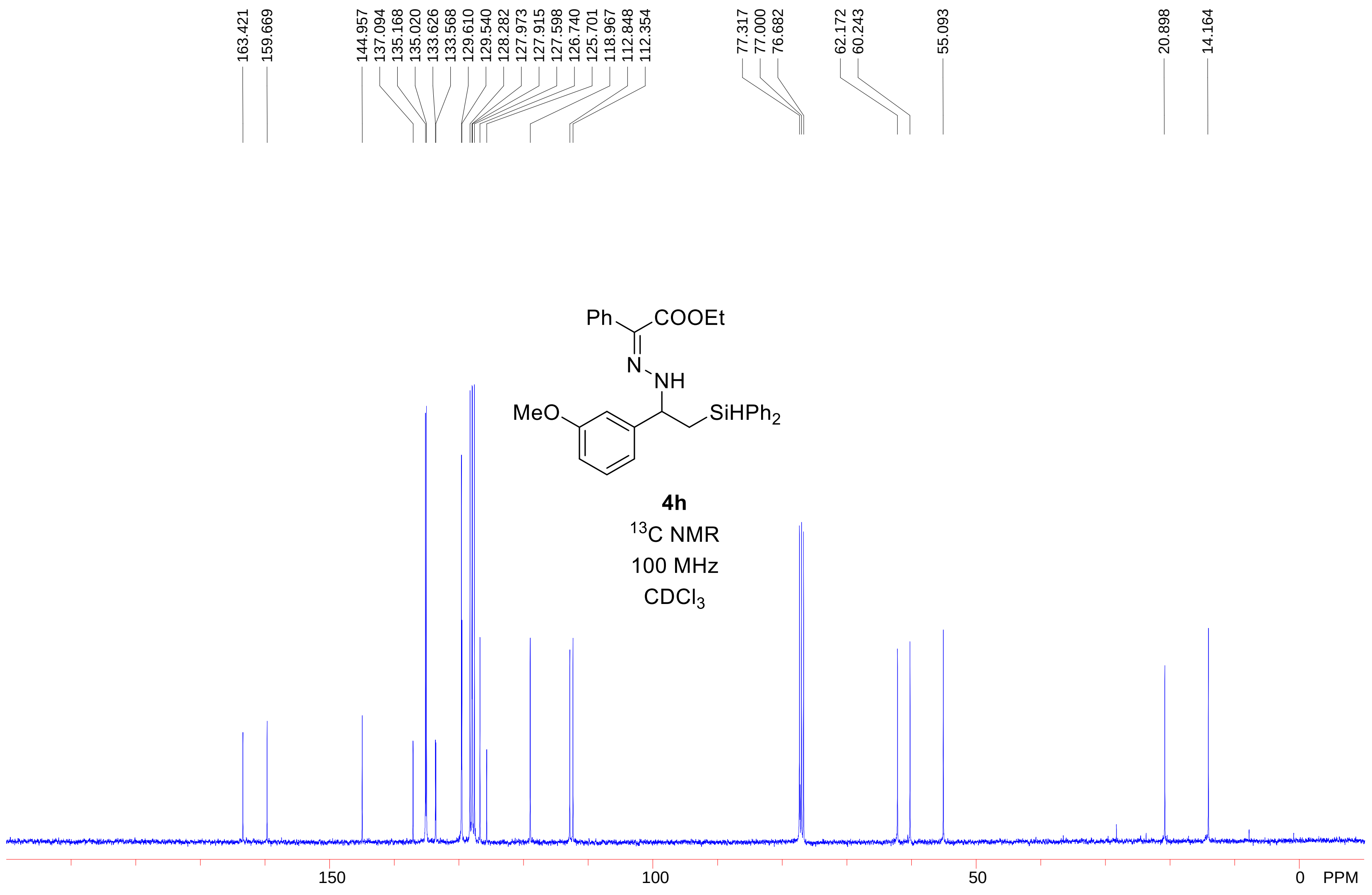

Supplementary Figure 48. ^{13}C NMR spectrum of **4h**.

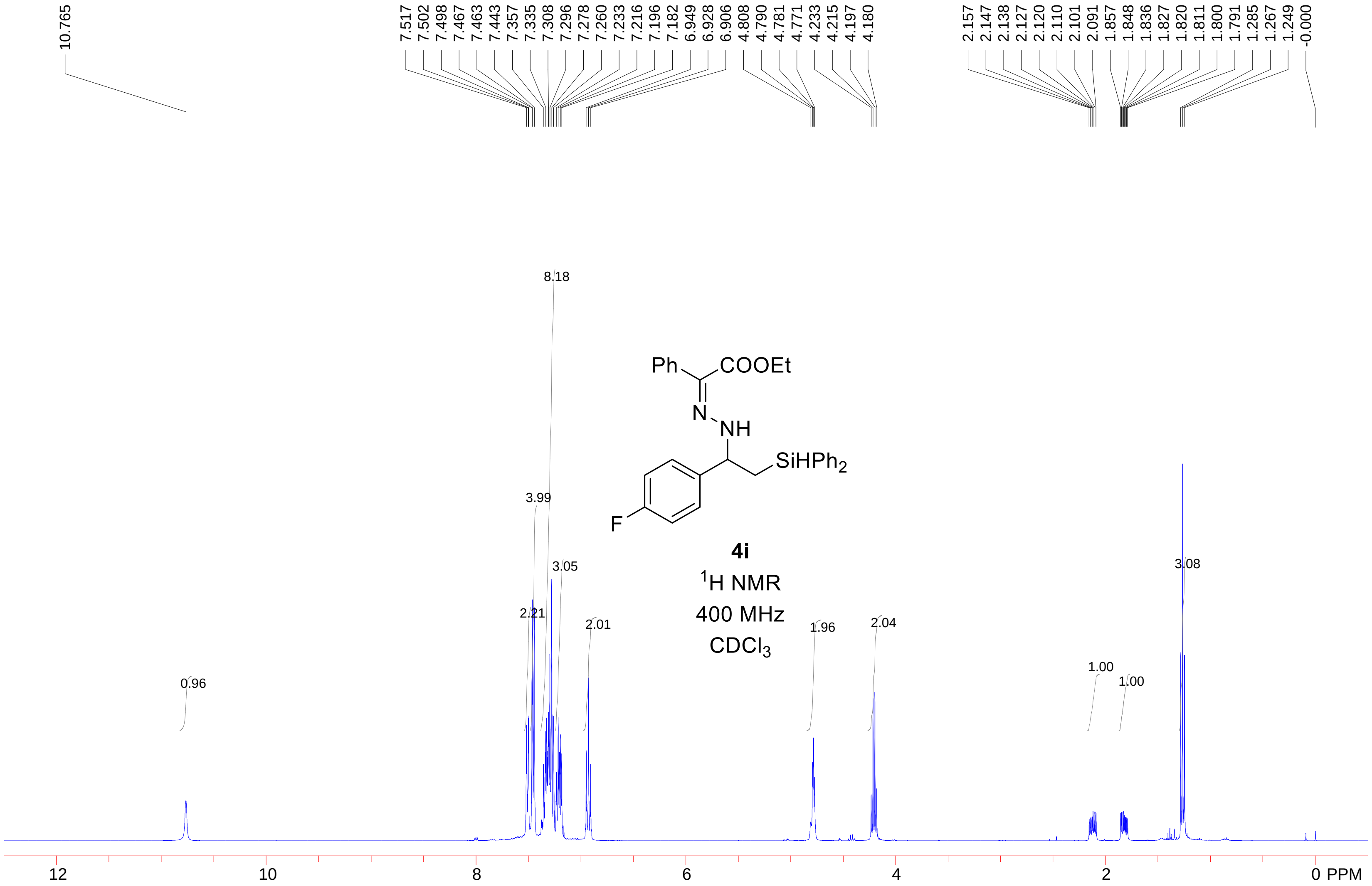

Supplementary Figure 49. ^{1}H NMR spectrum of **4i**.

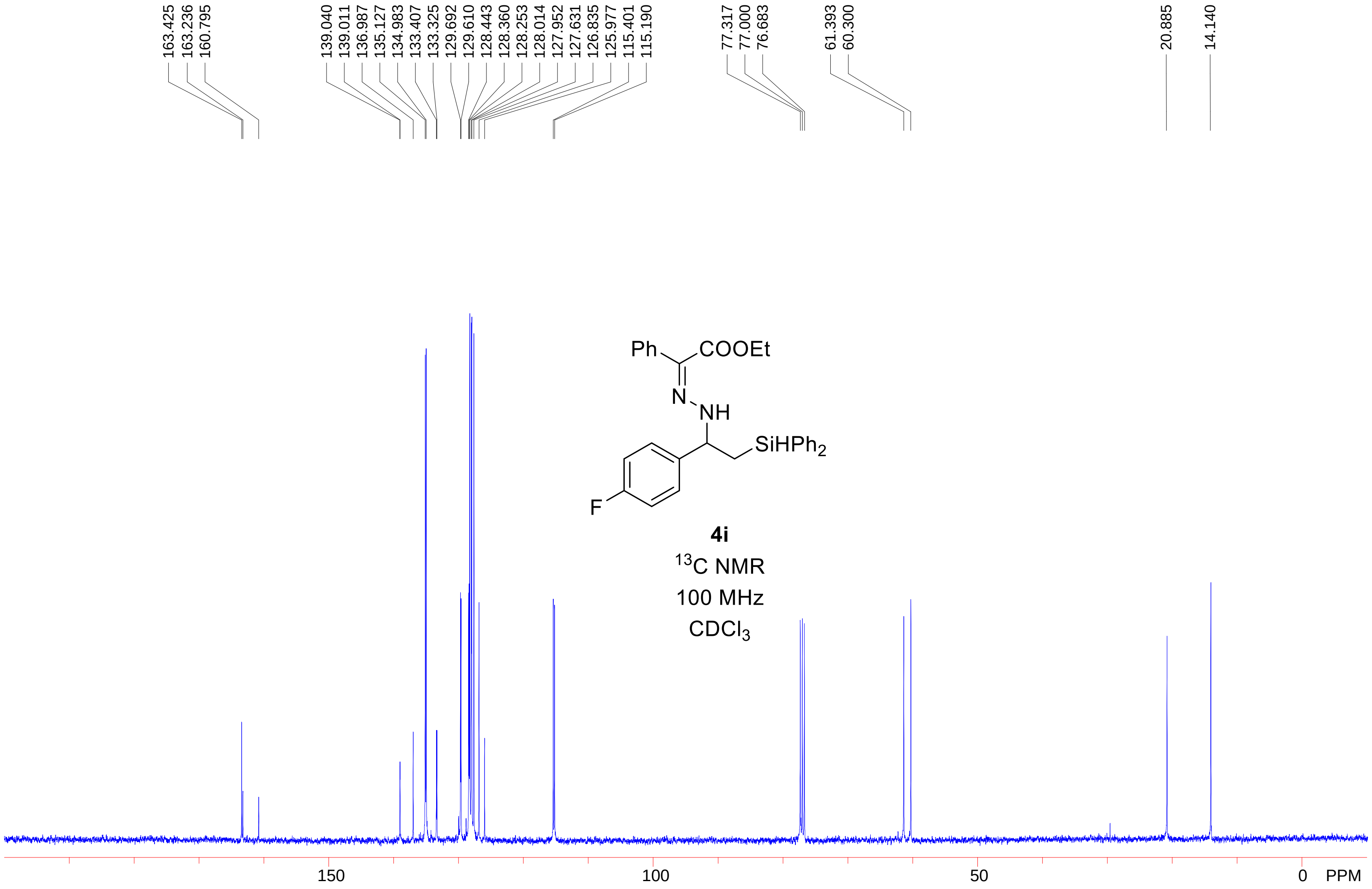

Supplementary Figure 50. ^{13}C NMR spectrum of **4i**.

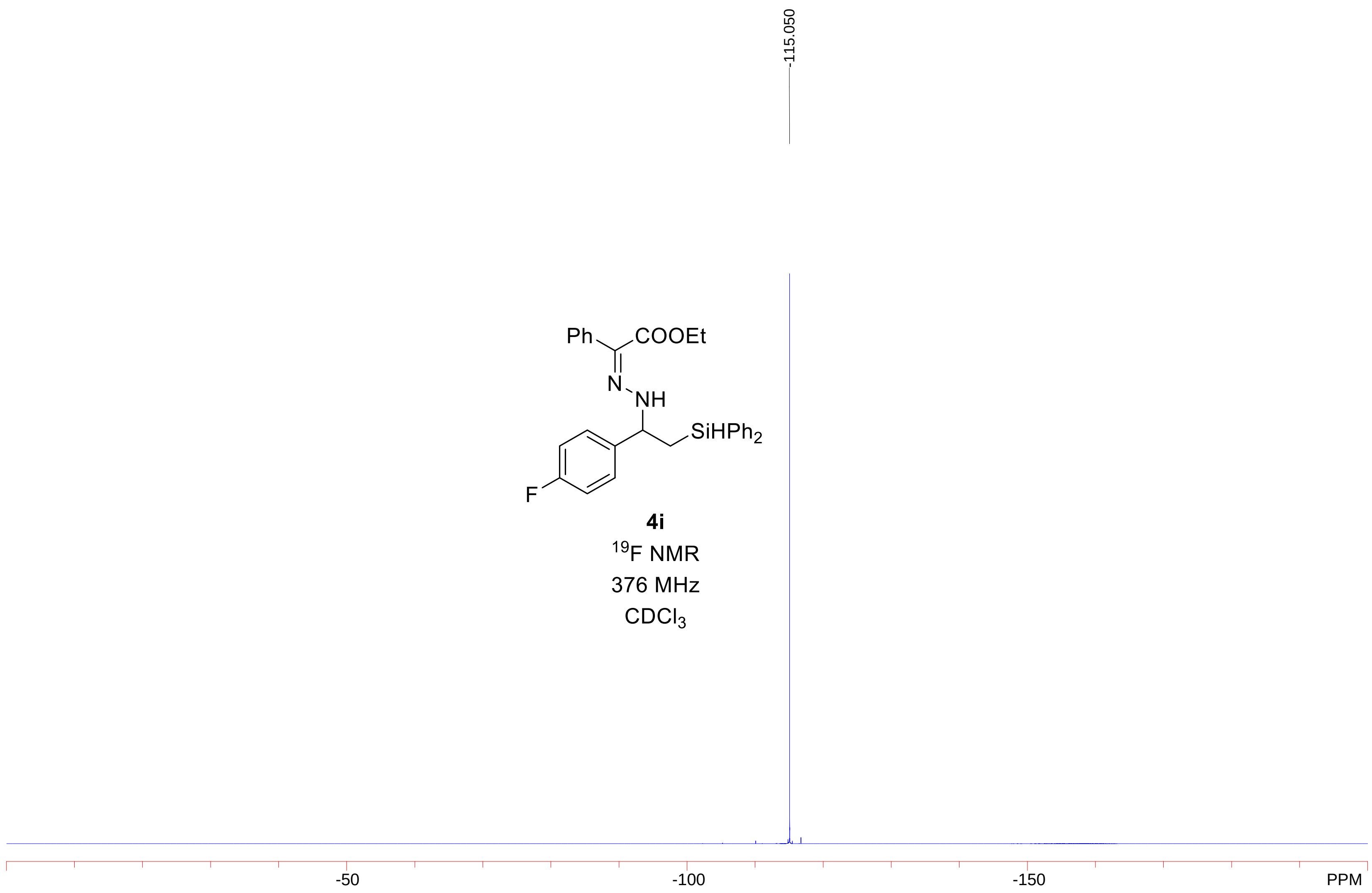

Supplementary Figure 51. ^{19}F NMR spectrum of **4i**.

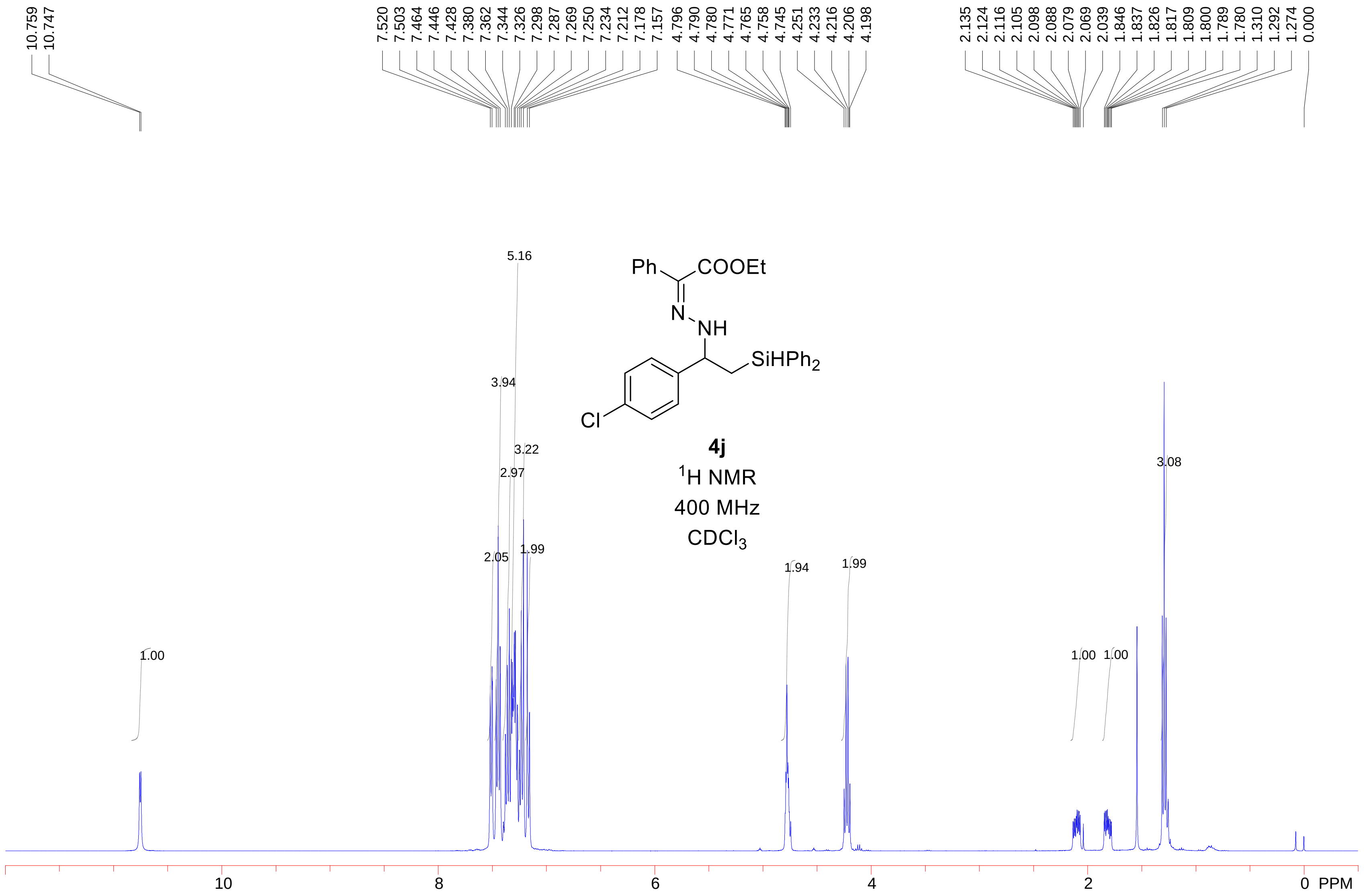

Supplementary Figure 52. ^{1}H NMR spectrum of **4j**.

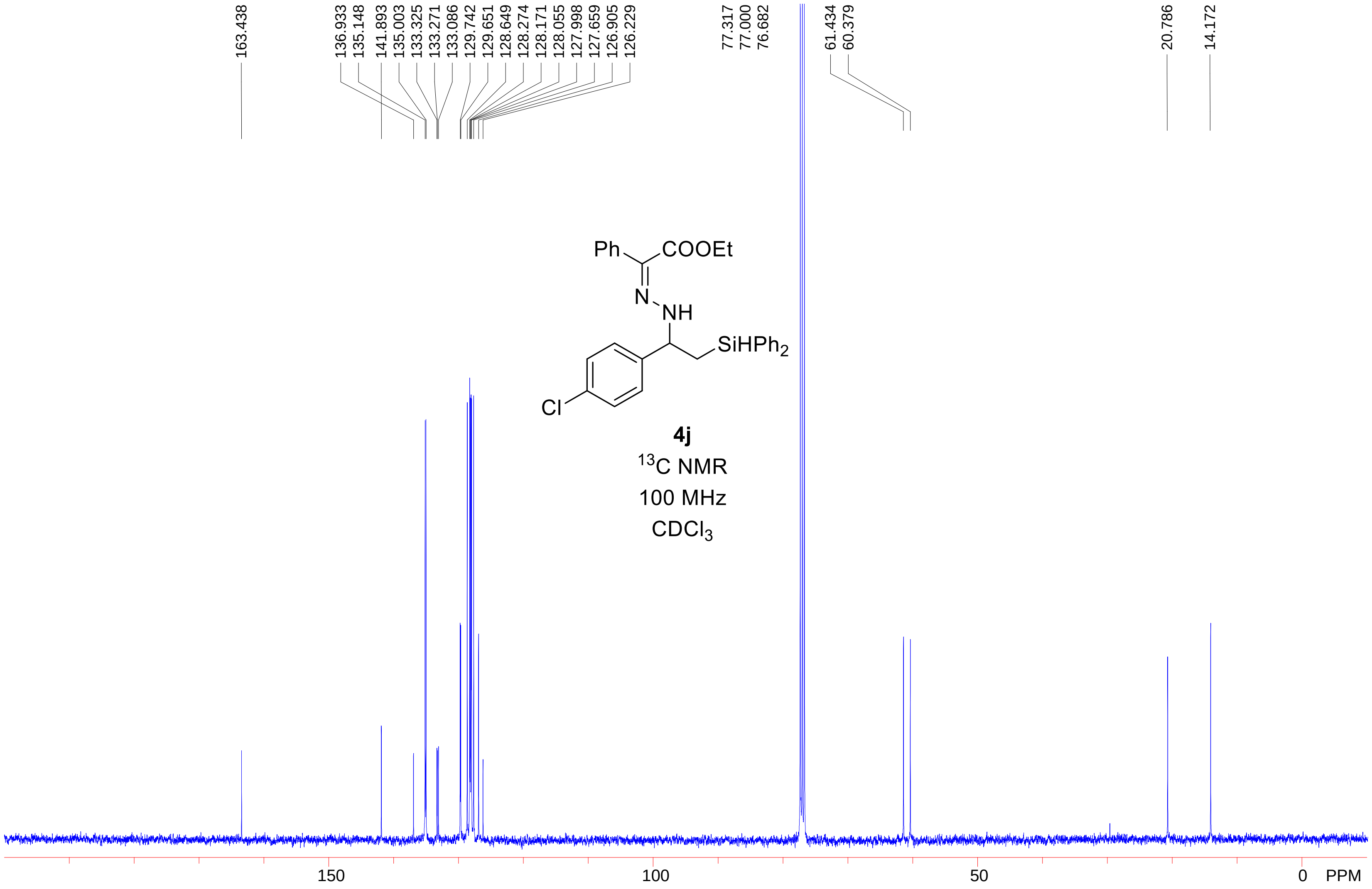

Supplementary Figure 53. ^{13}C NMR spectrum of **4j**.

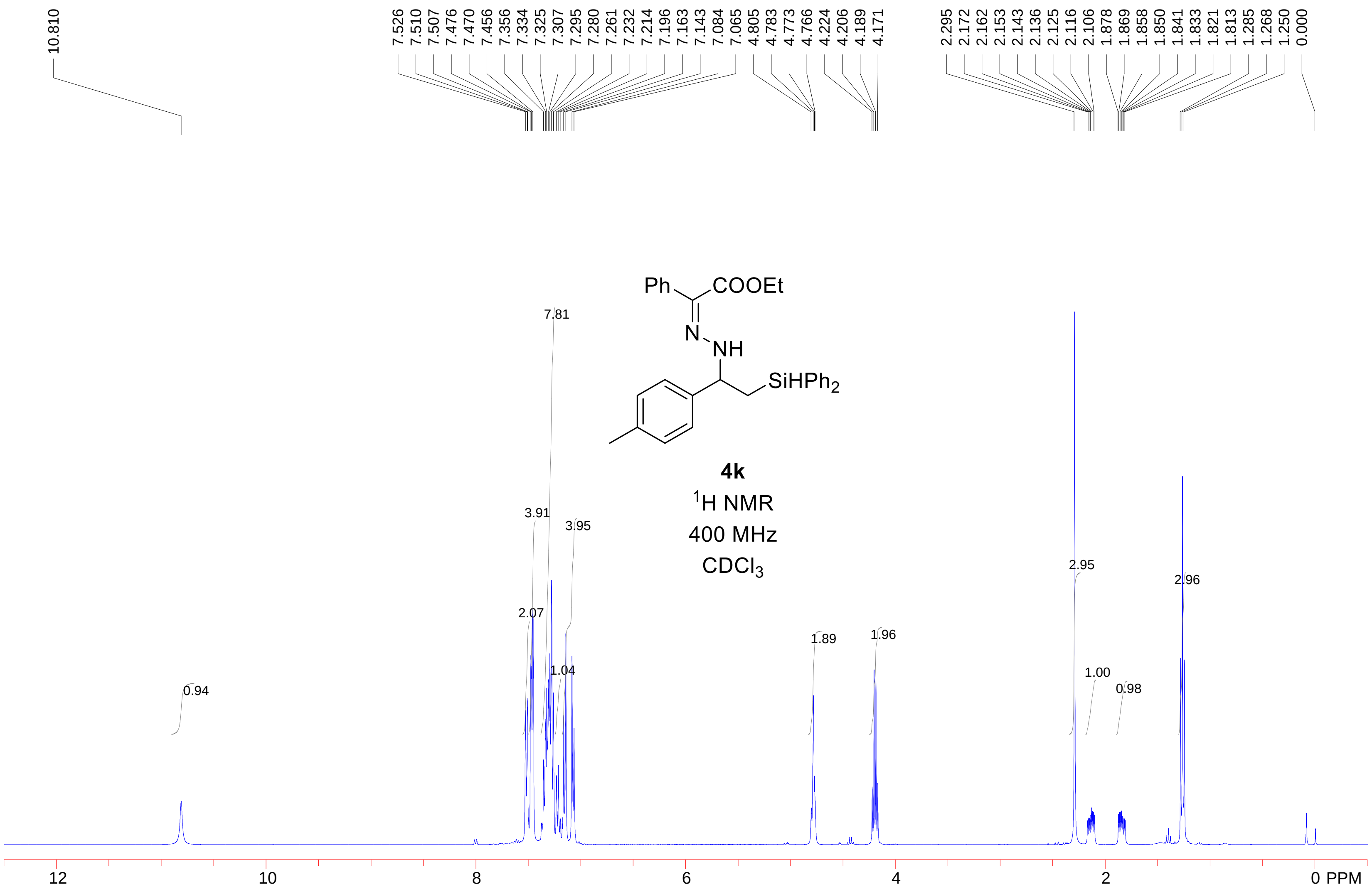

Supplementary Figure 54. ^{1}H NMR spectrum of **4k**.

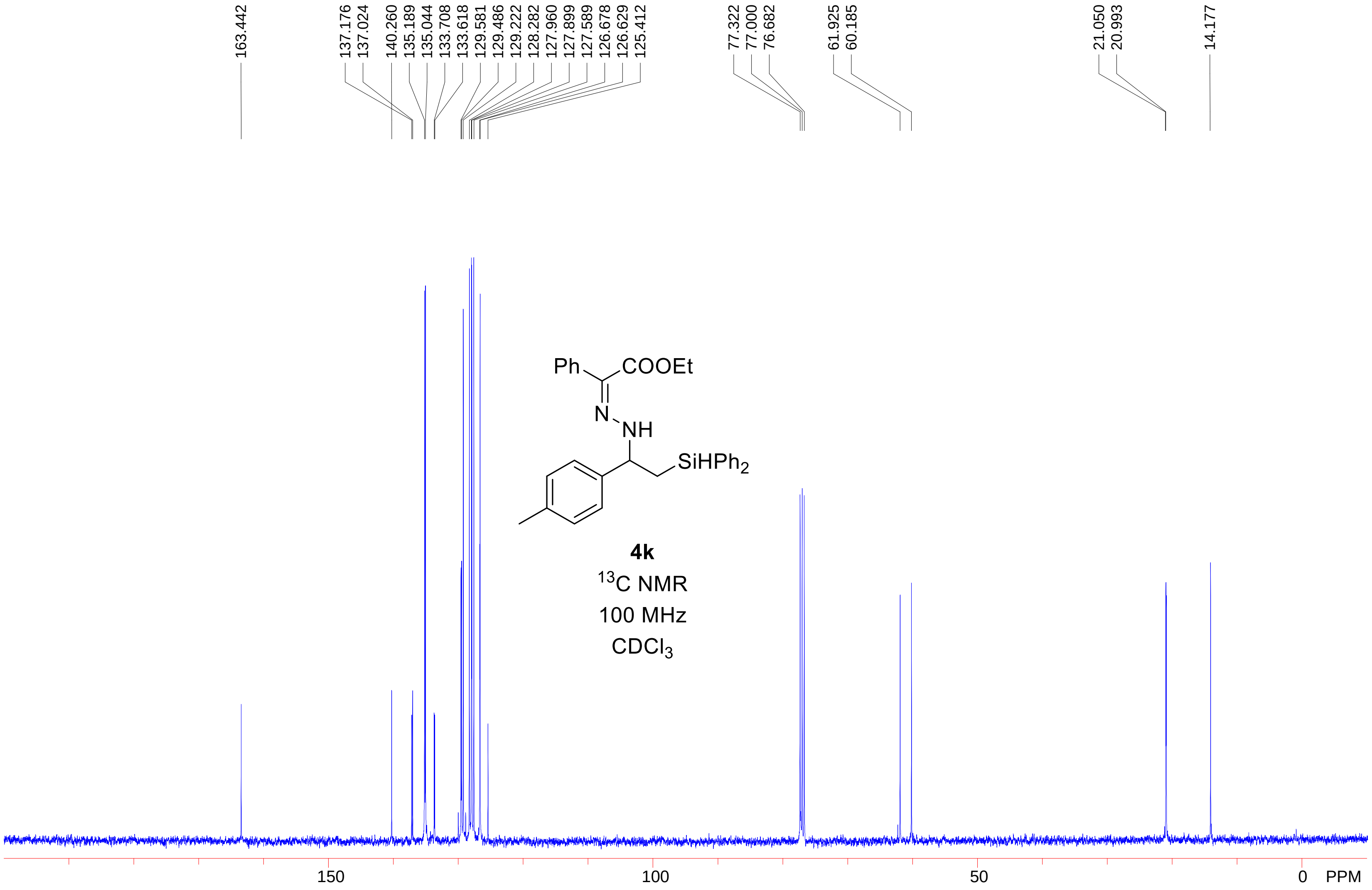

Supplementary Figure 55. ^{13}C NMR spectrum of **4k**.

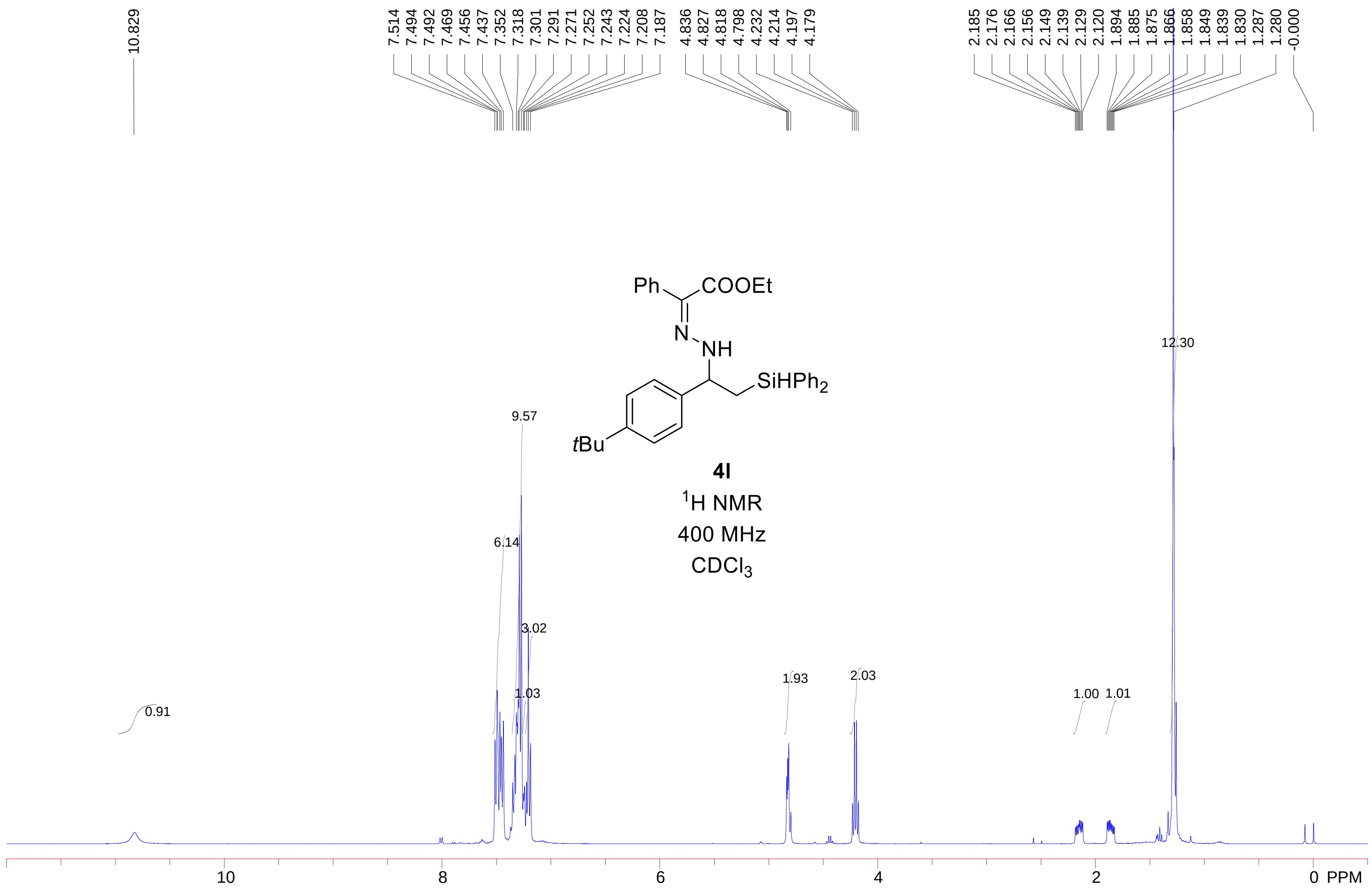

Supplementary Figure 56. ^{1}H NMR spectrum of **4l**.

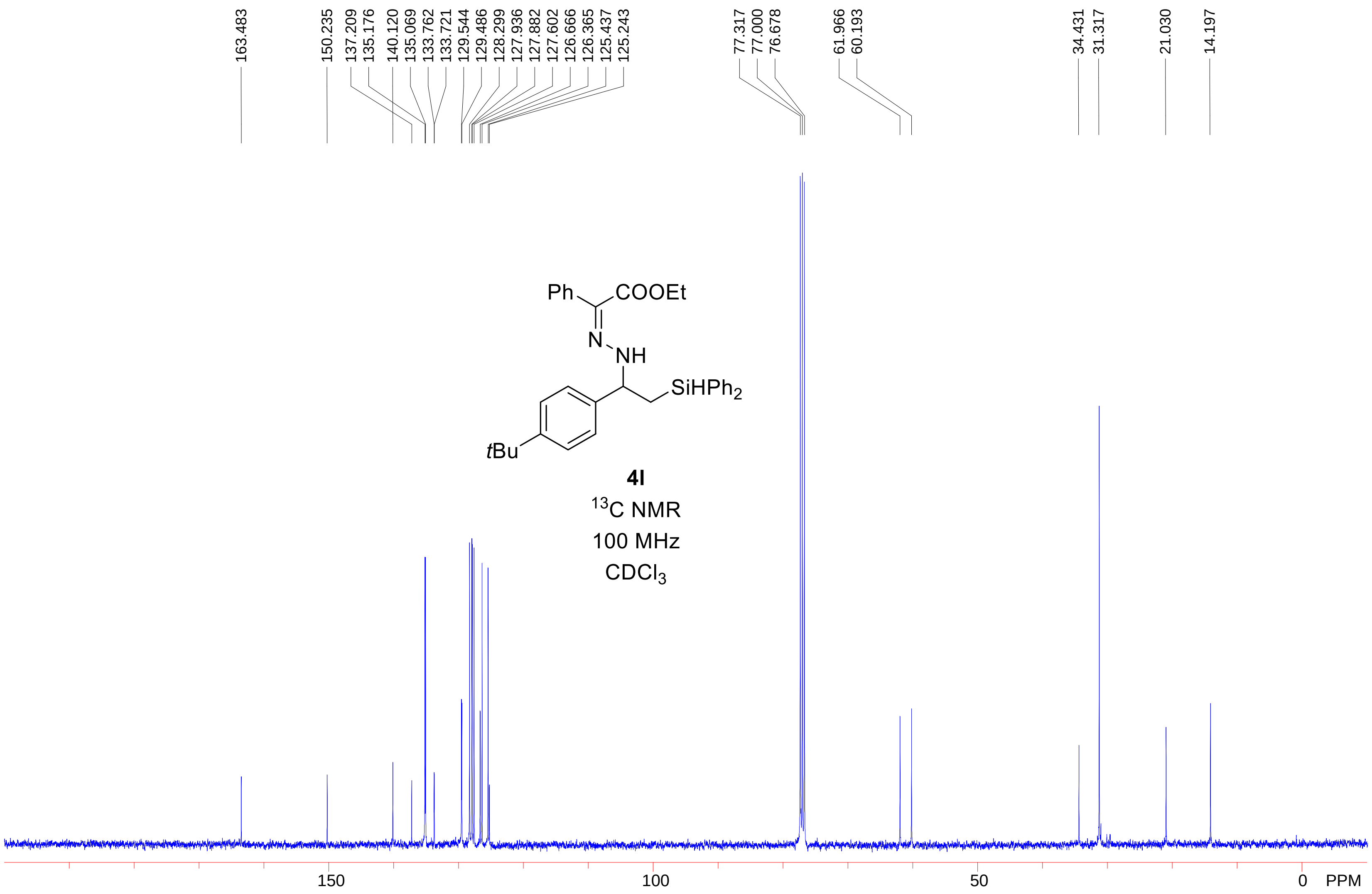

Supplementary Figure 57. ^{13}C NMR spectrum of **4l**.

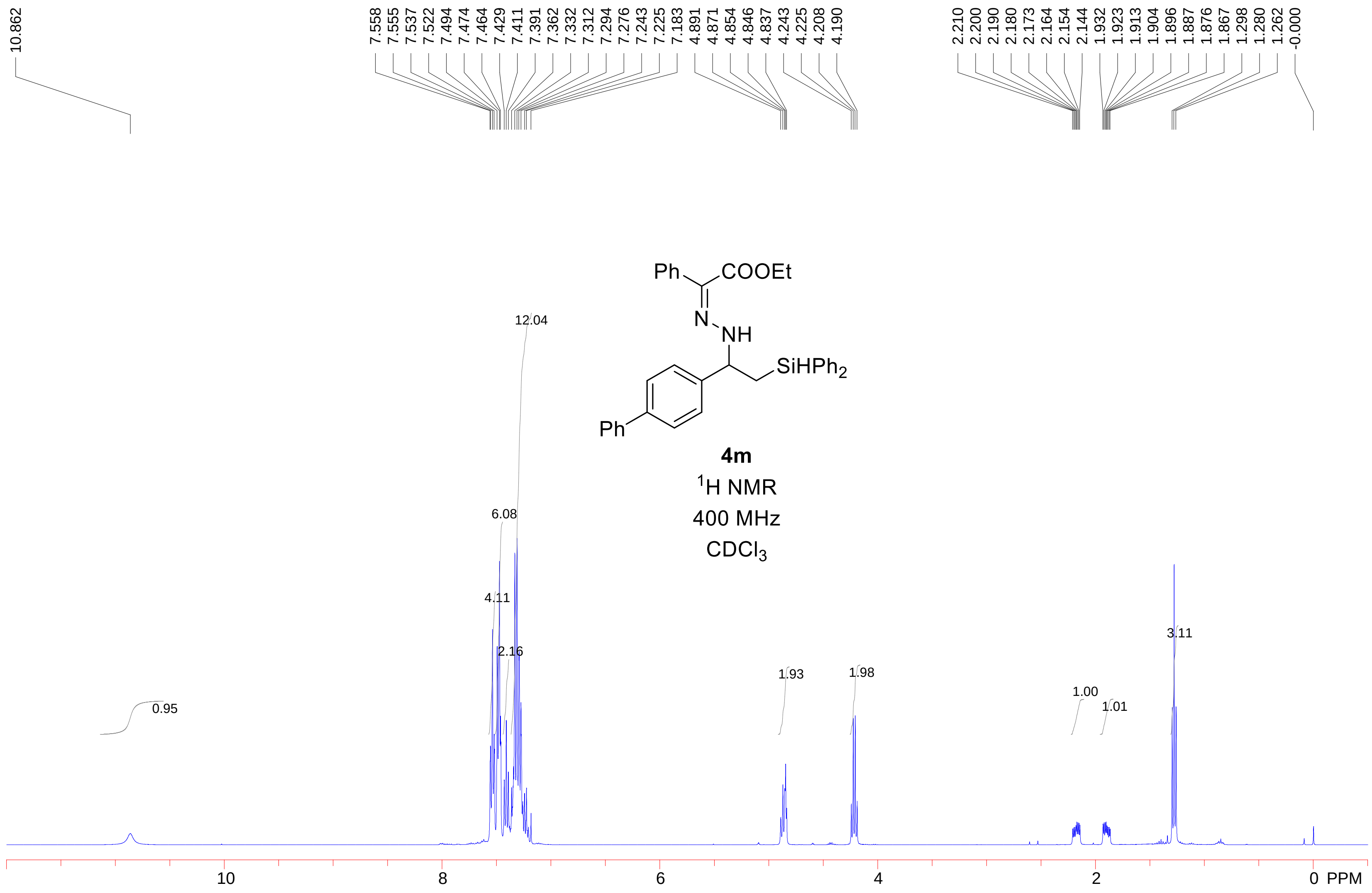

Supplementary Figure 58. ^{1}H NMR spectrum of **4m**.

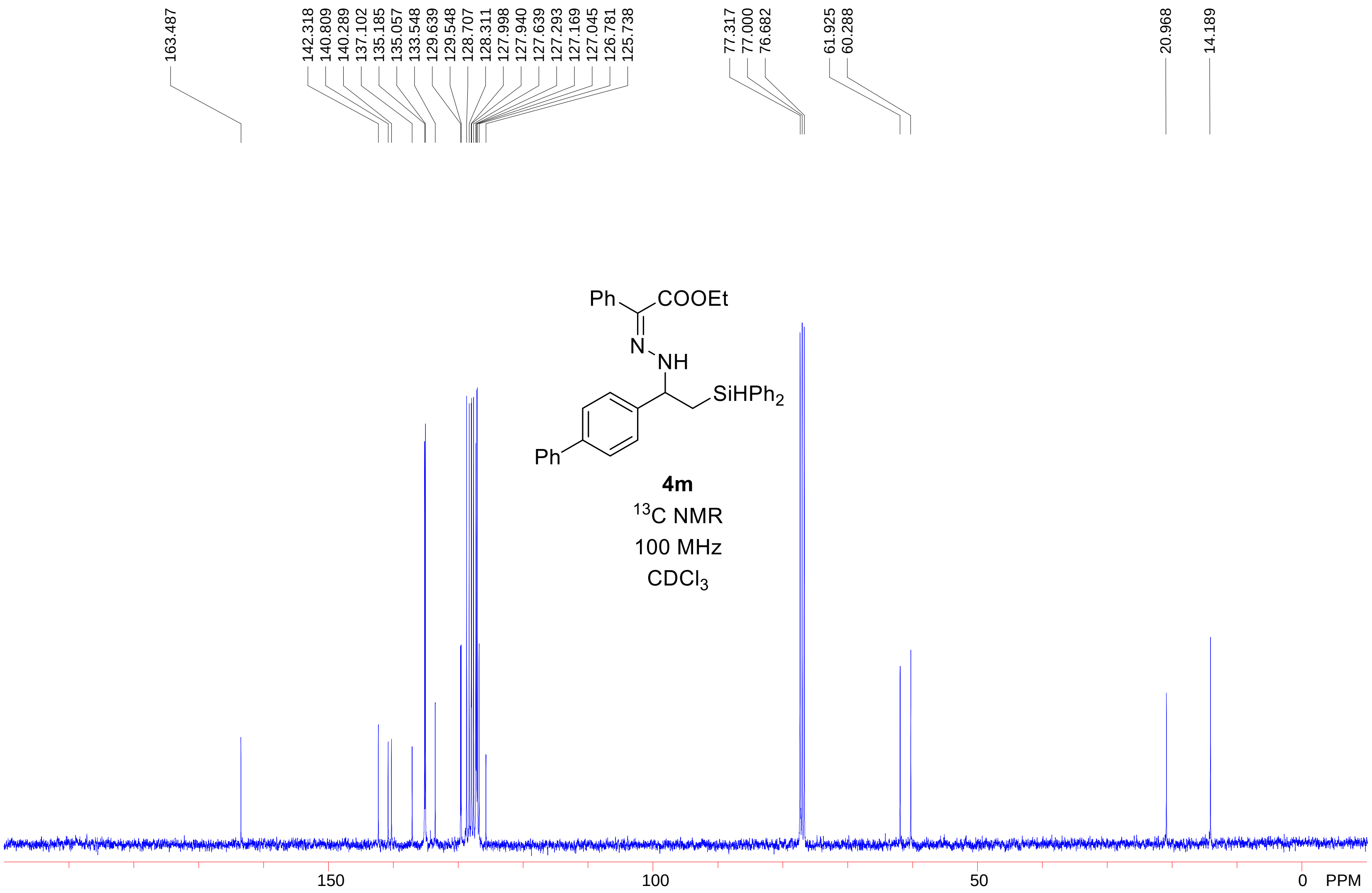

Supplementary Figure 59. ^{13}C NMR spectrum of **4m**.

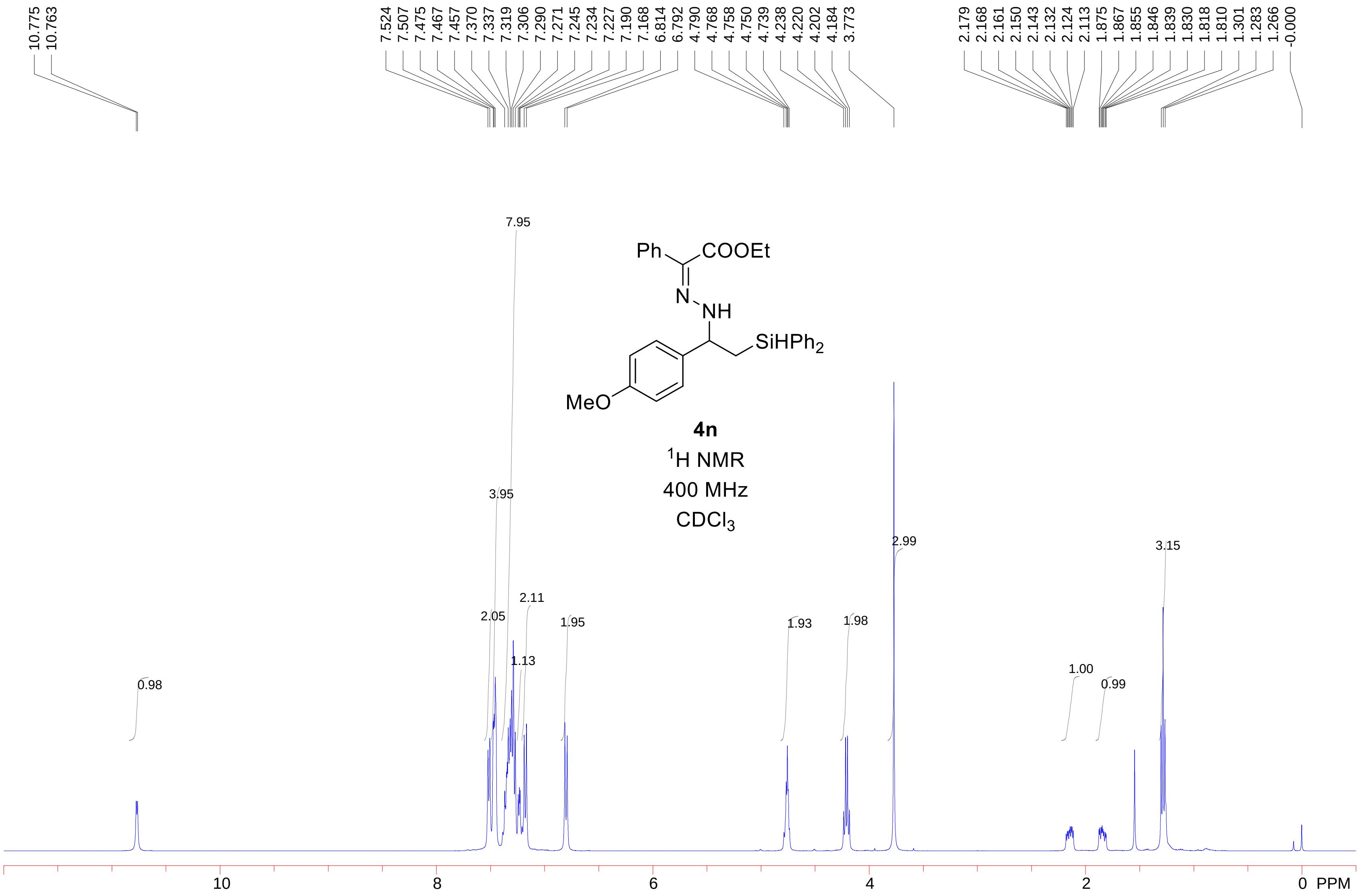

Supplementary Figure 60. ^{1}H NMR spectrum of **4n**.

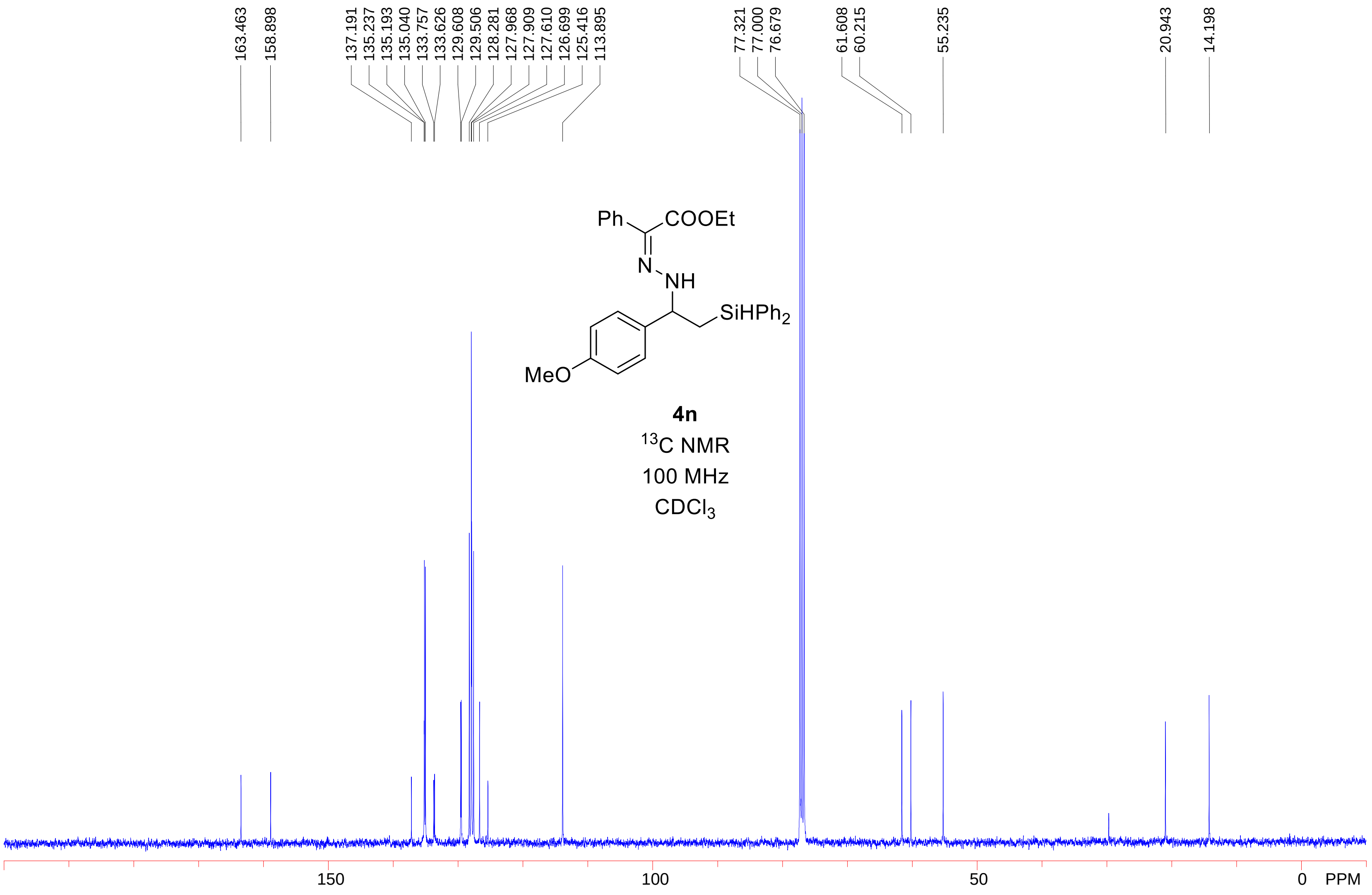

Supplementary Figure 61. ^{13}C NMR spectrum of **4n**.

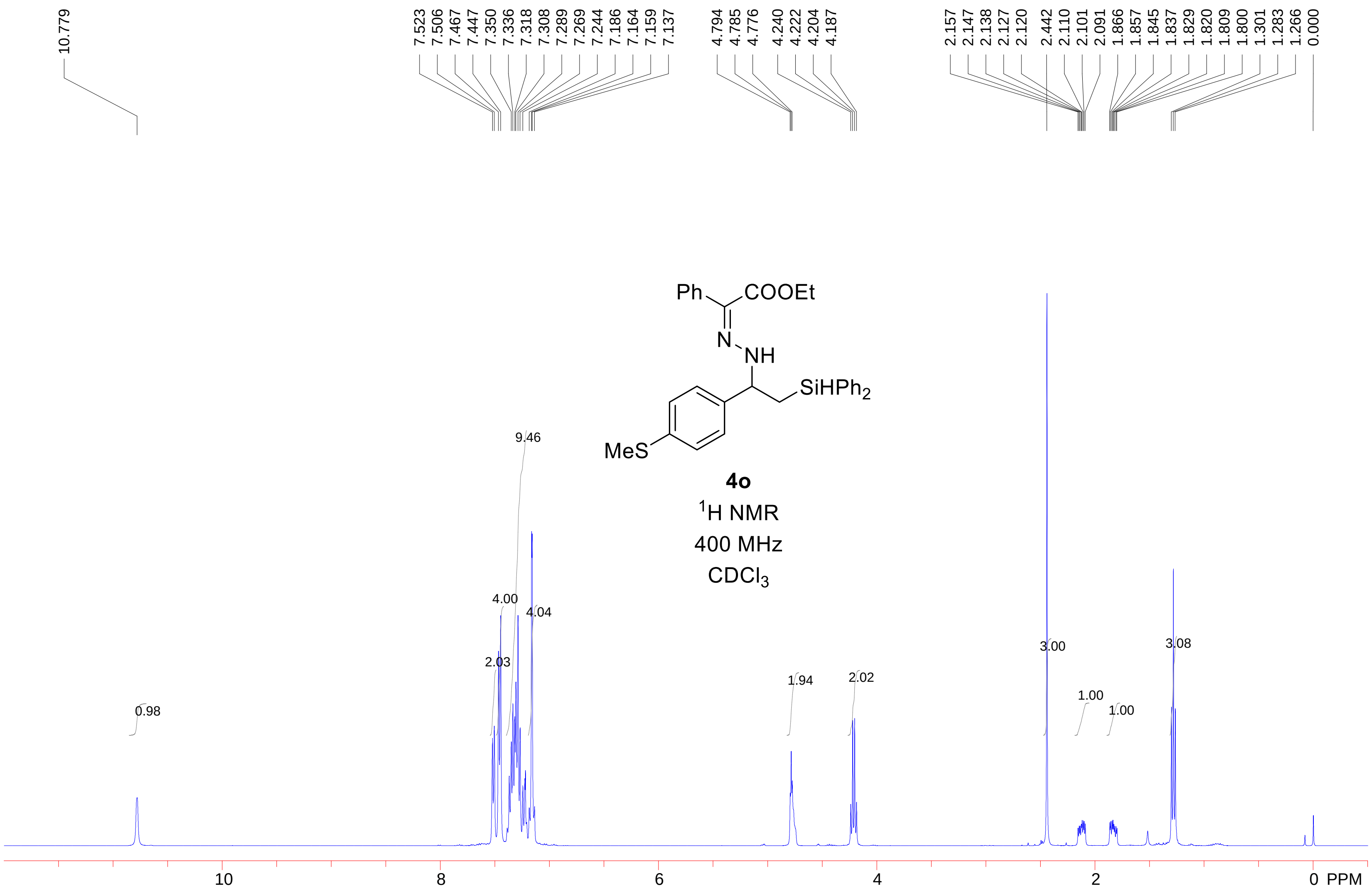

Supplementary Figure 62. ^{1}H NMR spectrum of **4o**.

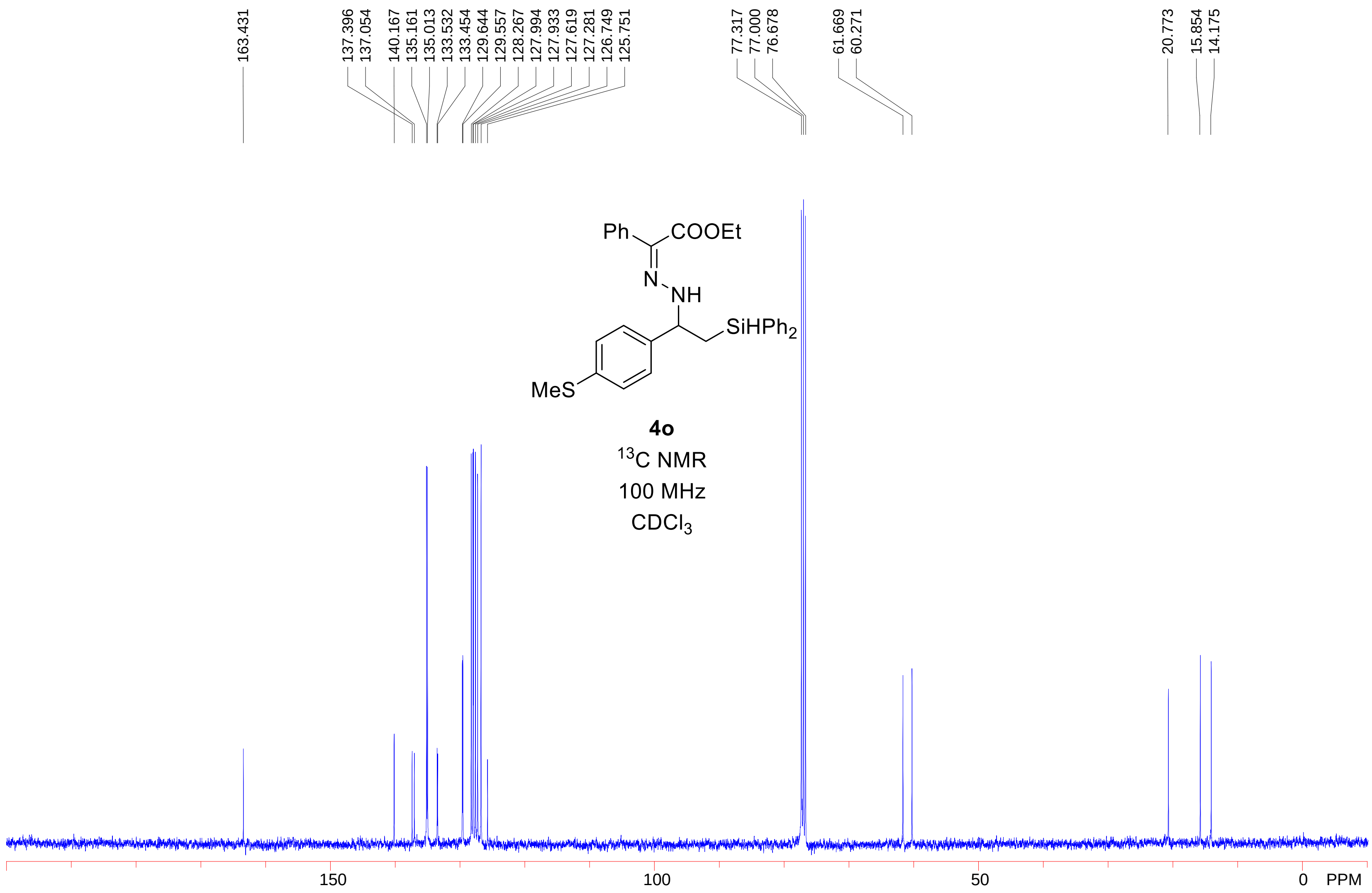

Supplementary Figure 63. ^{13}C NMR spectrum of **4o**.

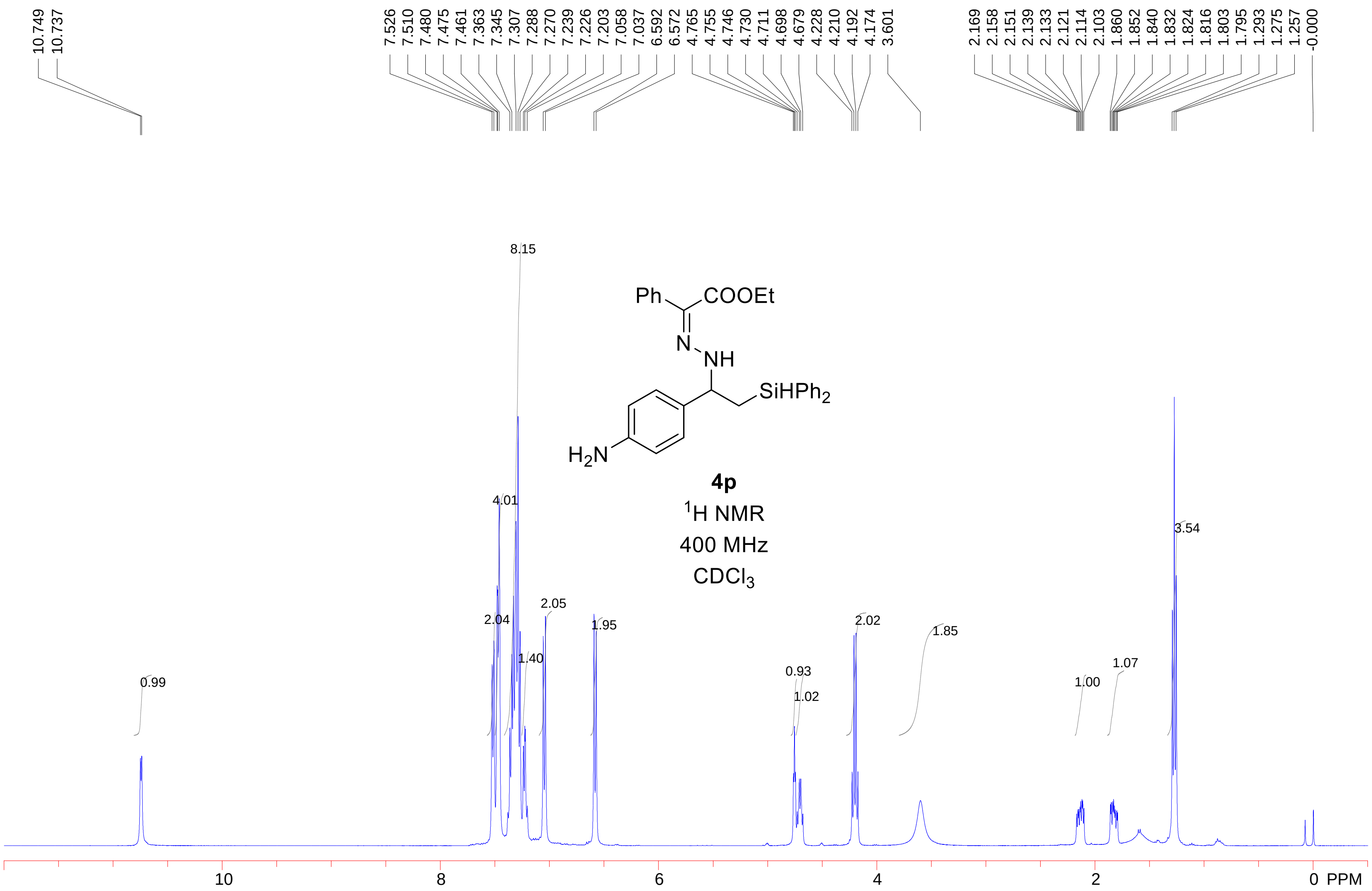

Supplementary Figure 64. ^{1}H NMR spectrum of **4p**.

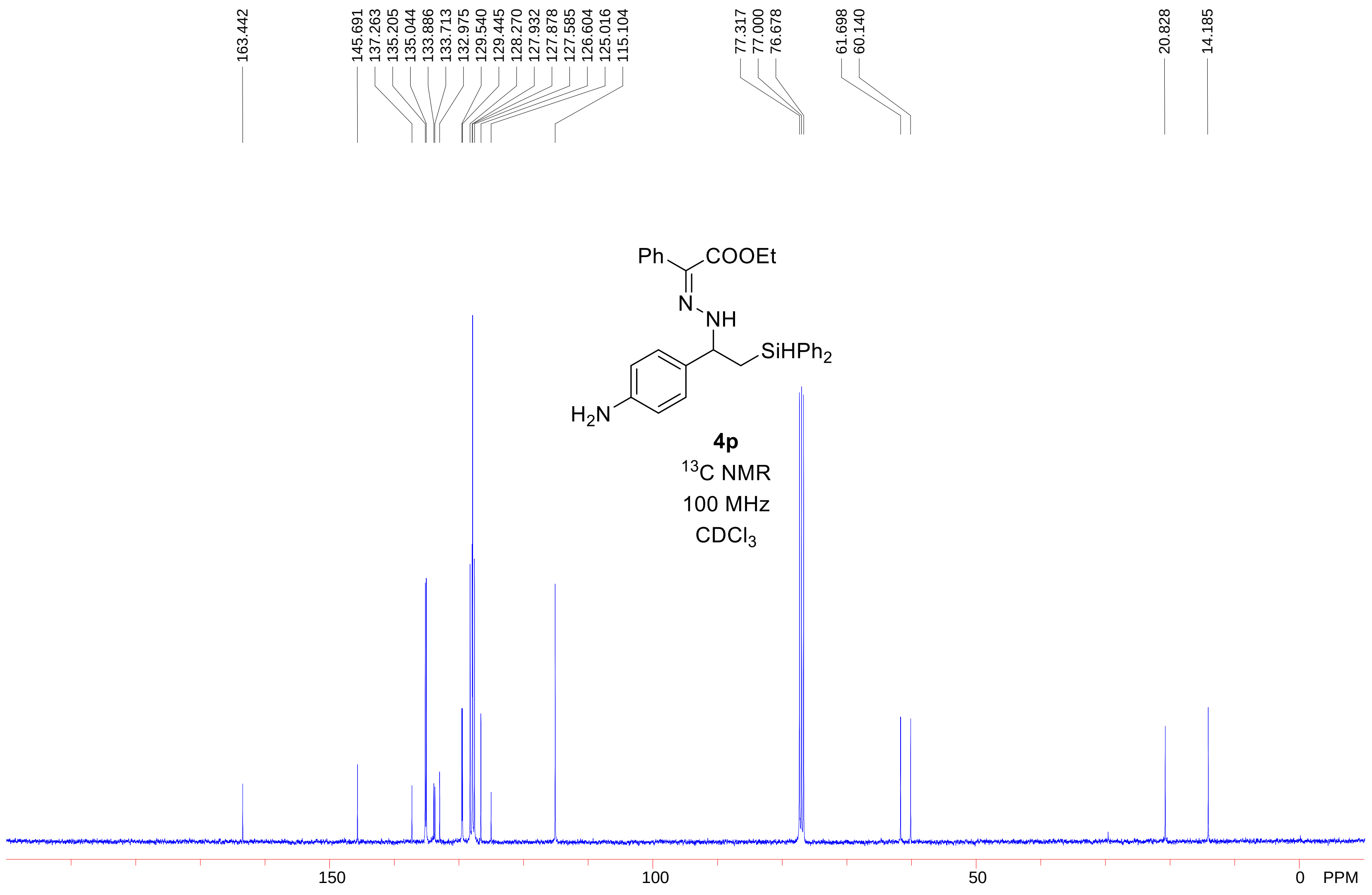

Supplementary Figure 65. ^{13}C NMR spectrum of **4p**.

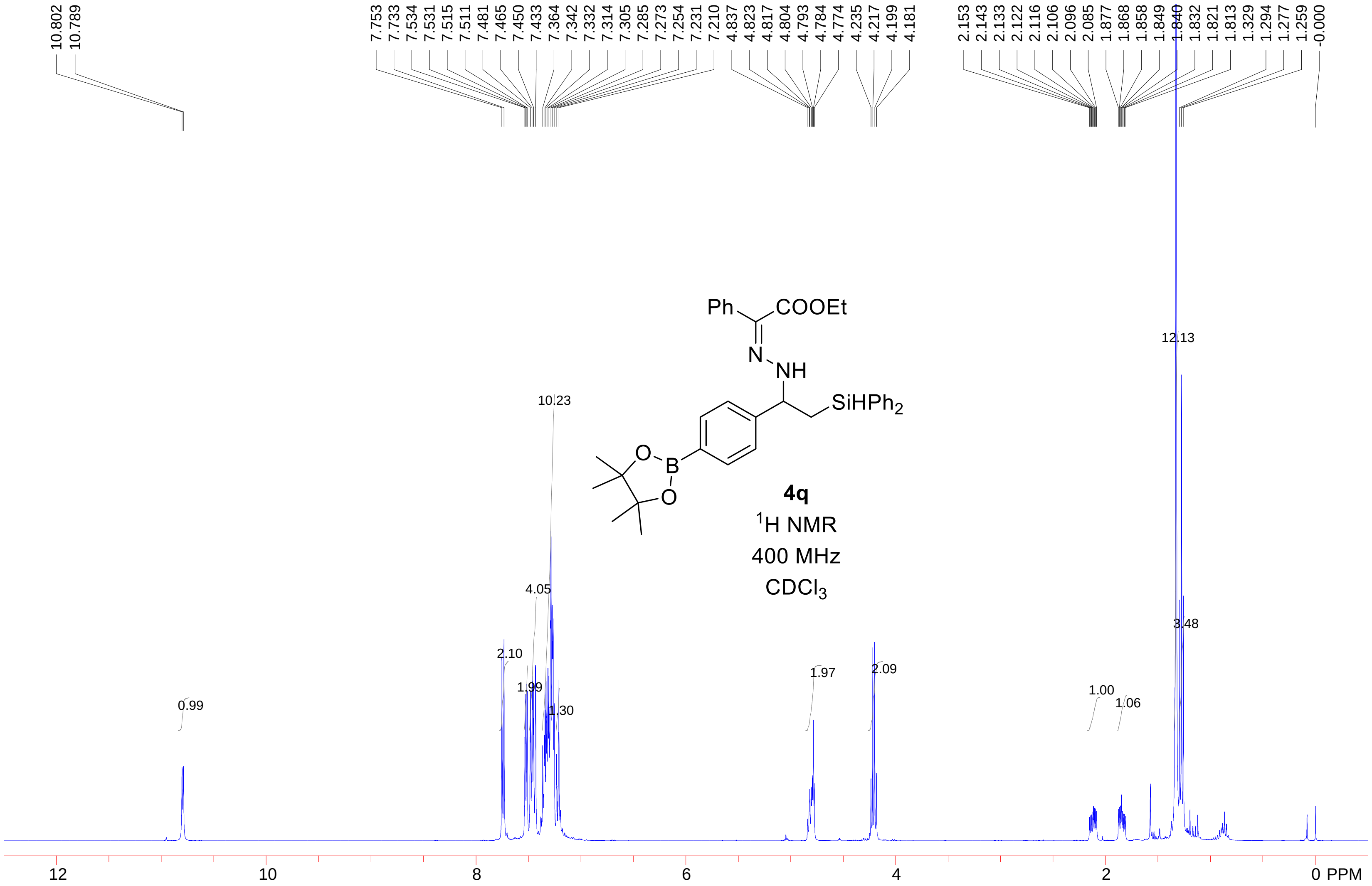

Supplementary Figure 66. ^{1}H NMR spectrum of **4q**.

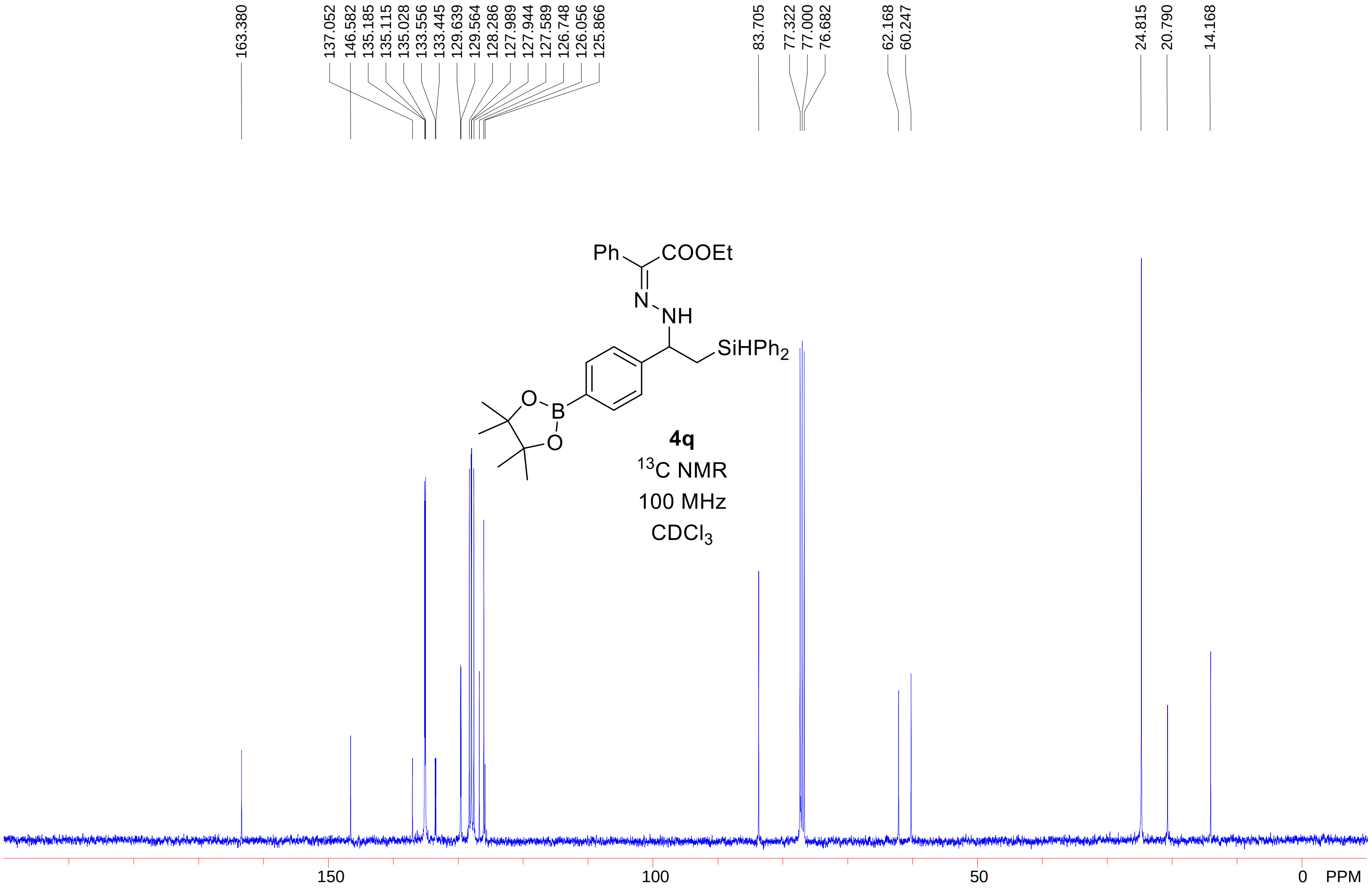

Supplementary Figure 67. ^{13}C NMR spectrum of **4q**.

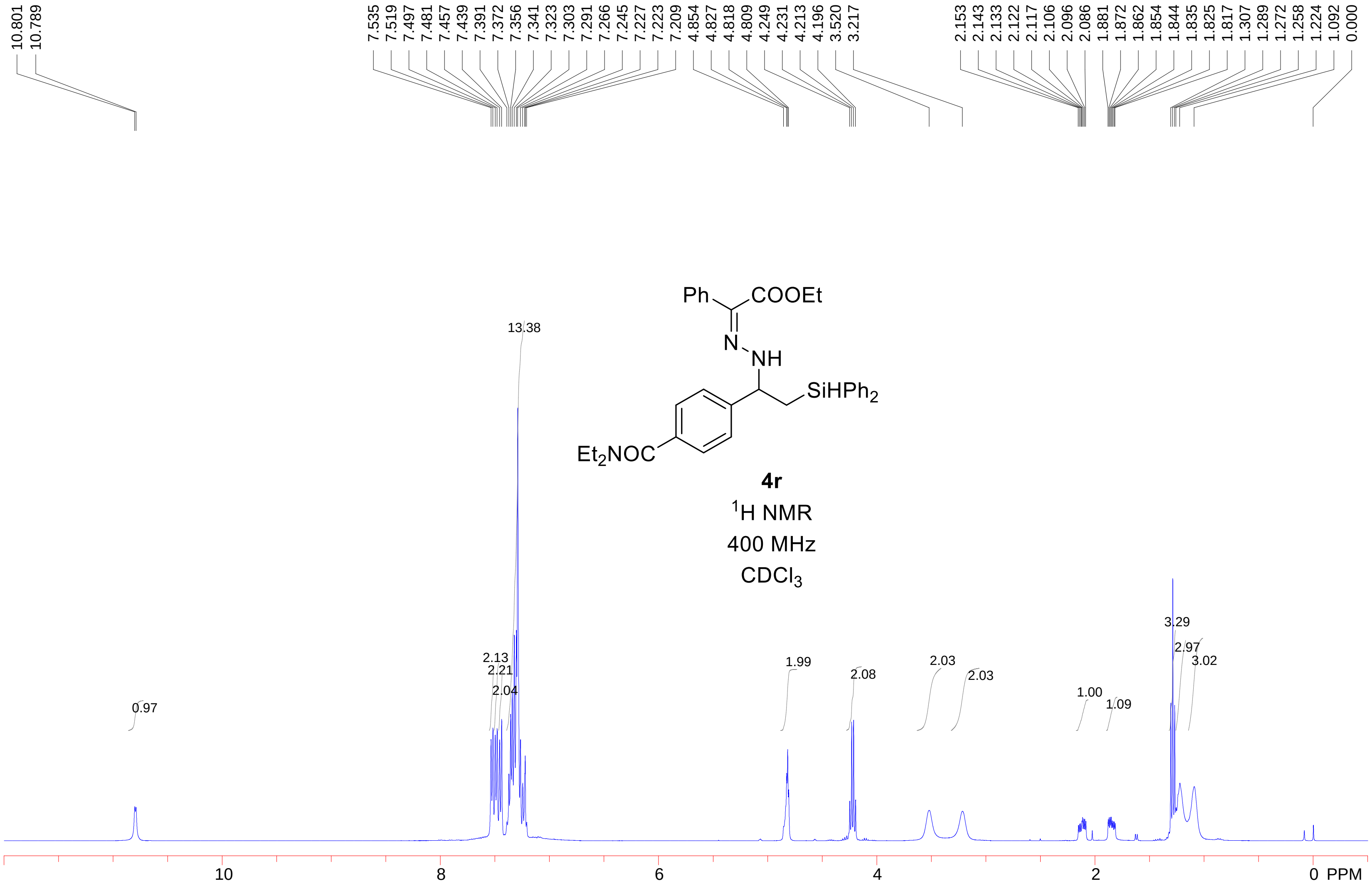

Supplementary Figure 68. ^{1}H NMR spectrum of **4r**.

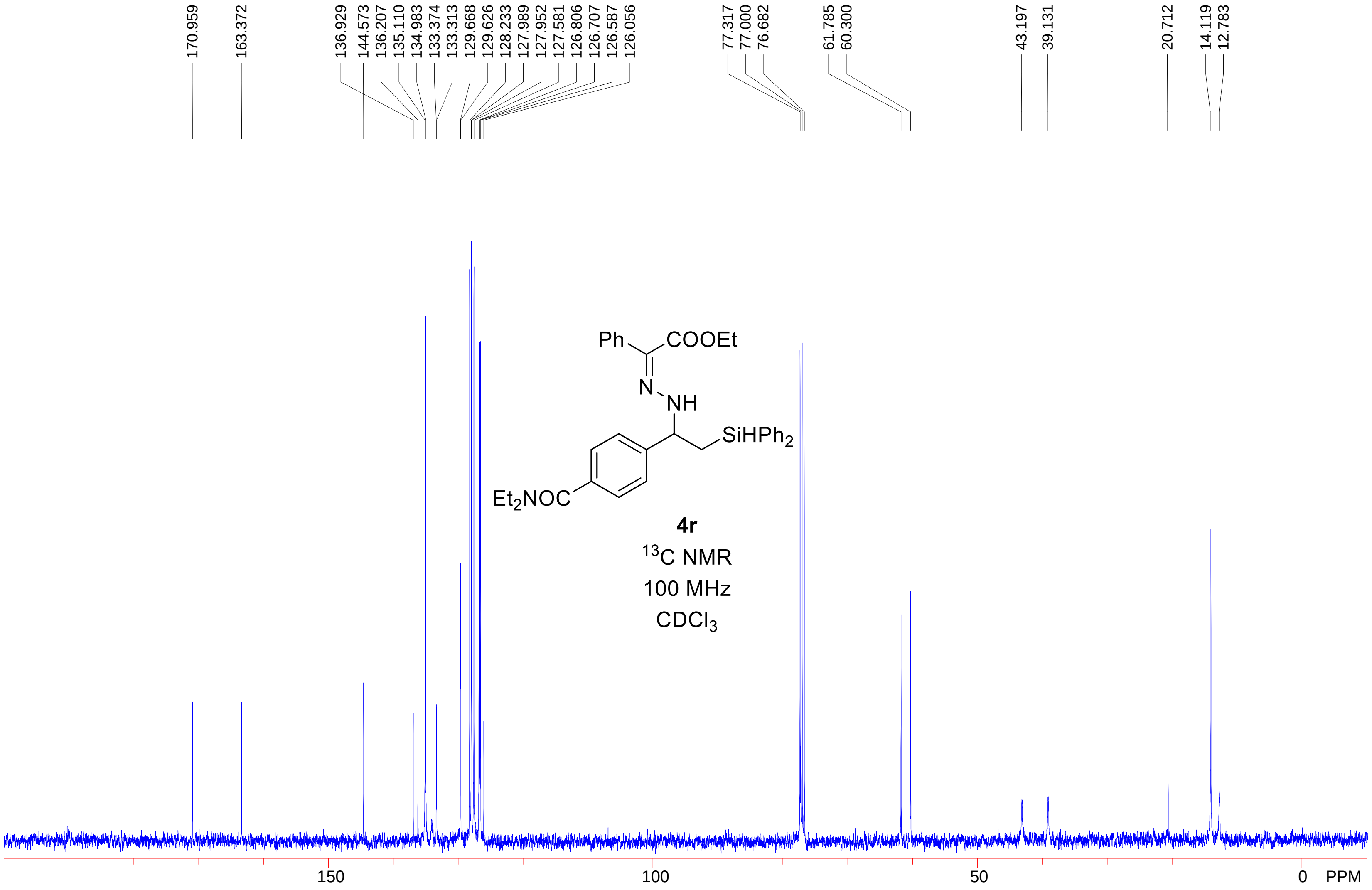

Supplementary Figure 69. ^{13}C NMR spectrum of **4r**.

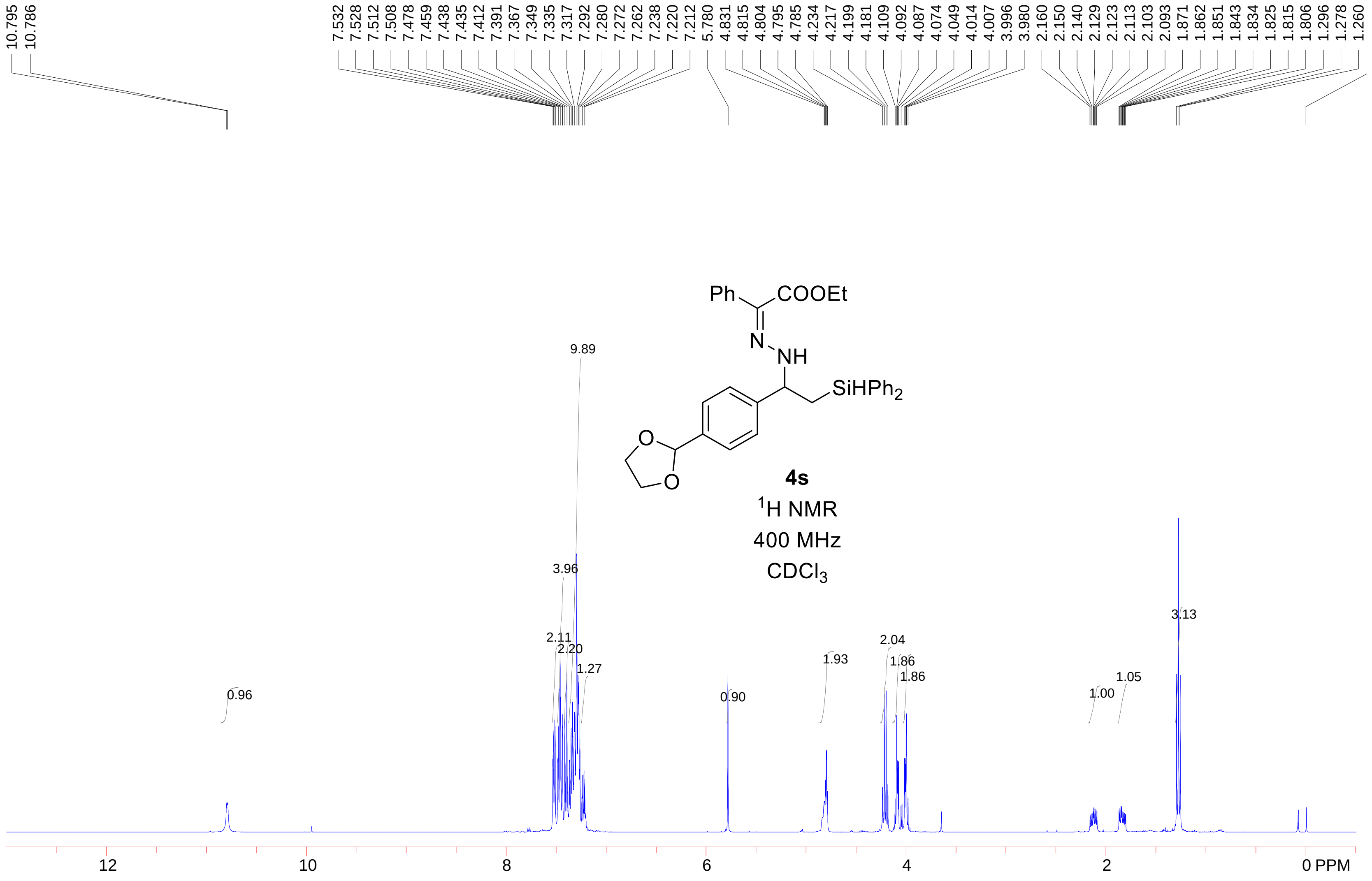

Supplementary Figure 70. ^{1}H NMR spectrum of **4s**.

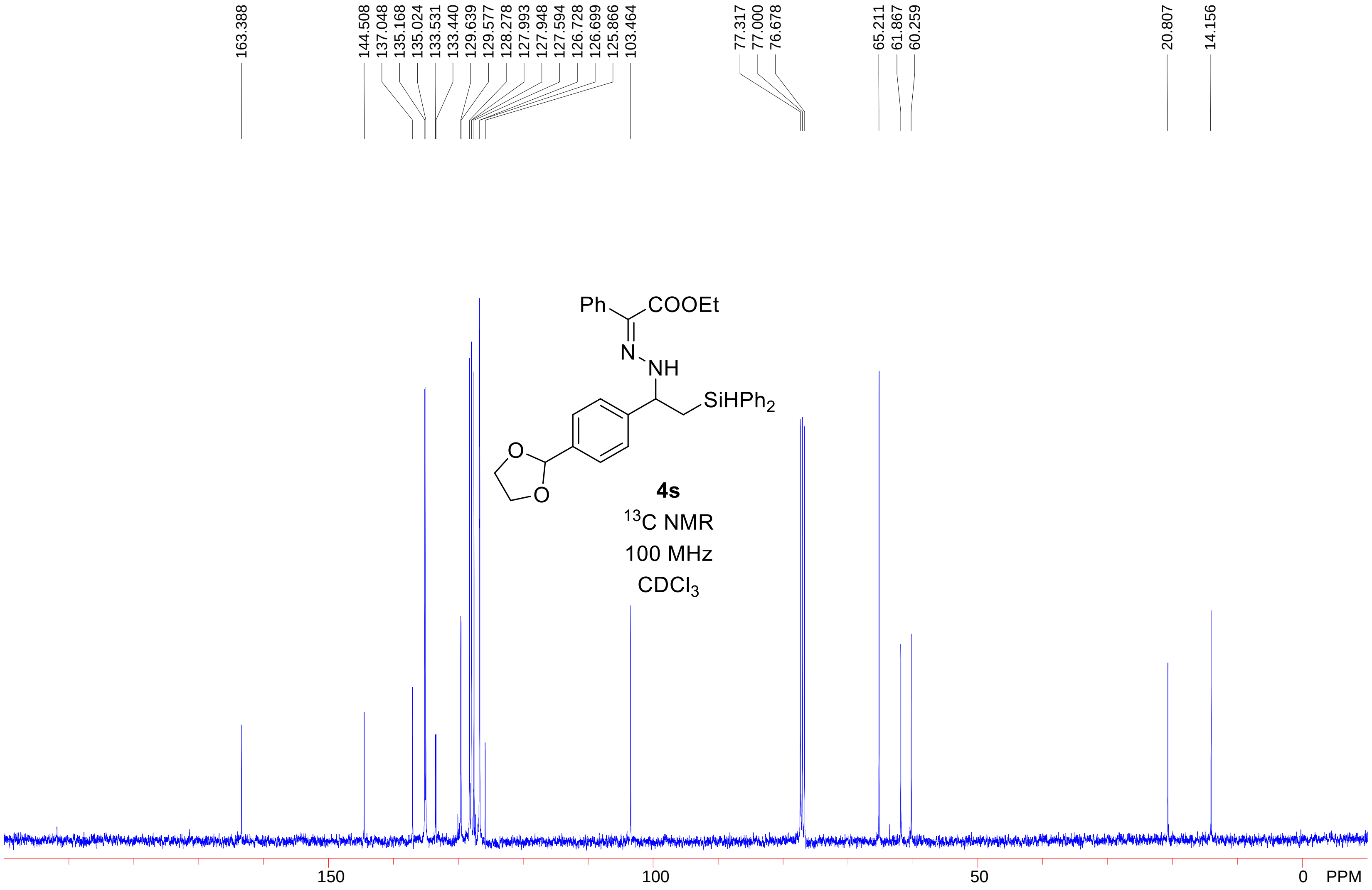

Supplementary Figure 71. ^{13}C NMR spectrum of **4s**.

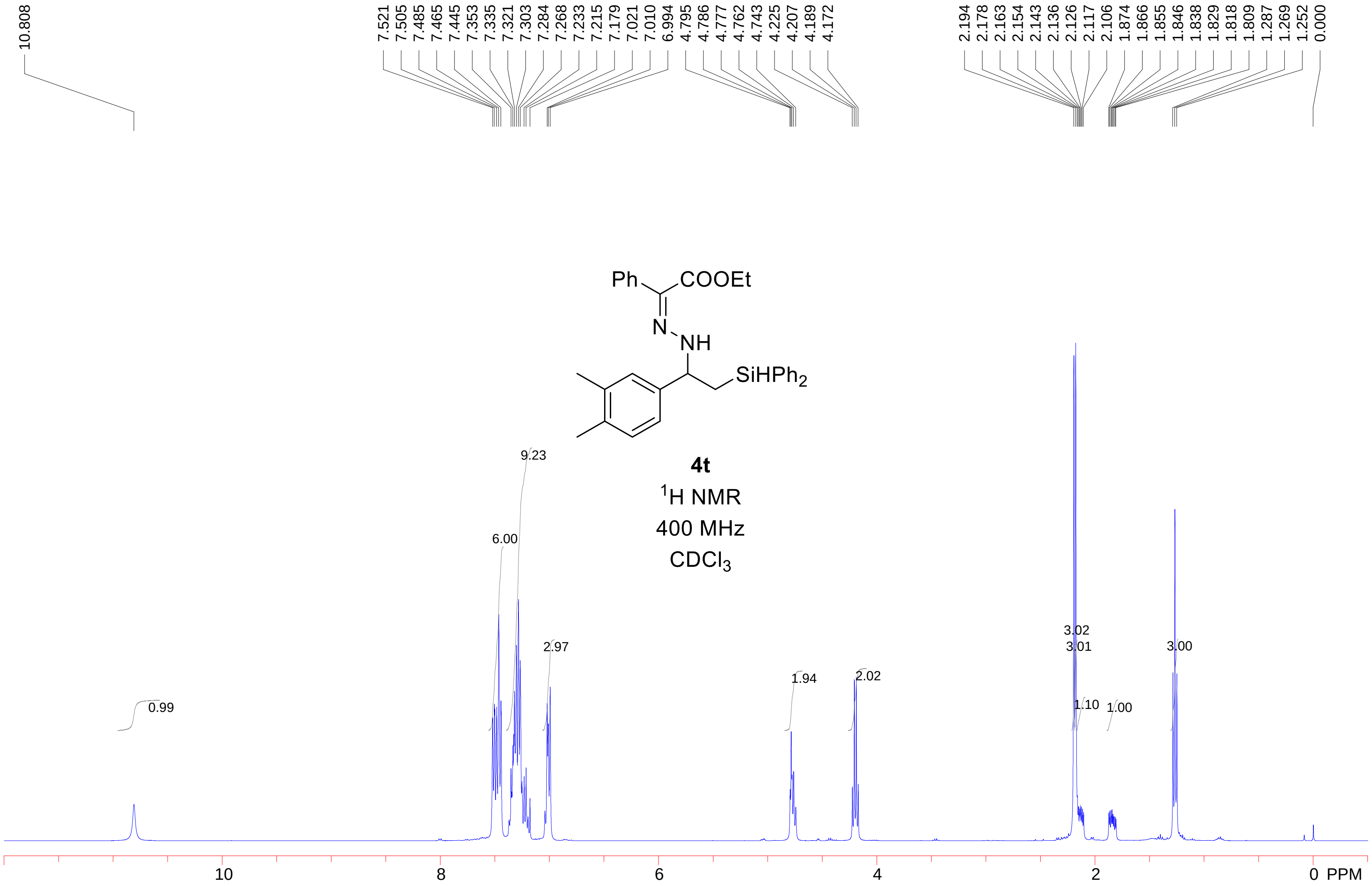

Supplementary Figure 72. ^{1}H NMR spectrum of **4t**.

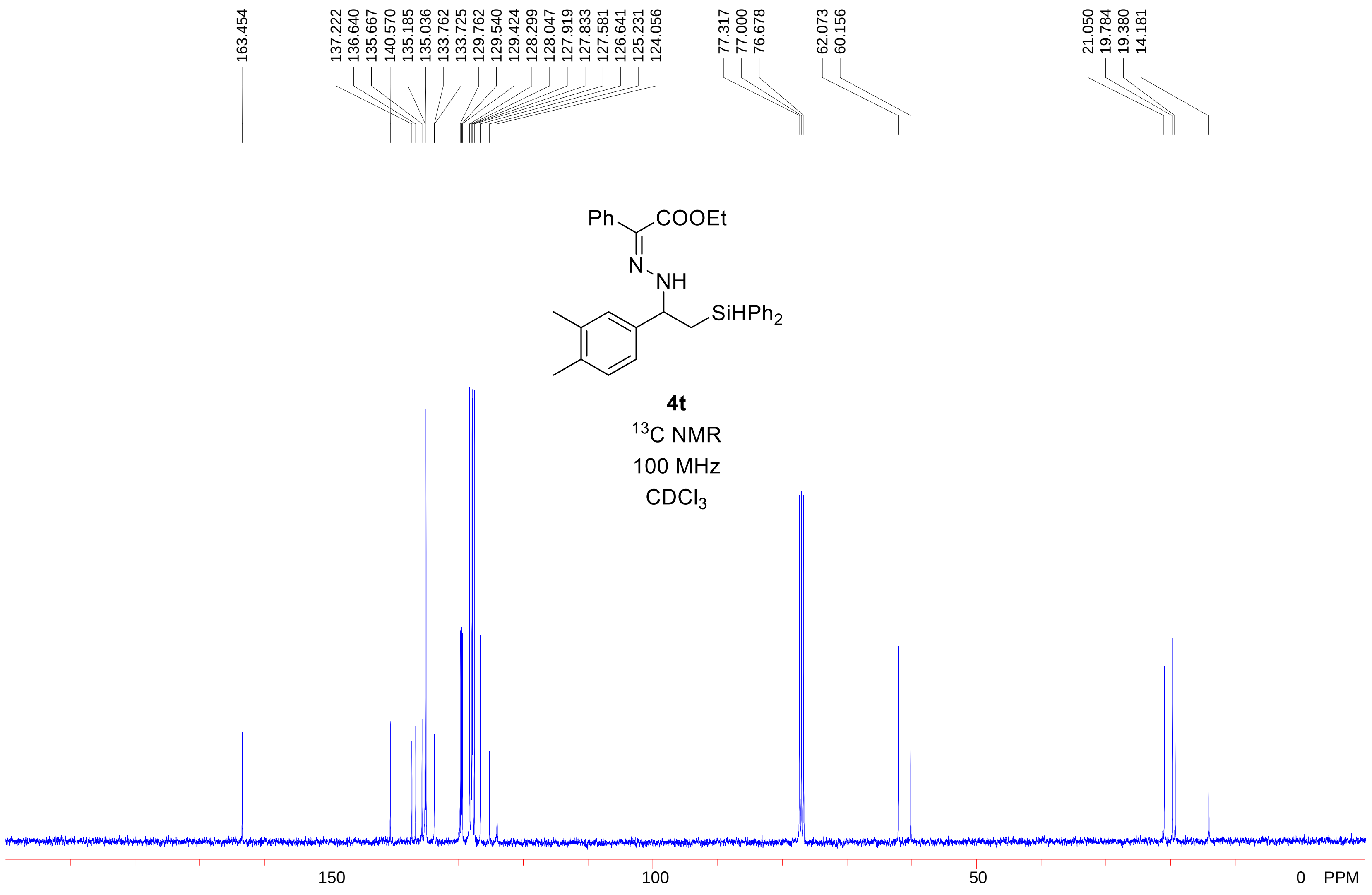

Supplementary Figure 73. ^{13}C NMR spectrum of **4t**.

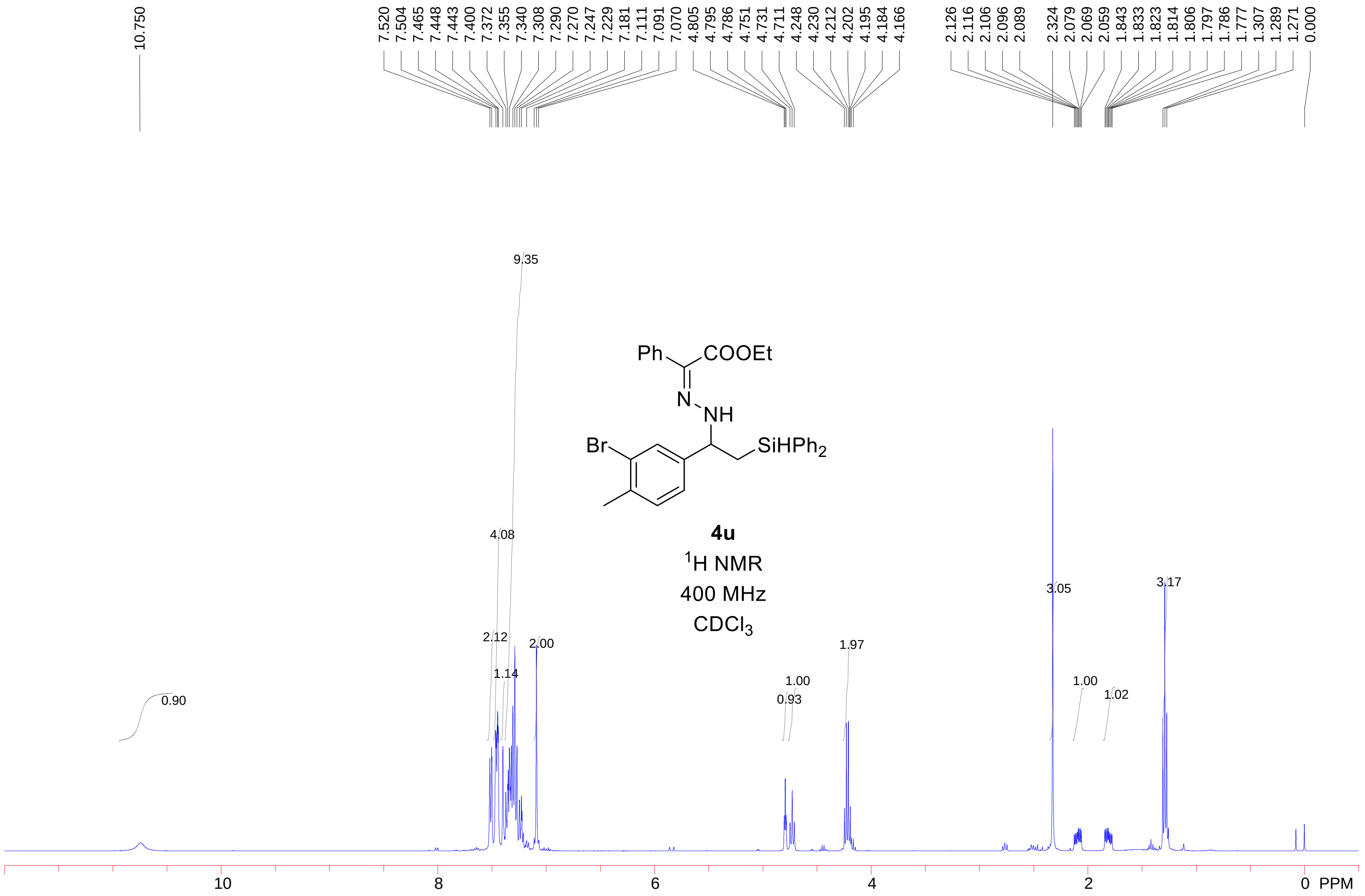

Supplementary Figure 74. ^{1}H NMR spectrum of **4u**.

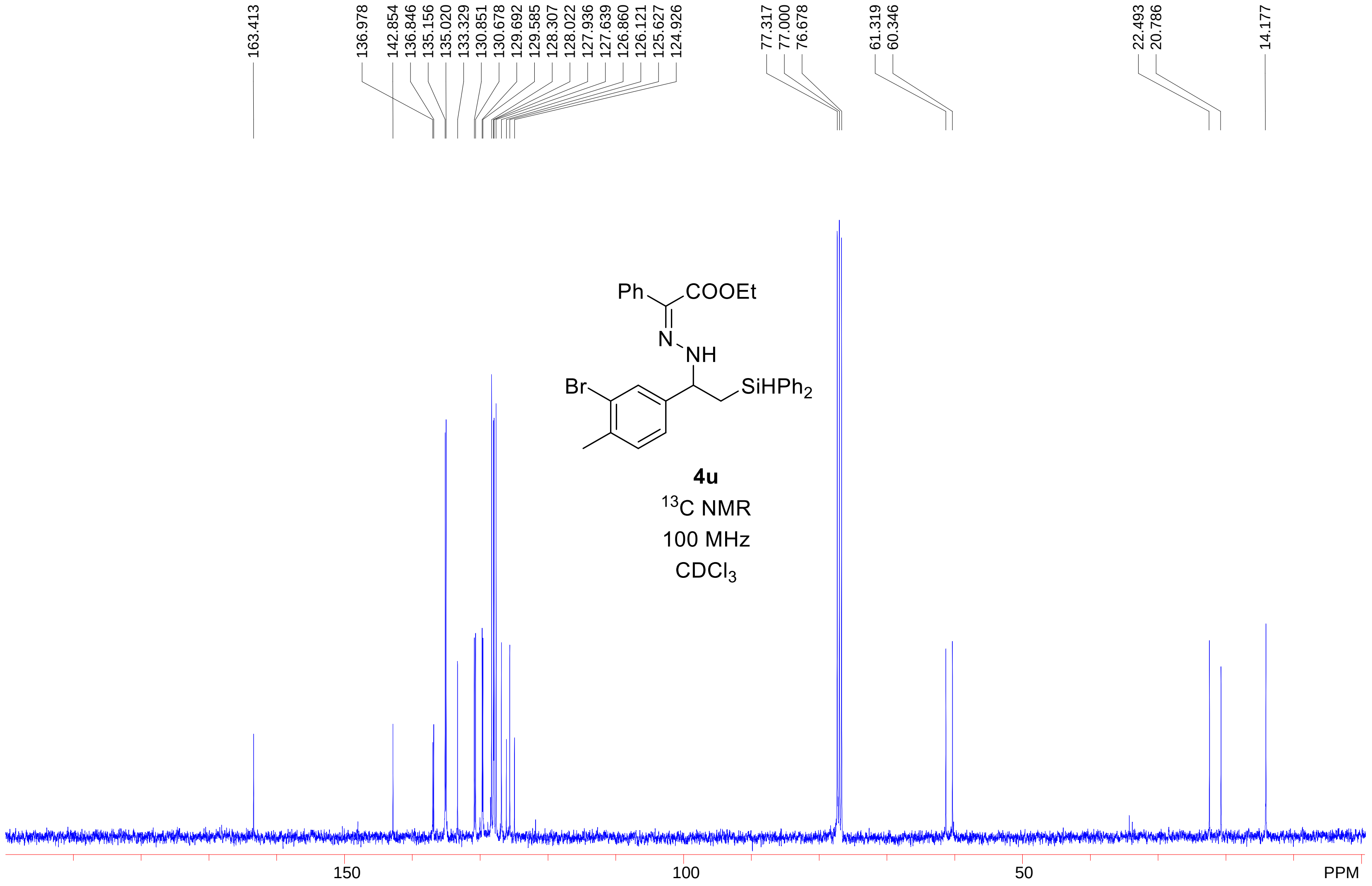

Supplementary Figure 75. ^{13}C NMR spectrum of **4u**.

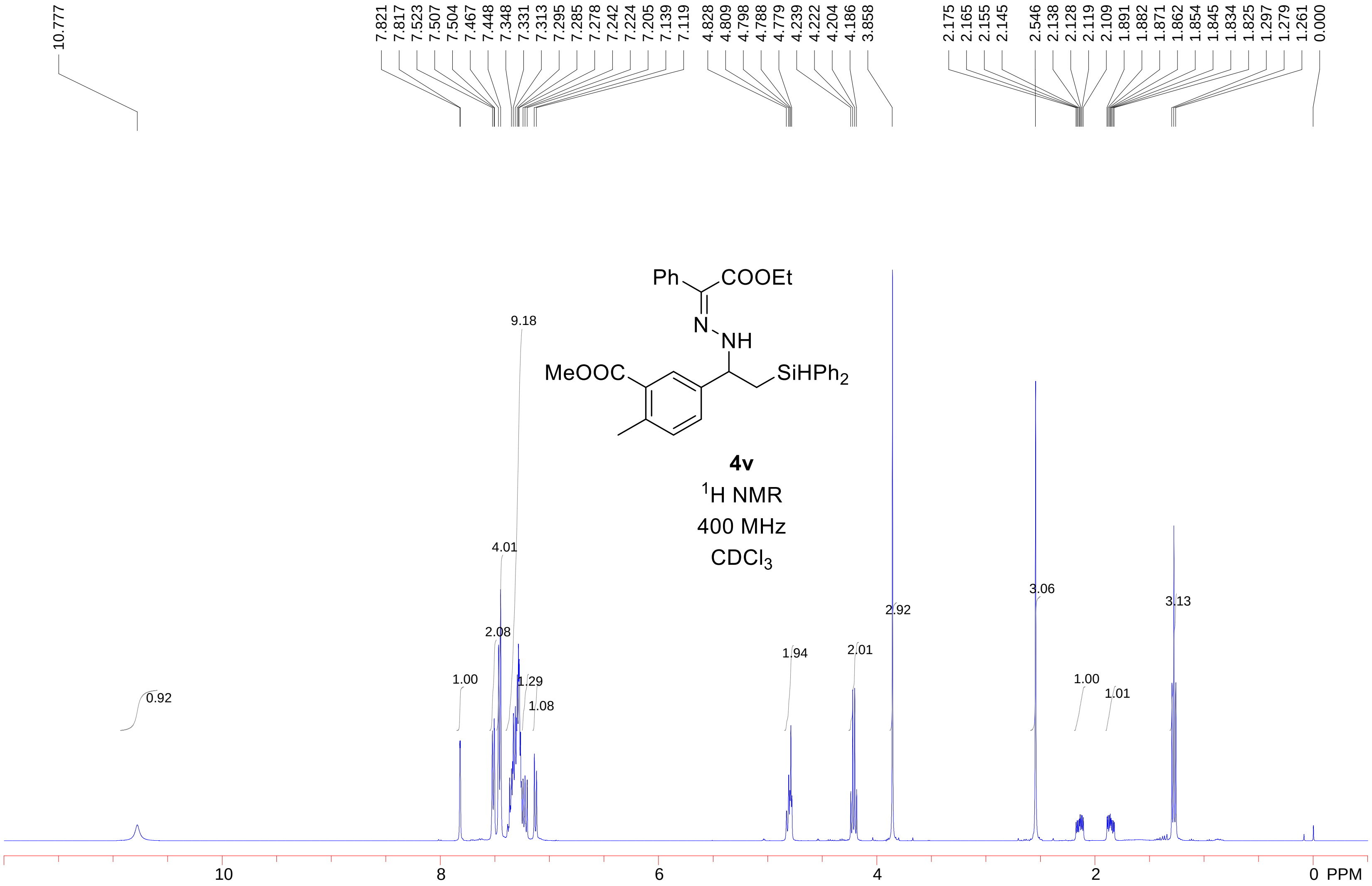

Supplementary Figure 76. ^{1}H NMR spectrum of **4v**.

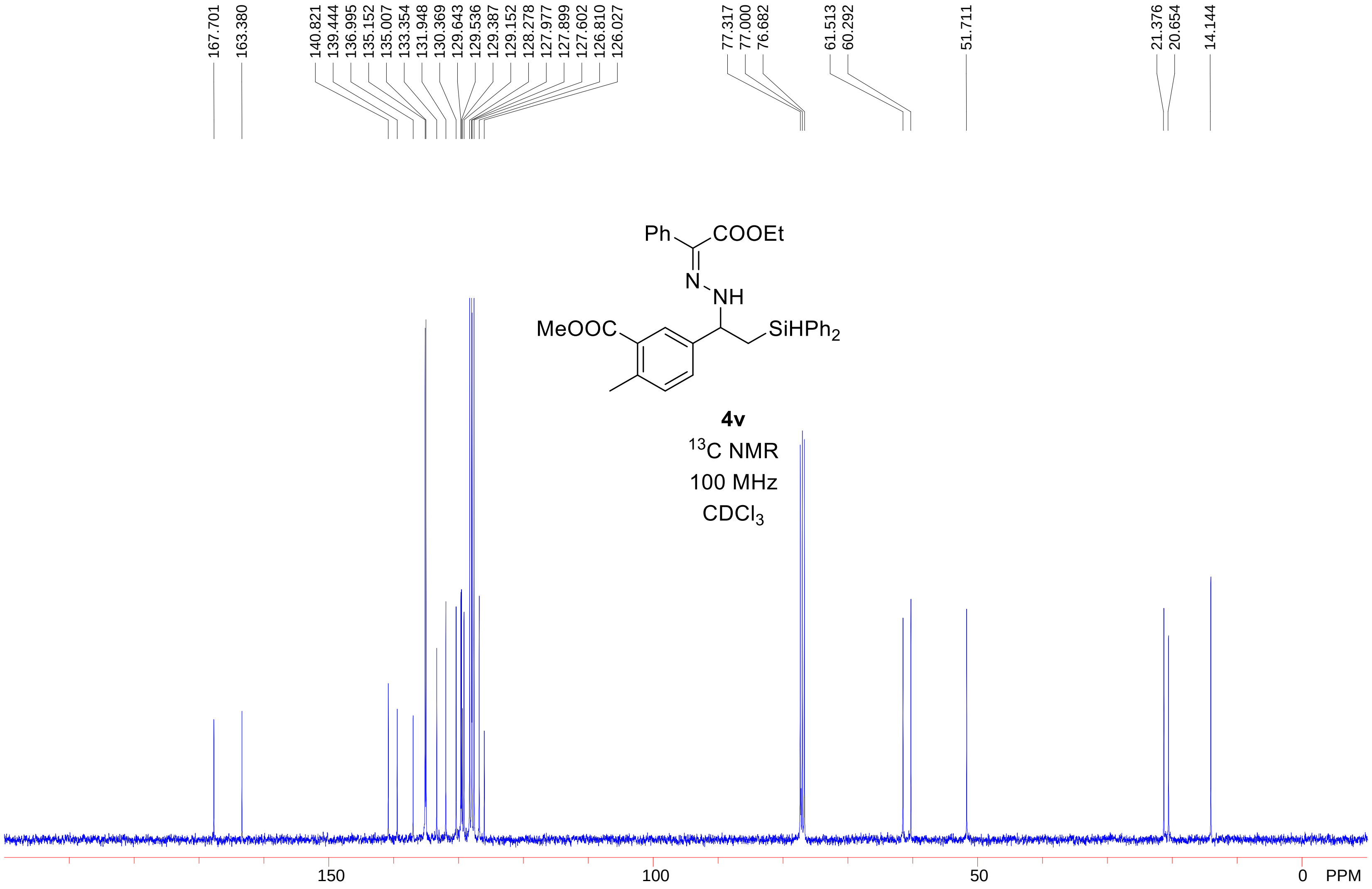

Supplementary Figure 77. ^{13}C NMR spectrum of **4v**.

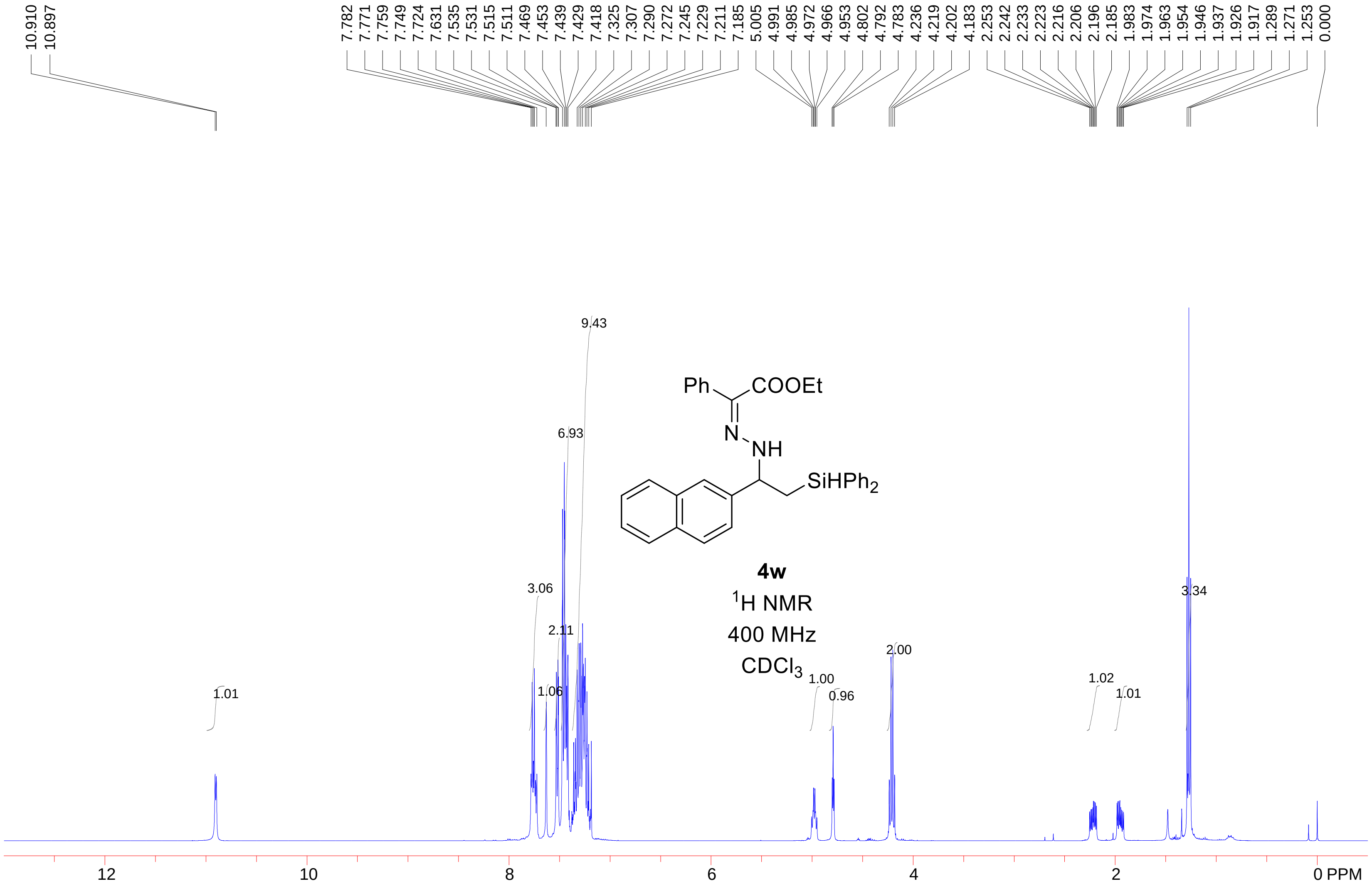

Supplementary Figure 78. ^{1}H NMR spectrum of **4w**.

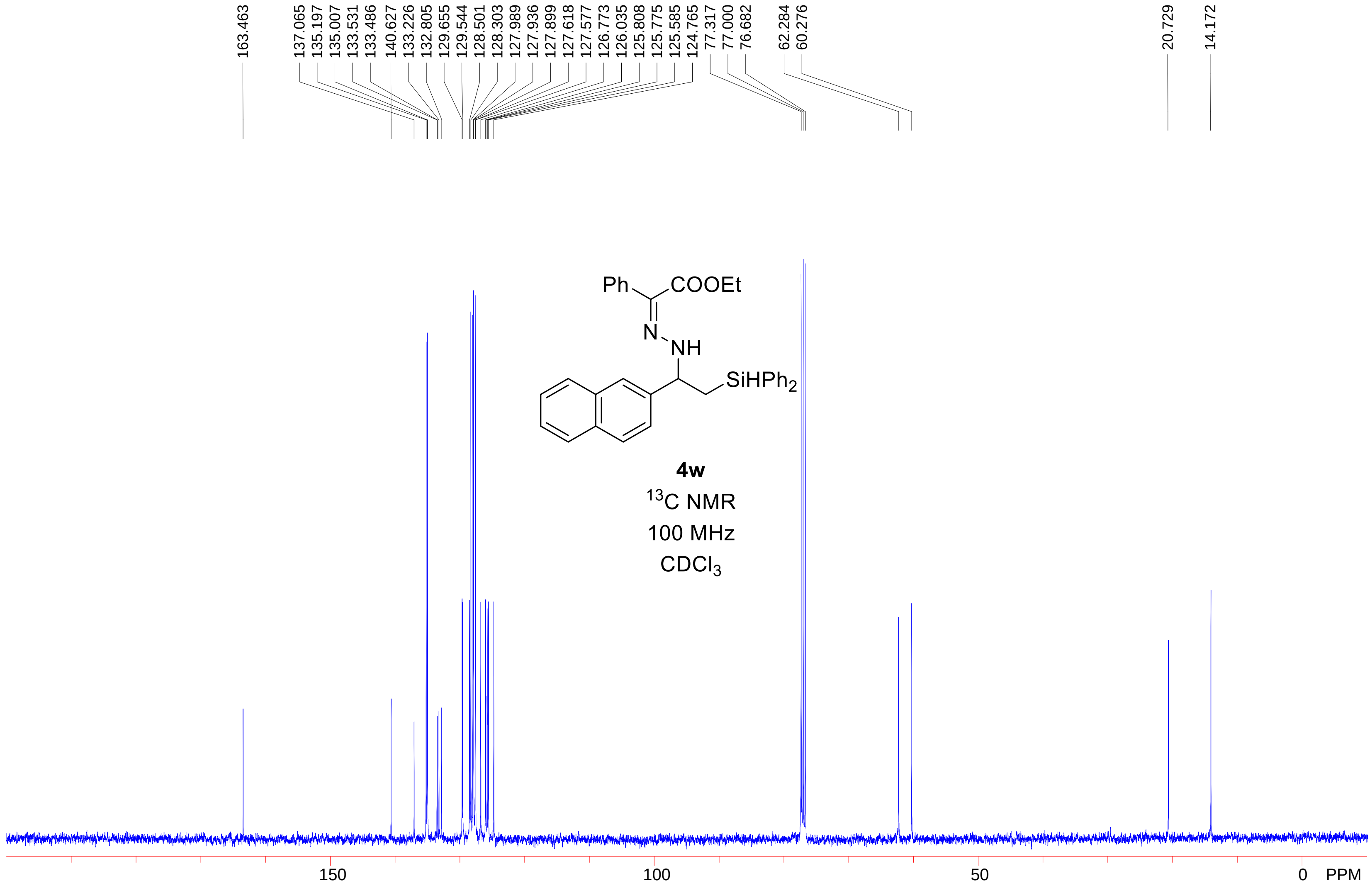

Supplementary Figure 79. ^{13}C NMR spectrum of **4w**.

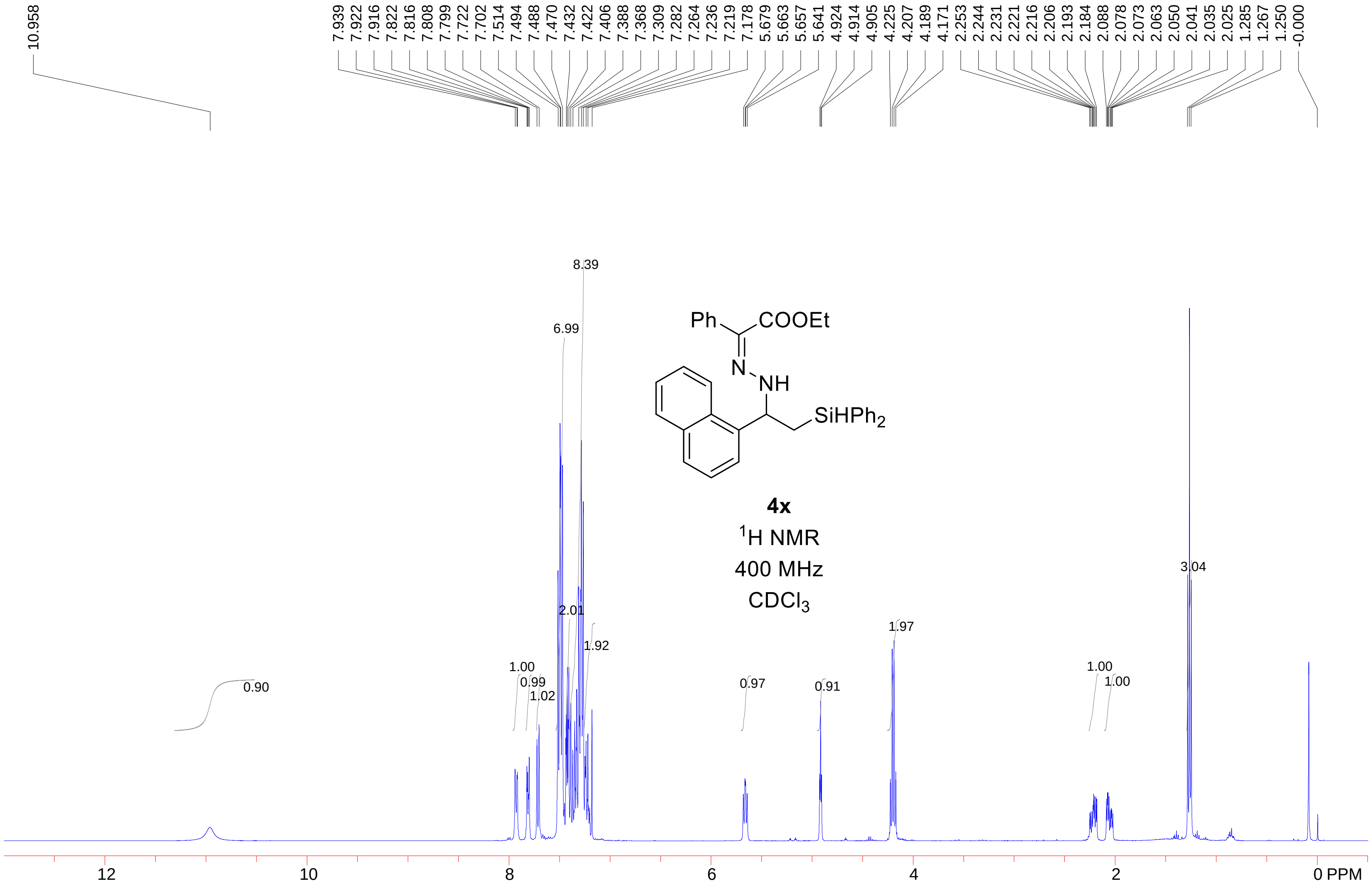

Supplementary Figure 80. ^{1}H NMR spectrum of **4x**.

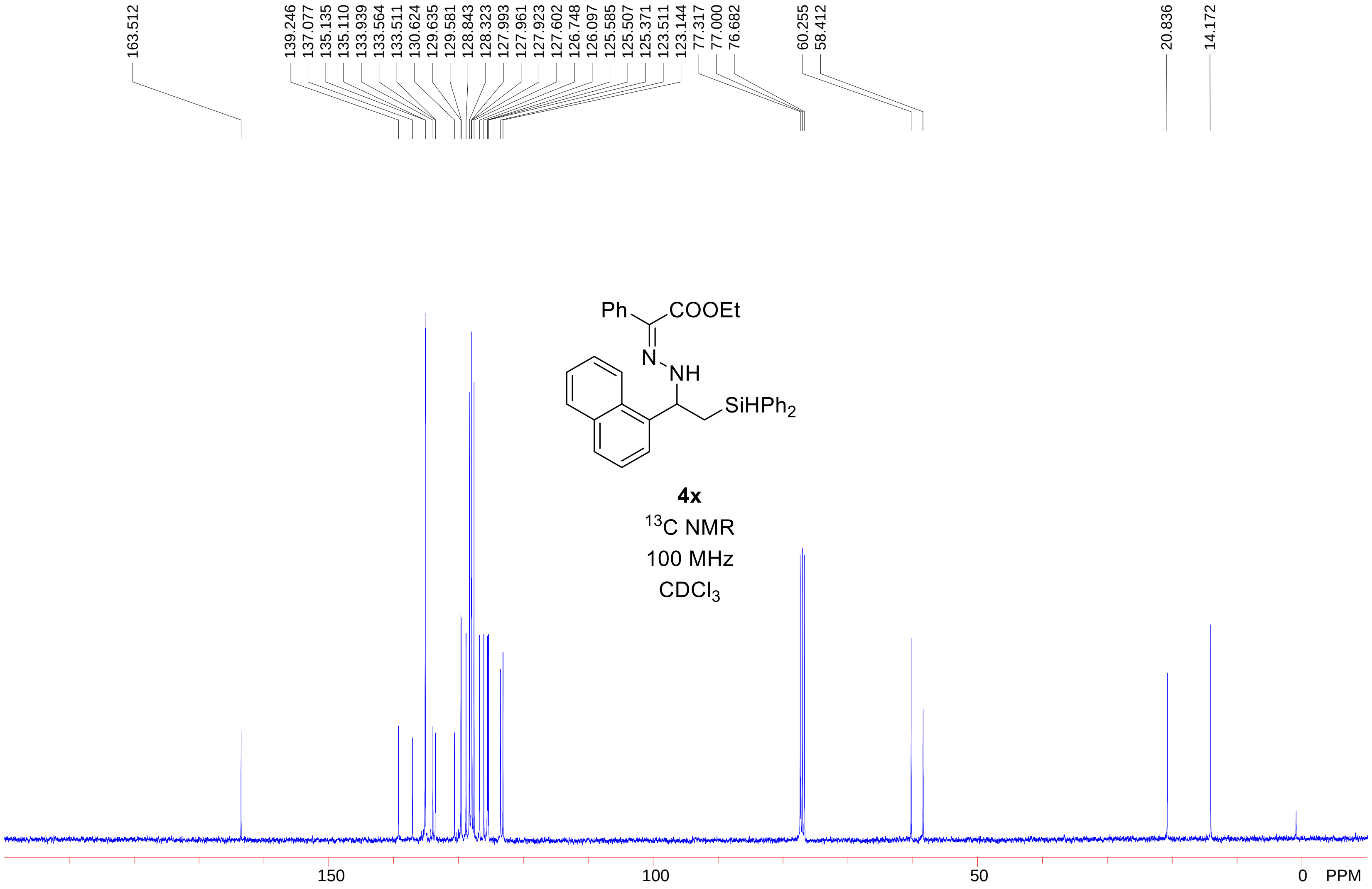

Supplementary Figure 81. ^{13}C NMR spectrum of **4x**.

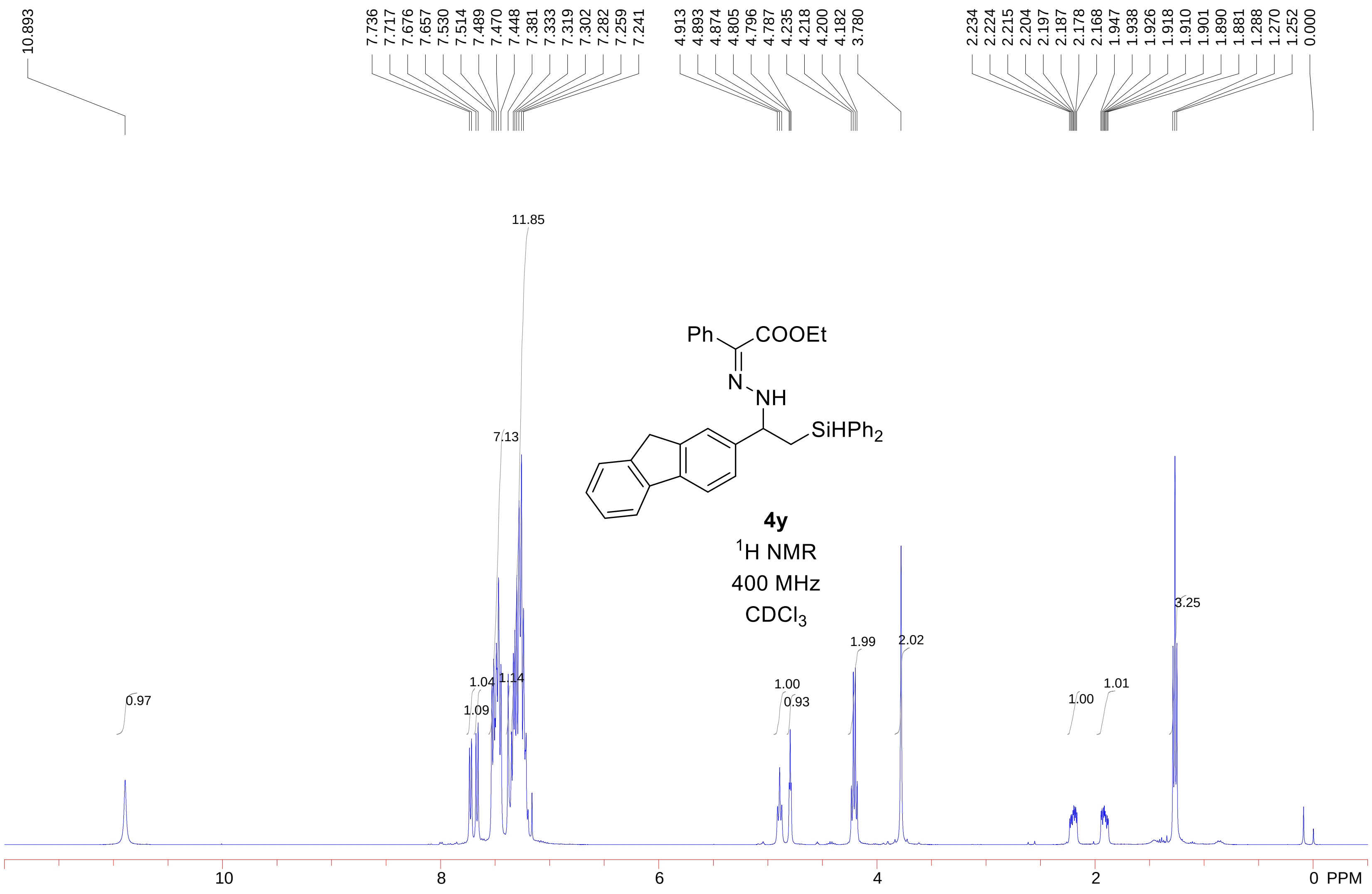

Supplementary Figure 82. ^{1}H NMR spectrum of **4y**.

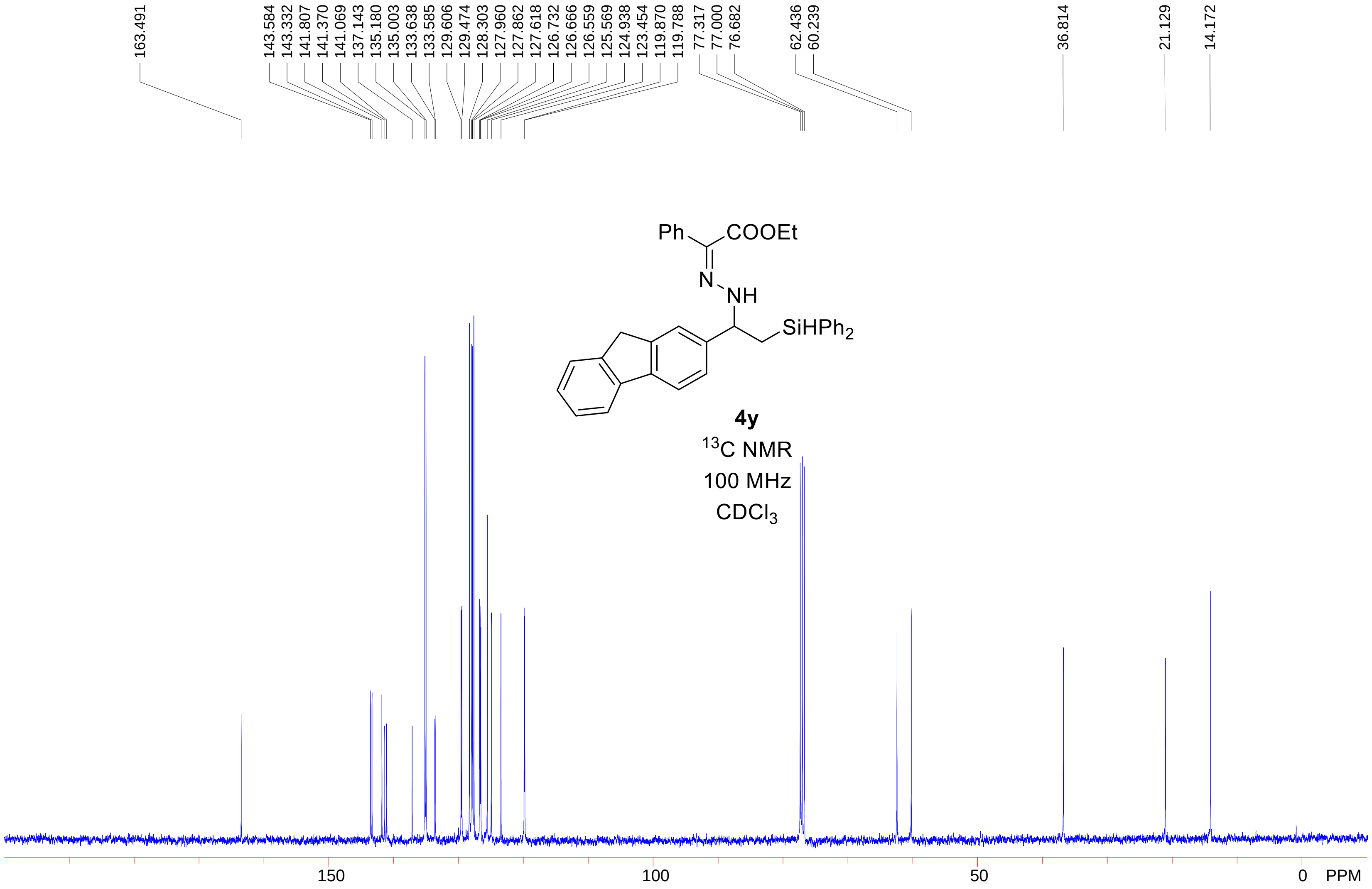

Supplementary Figure 83. ^{13}C NMR spectrum of **4y**.

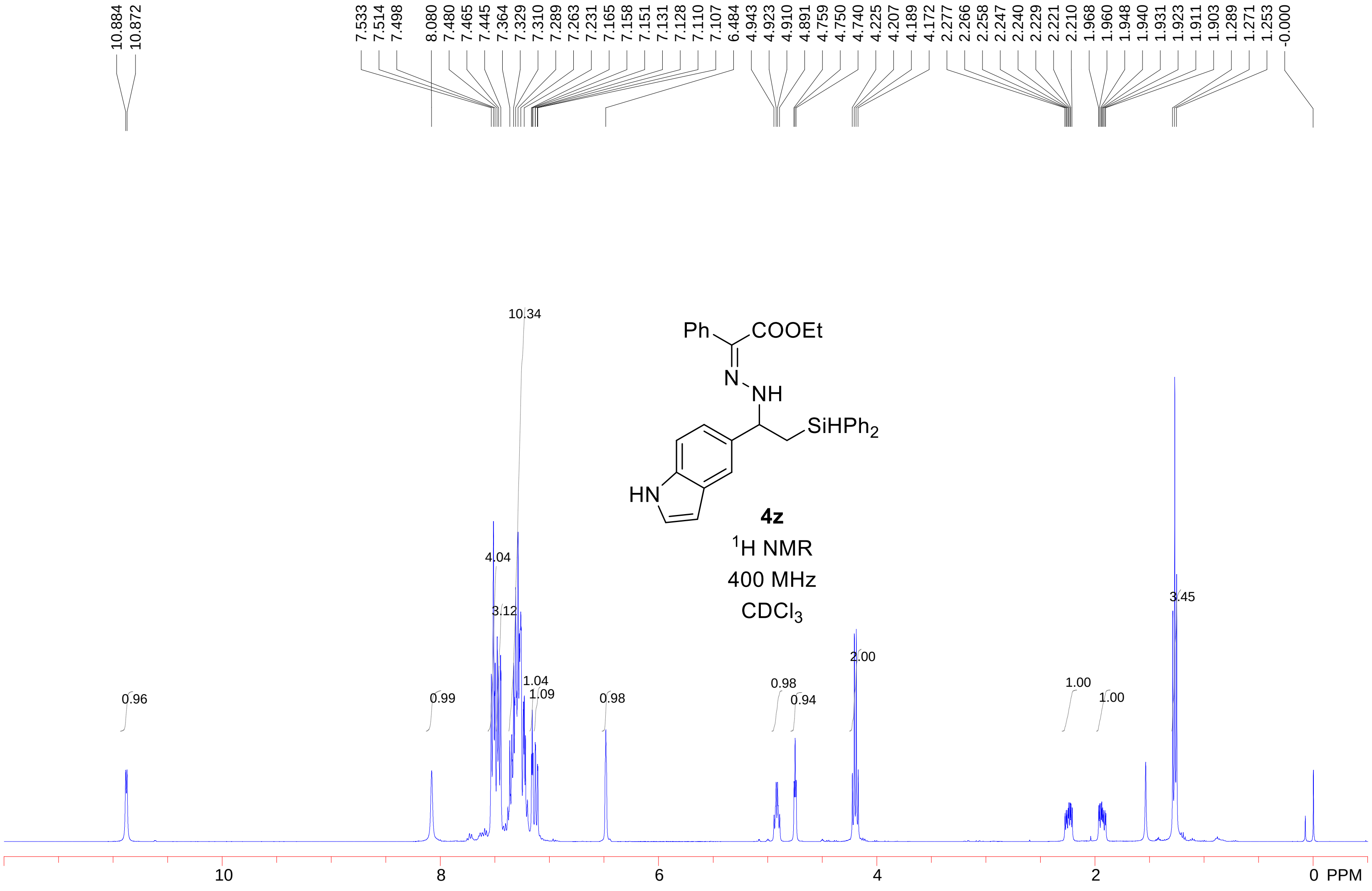

Supplementary Figure 84. ^{1}H NMR spectrum of **4z**.

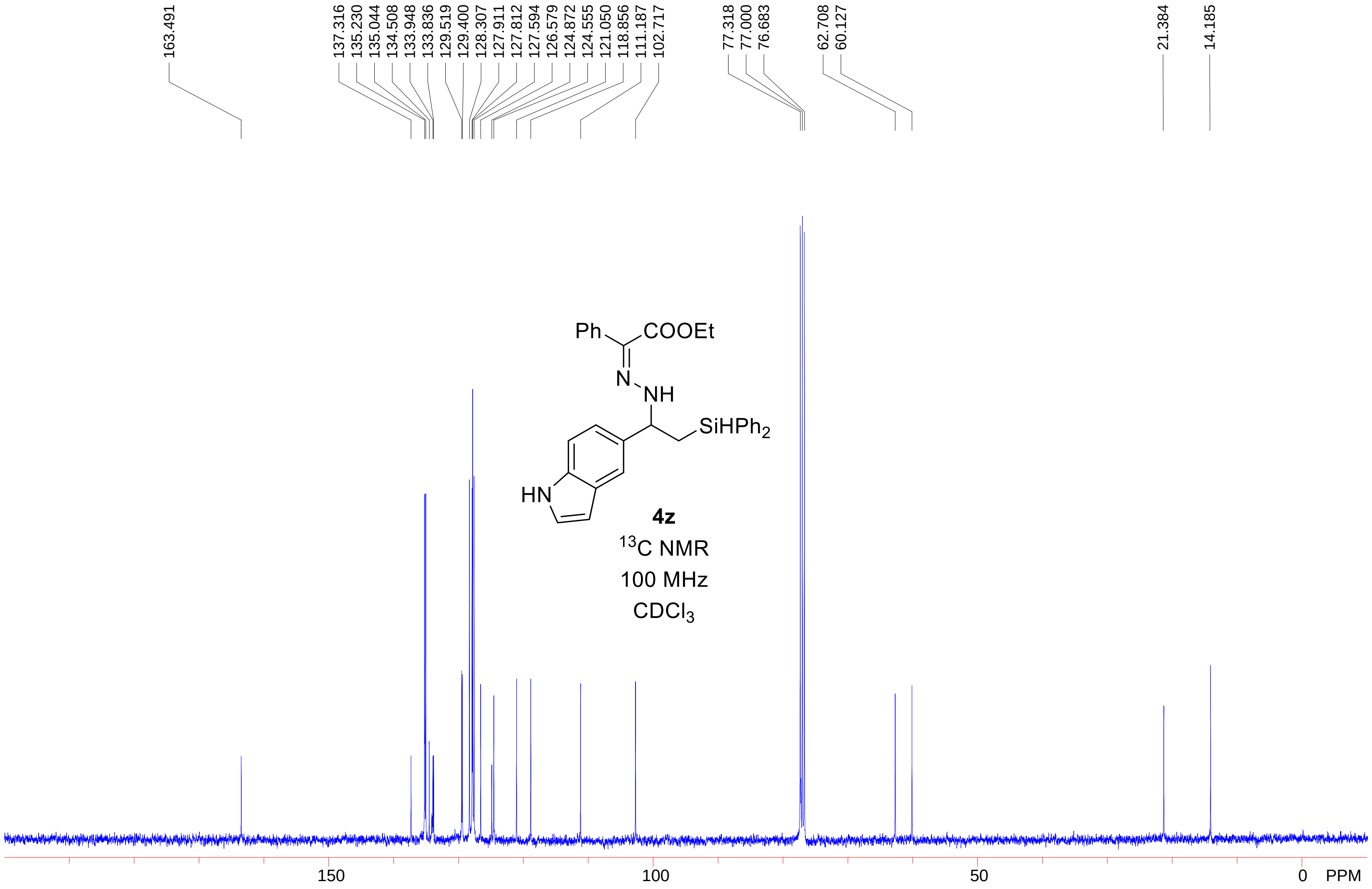

Supplementary Figure 85. ^{13}C NMR spectrum of **4z**.

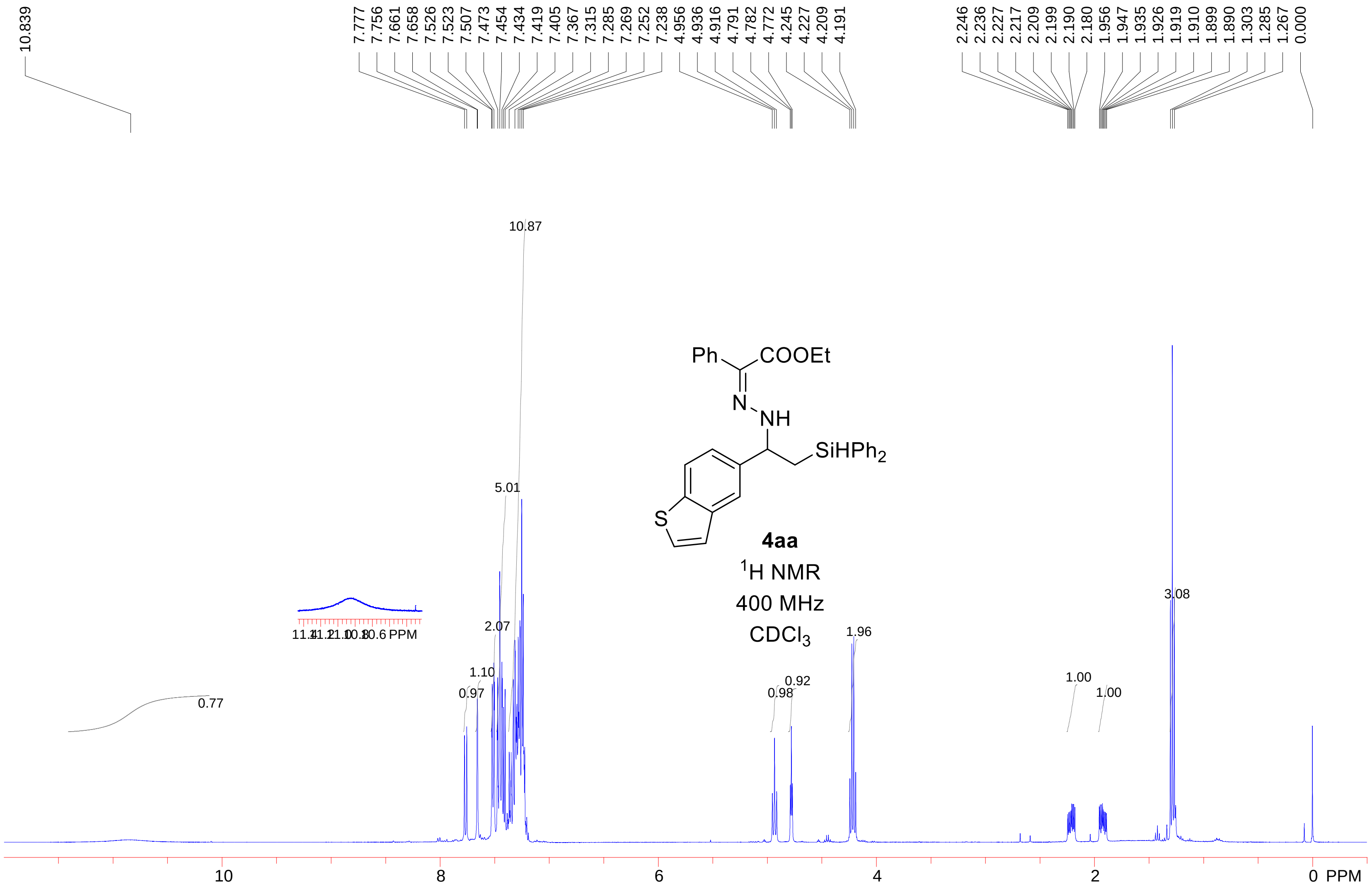

Supplementary Figure 86. ^{1}H NMR spectrum of **4aa**.

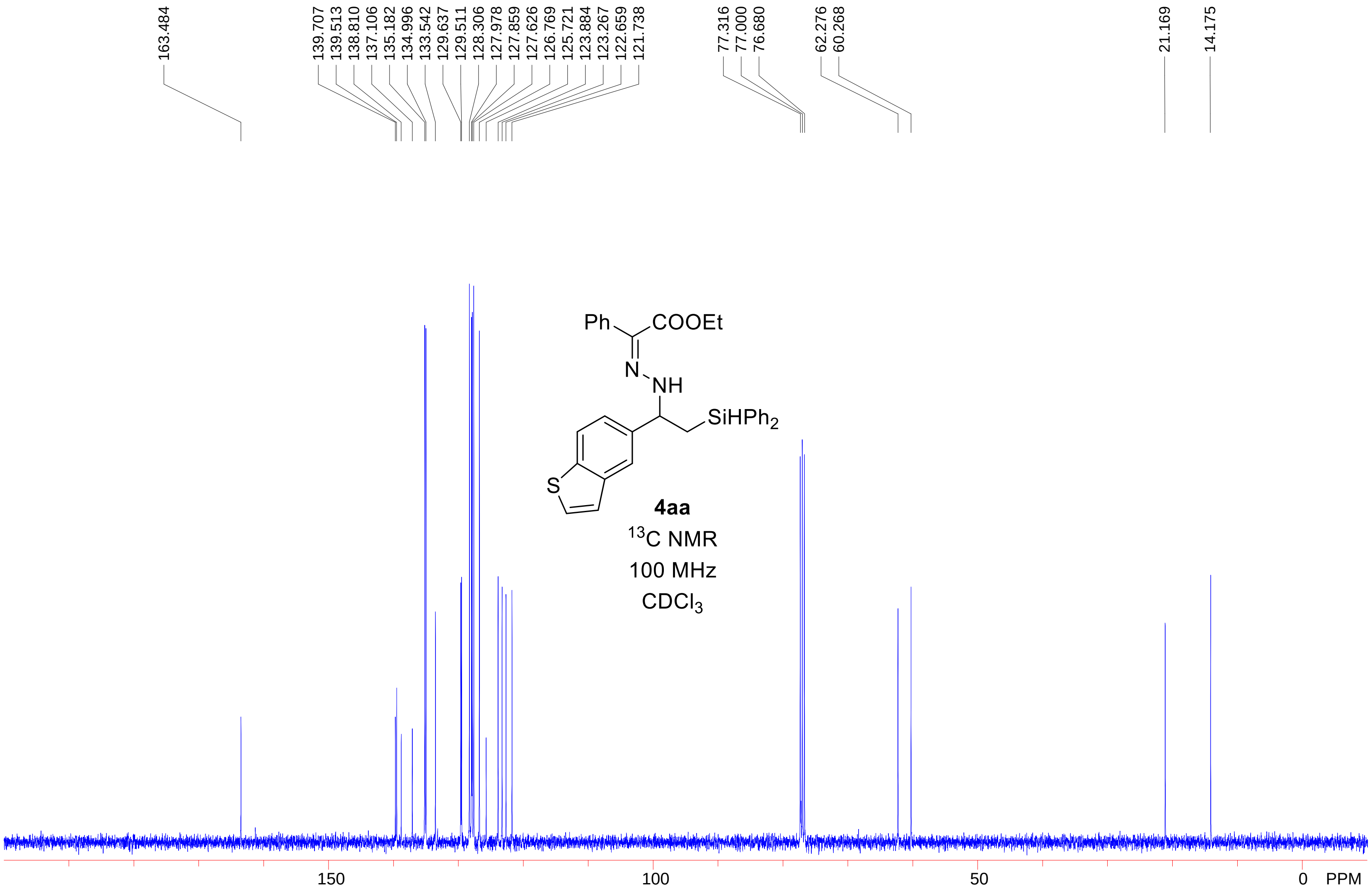

Supplementary Figure 87. ^{13}C NMR spectrum of **4aa**.

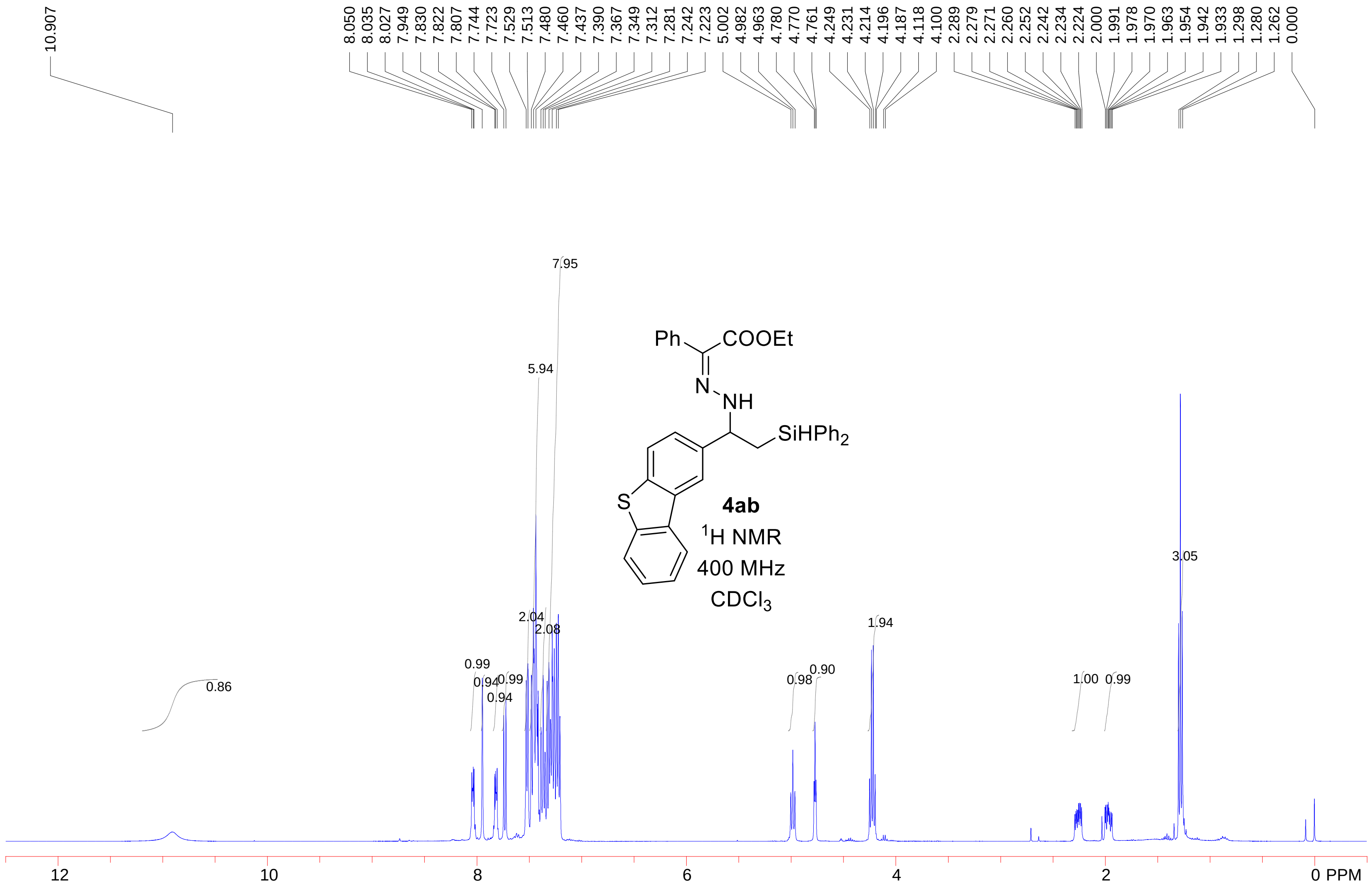

Supplementary Figure 88. ^{1}H NMR spectrum of **4ab**.

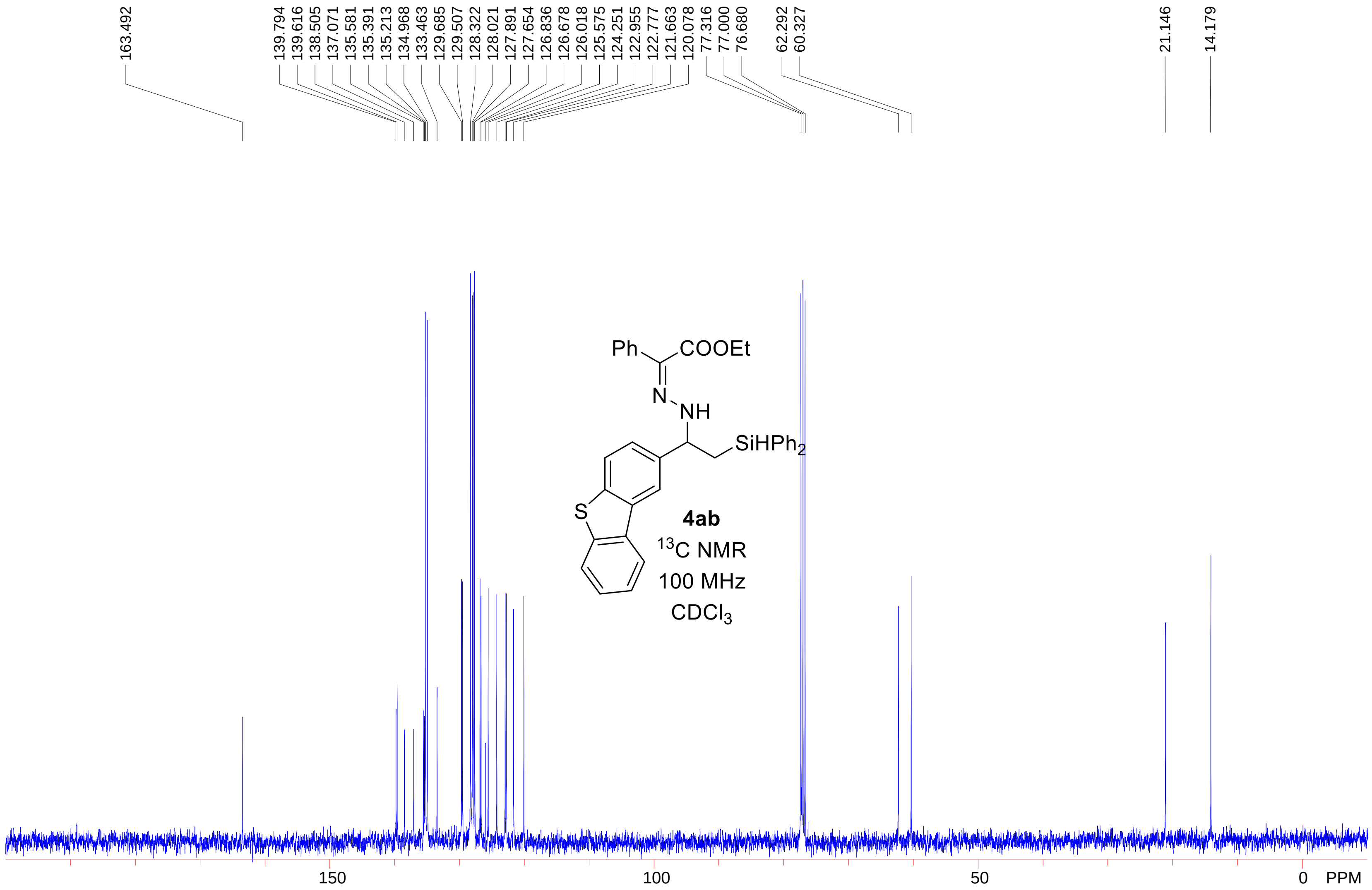

Supplementary Figure 89. ^{13}C NMR spectrum of **4ab**.

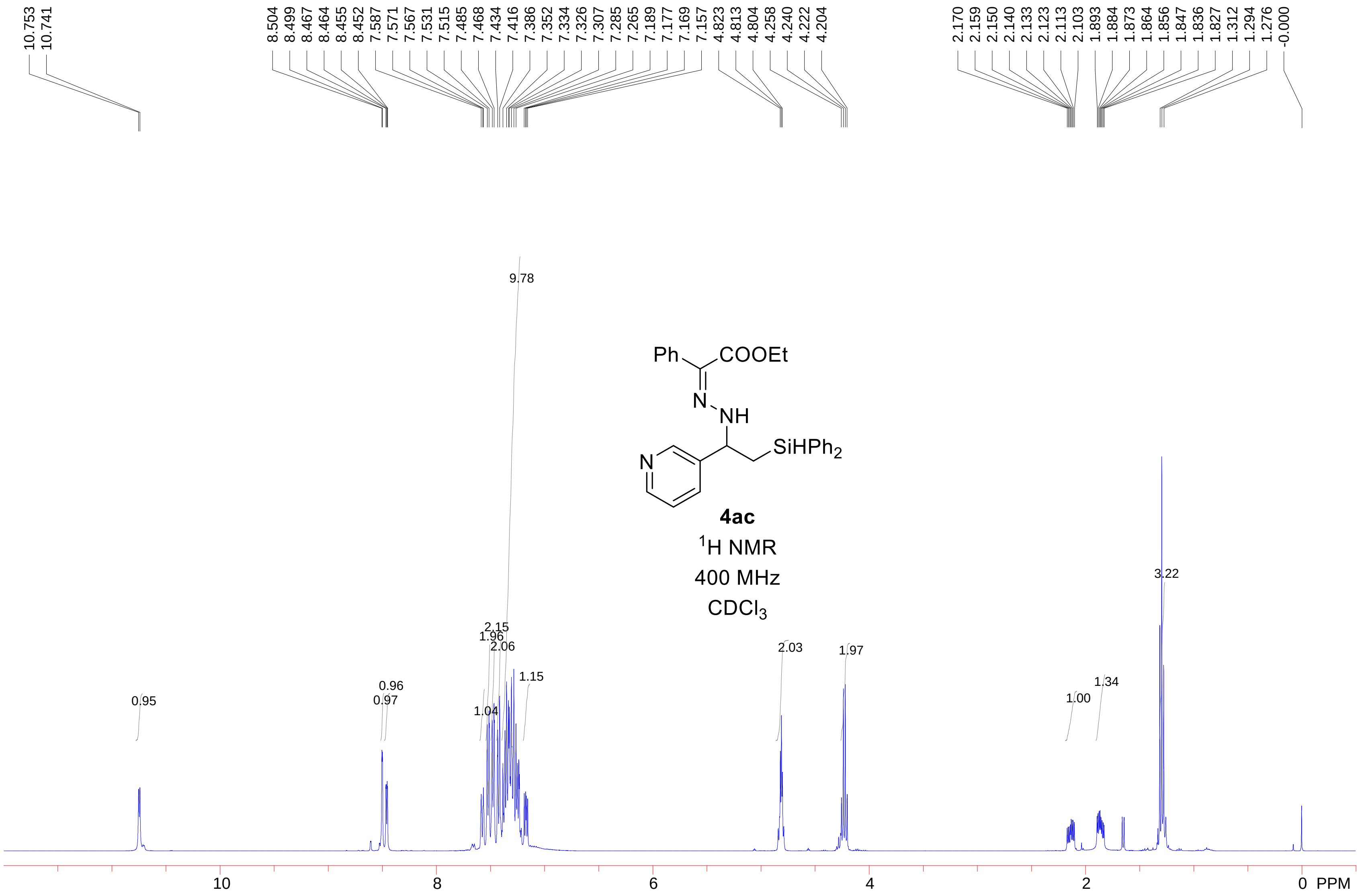

Supplementary Figure 90. ^{1}H NMR spectrum of **4ac**.

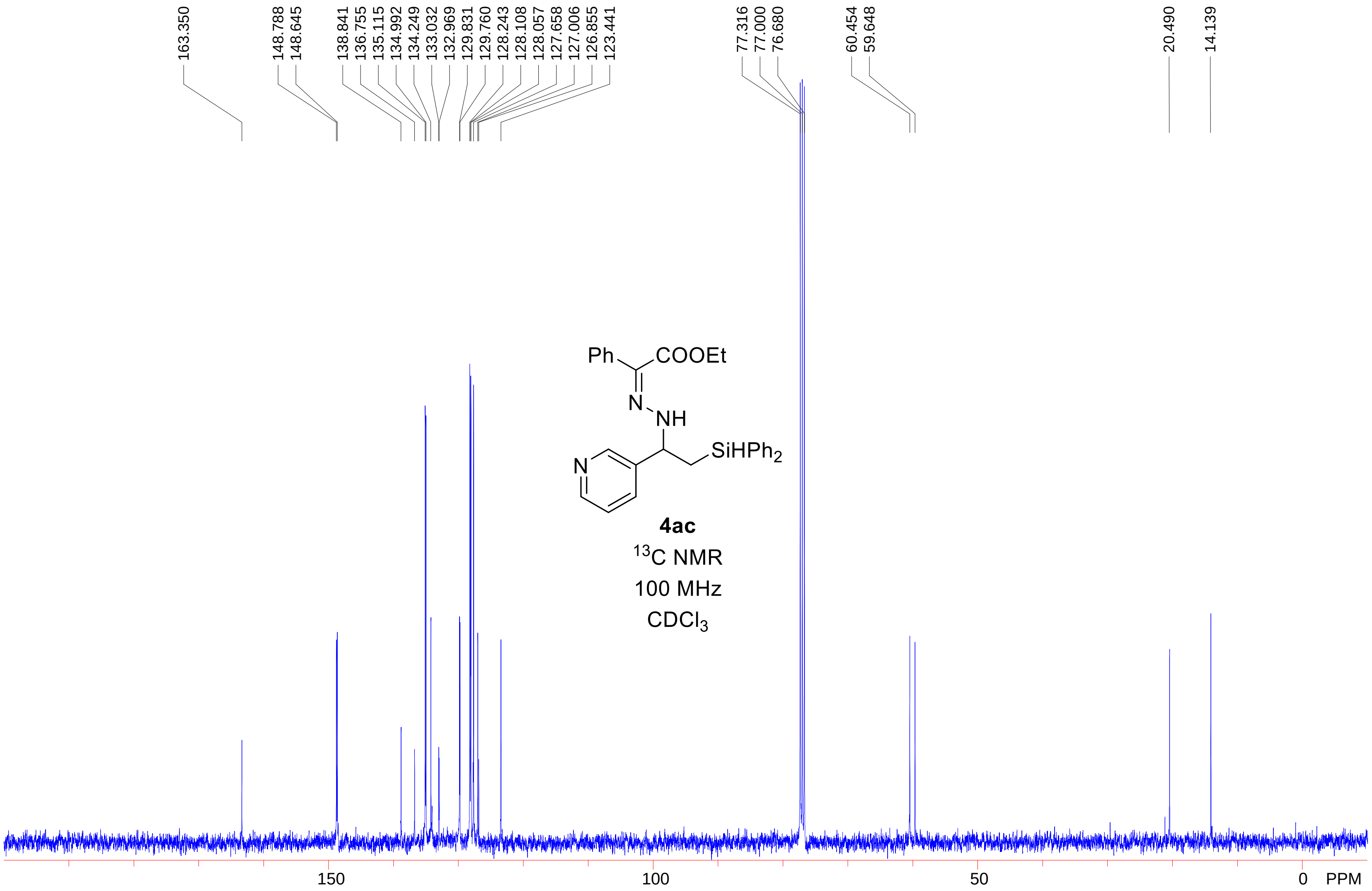

Supplementary Figure 91. ^{13}C NMR spectrum of **4ac**.

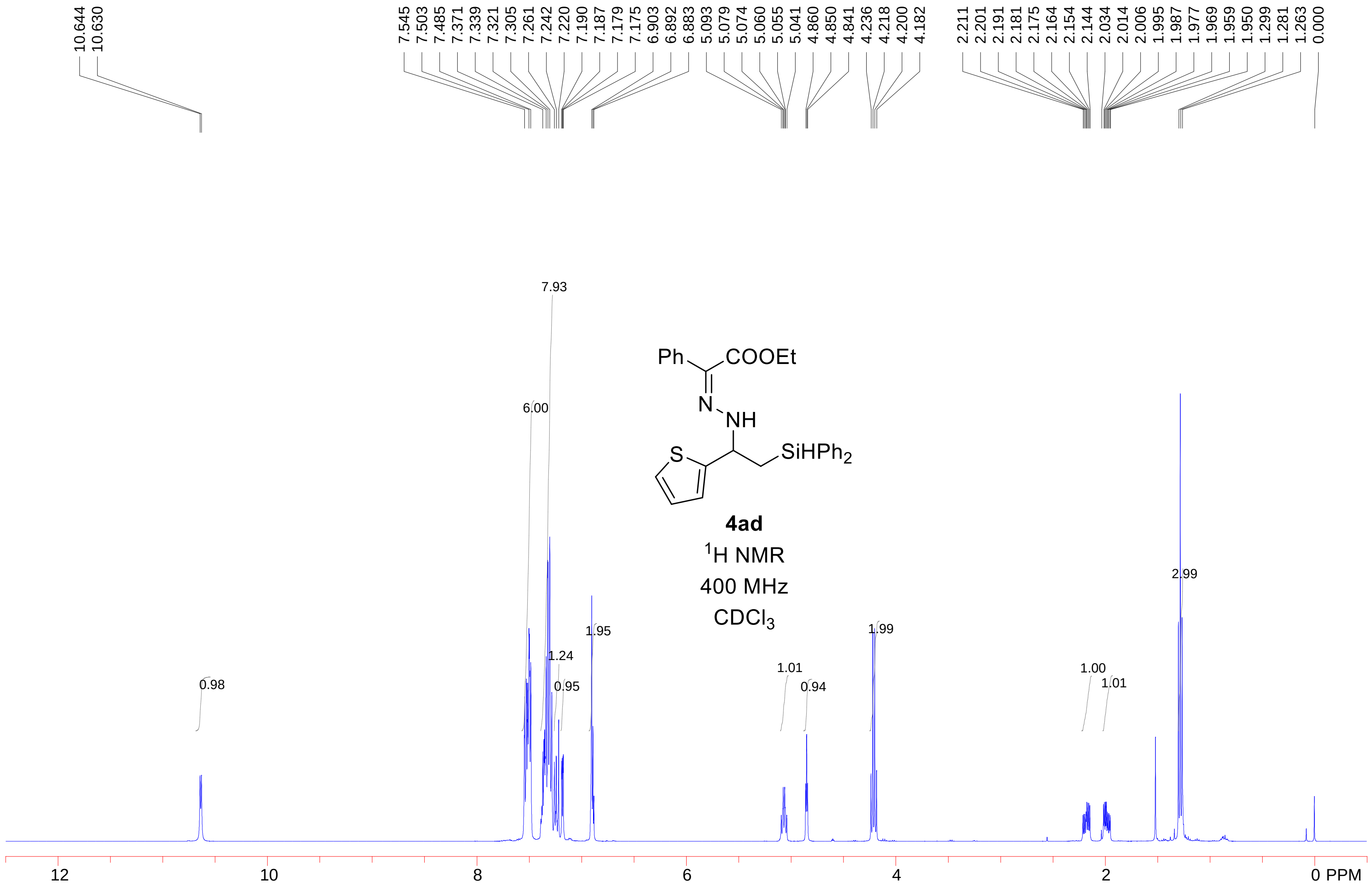

Supplementary Figure 92. ^{1}H NMR spectrum of **4ad**.

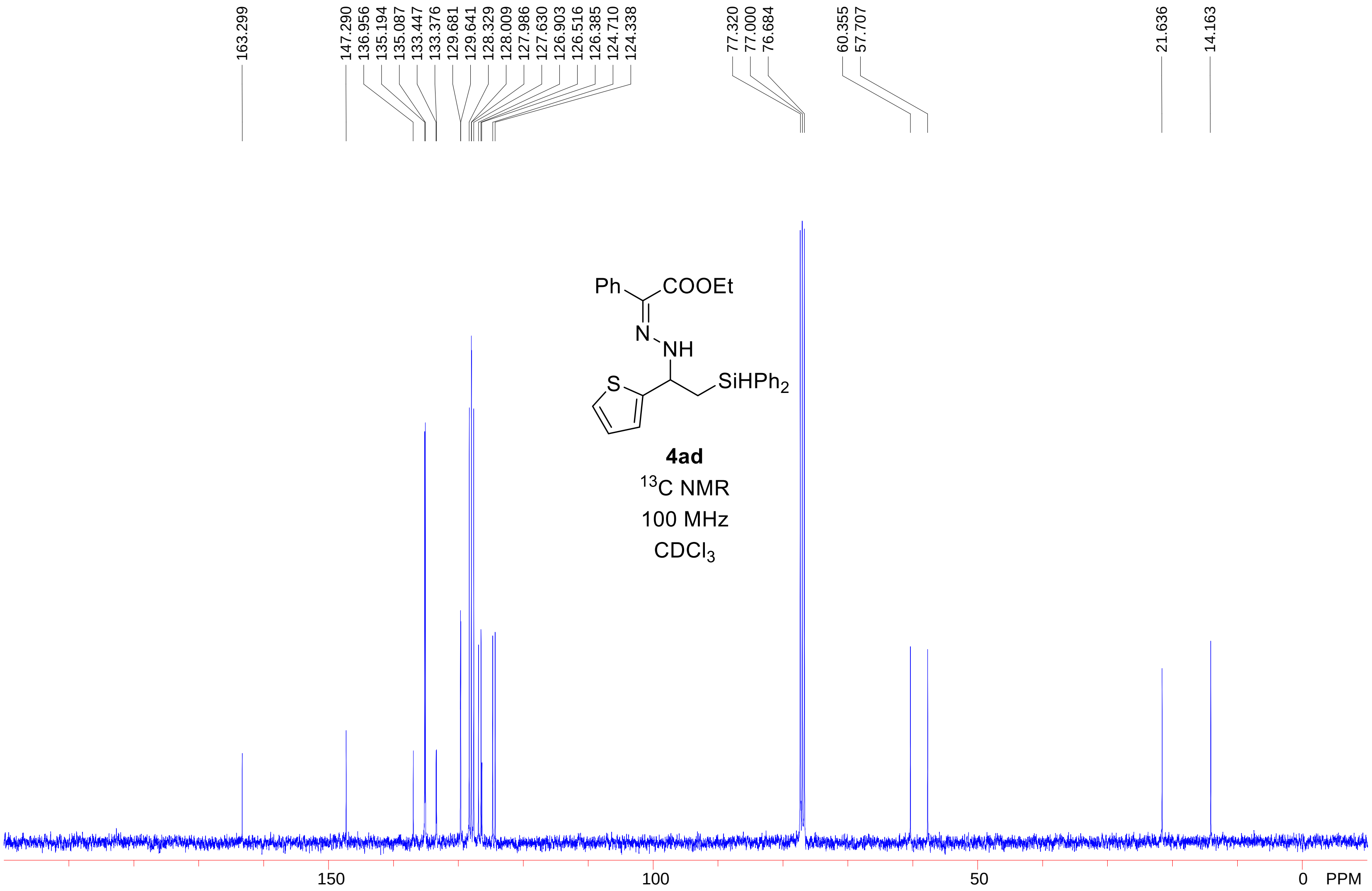

Supplementary Figure 93. ^{13}C NMR spectrum of **4ad**.

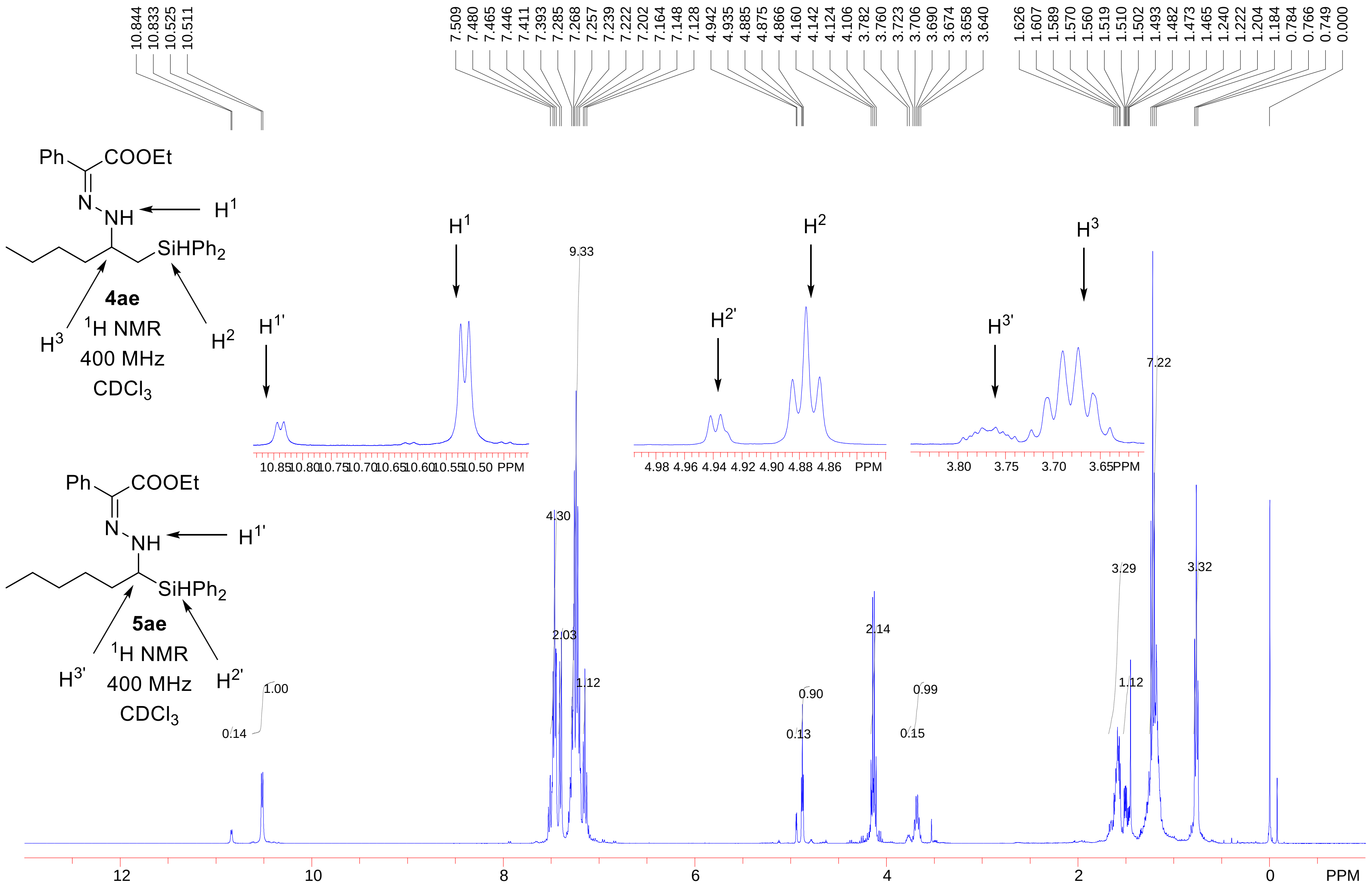

Supplementary Figure 94. ^{1}H NMR spectra of **4ae** & **5ae**.

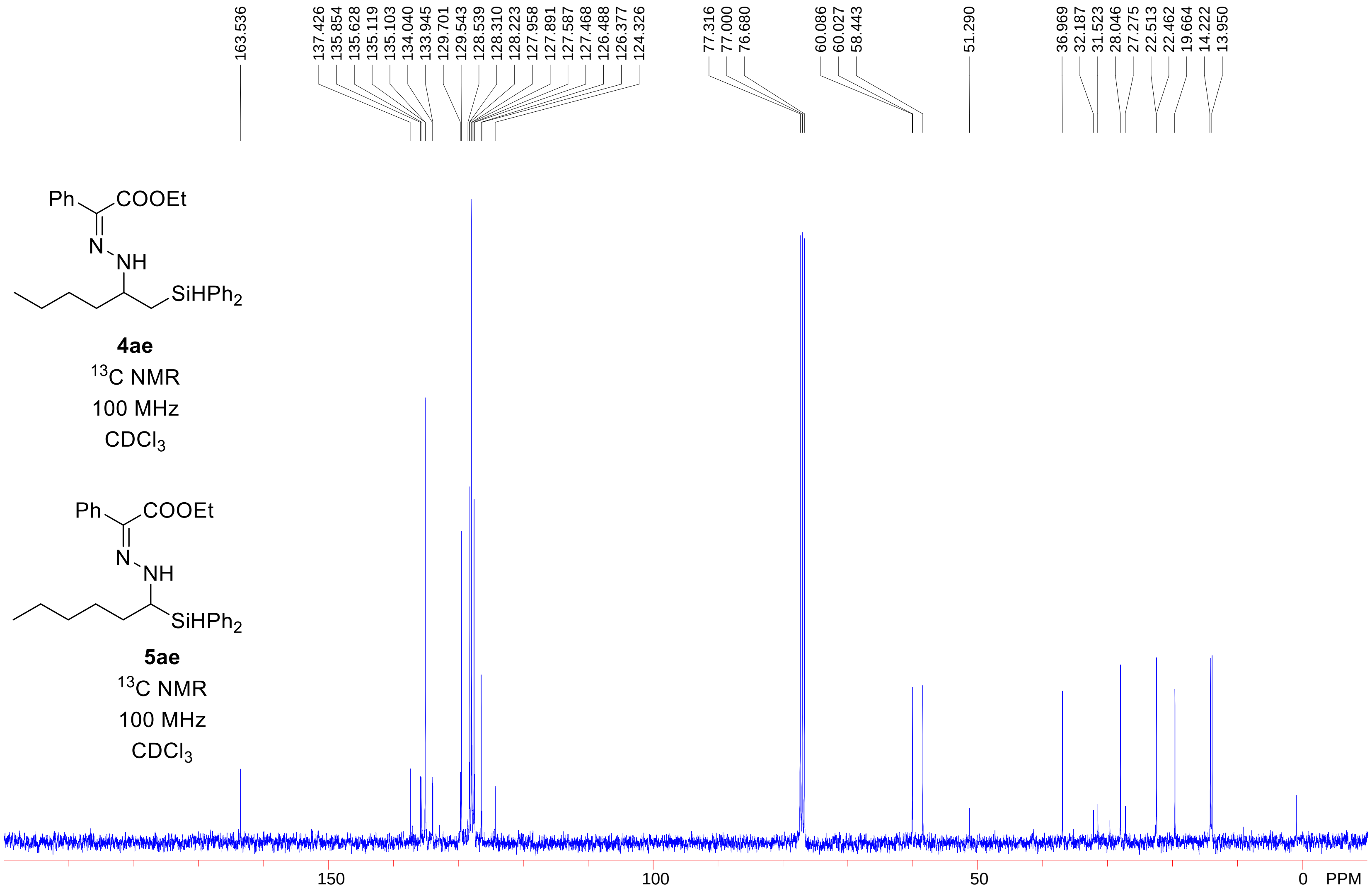

Supplementary Figure 95. ^{13}C NMR spectra of **4ae** & **5ae**.

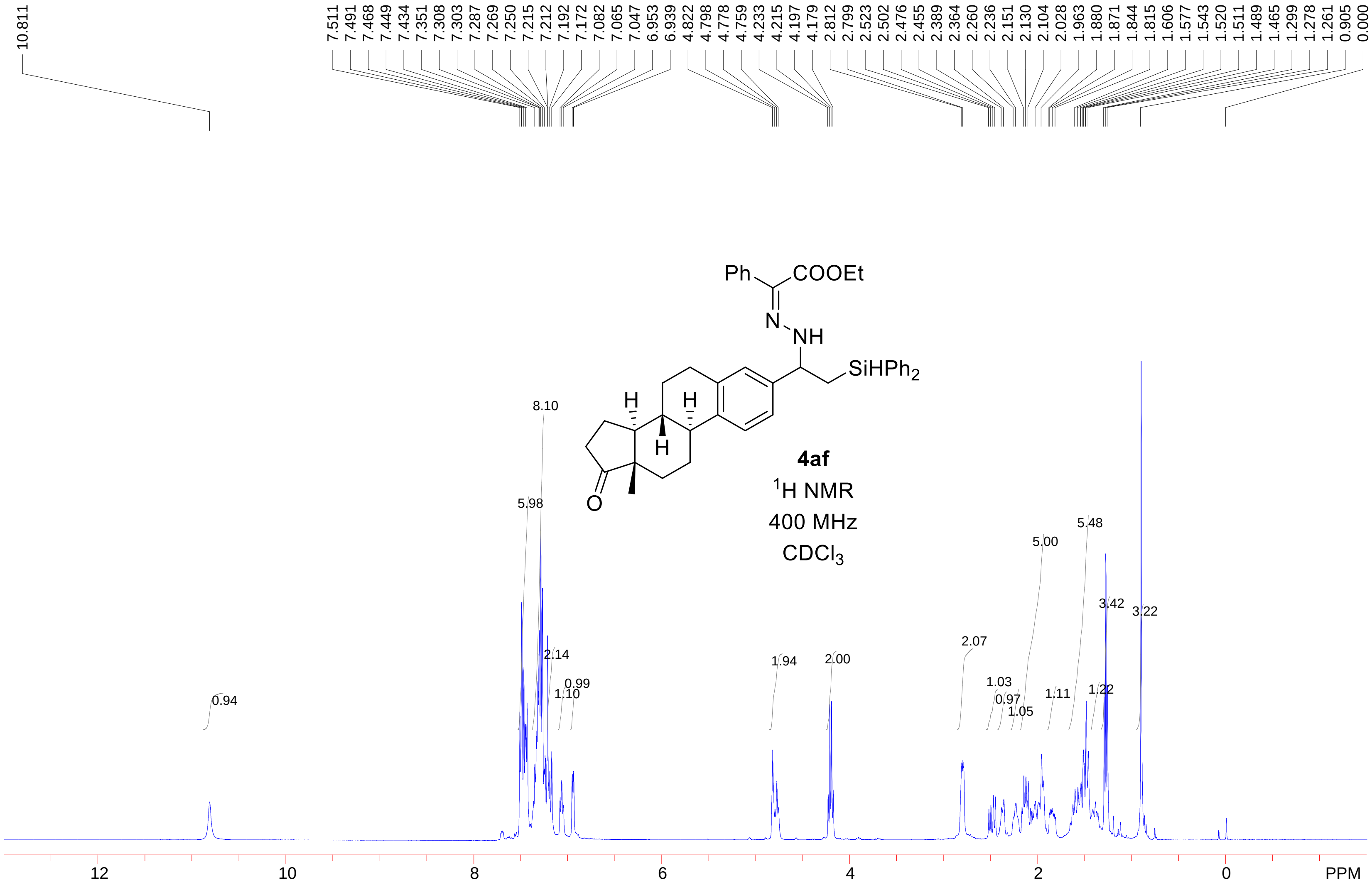

Supplementary Figure 96. ^{1}H NMR spectrum of **4af**.

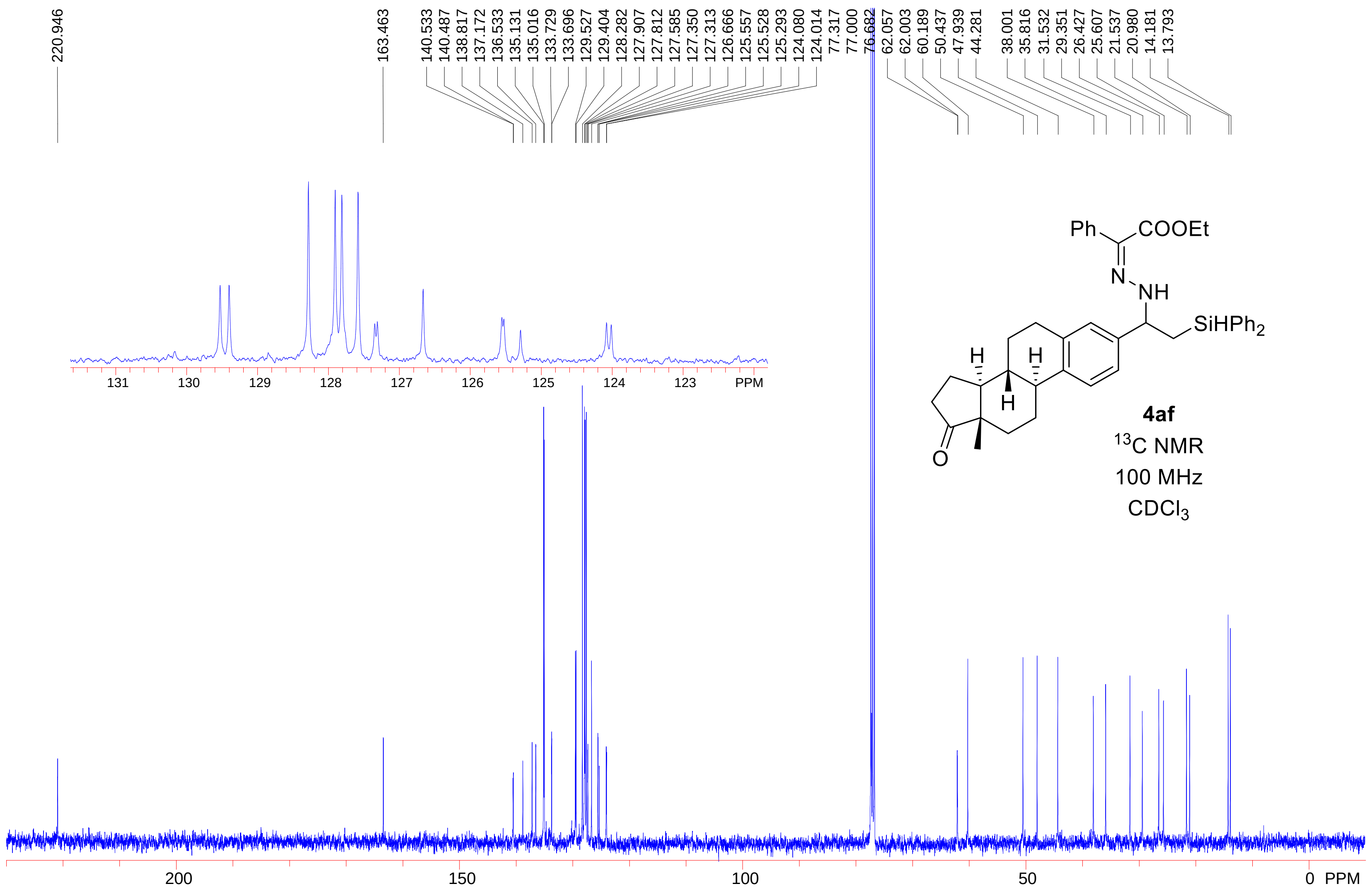

Supplementary Figure 97. ^{13}C NMR spectrum of **4af**.

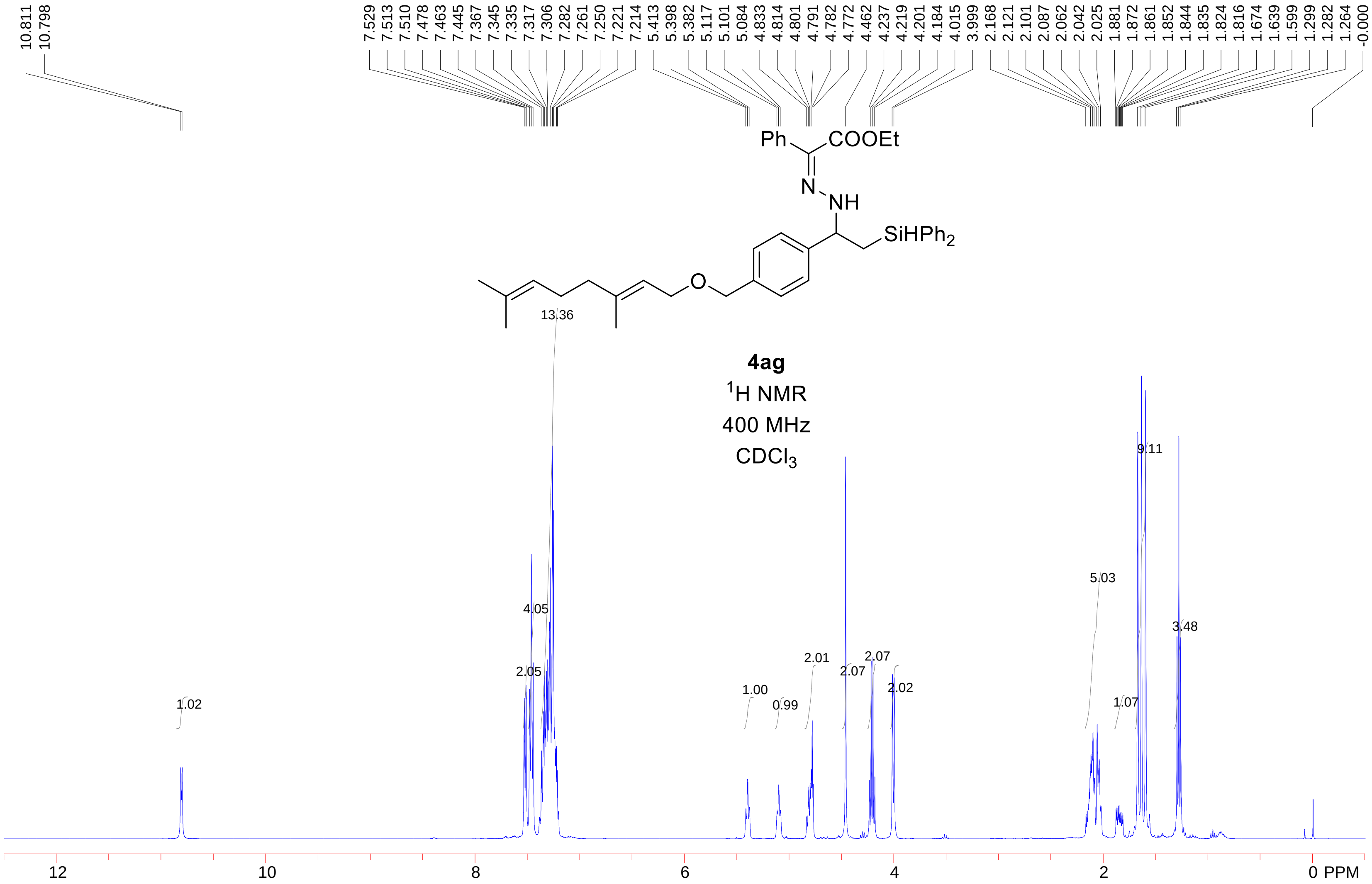

Supplementary Figure 98. ^{1}H NMR spectrum of **4ag**.

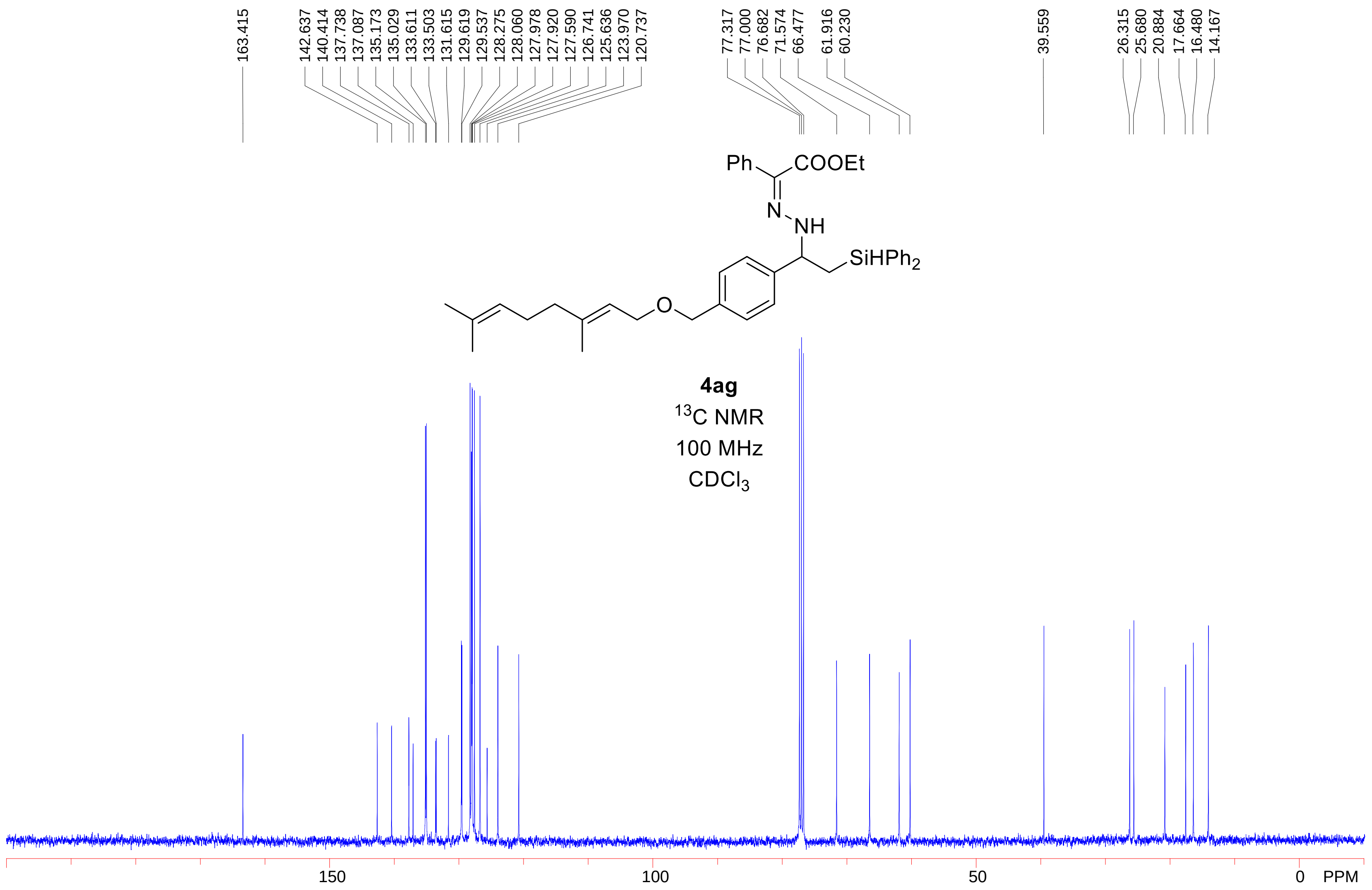

Supplementary Figure 99. ^{13}C NMR spectrum of **4ag**.

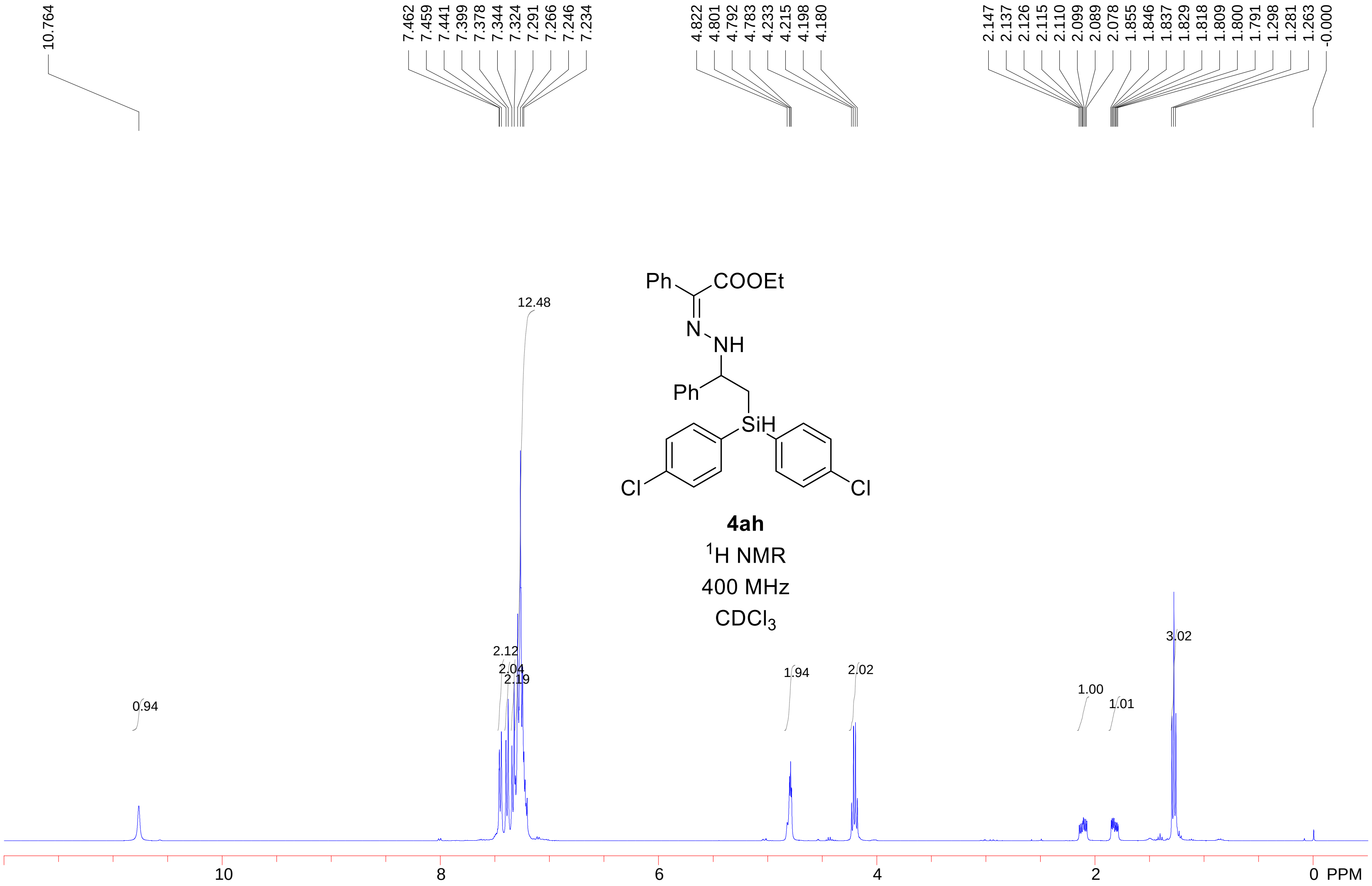

Supplementary Figure 100. ^{1}H NMR spectrum of **4ah**.

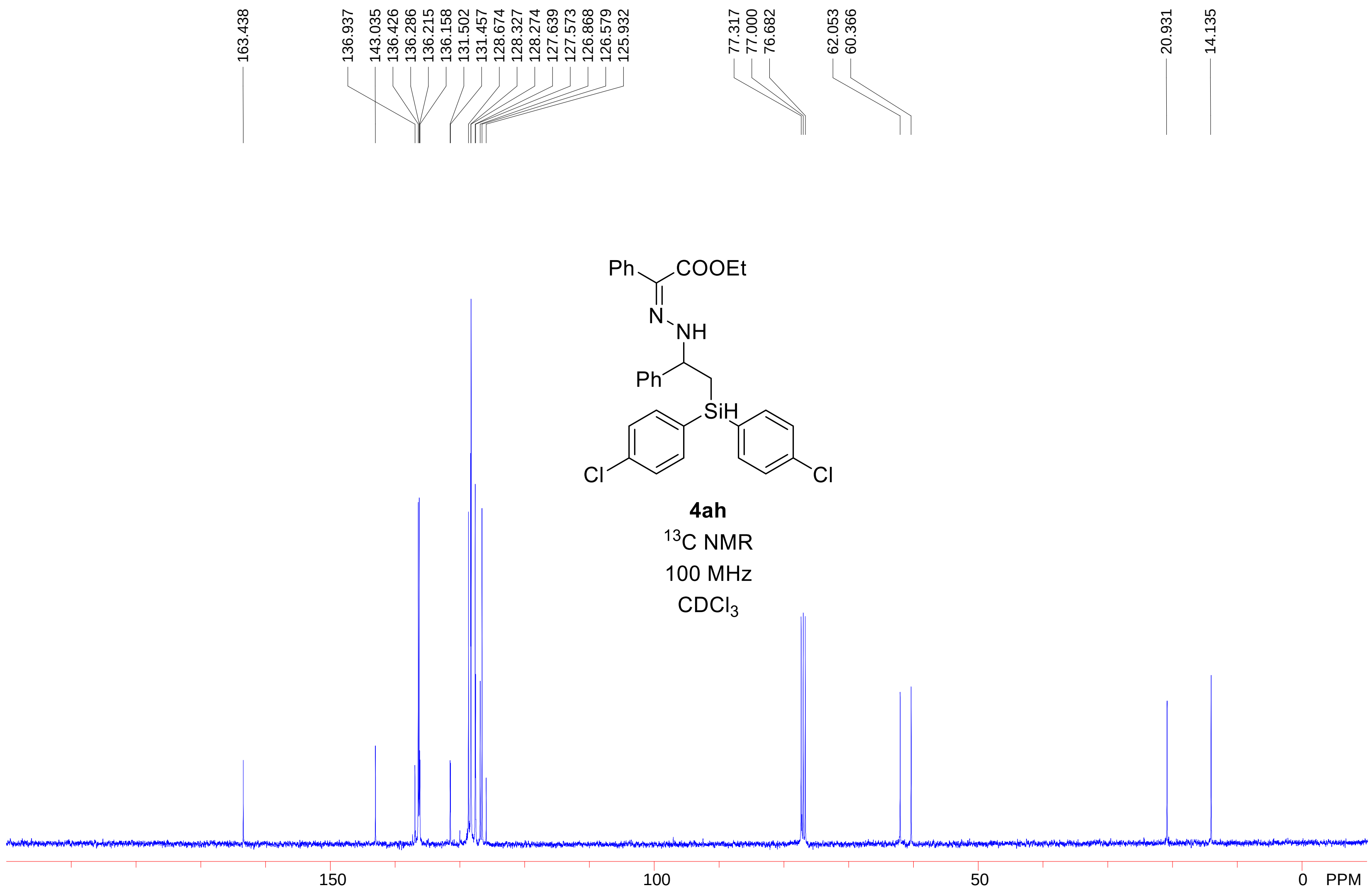

Supplementary Figure 101. ^{13}C NMR spectrum of **4ah**.

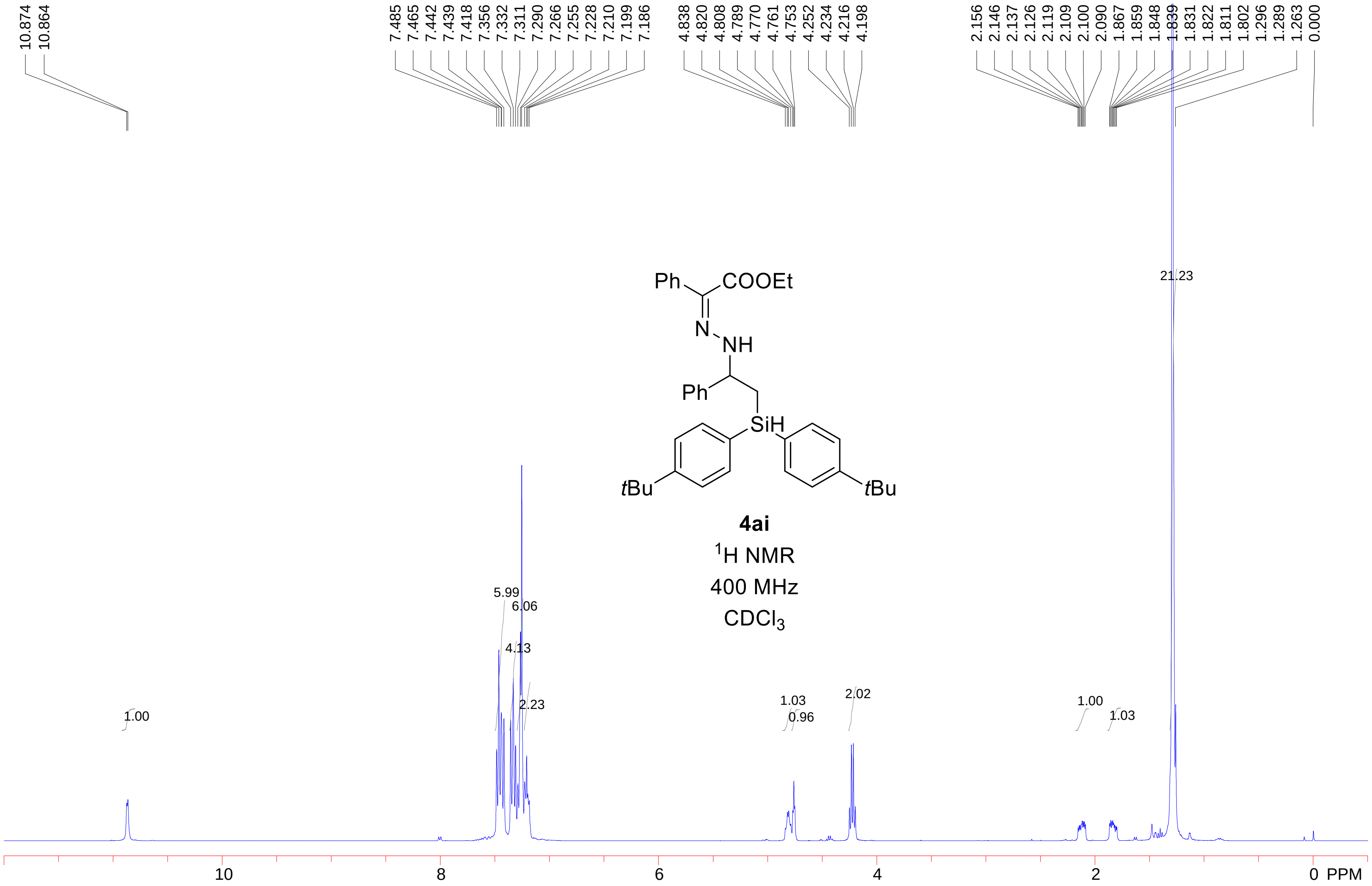

Supplementary Figure 102. ^{1}H NMR spectrum of **4ai**.

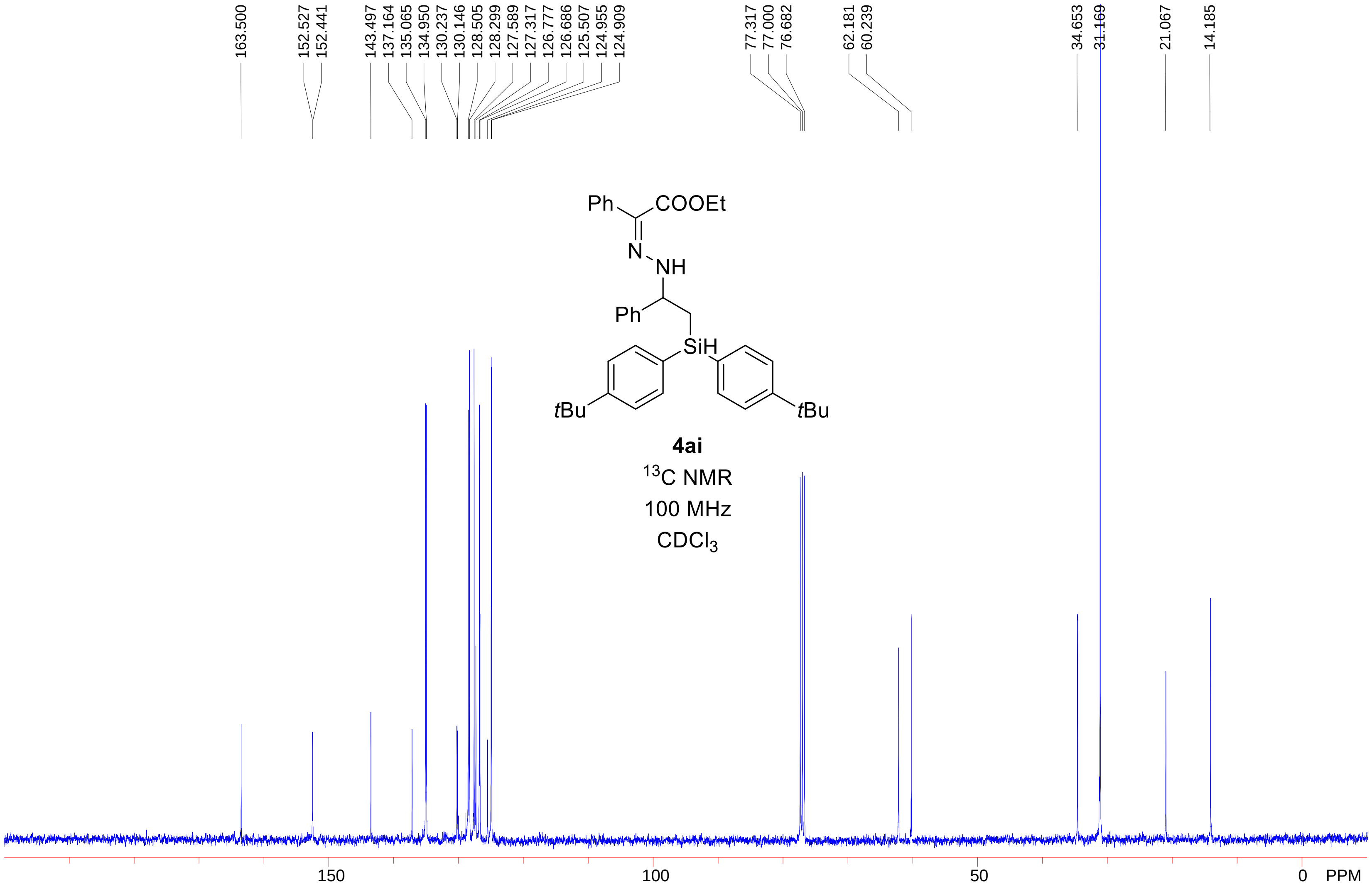

Supplementary Figure 103. ^{13}C NMR spectrum of **4ai**.

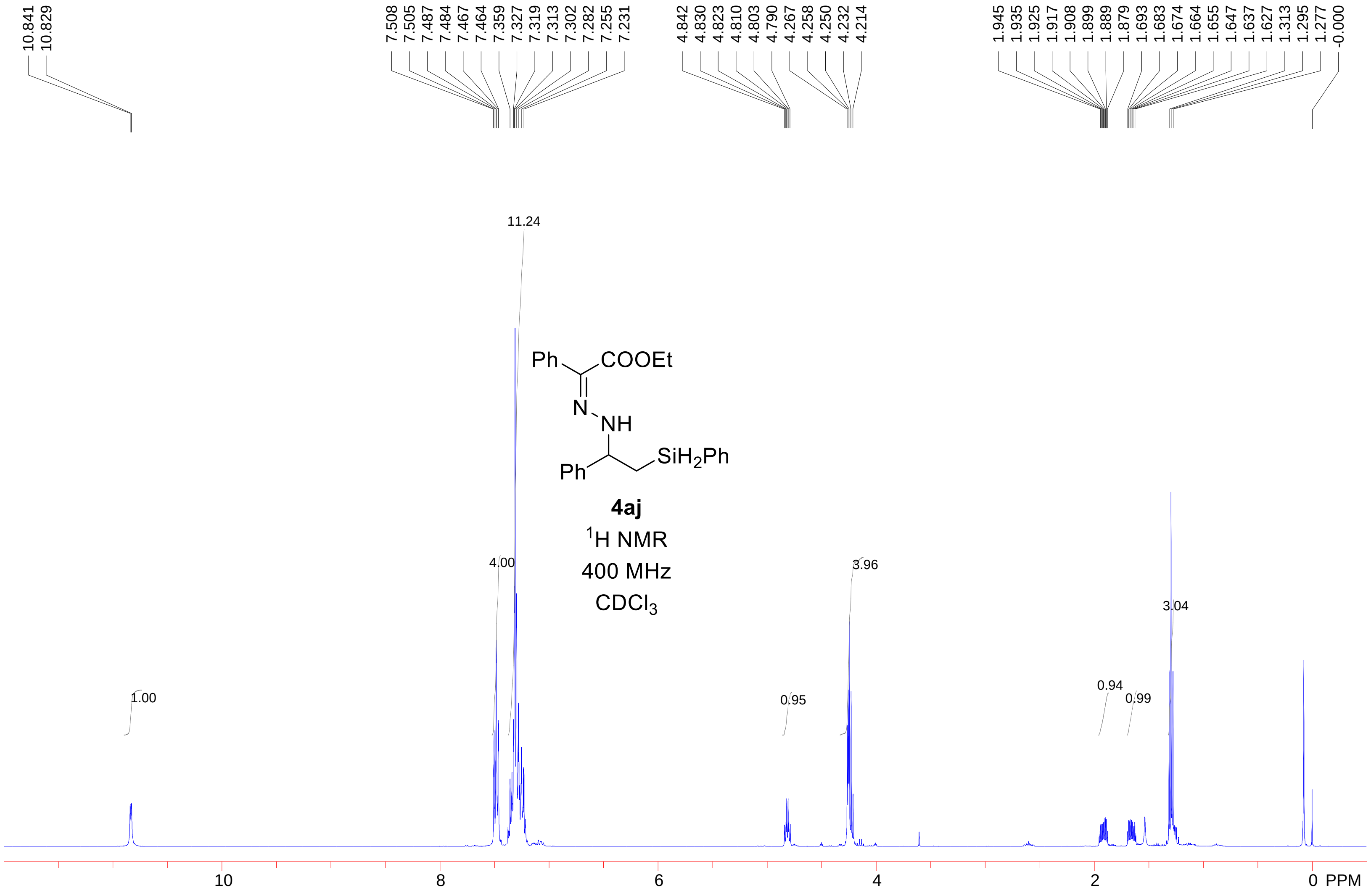

Supplementary Figure 104. ^{1}H NMR spectrum of **4aj**.

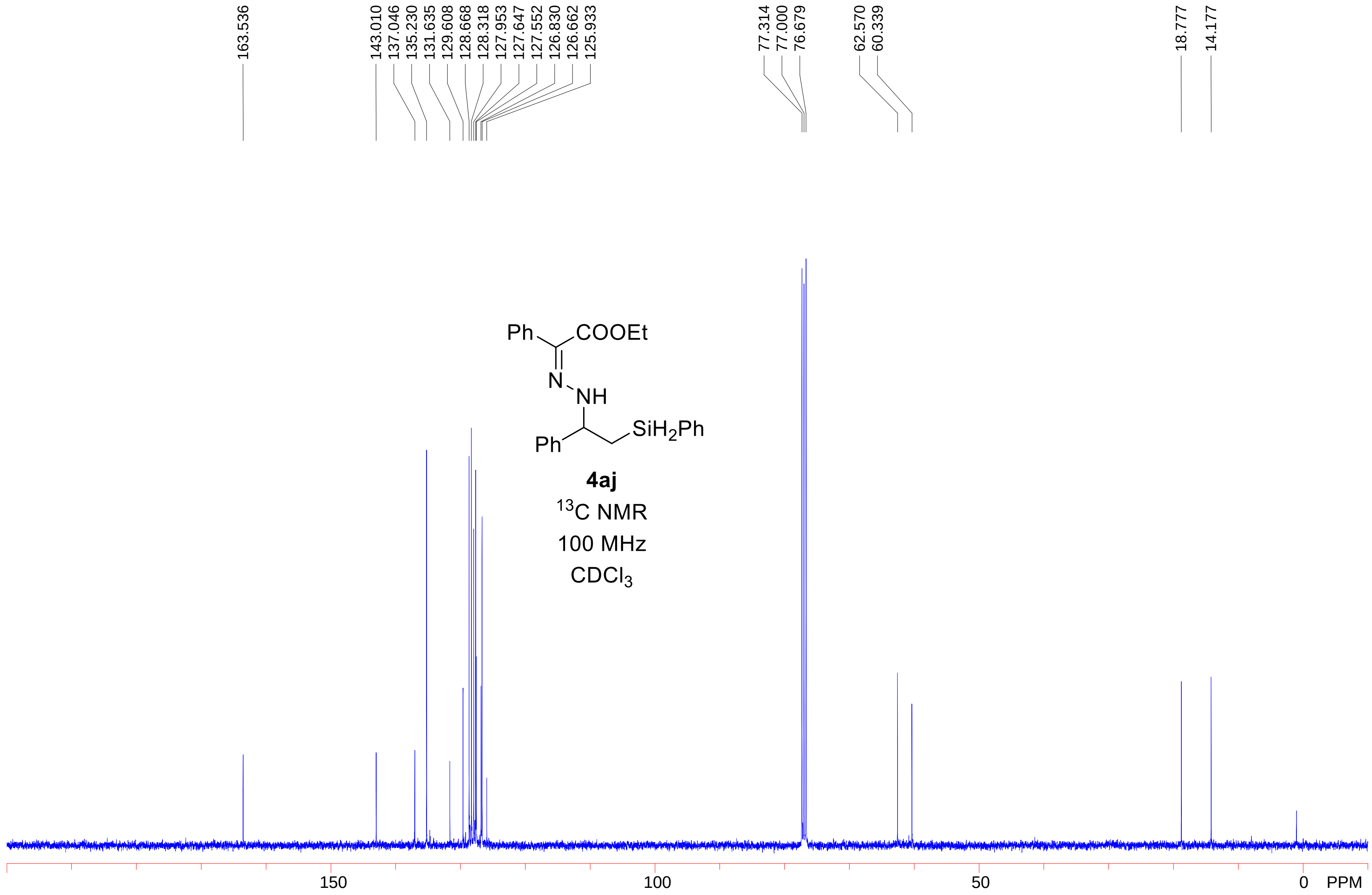

Supplementary Figure 105. ^{13}C NMR spectrum of **4aj**.

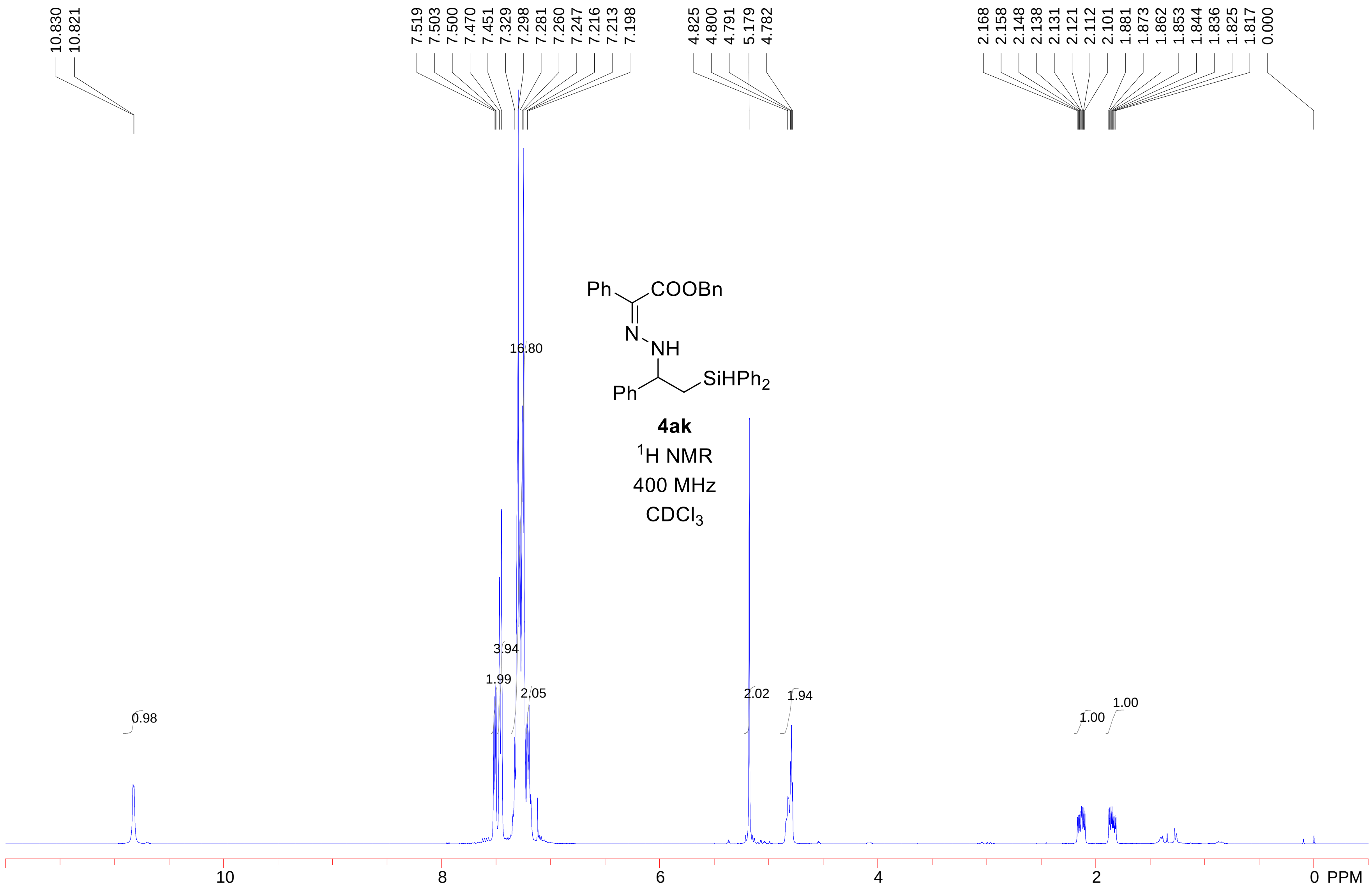

Supplementary Figure 106. ^{1}H NMR spectrum of **4ak**.

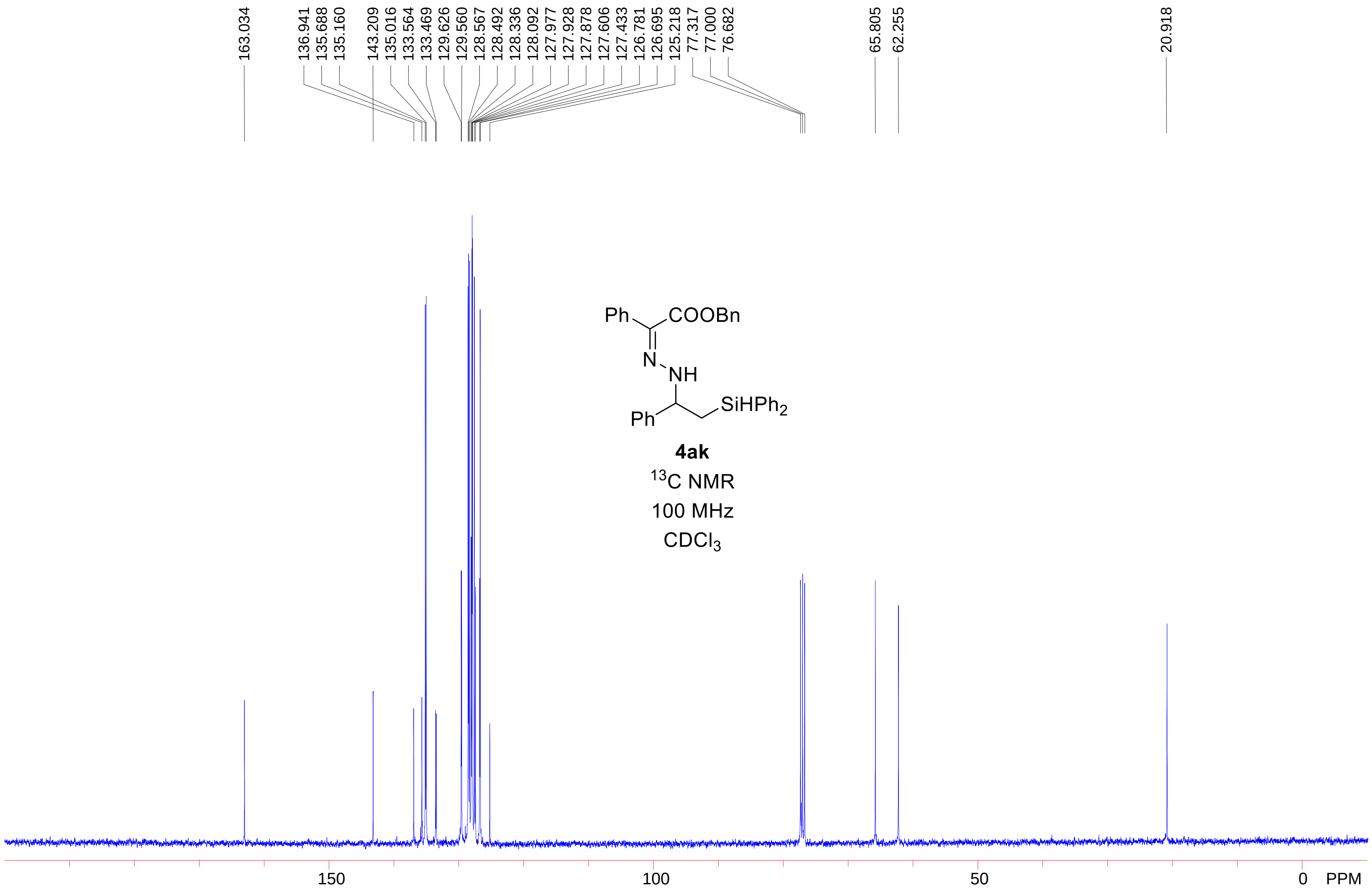

Supplementary Figure 107. ^{13}C NMR spectrum of **4ak**.

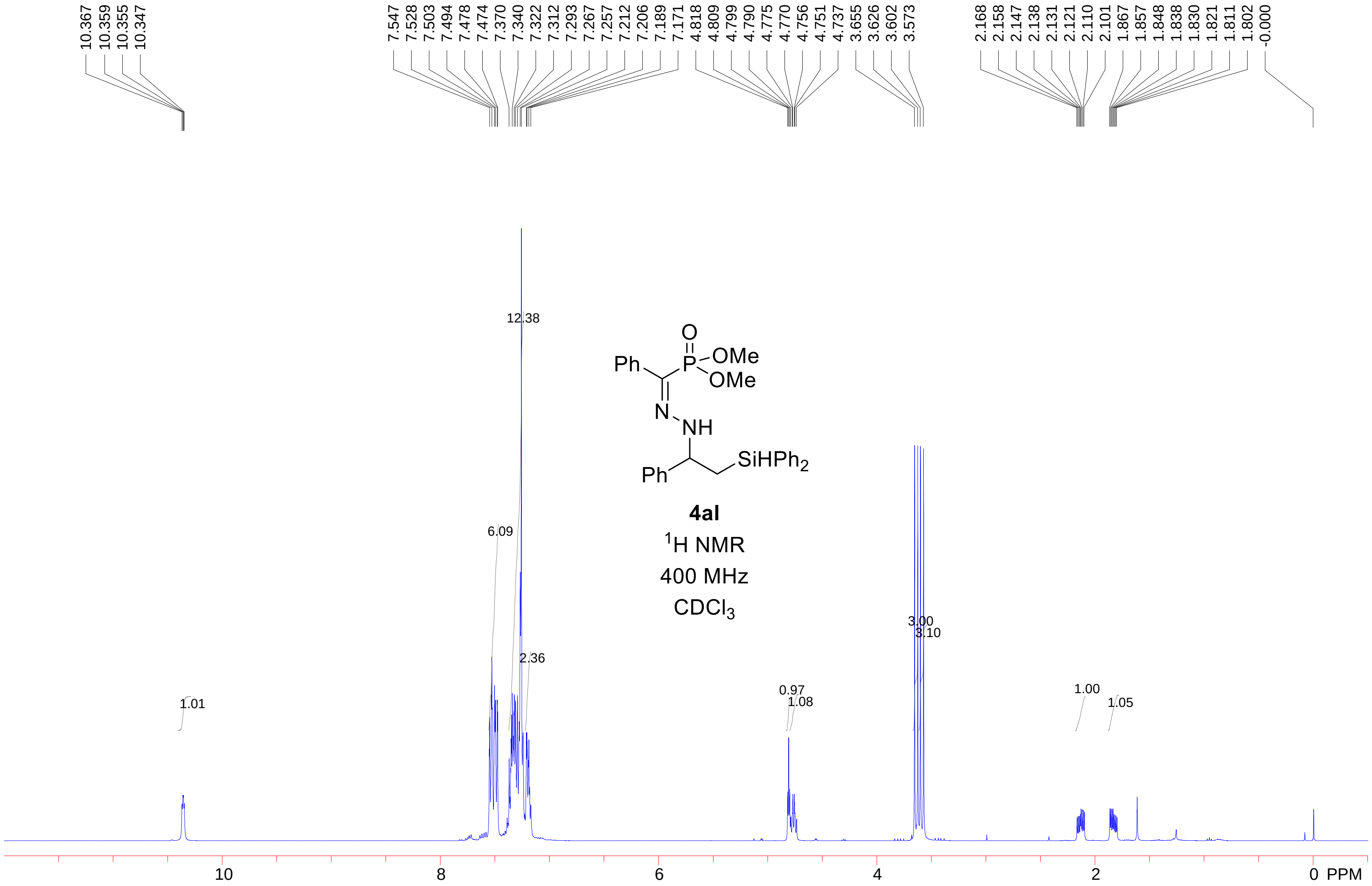

Supplementary Figure 108. ^{1}H NMR spectrum of **4al**.

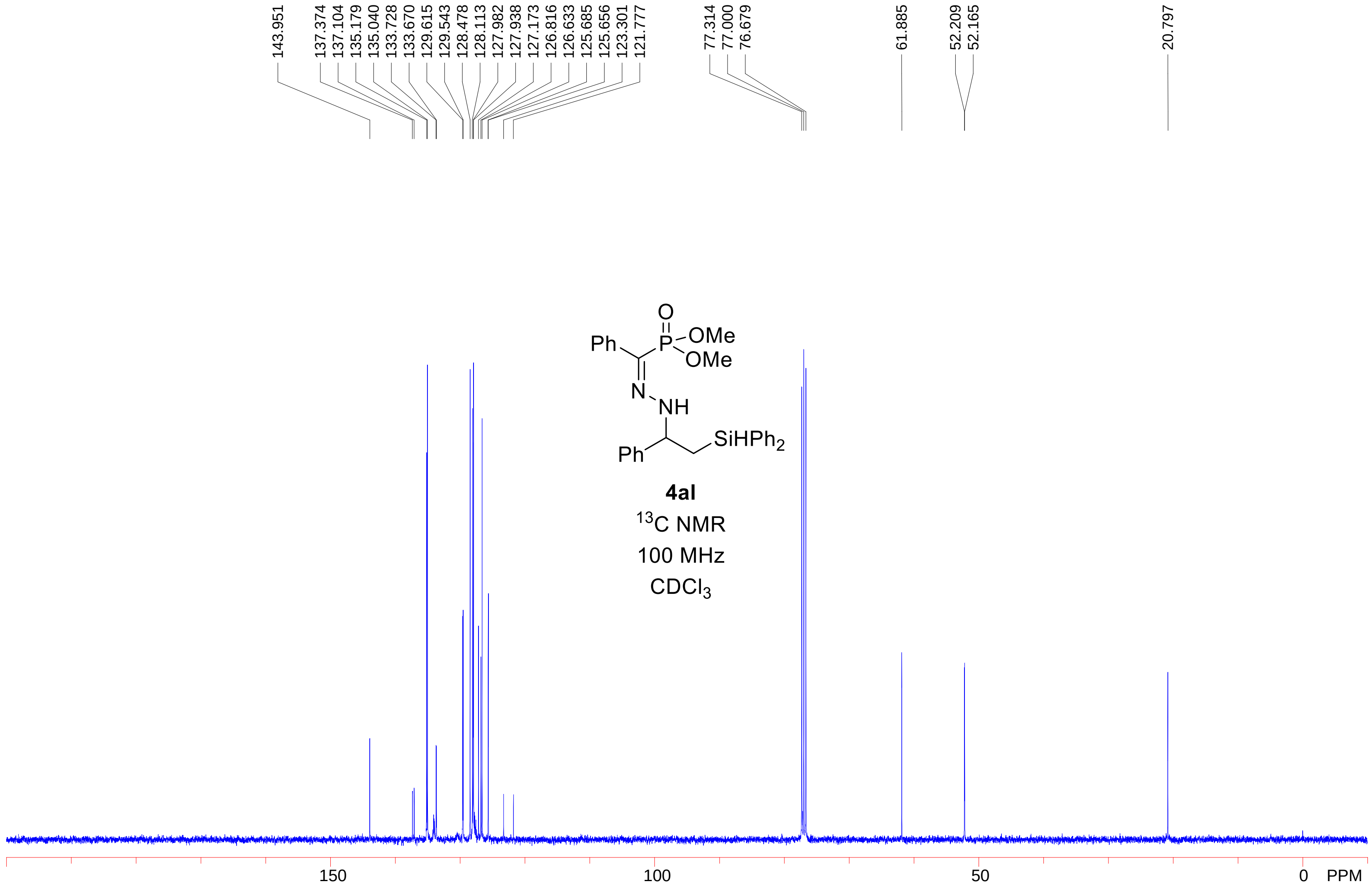

Supplementary Figure 109. ^{13}C NMR spectrum of **4al**.

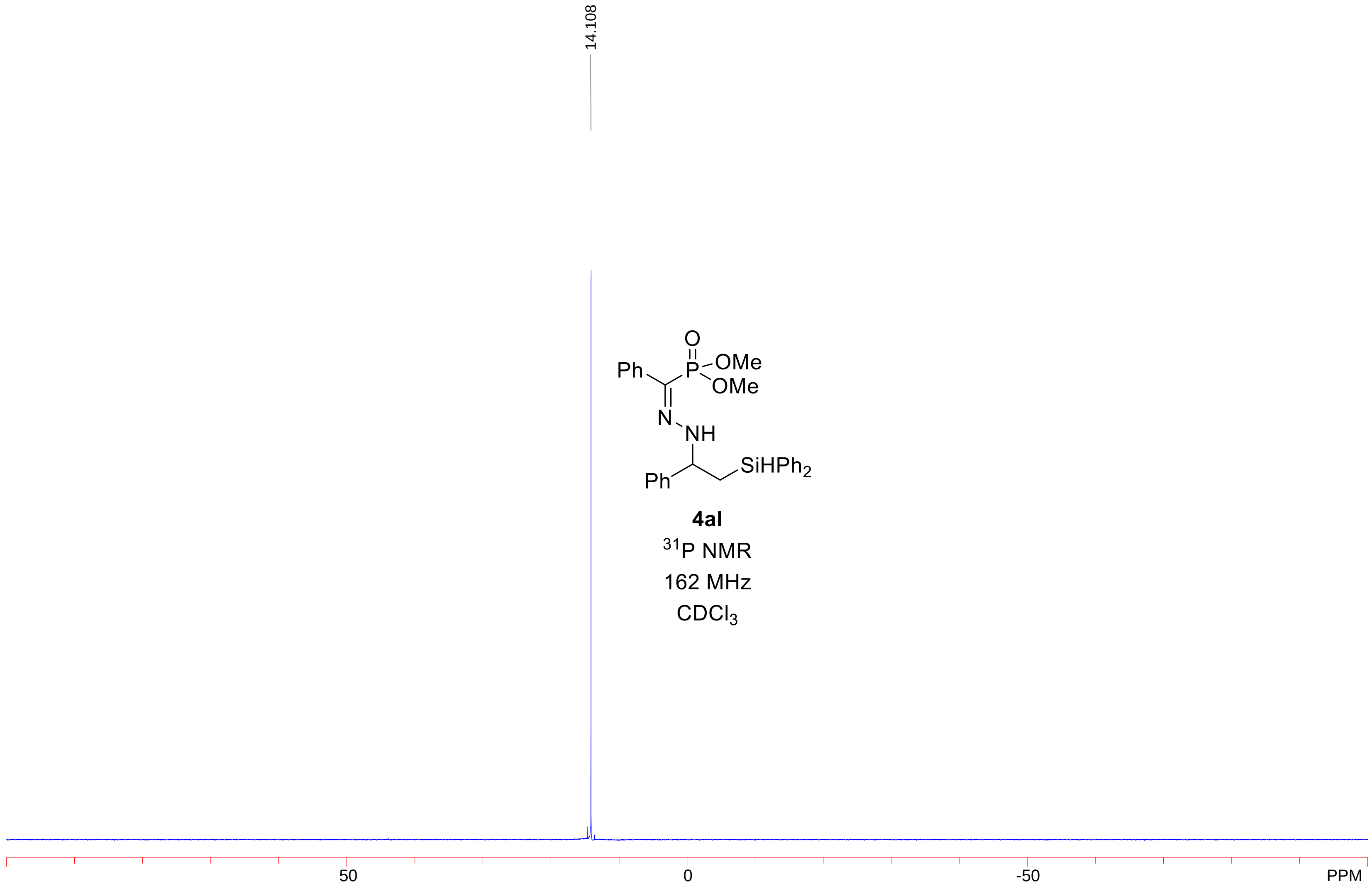

Supplementary Figure 110. ^{31}P NMR spectrum of **4al**.

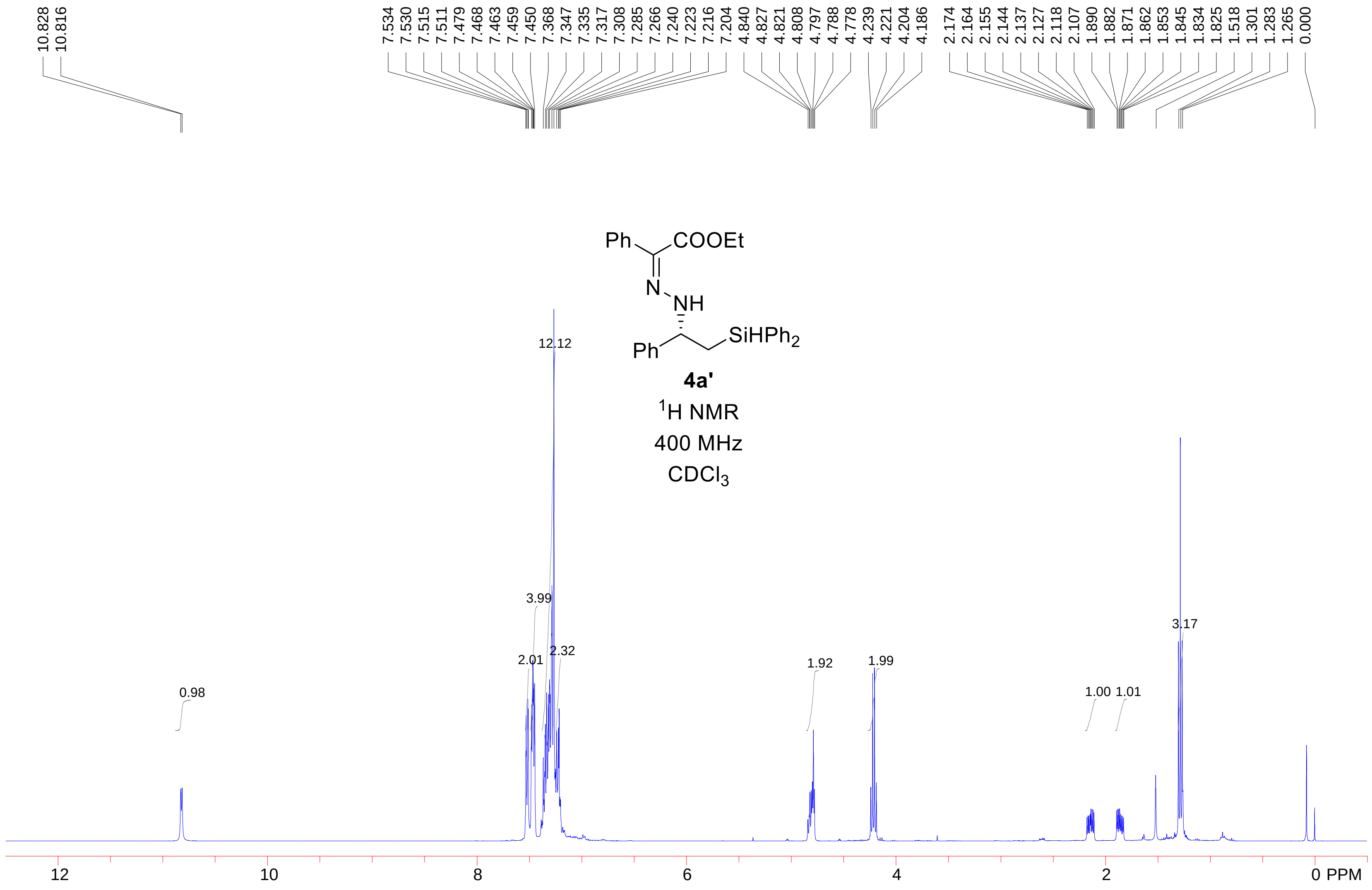

Supplementary Figure 111. ^{1}H NMR spectrum of **4a’**.

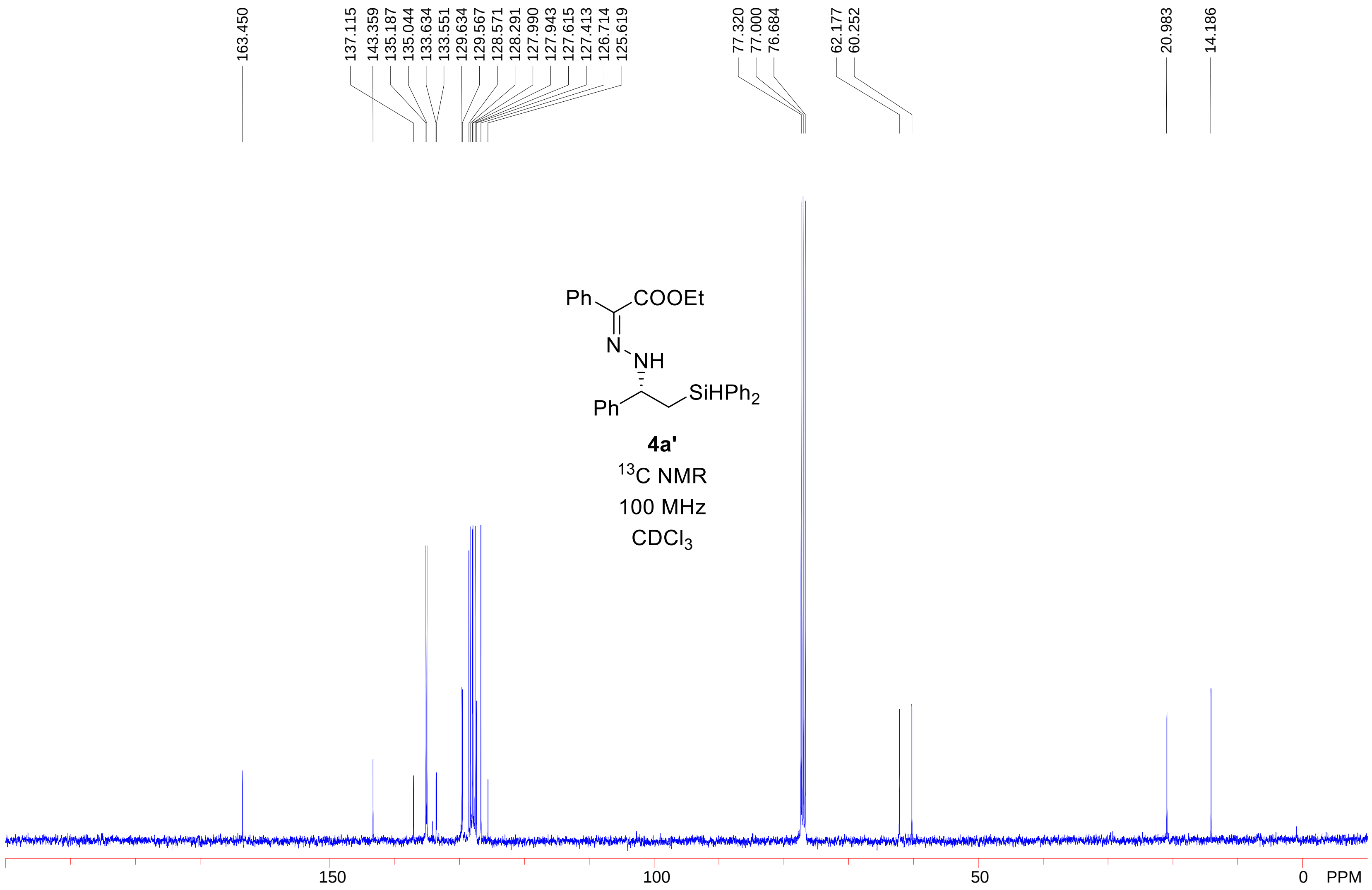

Supplementary Figure 112. ^{13}C NMR spectrum of **4a’**.

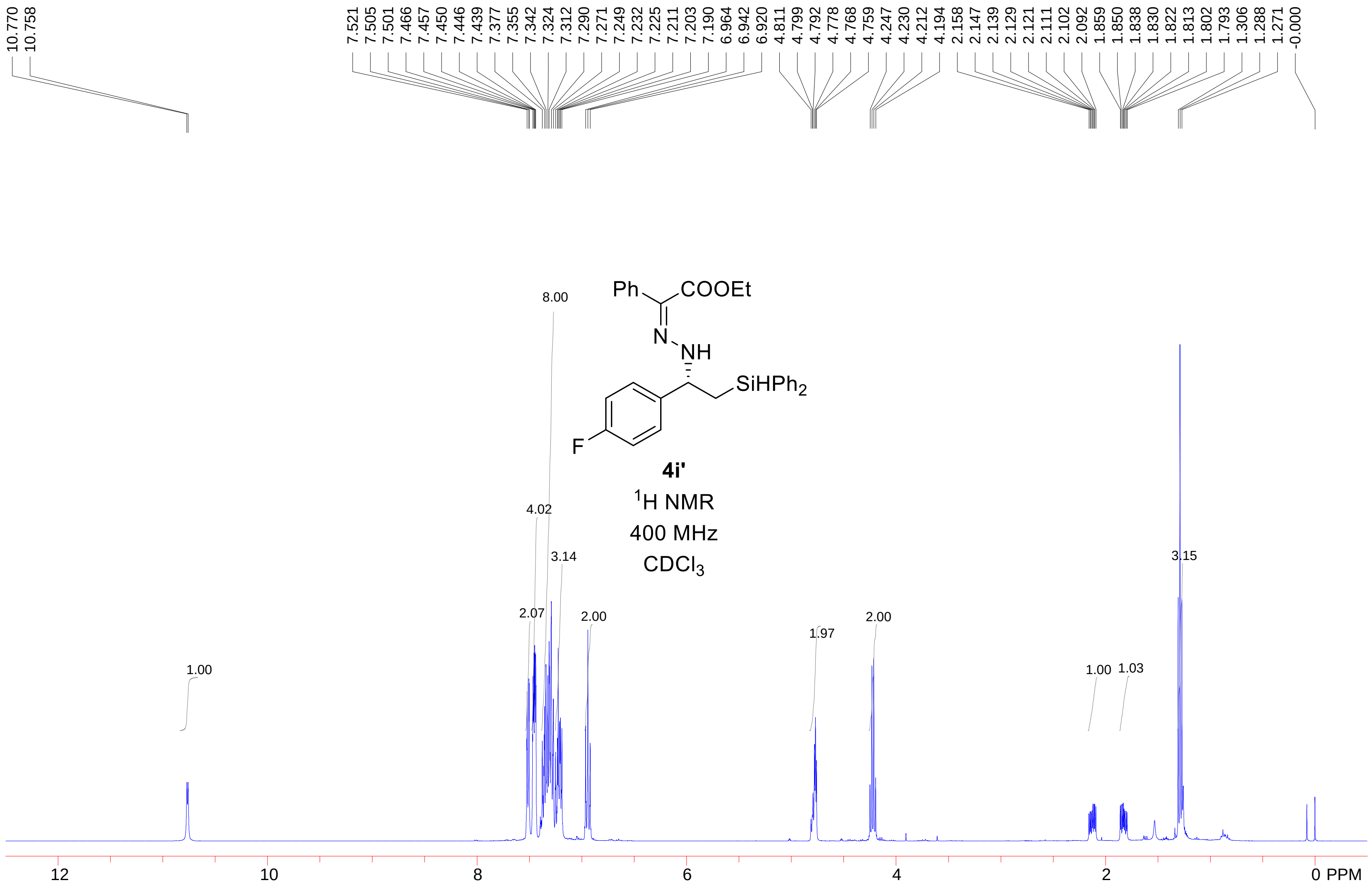

Supplementary Figure 113. ^{1}H NMR spectrum of **4i’**.

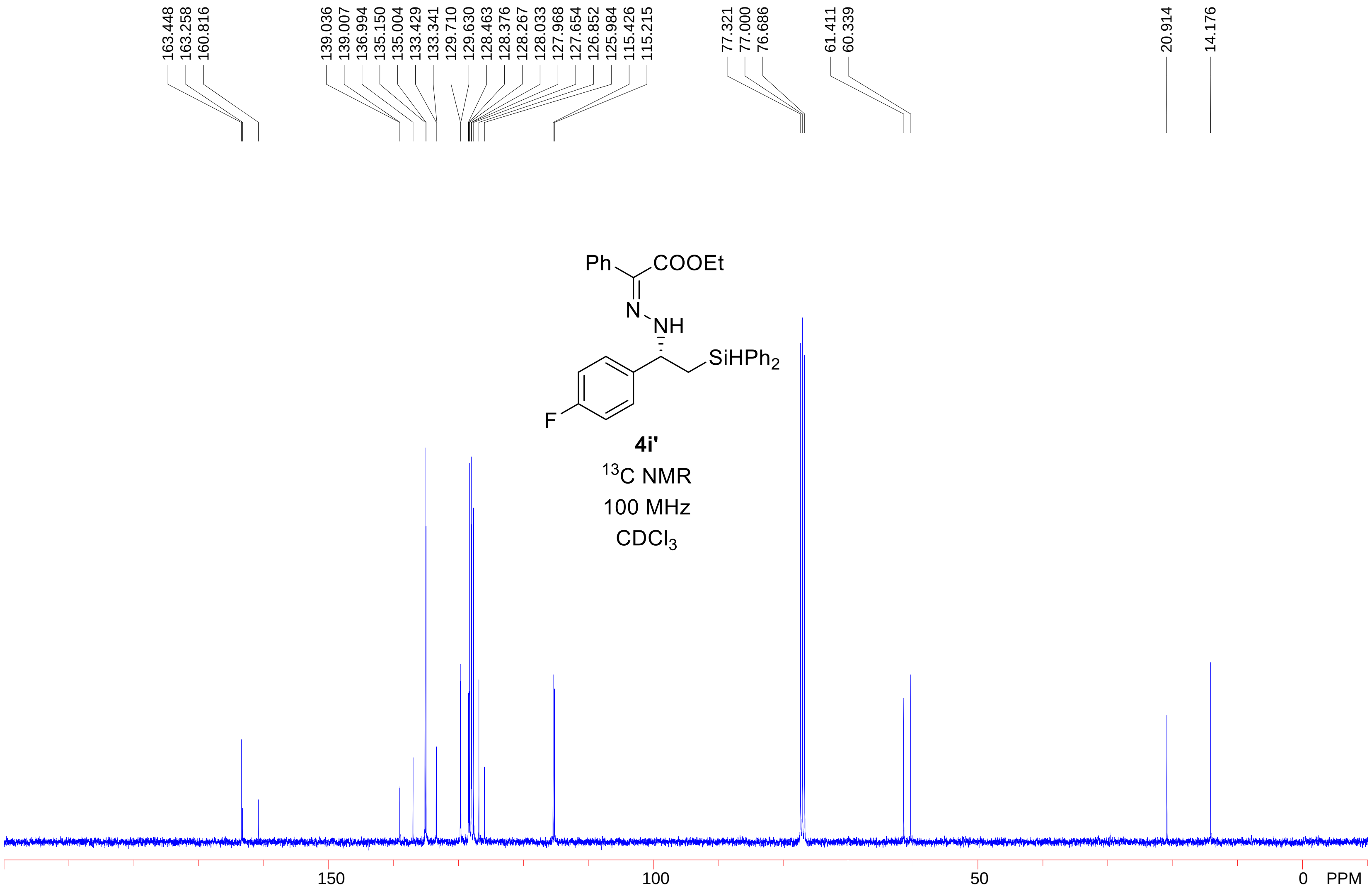

Supplementary Figure 114. ^{13}C NMR spectrum of **4i’**.

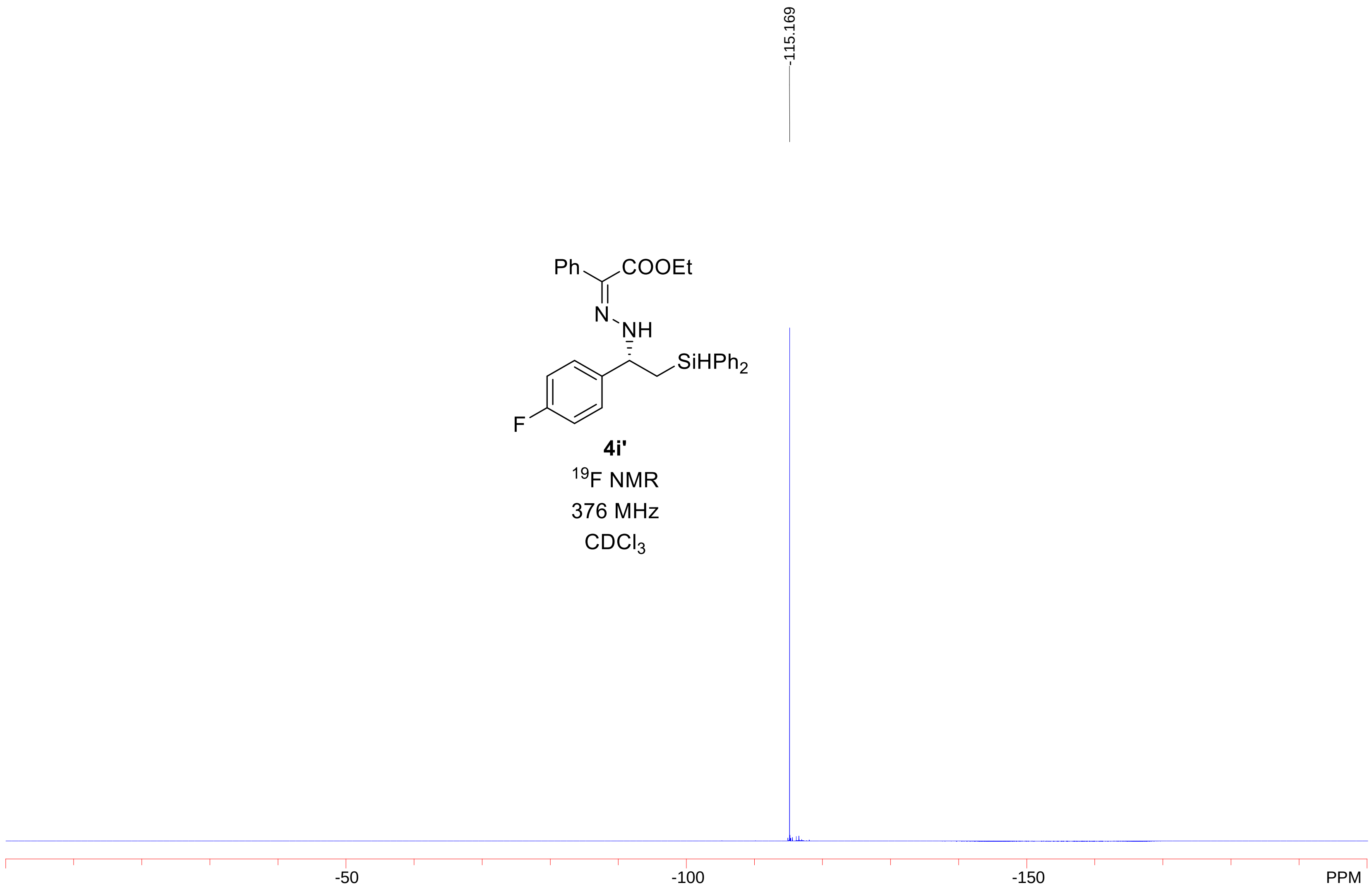

Supplementary Figure 115. ^{19}F NMR spectrum of **4i'**.

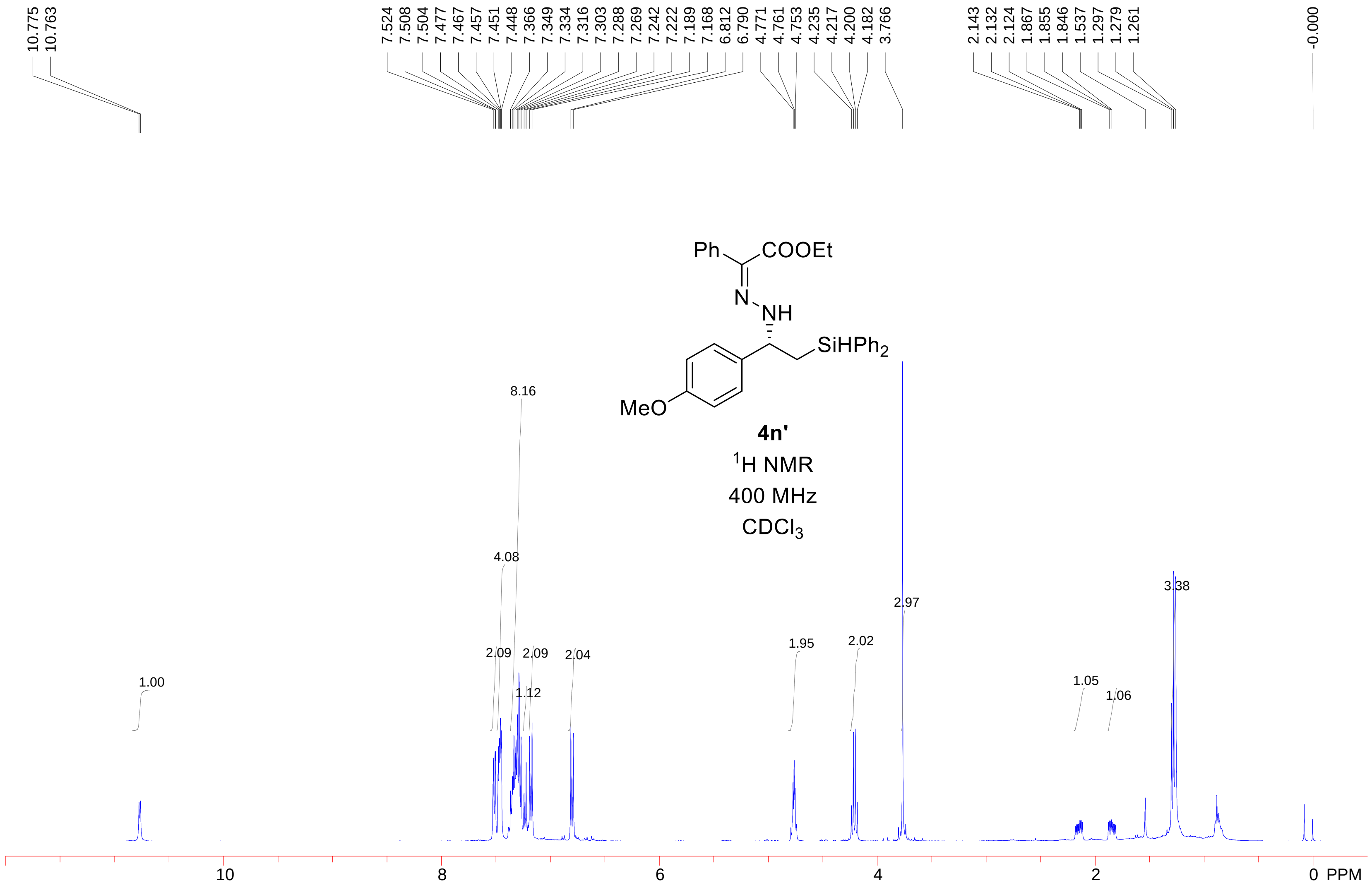

Supplementary Figure 116. ^{1}H NMR spectrum of **4n'**.

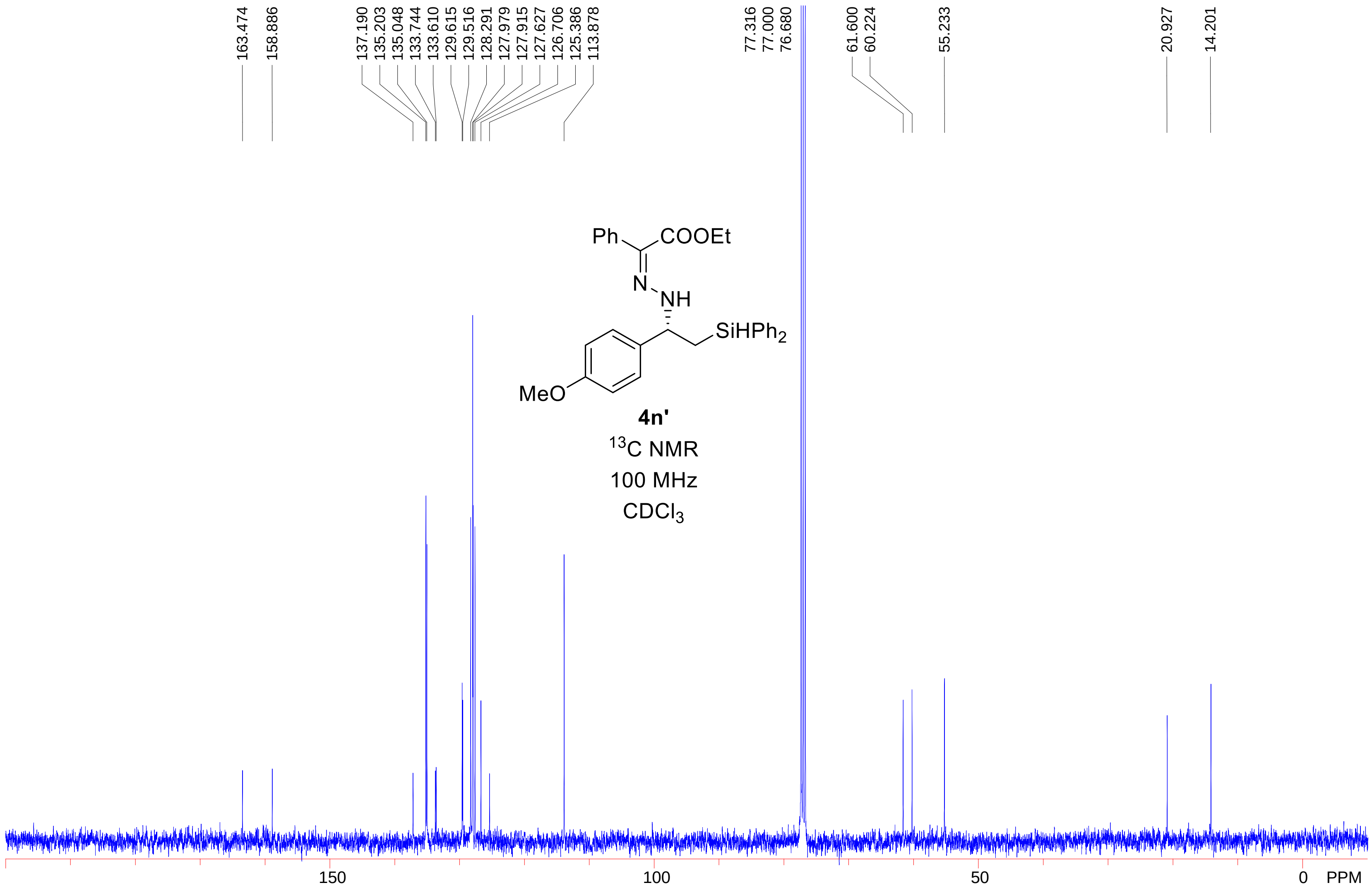

Supplementary Figure 117. ^{13}C NMR spectrum of **4n'**.

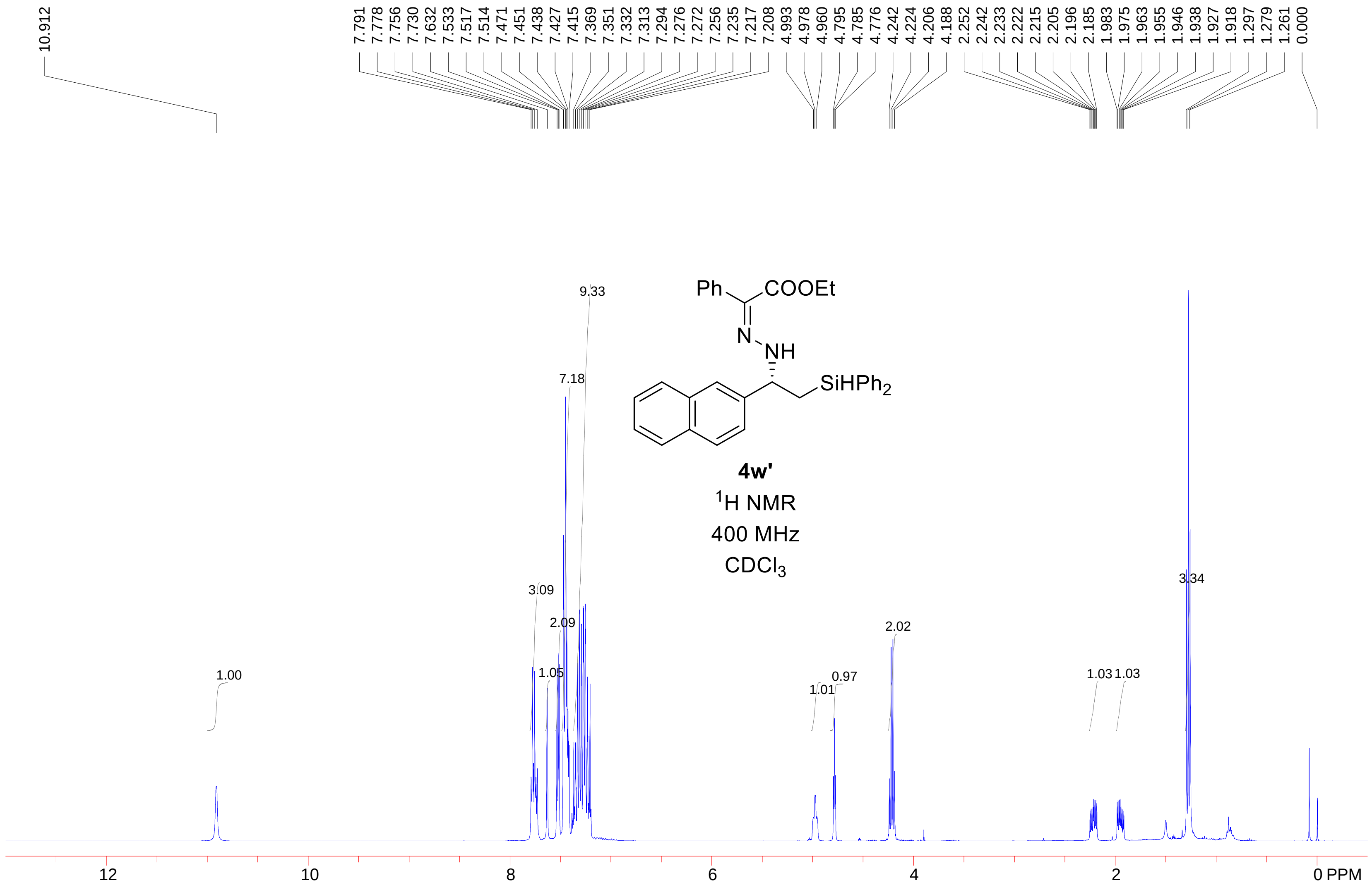

Supplementary Figure 118. ^{1}H NMR spectrum of **4w'**.

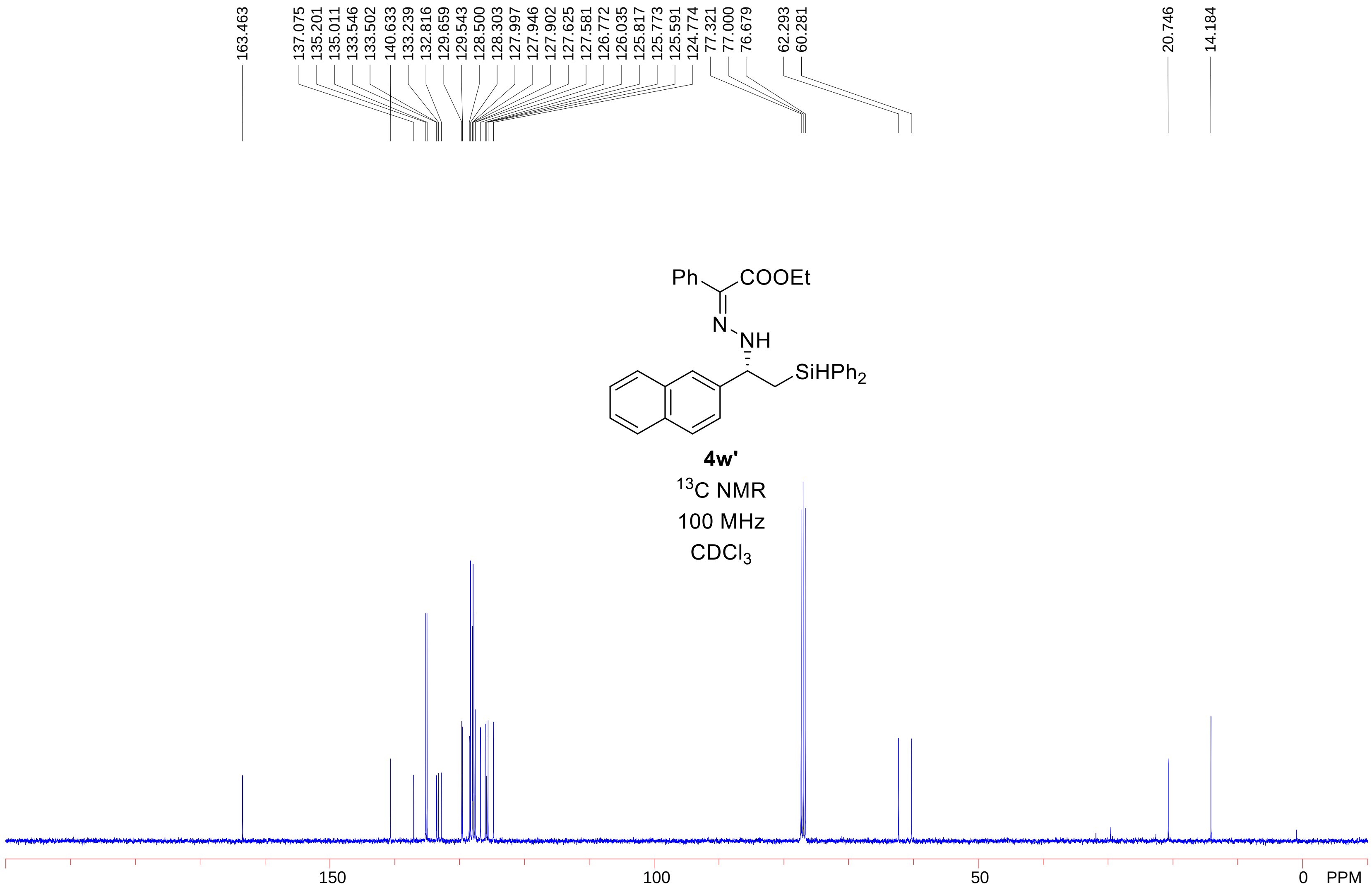

Supplementary Figure 119. ^{13}C NMR spectrum of **4w’**.

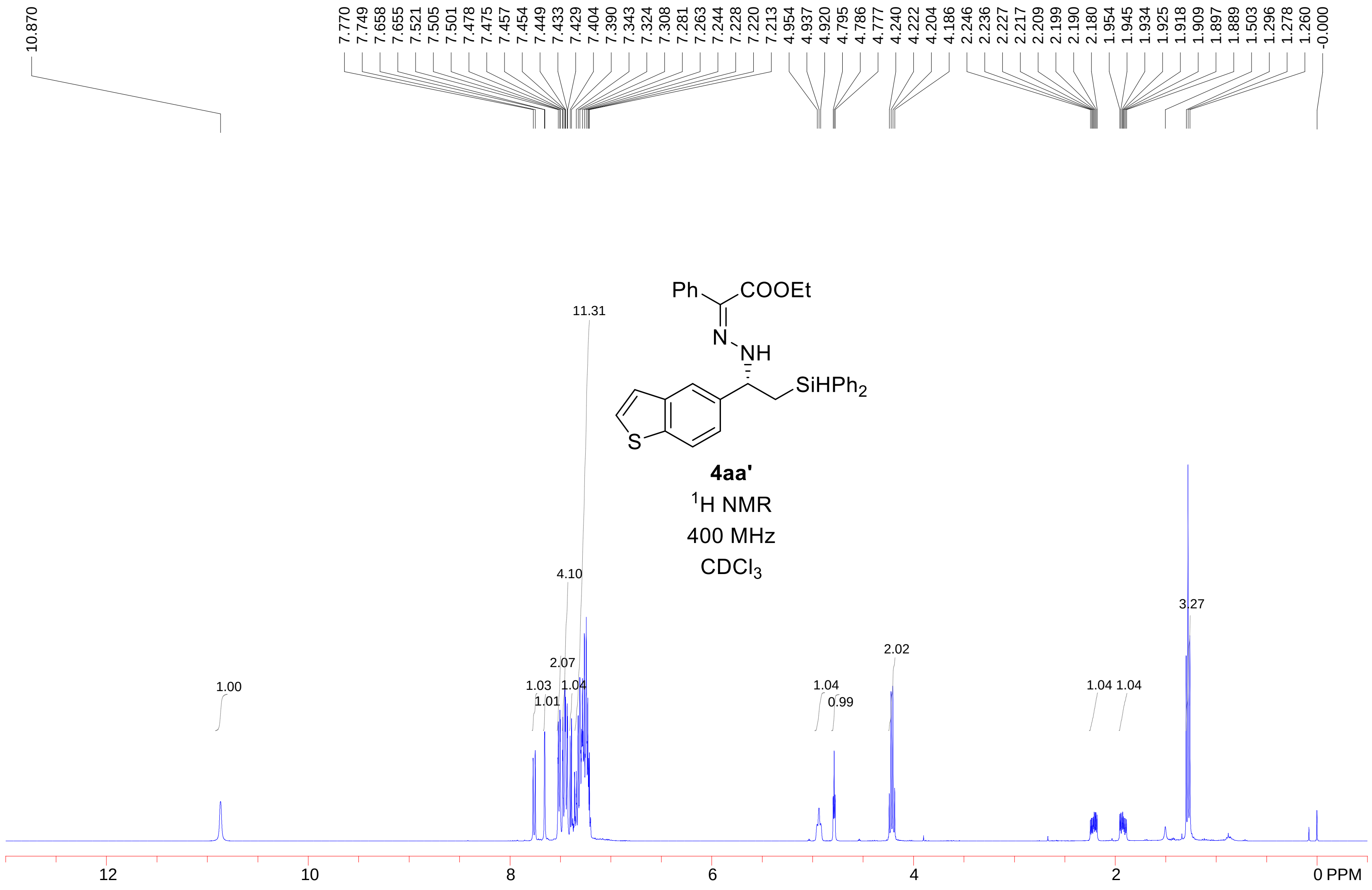

Supplementary Figure 120. ^{1}H NMR spectrum of **4aa'**.

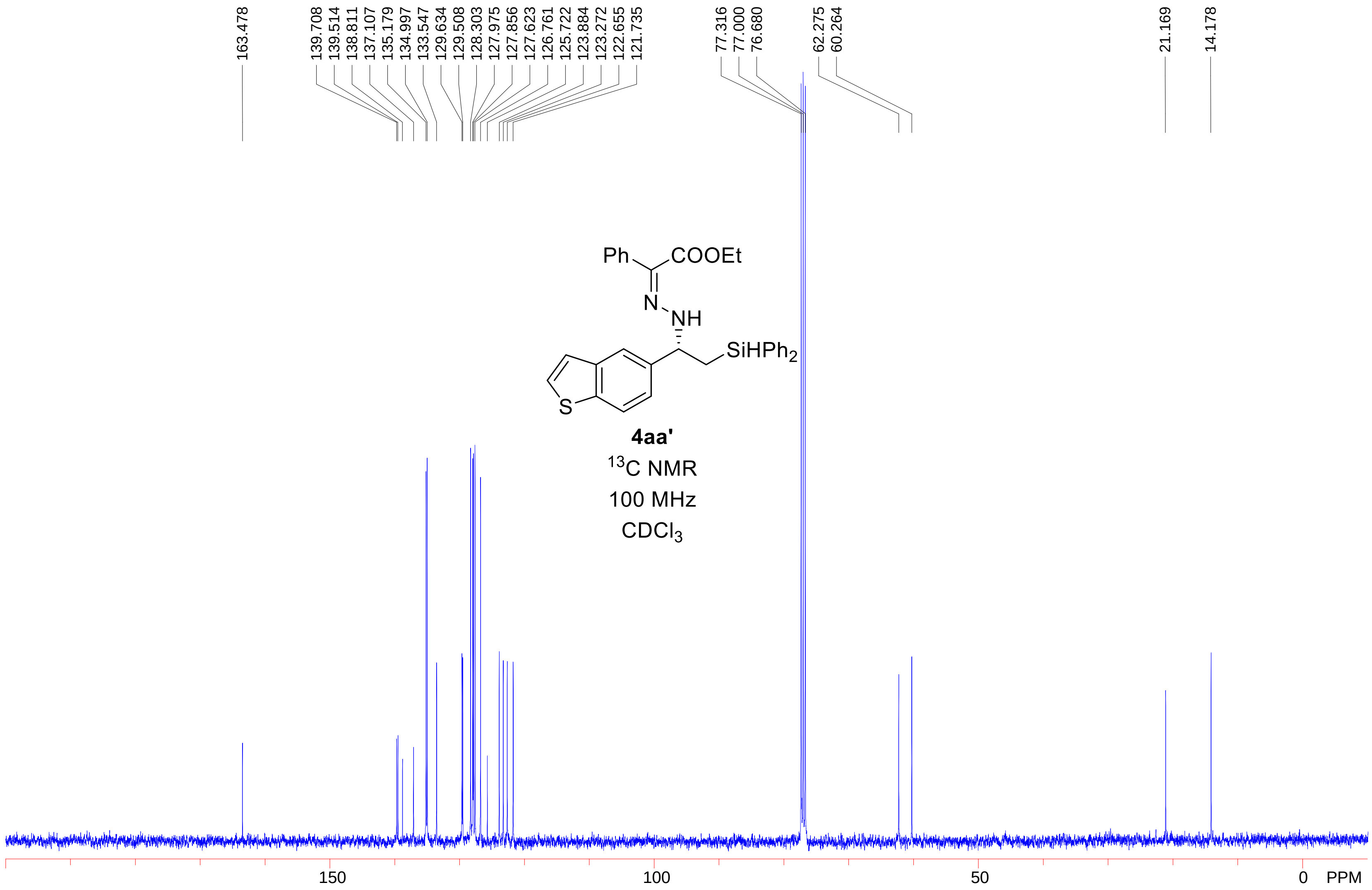

Supplementary Figure 121. ^{13}C NMR spectrum of **4aa’**.

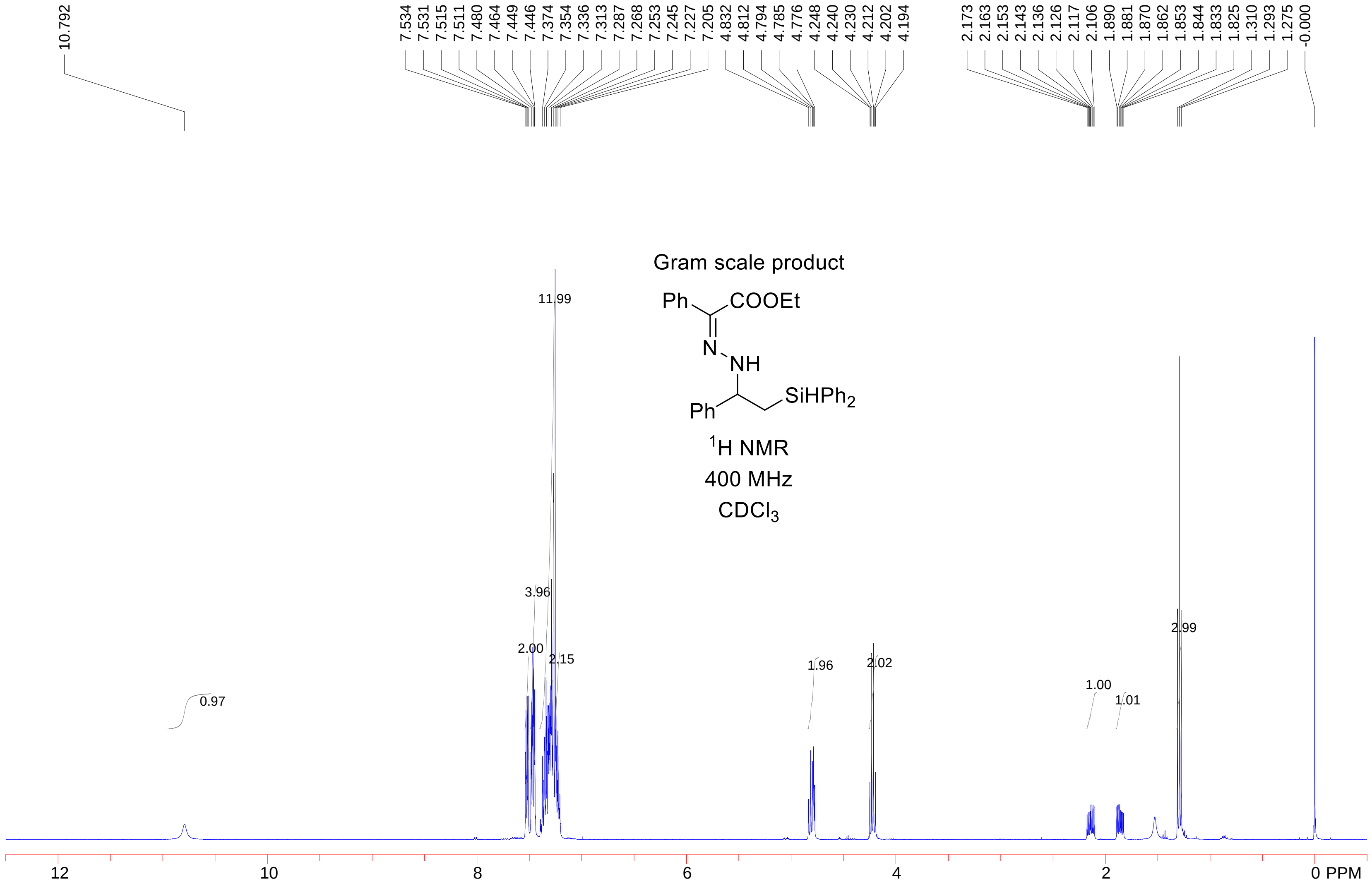

Supplementary Figure 122. ^{1}H NMR spectrum of **4a** in gram scale reaction.

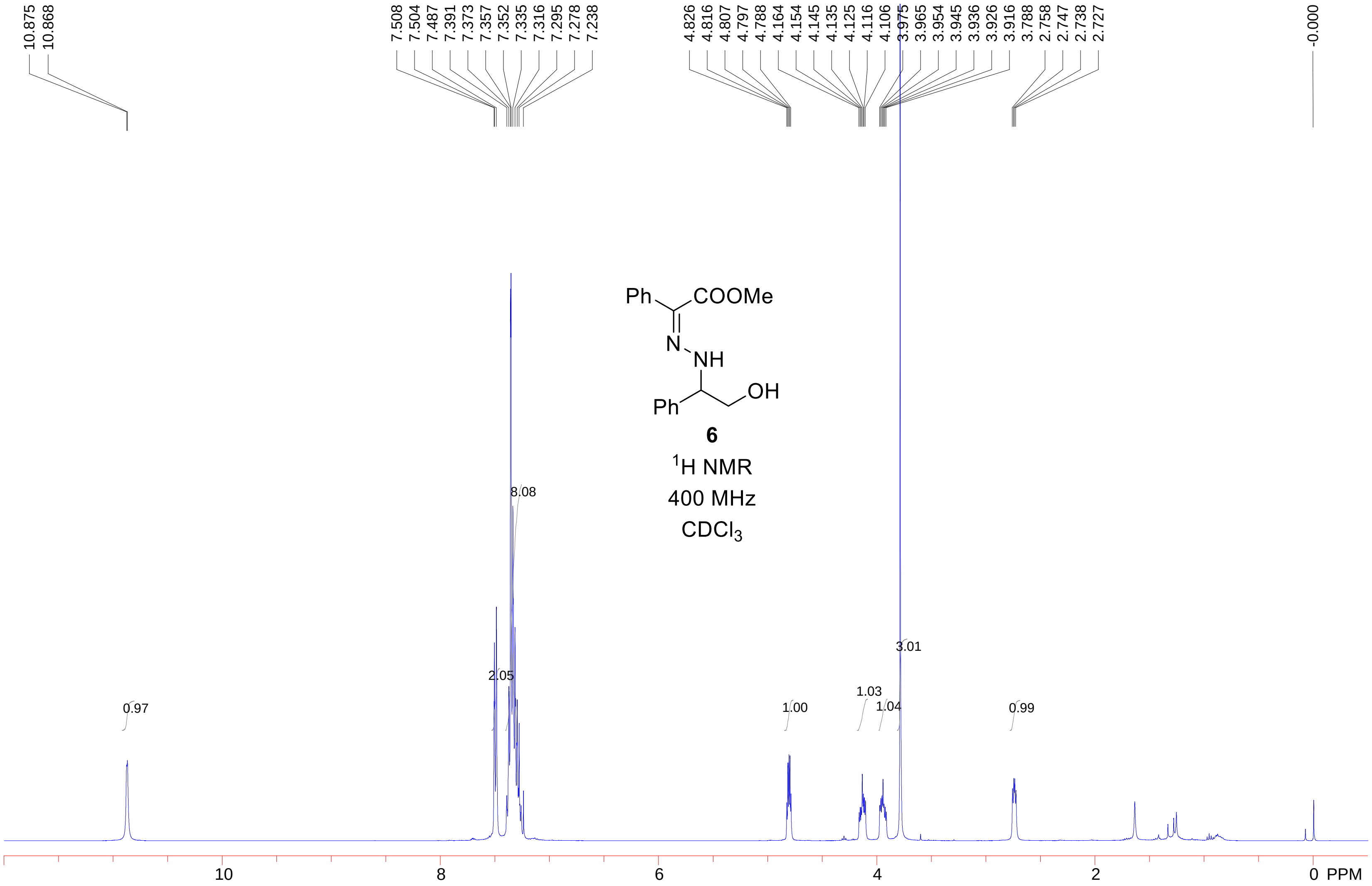

Supplementary Figure 123. ^{1}H NMR spectrum of **6**.

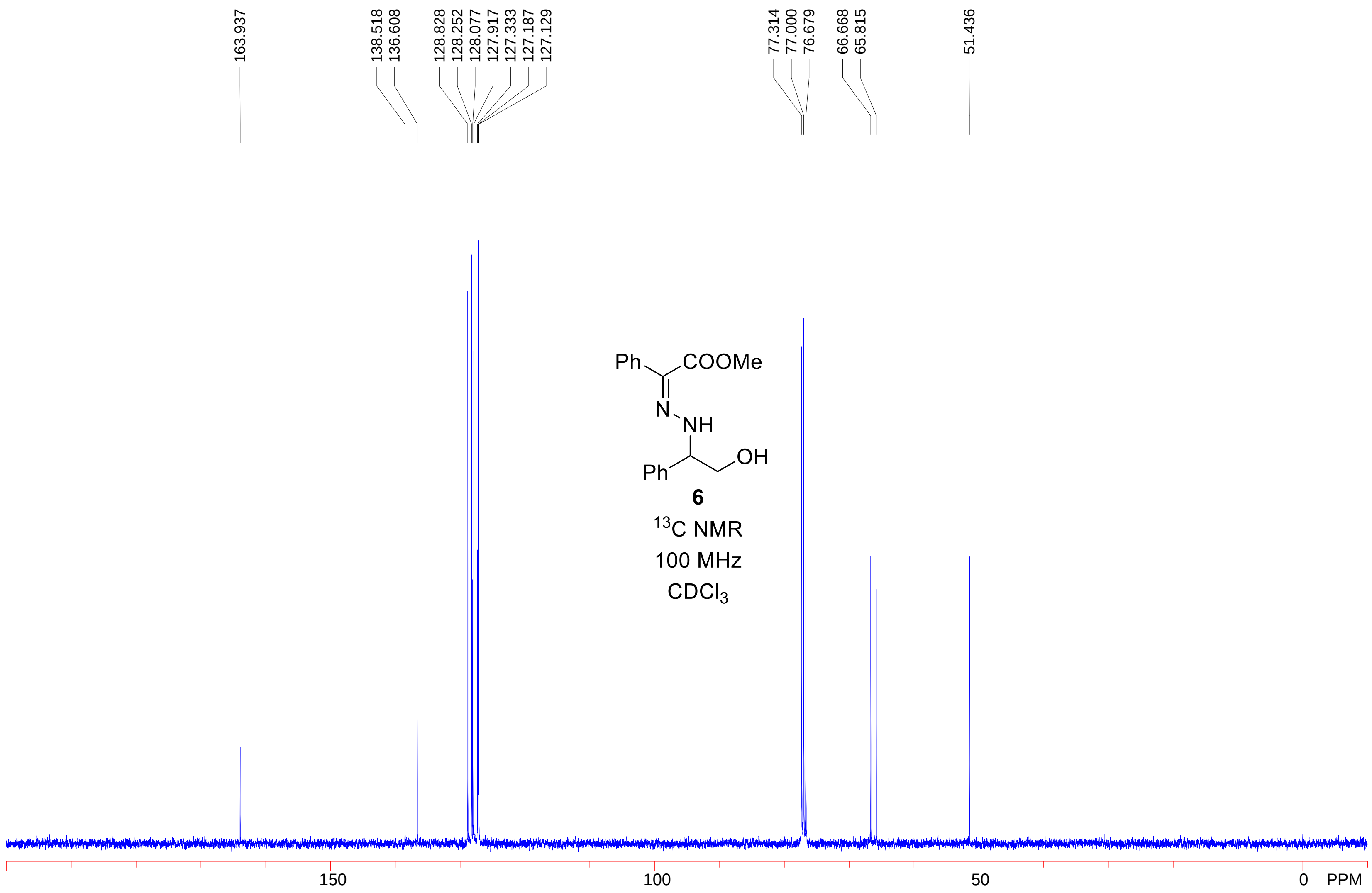

Supplementary Figure 124. ^{13}C NMR spectrum of **6**.

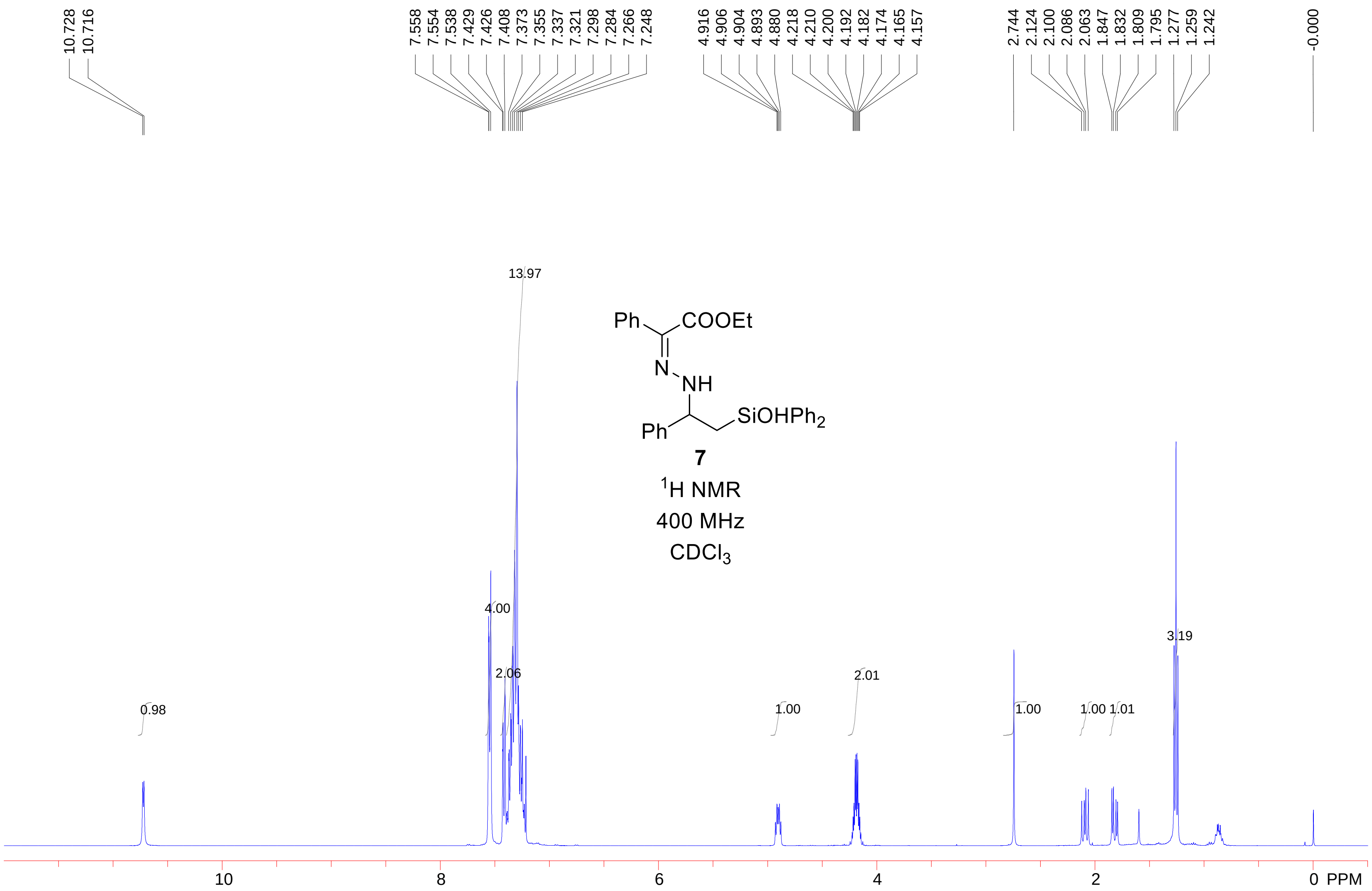

Supplementary Figure 125. ^{1}H NMR spectrum of **7**.

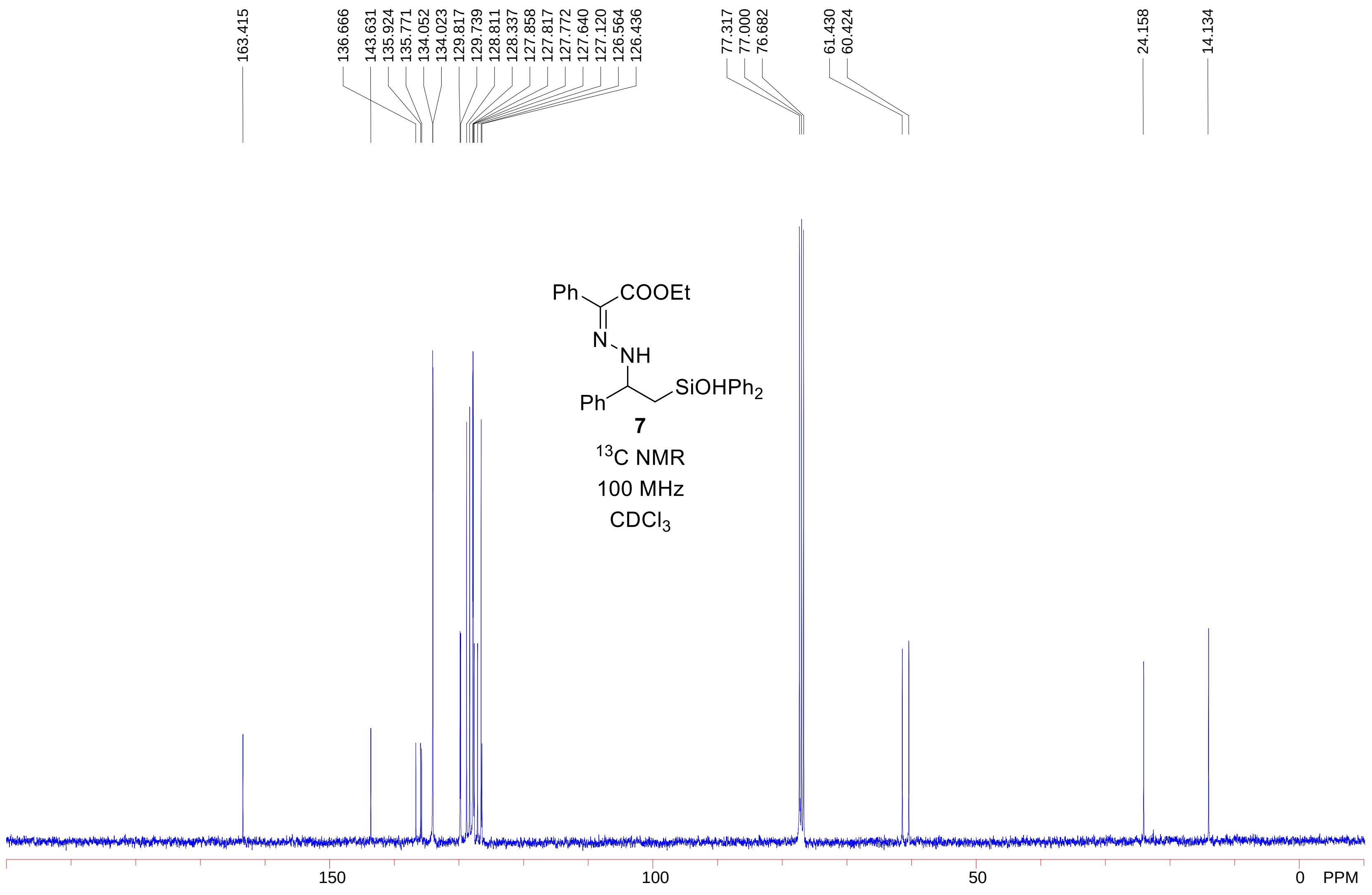

Supplementary Figure 126. ^{13}C NMR spectrum of **7**.

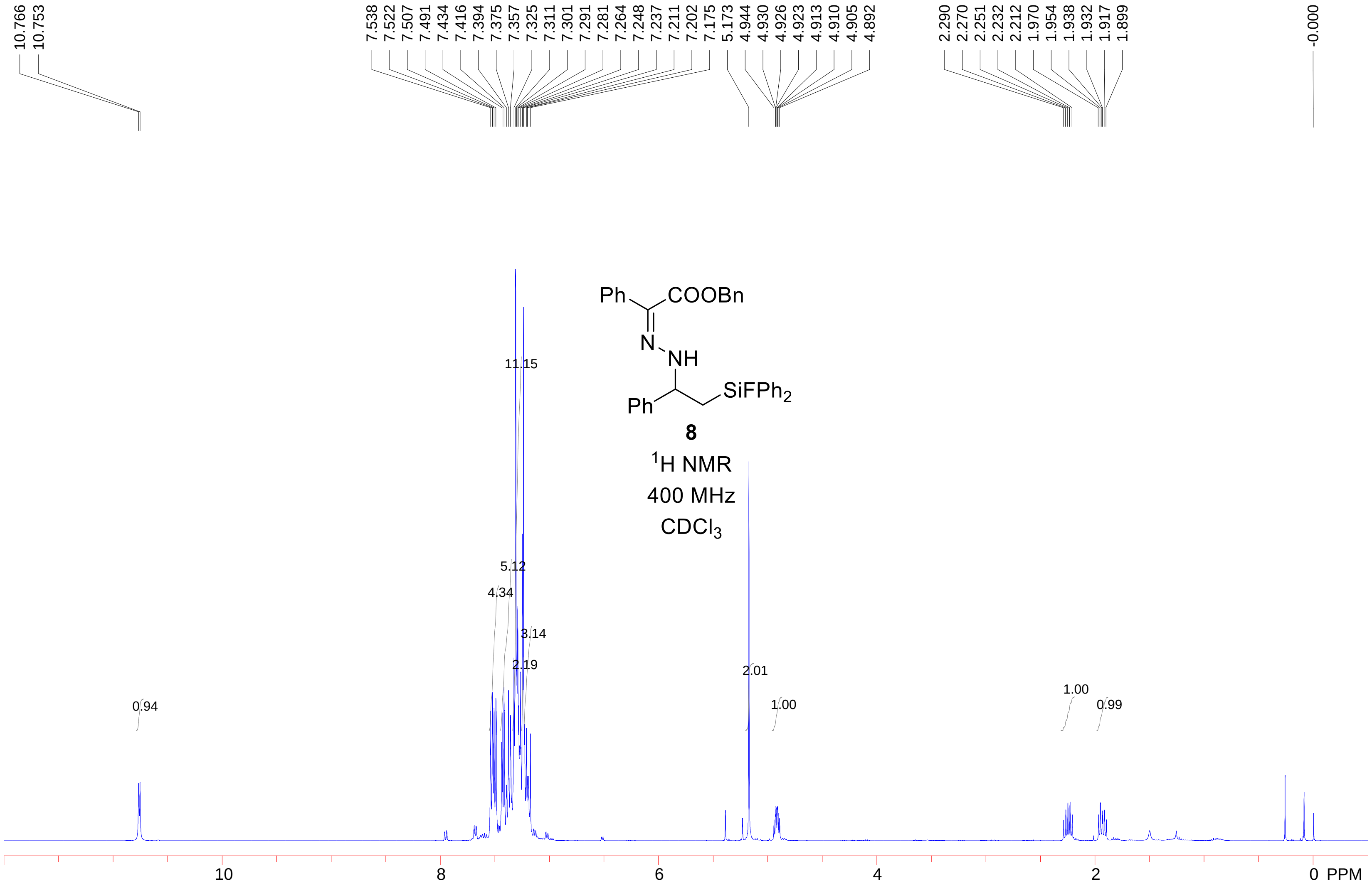

Supplementary Figure 127. ^{1}H NMR spectrum of **8**.

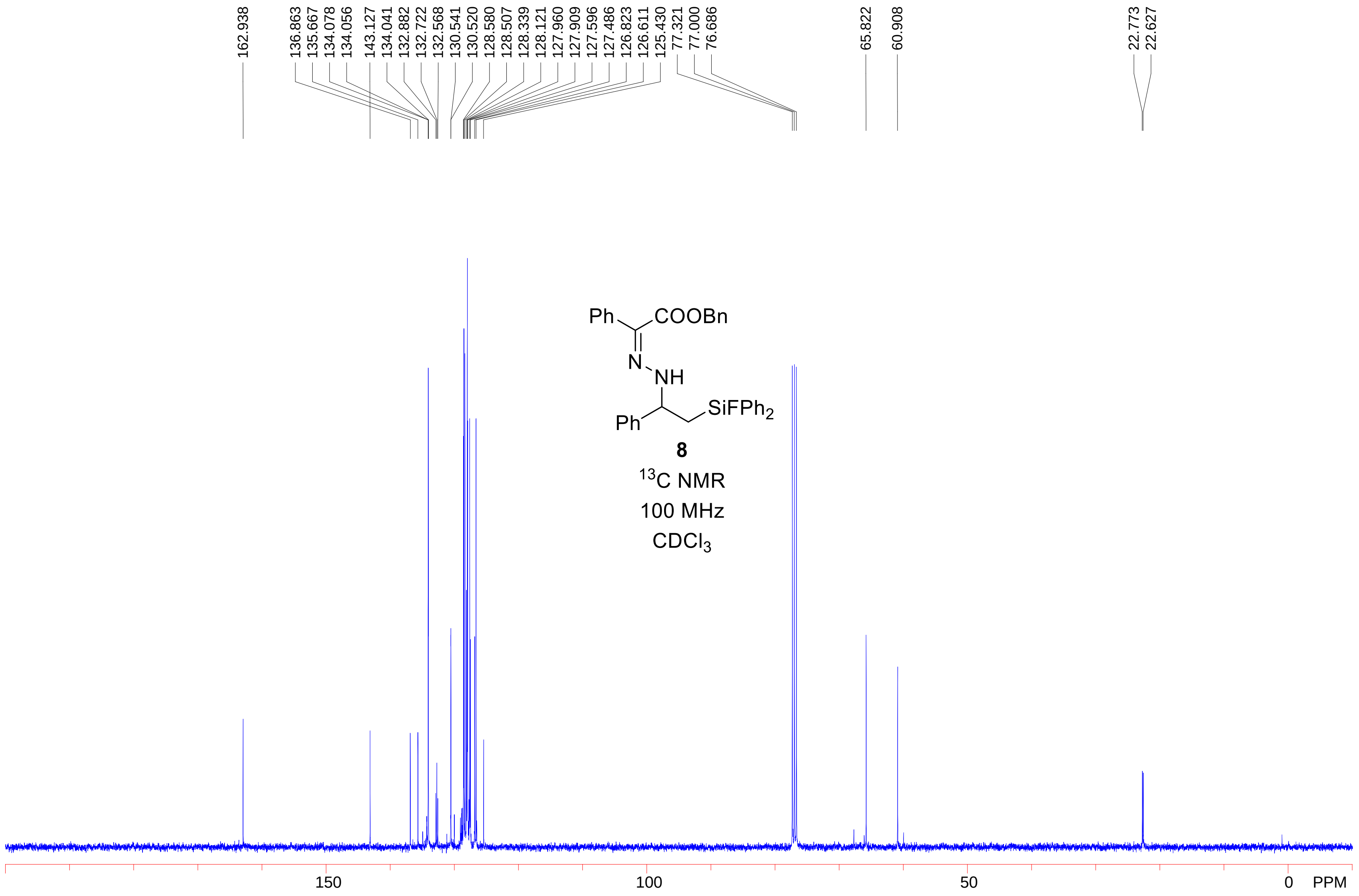

Supplementary Figure 128. ^{13}C NMR spectrum of **8**.

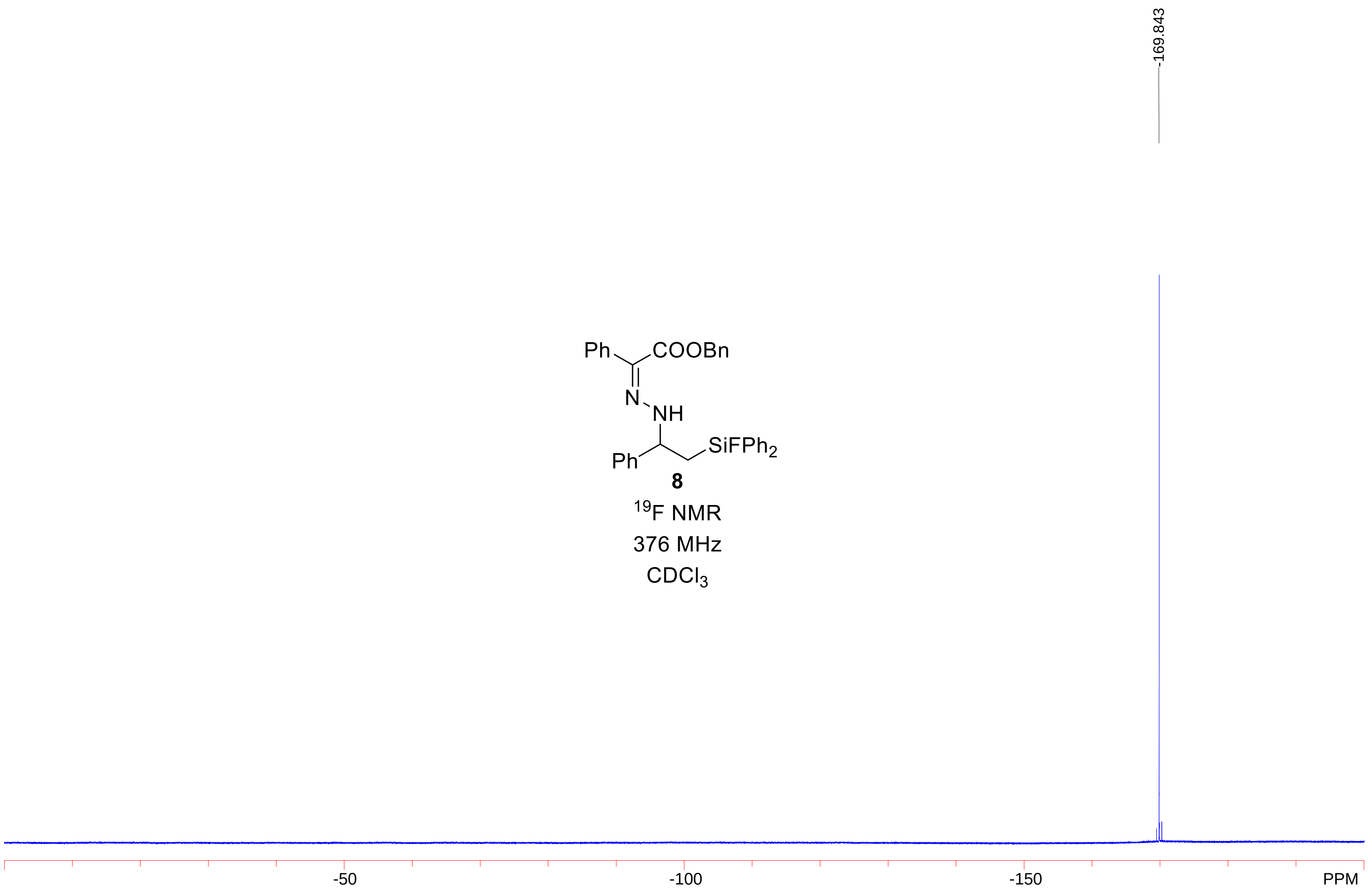

Supplementary Figure 129. ^{19}F NMR spectrum of **8**.

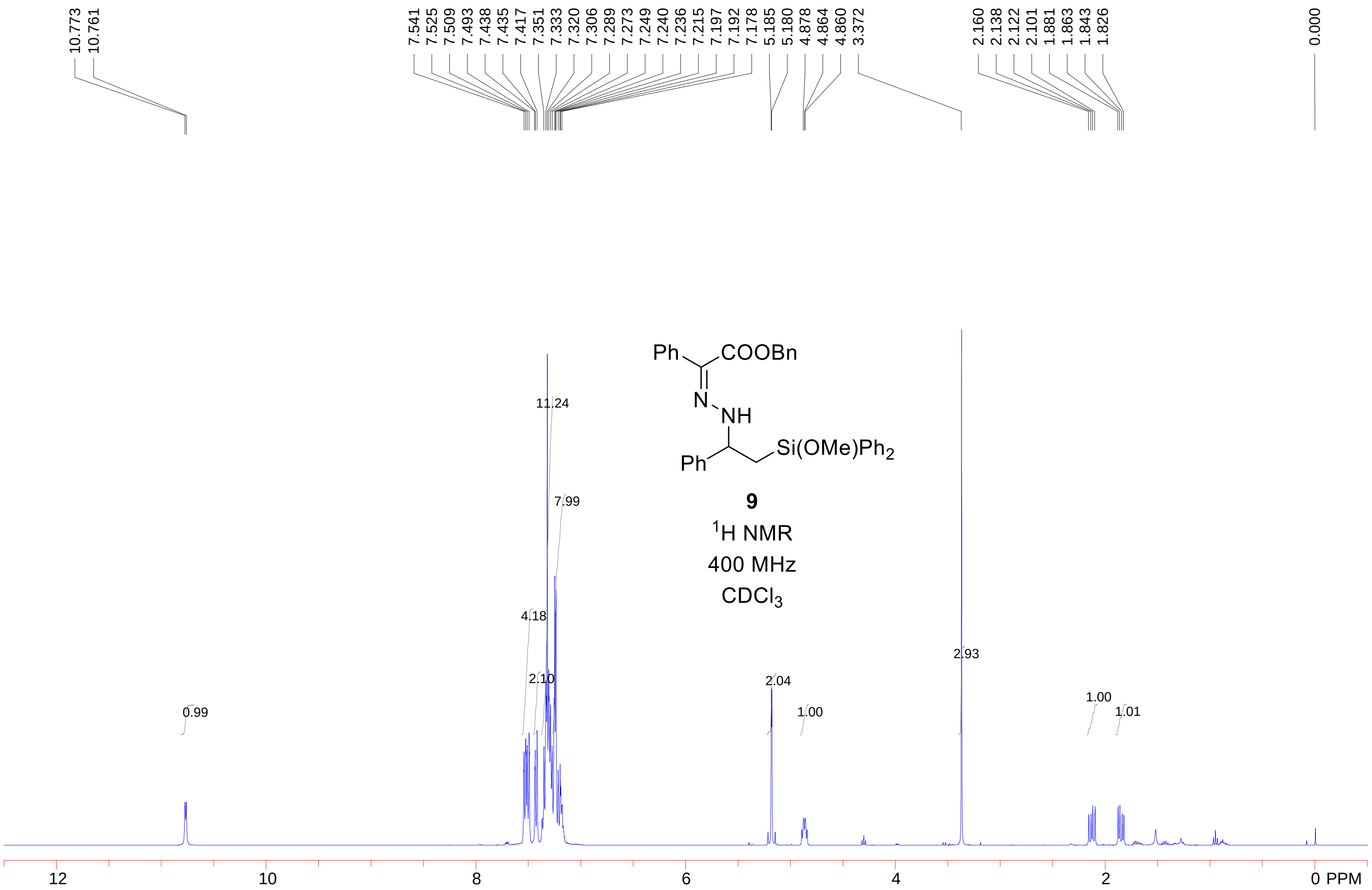

Supplementary Figure 130. ^{1}H NMR spectrum of **9**.

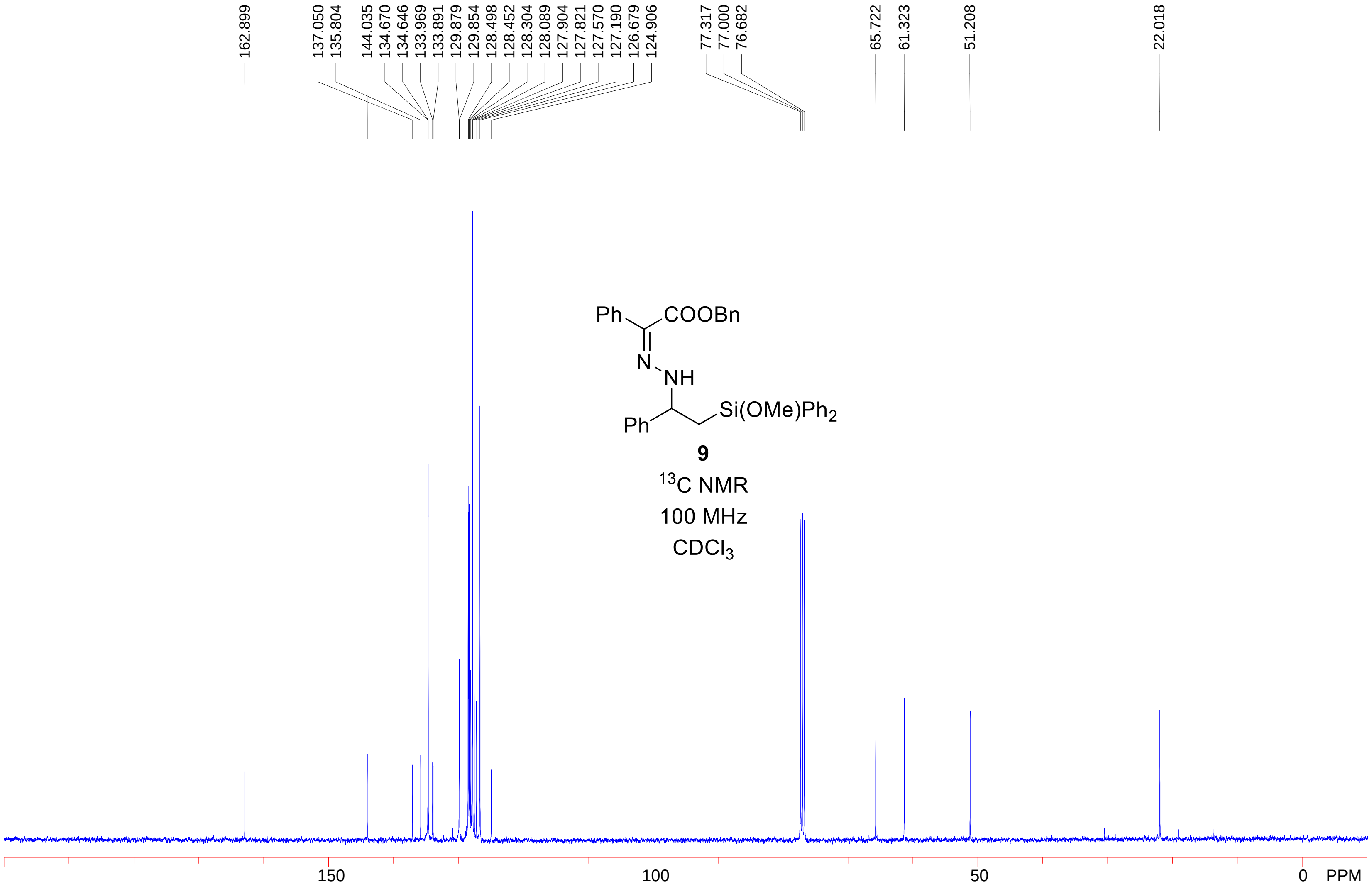

Supplementary Figure 131. ^{13}C NMR spectrum of **9**.

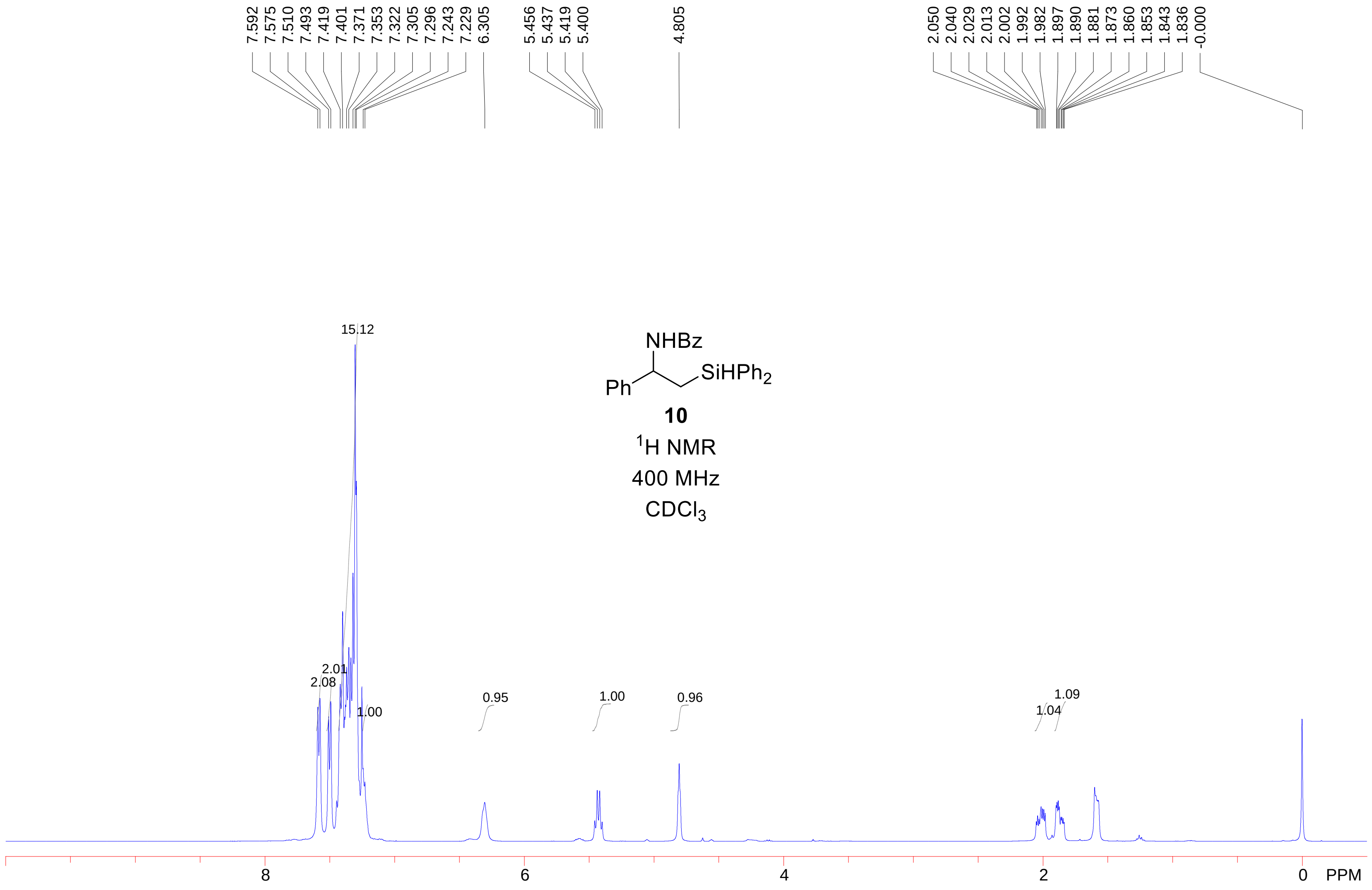

Supplementary Figure 132. ^{1}H NMR spectrum of **10.**

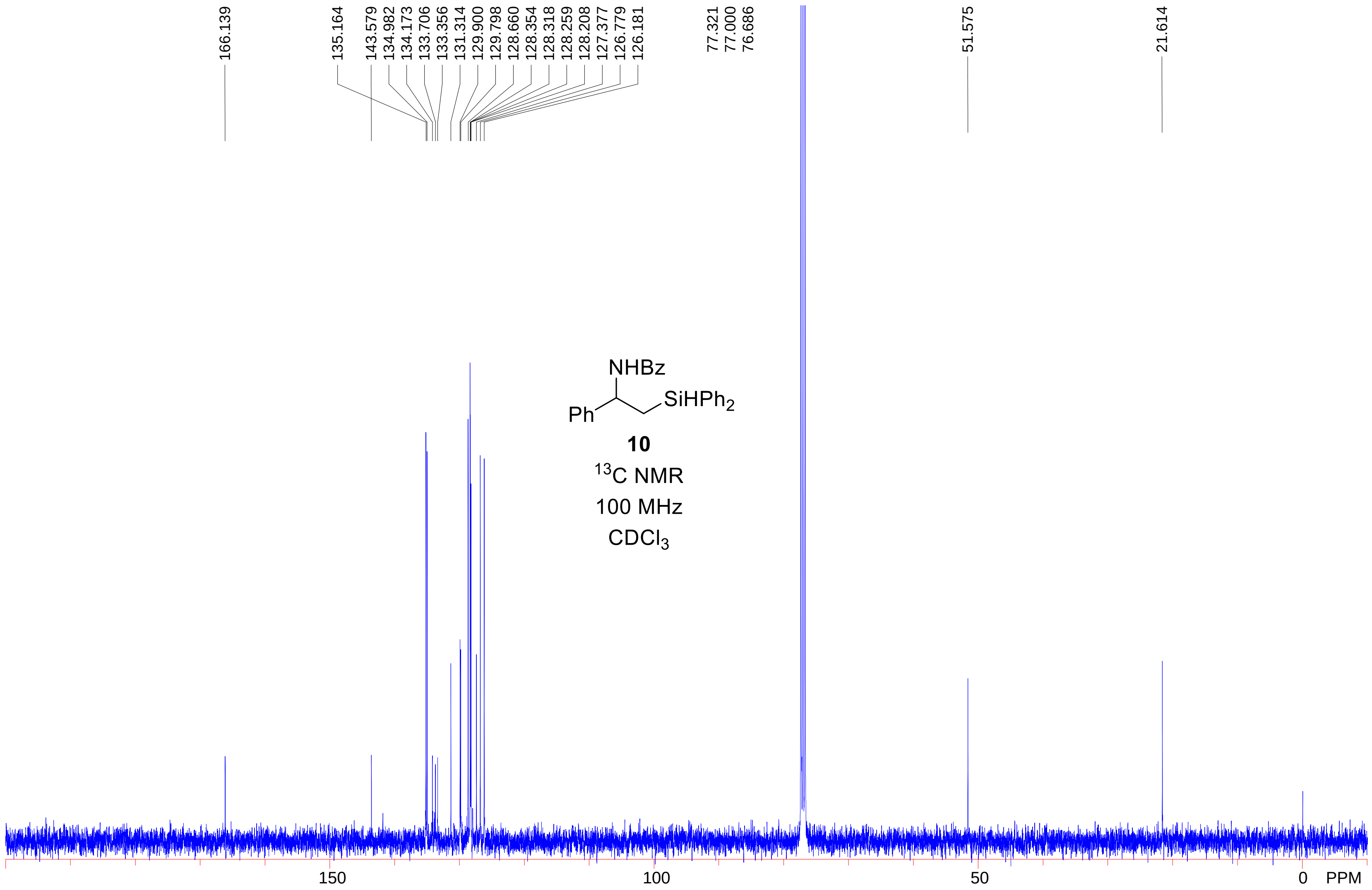

Supplementary Figure 133. ^{13}C NMR spectrum of **10**.

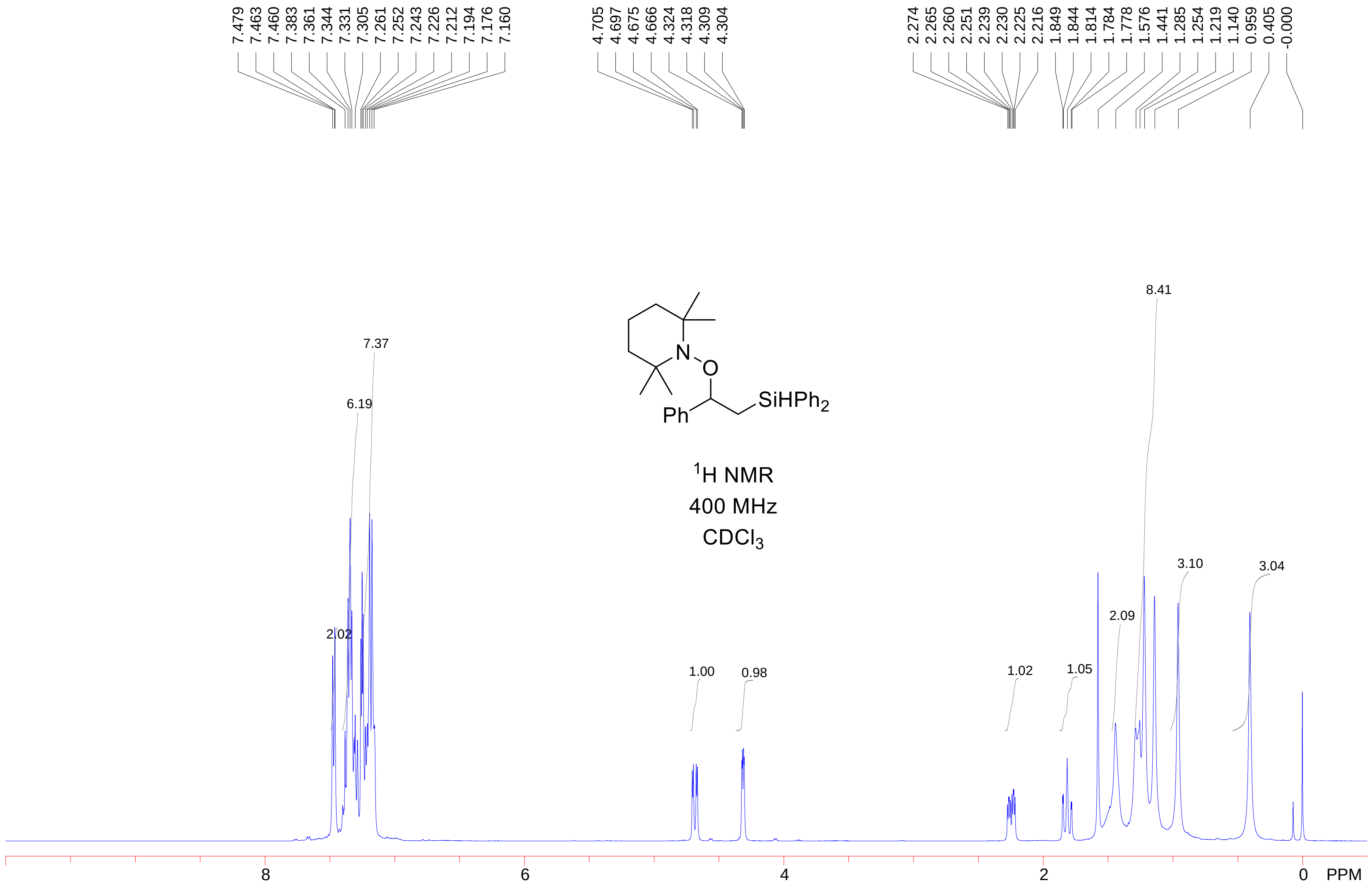

Supplementary Figure 134. ^{1}H NMR spectrum of product of benzyl radical trapped by TEMPO.

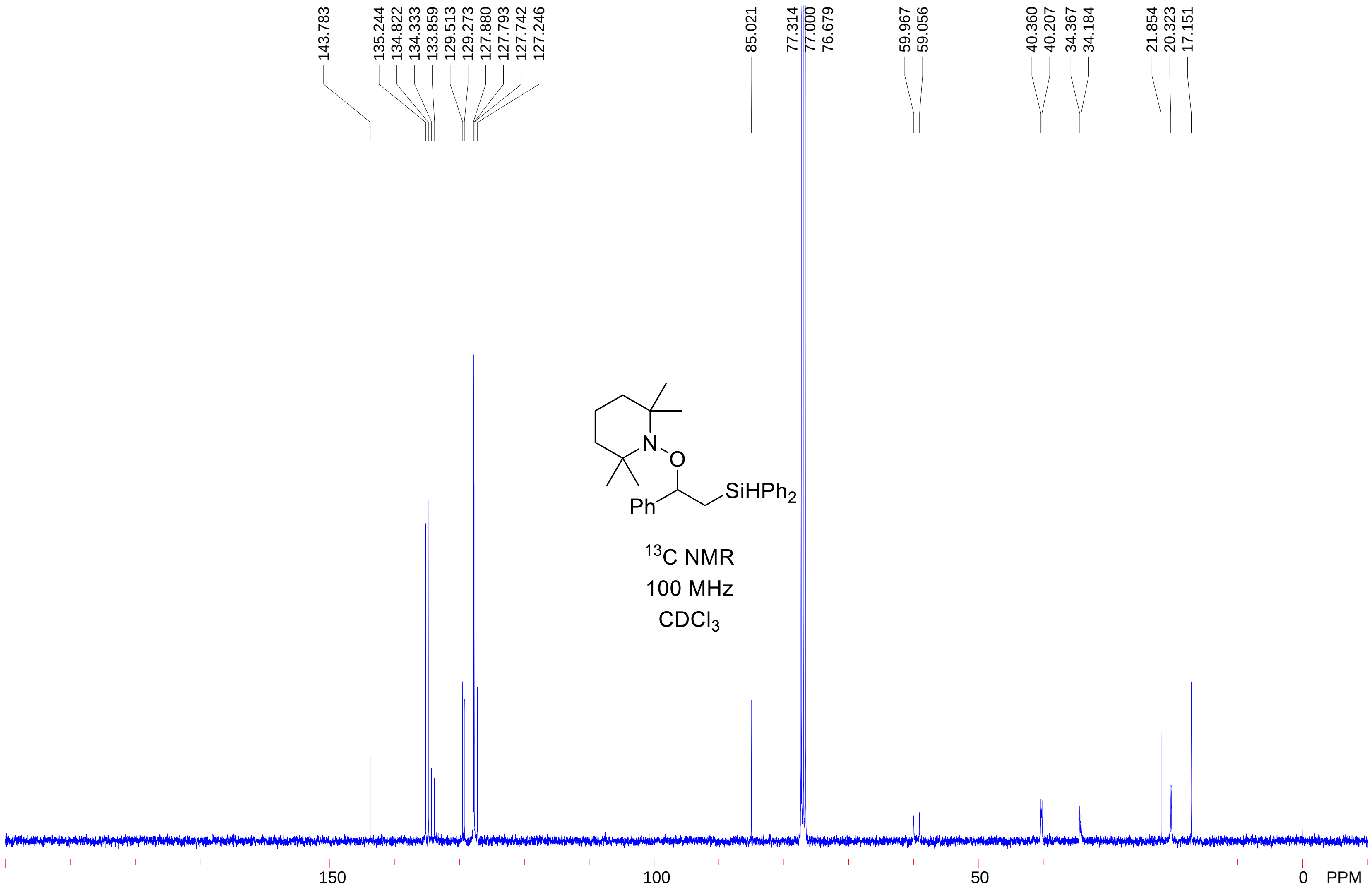

Supplementary Figure 135. ^{13}C NMR spectrum of product of benzyl radical trapped by TEMPO.

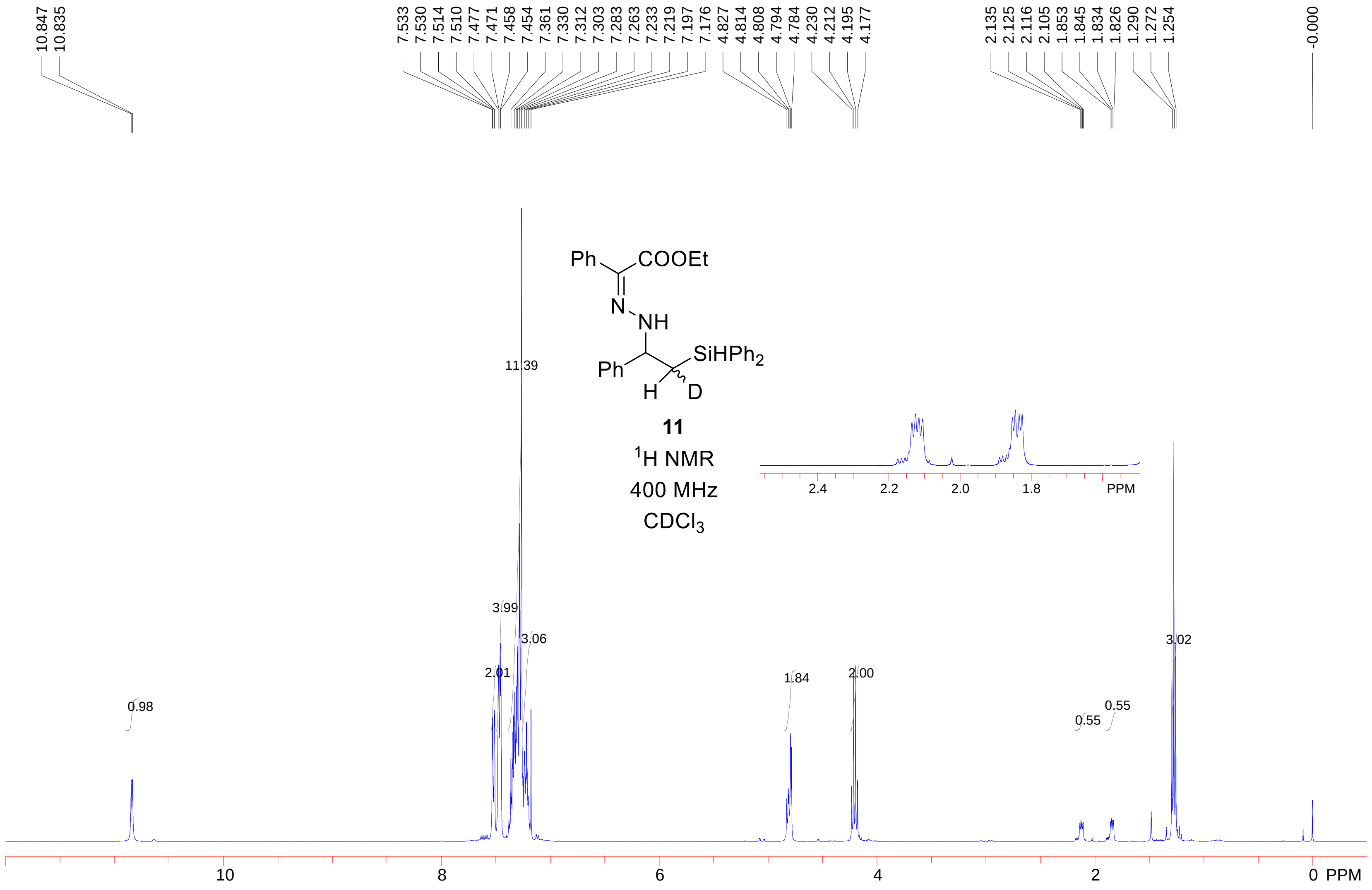

Supplementary Figure 136. ^{1}H NMR spectrum of **11.**

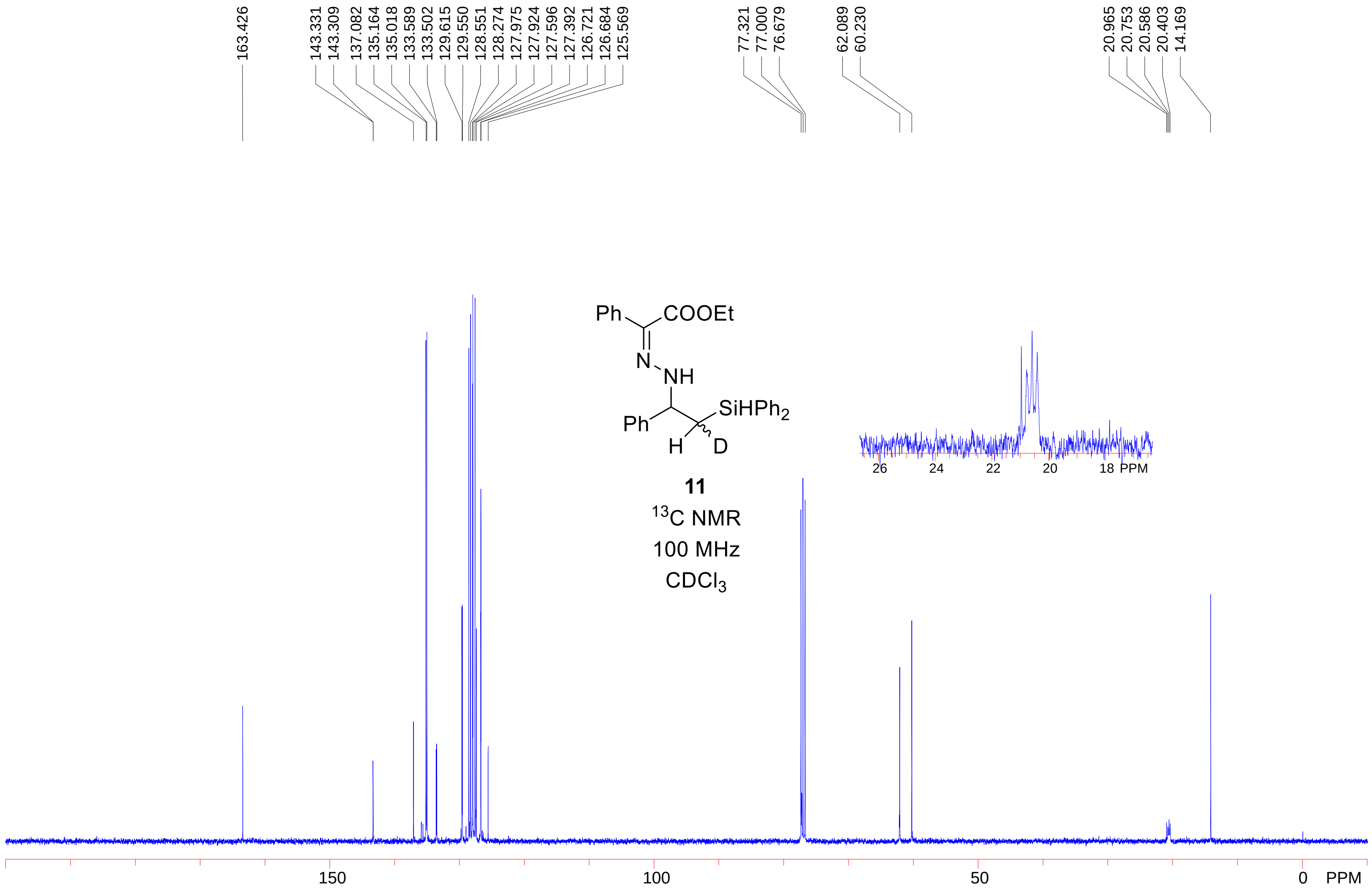

Supplementary Figure 137. ^{13}C NMR spectrum of **11.**

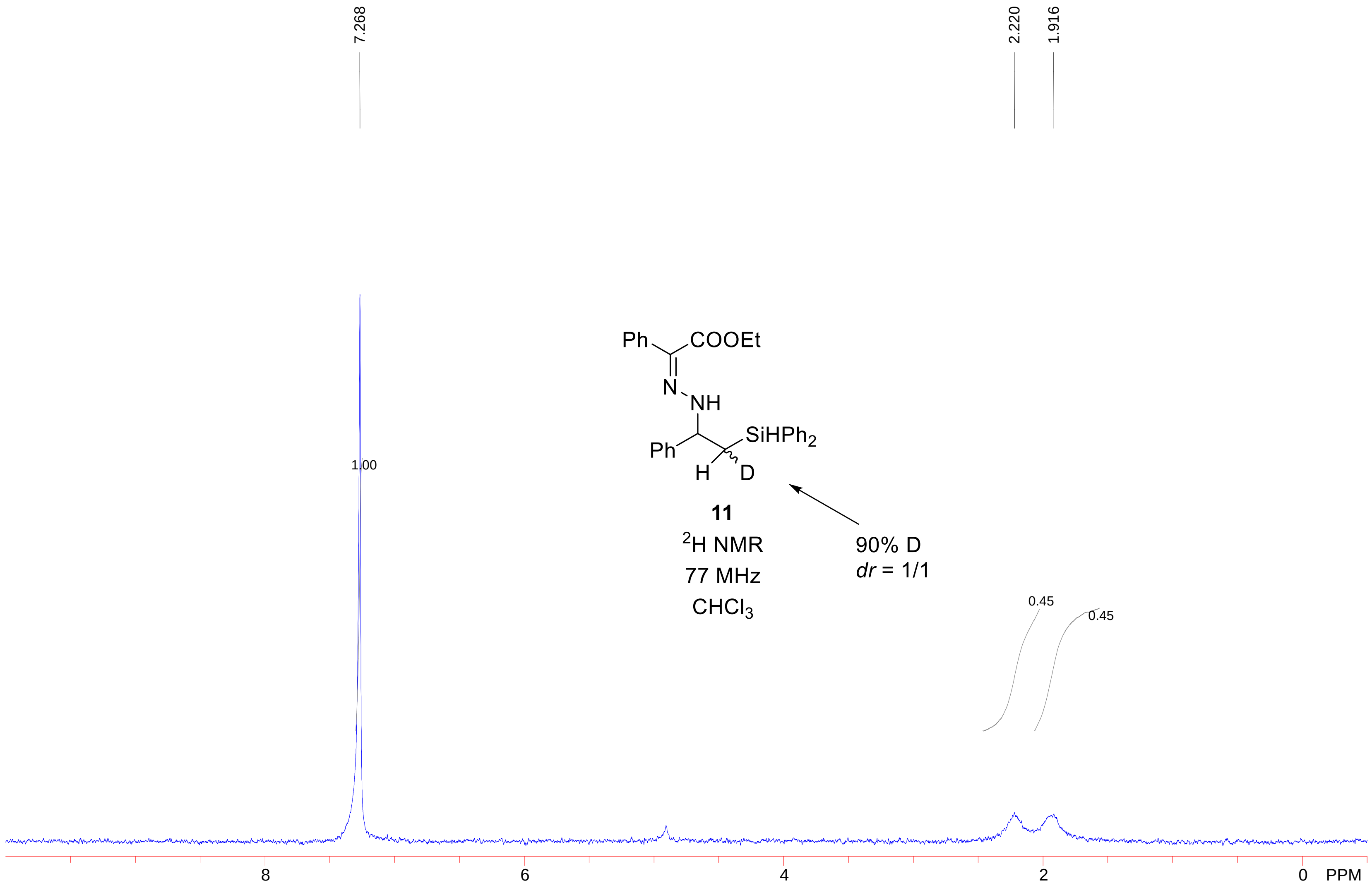

Supplementary Figure 138. ^{2}H NMR spectrum of **11.**

X. HPLC Spectra

分析日期 : 2021/6/30 19:59:42
处理日期 : 2021/6/30 20:18:39
描述 : OD-H, n-hexane/iPrOH=95/5, 1.0 ml/min, 220 nm

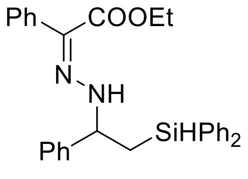

rac-**4a'**

色谱图

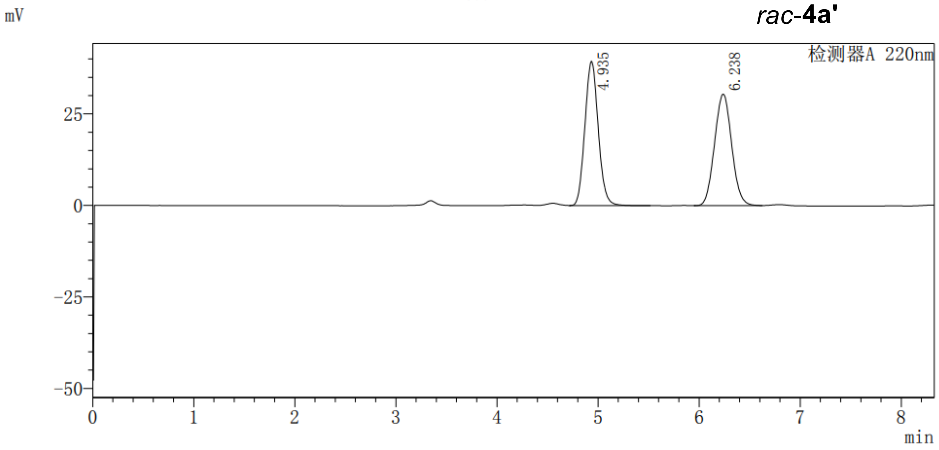

峰表

检测器A 220nm

峰号	保留时间	面积	高度	标记	面积%
1	4.935	362681	39412		50.202
2	6.238	359756	30480		49.798
总计		722437	69892		100.000

分析日期 : 2021/6/19 21:22:22
处理日期 : 2021/6/19 21:38:44
描述 : OD-H, n-hexane/iPrOH=95/5, 1.0 ml/min, 220 nm

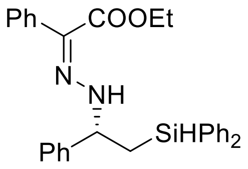

S-**4a'**

色谱图

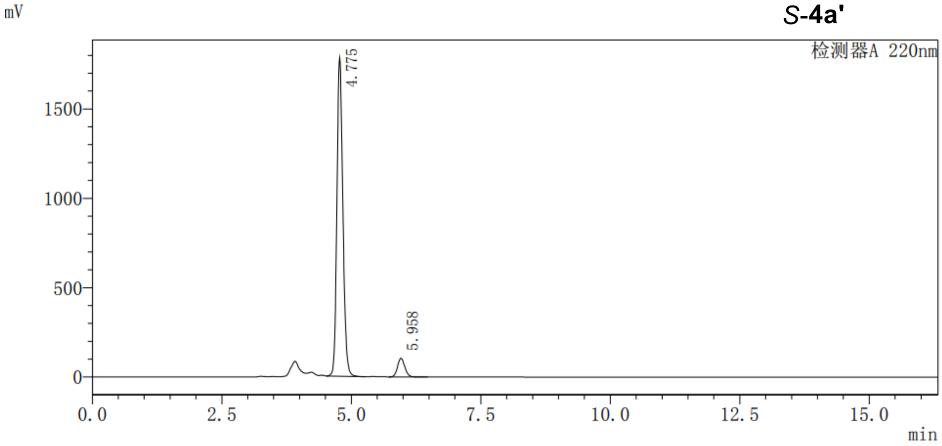

峰表

检测器A 220nm

峰号	保留时间	面积	高度	标记	面积%
1	4.775	14182600	1781496		93.297
2	5.958	1018888	104366		6.703
总计		15201487	1885862		100.000

Supplementary Figure 139. HPLC spectra of *S*-**4a'**, related Table 3.

分析日期　　　: 2021/6/23 20:09:04
处理日期　　　: 2021/6/23 20:17:47
描述　　　　　: OD-H, n-hexane/iPrOH=95/5, 1.0 ml/min, 220 nm

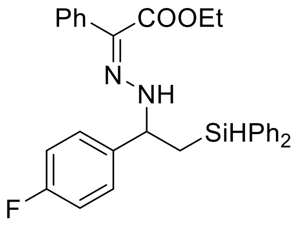

rac-**4i'**

色谱图

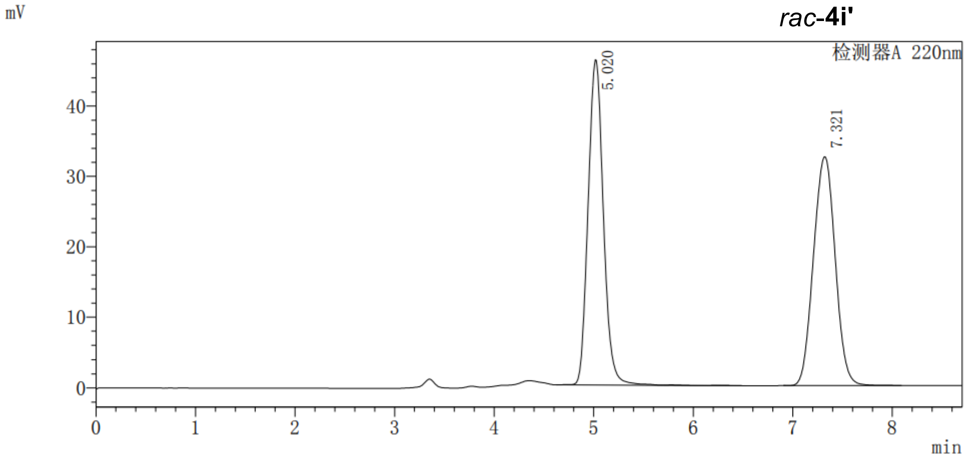

峰表

检测器A 220nm

峰号	保留时间	面积	高度	标记	面积%
1	5.020	492141	46146	S	50.018
2	7.321	491777	32451		49.982
总计		983918	78597		100.000

处理日期　　　: 2021/6/23 20:27:40
描述　　　　　: OD-H, n-hexane/iPrOH=95/5, 1.0 ml/min, 220 nm

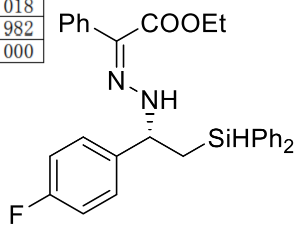

色谱图

S-**4i'**

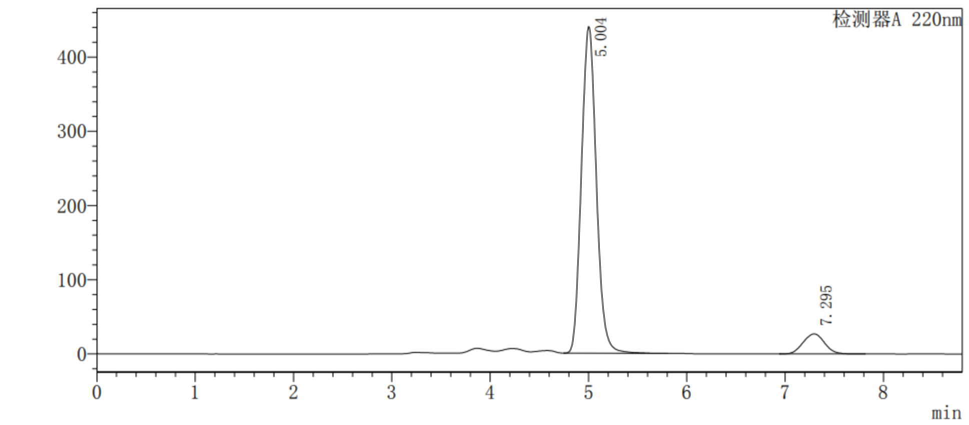

峰表

检测器A 220nm

峰号	保留时间	面积	高度	标记	面积%
1	5.004	4649672	439942		91.914
2	7.295	409039	26998		8.086
总计		5058711	466940		100.000

Supplementary Figure 140. HPLC spectra of *S*-**4i'**, related Table 3.

分析日期 : 2021/6/10 10:33:13
处理日期 : 2021/6/10 10:49:49
描述 : OD-H, n-hexane/iPrOH=95/5, 1 ml/min, 220 nm

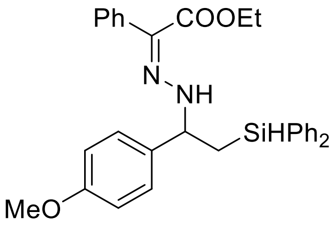

rac-**4n'**

色谱图

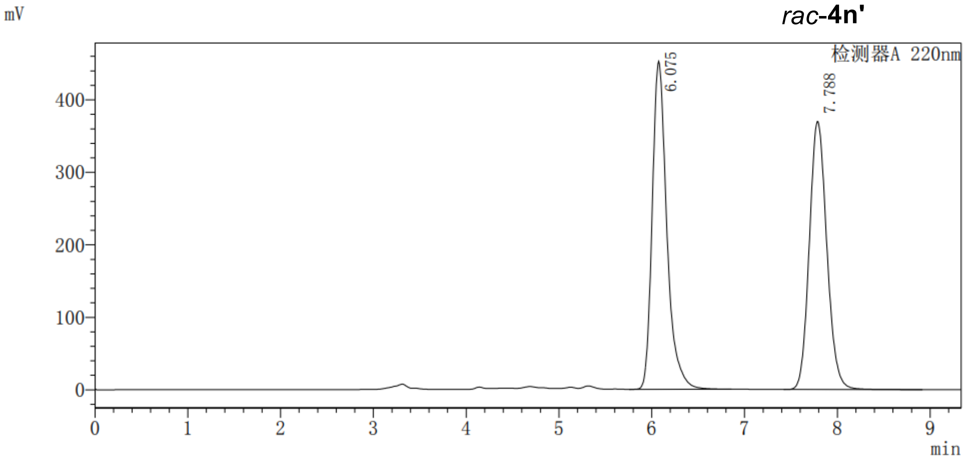

峰表

检测器A 220nm

峰号	保留时间	面积	高度	标记	面积%
1	6.075	4959055	452397		50.561
2	7.788	4848929	369726		49.439
总计		9807984	822122		100.000

分析日期 : 2021/6/19 21:41:56
处理日期 : 2021/6/19 21:50:57
描述 : OD-H, n-hexane/iPrOH=95/5, 1.0 ml/min, 220 nm

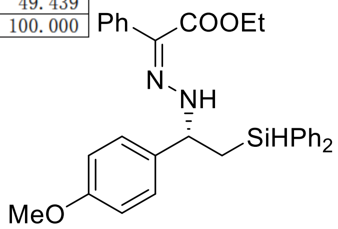

S-**4n'**

色谱图

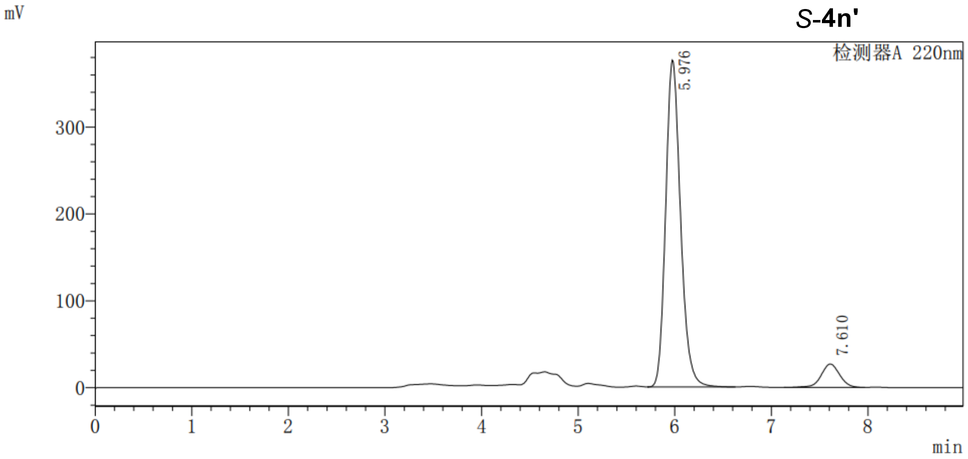

峰表

检测器A 220nm

峰号	保留时间	面积	高度	标记	面积%
1	5.976	4029944	376095		91.833
2	7.610	358419	26941		8.167
总计		4388362	403036		100.000

Supplementary Figure 141. HPLC spectra of *S*-**4n’**, related Table 3.

分析日期　　　: 2021/6/23 20:30:27
处理日期　　　: 2021/6/23 20:39:53
描述　　　　　: OD-H, n-hexane/iPrOH=95/5, 1.0 ml/min, 220 nm

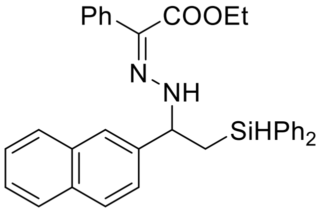

色谱图

rac-**4w'**

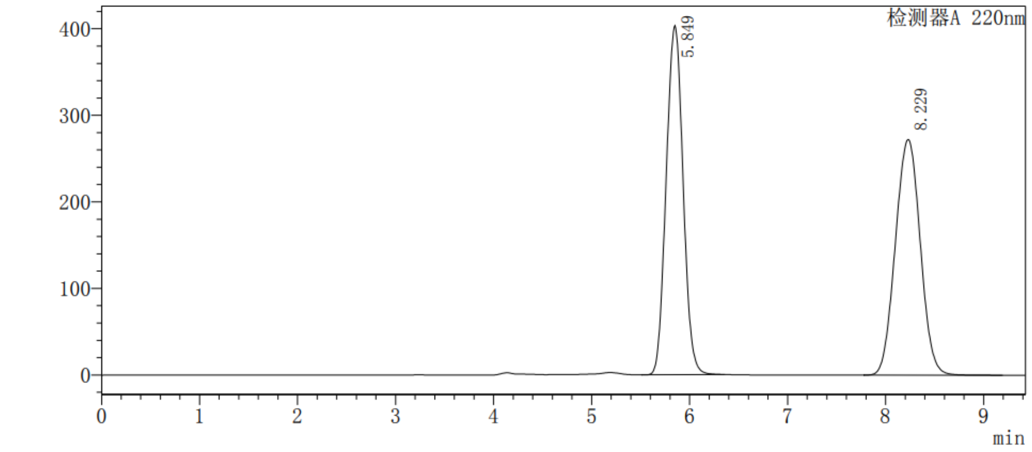

峰表

检测器A 220nm

峰号	保留时间	面积	高度	标记	面积%
1	5.849	4834446	403329	M	50.164
2	8.229	4802752	272174		49.836
总计		9637197	675504		100.000

分析日期　　　: 2021/6/23 20:40:25
处理日期　　　: 2021/6/23 20:50:39
描述　　　　　: OD-H, n-hexane/iPrOH=95/5, 1.0 ml/min, 220 nm

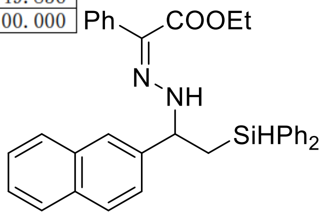

色谱图

S-**4w'**

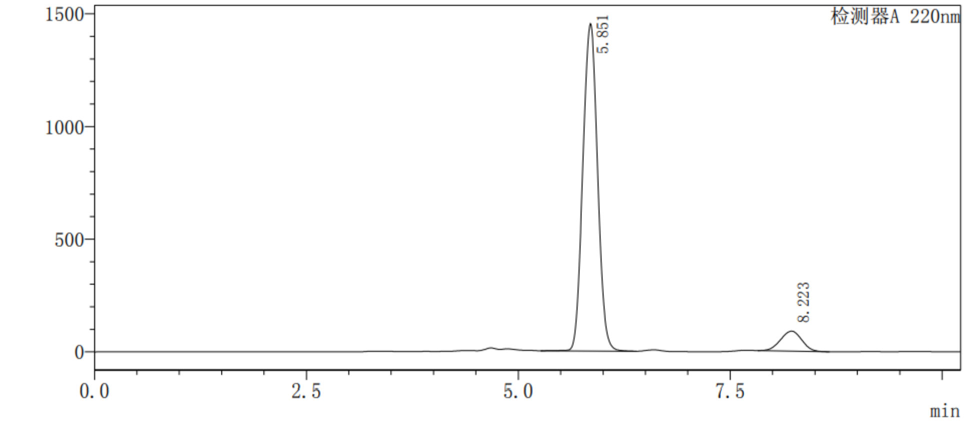

峰表

检测器A 220nm

峰号	保留时间	面积	高度	标记	面积%
1	5.851	17544502	1452425		91.920
2	8.223	1542200	88470		8.080
总计		19086702	1540895		100.000

Supplementary Figure 142. HPLC spectra of *S*-**4w'**, related Table 3.

分析日期　　: 2021/6/23 20:51:28
处理日期　　: 2021/6/23 21:18:33
描述　　　　: OD-H, n-hexane/iPrOH=95/5, 1.0 ml/min, 220 nm

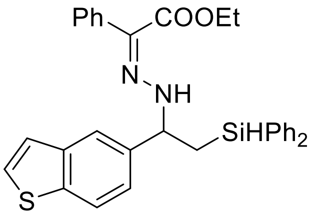

rac-**4aa'**

色谱图

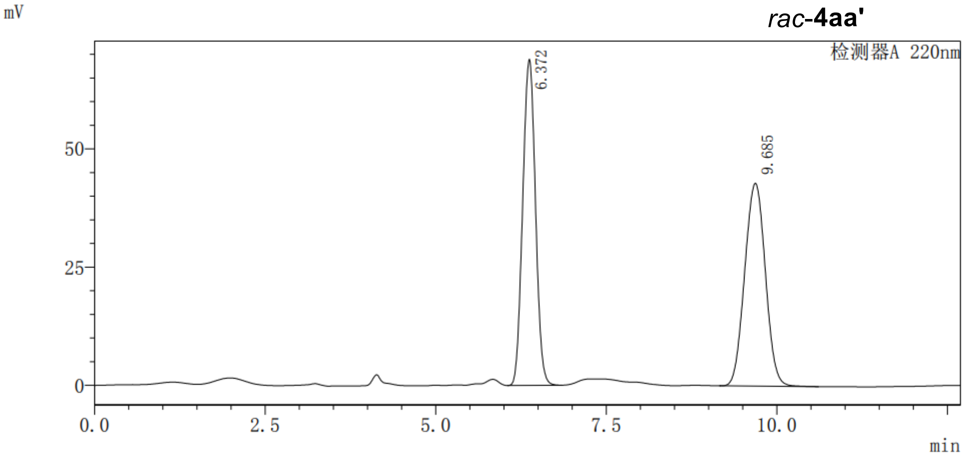

峰表

检测器A 220nm

峰号	保留时间	面积	高度	标记	面积%
1	6.372	898327	68931		49.832
2	9.685	904387	42913		50.168
总计		1802714	111844		100.000

分析日期　　: 2021/6/23 21:07:12
处理日期　　: 2021/6/23 21:18:21
描述　　　　: OD-H, n-hexane/iPrOH=95/5, 1.0 ml/min, 220 nm

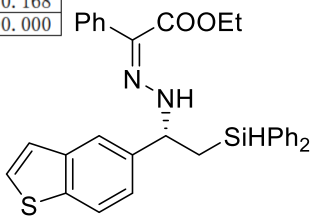

S-**4aa'**

色谱图

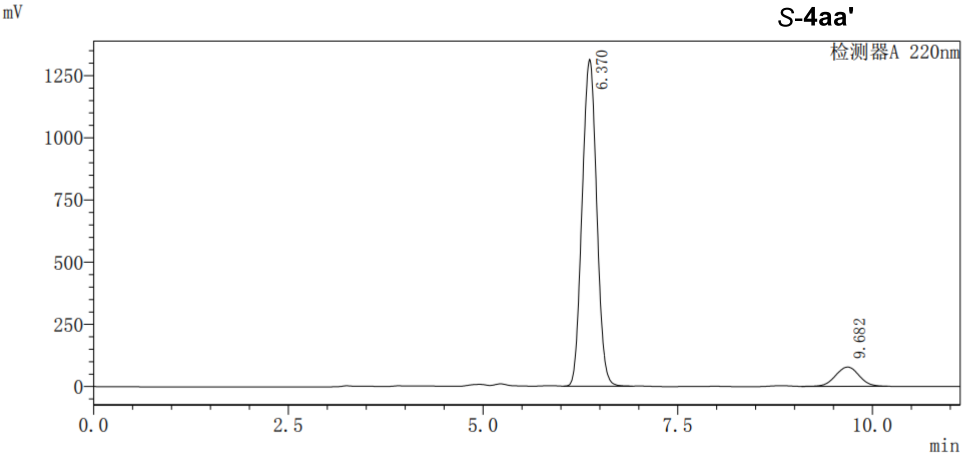

峰表

检测器A 220nm

峰号	保留时间	面积	高度	标记	面积%
1	6.370	17111796	1313512		91.249
2	9.682	1641037	77556	M	8.751
总计		18752833	1391068		100.000

Supplementary Figure 143. HPLC spectra of *S*-**4aa’**, related Table 3.

Supplementary References

(1) Revunova, K.; Nikonov, G. I., *Chem. Eur. J.* **2014**, 20, 839-845.

(2) Neumeier, M.; Chakraborty, U.; Schaarschmidt, D.; de la Pena O'Shea, V.; Perez-Ruiz, R.; Jacobi von Wangelin, A., *Angew. Chem., Int. Ed.* **2020**, 59, 13473-13478.

(3) Cui, J.; Wong, Y.-L.; Zeller, M.; Hunter, A. D.; Xu, Z., *Angew. Chem., Int. Ed.* **2014**, 53, 14438-14442.

(4) Guo, J.; Wang, H.; Xing, S.; Hong, X.; Lu, Z., *Chem* **2019**, 5, 881-895.

(5) Shimasaki, T.; Okajima, S.; Ishikawa, R.; Kawaguchi, S.; Akimoto, T.; Asano, N.; Iwanaga, T.; Watanabe, M.; Teramoto, N.; Shibata, M., *Tetrahedron* **2018**, 74, 2454-2465.

(6) Bao, H.; Zhou, B.; Luo, S.-P.; Xu, Z.; Jin, H.; Liu, Y., *ACS Catal.* **2020**, 10, 7563-7572.

(7) Guo, J.; Shen, X.; Lu, Z., *Angew. Chem., Int. Ed.* **2017**, 56, 615-618.

(8) Guo, J.; Lu, Z., *Angew. Chem., Int. Ed.* **2016**, 55, 10835-10838.

(9) Lin, Y.; Song, Q., *Eur. J. Org. Chem.* **2016**, 2016, 3056-3059.

(10) Chen, J.; Shen, X.; Lu, Z., *J. Am. Chem. Soc.* **2020**, 142, 14455-14460.

(11) Zhao, J.; Cheng, B.; Chen, C.; Lu, Z., *Org. Lett.* **2020**, 22, 837-841.

(12) Carney, J. R.; Dillon, B. R.; Campbell, L.; Thomas, S. P., *Angew. Chem., Int. Ed.* **2018**, 57, 10620-10624.

(13) Kennedy, C. R.; Zhong, H.; Macaulay, R. L.; Chirik, P. J., *J. Am. Chem. Soc.* **2019**, 141, 8557-8573.

(14) Garcia, K. J.; Gilbert, M. M.; Weix, D. J., *J. Am. Chem. Soc.* **2019**, 141, 1823-1827.

(15) Chen, X.; Cheng, Z.; Guo, J.; Lu, Z., *Nat. Commun.* **2018**, 9, 3939.

(16) Cheng, B.; Lu, P.; Zhang, H.; Cheng, X.; Lu, Z., *J. Am. Chem. Soc.* **2017**, 139, 9439-9442.
